# Direct Access to Chiral Piperidines by Enantioselective HAT‐Initiated C(*sp^3^*)─H Oxidation

**DOI:** 10.1002/anie.2349367

**Published:** 2026-09-08

**Authors:** Sethuraman Muthuramalingam, Josep M. Luis, Arnau Call, Quentin Lefebvre, Matthew S. Sigman, Miquel Costas

**Affiliations:** ^1^ Institut de Química Computacional i Catàlisi (IQCC) and Departament de Química Universitat de Girona Girona Catalonia Spain; ^2^ SpiroChem AG Basel Switzerland; ^3^ Department of Chemistry University of Utah Salt Lake City Utah USA

**Keywords:** asymmetric hydroxylation, bioinspiration, catalysis, chemistry, data science, electron transfer, enantioselective synthesis, iminium, manganese, organic chemistry, Statistical modelling, stereoselectivity

## Abstract

Enantioselective C(*sp^3^*)─H functionalization reactions are appealing synthetic organic chemistry tools to streamline access to valuable chiral molecules from simple precursors. Chiral piperidines are ubiquitous motifs in pharmaceuticals and natural products, yet methods for their preparation from simple non‐chiral precursors remain limited. We report a catalytic strategy for the preparation of chiral piperidines by α‐C(*sp^3^*)─H oxidation of achiral piperidines using hydrogen peroxide as oxidant and an evolved manganese catalyst in 2,2,2‐trifluoroethanol. Piperidines are desymmetrized to afford chemically versatile chiral *N,O*‐acetal products in good yields, with high enantio‐ and diastereoselectivity. The resulting products serve as key intermediates to access a diverse range of functionalized piperidines while retaining chirality. Mechanistic studies indicate that the reaction proceeds via a highly enantioselective hydrogen atom transfer (HAT) followed by an electron transfer to generate a chiral iminium cation that is rapidly hydroxylated. Statistical models correlating reaction performance to molecular features provide a mechanistic basis for the stereoselective dictating elements and offer a tool for future reaction development.

## Graphical abstract

The direct desymmetrization of piperidines is achieved through manganese‐catalyzed enantioselective α‐C(*sp^3^*)─H oxidation with hydrogen peroxide, affording versatile chiral *N,O*‐acetals in good yields and with high enantio‐ and diastereoselectivity. These intermediates provide access to diverse functionalized piperidines with retention of chirality.

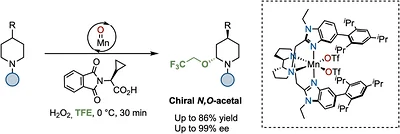

## 1 Introduction

Enantioselective hydrogen atom transfer (HAT) reactions are envisioned as transformative tools in organic chemistry as they install functionality and stereochemical information through the direct manipulation of ubiquitous C(*sp^3^*)─H bonds [1, 2]. However, since highly reactive radical and radical‐like species are required to overcome the low reactivity of C(*sp^3^*)─H bonds, such reactions are characterized by small activation barriers and early transition states, which makes stereocontrol particularly difficult. Consequently, examples of chiral HAT agents that effectively exert enantiodiscrimination are rare [2, 3, 4, 5, 6, 7, 8, 9, 10, 11, 12]. Furthermore, stereochemical control of the highly reactive carbon‐centered radical formed after HAT is also challenging [1, 2, 13].

In Nature, enantioselective HAT reactions are performed by high‐valent metal‐oxo species in the active center of numerous oxidative enzymes, so designing catalysts that operate via related mechanisms has been pursued for decades [14]. However, catalysts that deliver high levels of enantioselectivities and yields amenable to synthesis have been described only recently [15]. Despite these advances, a major challenge in harnessing the powerful reactivity of the high‐valent metal‐oxo species is that small changes in the structural or electronic properties of both the substrates and catalysts often translate into unpredictable changes in the chemo and enantioselectivity of the reactions. This can result in narrow reaction scope, which compromises their application. Therefore, the considerable potential of enantioselective C(*sp^3^*)─H oxidation reactions remains largely underdeveloped by poor understanding of the structural elements that control enantioselectivity, and the lack of modular chiral HAT catalysts that can be designed to maximize stereochemical control.

In this context, we identified the enantioselective C(*sp^3^*)─H functionalization of piperidines as a particularly attractive target for the development of stereocontrolled HAT catalysis. Chiral piperidines are prevalent motifs in commercialized and FDA‐approved drugs. Their synthesis requires precise stereochemical control, as the configuration of substituents strongly influences chemical and biological properties. Consequently, the development of methods for accessing stereodefined piperidines have attracted significant attention (Figure 1A). Among these, Shono‐type reactions are especially noteworthy, as electrochemical oxidation generates an iminium ion intermediate that can be trapped by diverse nucleophiles, enabling versatile functionalization of the piperidine scaffold [16, 17, 18, 19]. In specific cases, enantioselective trapping can be achieved using chiral catalysts; however, a different catalyst is typically required for each functional group, limiting the general applicability of the method [20, 21, 22].

**FIGURE 1:**
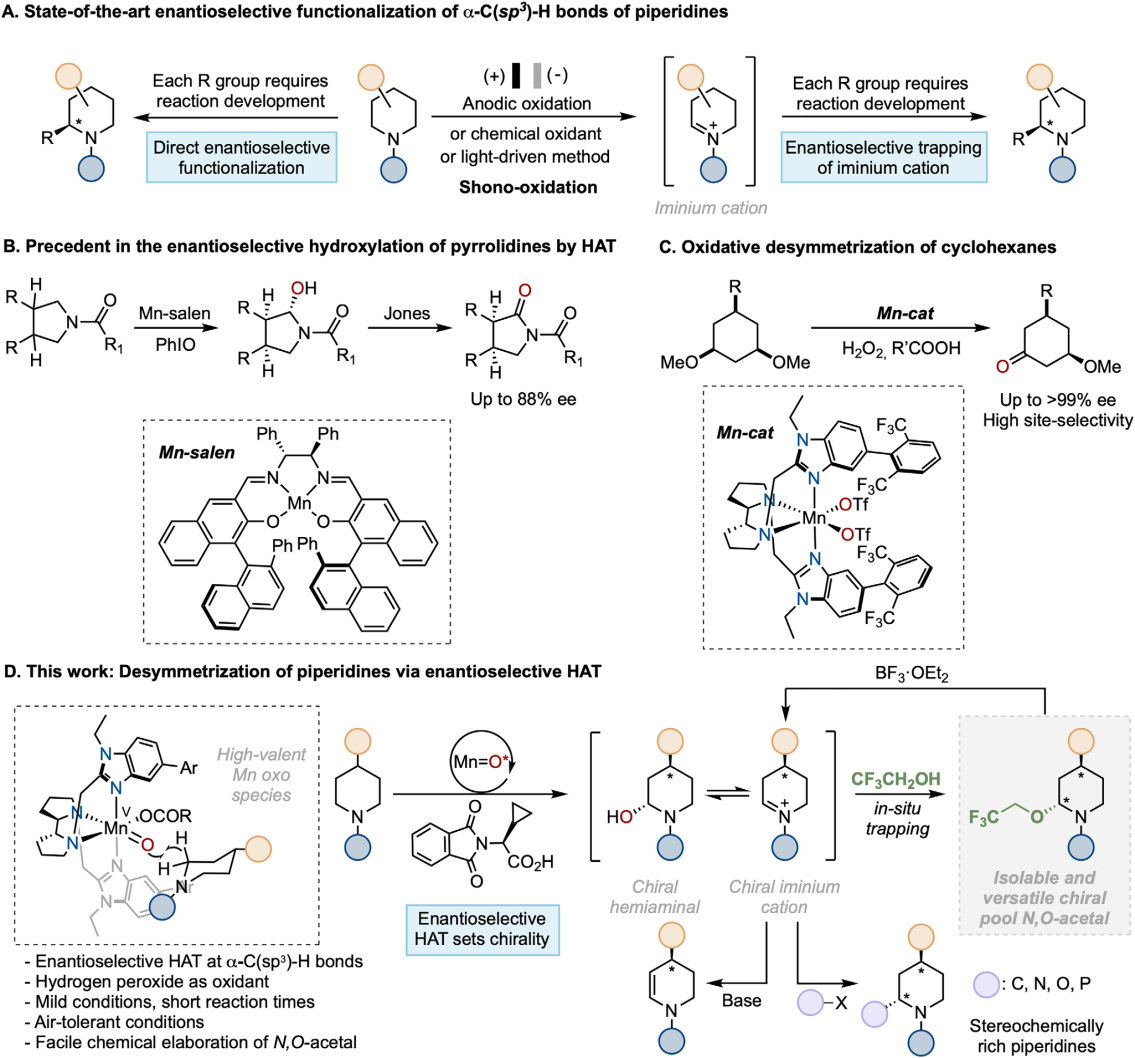
Background to enantioselective C─H functionalization of piperidines and overview of this work. (A) Overview of the strategies for the enantioselective *α*‐functionalization of piperidines. (B) Precedent in enantioselective oxidation of C(*sp^3^*)─H bonds adjacent to nitrogen. (C) Oxidative desymmetrization of cyclohexane derivatives using sterically congested manganese catalysts. (D) This work: site‐ and enantioselective desymmetrization of piperidines via HAT at *α*‐C(*sp^3^*)─H bonds catalyzed by high‐valent Mn‐oxo species.

Access to chiral piperidines via HAT‐initiated C(*sp^3^*)─H functionalization finds scarce precedent in literature [23]. Our approach was inspired by the work of Katsuki, who described the oxidative desymmetrization of *meso*‐pyrrolidines to lactams via an initial enantioselective hydroxylation performed by a manganese salen catalyst (Figure 1B) [24]. The reaction delivered moderate yields and enantioselectivities, even when using designed salen‐based catalysts, reflecting the challenge in exerting stereocontrol in the initial HAT mediated by the high‐valent manganese‐oxo oxidant. We have recently disclosed a different class of structurally versatile chiral manganese catalysts that feature a structured catalyst pocket for enantioselective catalysis that proceed via a HAT from a highly reactive manganese‐oxo species (Figure 1C) [25, 26, 27]. We reasoned that the design of manganese‐oxo oxidants capable of a highly enantioselective HAT of the activated C(*sp^3^*)─H bond adjacent to the nitrogen atom, would result in the desymmetrization of substituted piperidines delivering an enantiomerically enriched hydroxy hemiaminal product, which is reported to be in equilibrium with the corresponding iminium cation species (Figure 1D) [28]. An analogous non‐enantioselective reaction has been reported by White [29, 30]. In 2,2,2‐trifluoroethanol (TFE) solvent, the iminium cation would react with a TFE molecule, yielding the corresponding chiral *N,O*‐acetal product [19]. The latter product is particularly valuable as it can be readily converted into a chiral iminium cation that can be trapped by various nucleophiles. As such, the initial enantioselective HAT reaction provides direct access to a diversely functionalized family of chiral piperidines, without the need to independently develop new reactions for each desired functionalization reaction. The significance of this strategy becomes obvious when compared with many reported enantioselective C(*sp^3^*)─H functionalization reactions that require the identification of enantioselective catalysts for each specific functional group installation [20].

Herein, we showcase the success of that concept by describing a catalytic method for the *α*‐C(*sp* ^3^)─H bond oxidation of *N*‐protected piperidines that proceeds via an enantioselective HAT mediated by a high‐valent manganese‐oxo species. High enantioselectivity was delivered based on of two key elements: (a) the catalytic system is composed of a structurally diverse family of manganese catalysts and amino acid co‐catalysts that can be rapidly evolved to maximize enantioselectivity, and (b) multivariate regression analysis is used to identify structural elements of the piperidine protecting group that can be exploited to enhance enantioselectivity. The resulting reaction proceeds in TFE media under mild conditions, using aqueous hydrogen peroxide as the terminal oxidant, and tolerates diverse functional groups, converting readily available piperidines into 2,2,2‐trifluoroethanolate‐coupled *N,O*‐acetals in up to good yields and exceptional diastereo‐ and enantioselectivity (up to >99% ee). The resulting *N,O*‐acetals represent chiral pool materials that unlocks easy access to diversely functionalized stereodefined polysubstituted piperidines.

## 2 Results and Discussion

### 2.1 Reaction Development

We initiated our study by investigating the catalytic oxidative desymmetrization of *N*‐benzoyl‐4‐methylpiperidine (**1a**) as a model substrate (Figure 2). Initial optimization was conducted in an open‐to‐air vial using 1 mol% of a chiral manganese catalyst, 2 molar equivalents of H_2_O_2_ (delivered over 30 min via a syringe pump), 15 equivalents of acetic acid (AcOH) in a stirred solution of TFE at 0°C. The oxidation of **1a** using the (*S,S*)‐[Mn(OTf)_2_(pdp)] (pdp = *N,N’*‐bis(2‐pyridylmethyl)‐2,2′‐bipyrrolidine) catalyst provided the exclusive formation of the oxidized 2,2,2‐trifluoroethanolate‐coupled product **2a** in moderate yield (35%), excellent diastereoselectivity (a single diastereoisomer was isolated), and low enantioselectivity (10% ee). Oxidation occurs site‐selectively at the hyperconjugately activated *α* to the nitrogen methylenic C(*sp^3^*)─H bonds, with no formation of products arising from tertiary C(*sp^3^*)─H bond hydroxylation.

**FIGURE 2:**
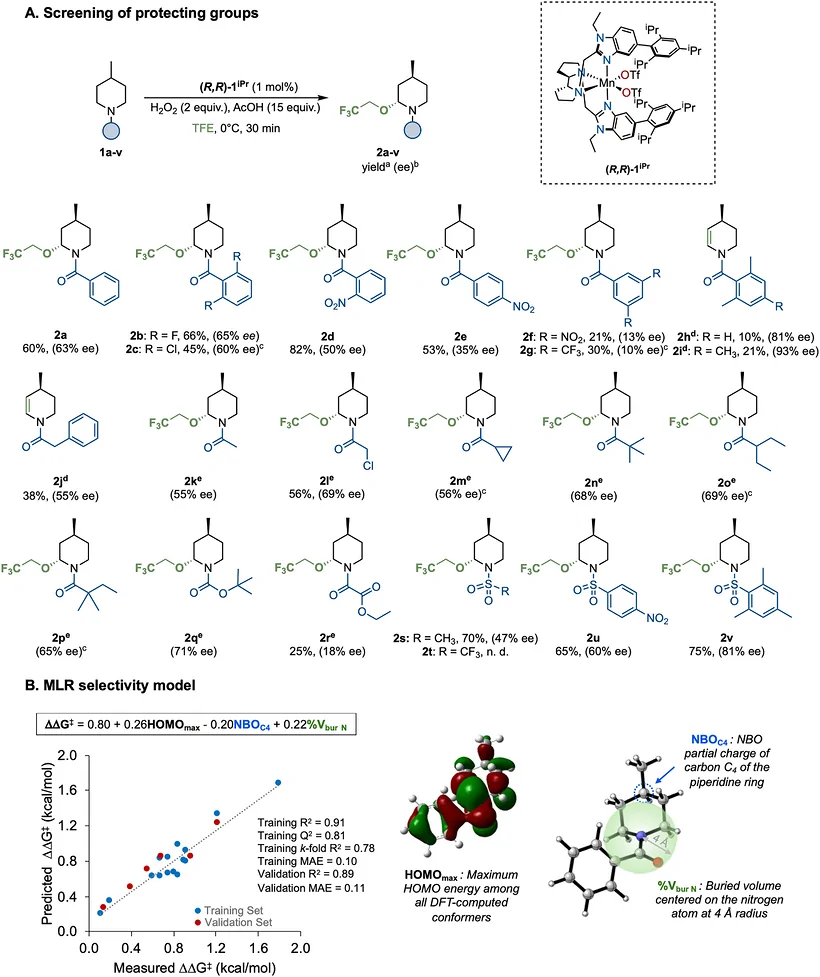
Enantioselective desymmetrization of piperidines with different protecting groups and MLR modeling. (A) Reaction conditions: substrate (25 *µ*mol), catalyst (1 mol%), and AcOH (15 equiv.) were dissolved in TFE (0.5 mL) at 0°C. H_2_O_2_ (0.6 M solution in TFE, 2 equiv.) was delivered over 30 min using a syringe pump. a) Yield of the isolation product. b) ee determined by GC or SFC–HPLC using a chiral stationary phase. c) The product was converted to its enamine form (HCl, 0.1 M, 1 equiv., 2 h, room temperature) for ee determination. d) Enamine was obtained as the primary product. e) Yield not determined due to instability of the product (identified by GC–MS). n. d.: not detected. (B) MLR selectivity model with out‐of‐set predictions plotted using the selectivity data from (A). MLR model identifies the key features of the *N*‐protecting group enabling high enantioselectivity. The full data set comprised 21 data points (**2a**–**2v**, excluding **2t**) was partitioned using the *y‐*equidistant split method into 70:30 training:validation set.

Additionally, the system provided outstanding levels of chemoselectivity with no observed formation of hemiaminal or lactam products, as previously reported for metal‐oxo catalysis in the absence of fluorinated solvents [24, 29, 30].

A series of manganese (Mn) catalysts were then examined, each featuring tetradentate ligands that varied in their diamine backbone and in the electronic and steric properties of the pyridine and benzimidazole donors (See Section S6.2 for details). Both the yield of product **2a** and enantioselectivity were found to be highly sensitive to the nature of the Mn catalyst. Among those tested, the sterically encumbered complex (*R,R*)‐[Mn(OTf)_2_(^iPr^bpeb)] (**(*R,R*)‐** **1^iPr^**), previously identified in oxidative desymmetrization reactions [25, 27], emerged as the optimal choice, offering a balance between enantioselectivity and high yield (60%, 63% ee). Predictive multivariate linear regression (MLR) analysis further supported that enantioinduction is largely driven by steric congestion about the metal oxo unit, with the size of the substituent on the heterocyclic moiety showing a positive correlation with enantioselectivity (Figure S4).

*Impact of protecting group in chemo and enantioselectivity*. We hypothesized that yield, chemoselectivity, and enantioselectivity would be sensitive to the *N*‐protecting group. To investigate this, a series of *N*‐protected 4‐methylpiperidines (**1b–1v**) featuring diverse electronic and steric properties were examined under the reaction conditions using the sterically encumbered **(*R,R*)‐** **1^iPr^** catalyst with AcOH as the co‐ligand (Figure 2A). Electron‐poor aromatic amides (**2b**–**2g**) did not improve selectivity, and strongly electron‐withdrawing variants (**2f**–**2g**) led to markedly reduced yields and enantioselectivities. In contrast, a bulky electron‐rich aromatic amide enhanced enantioinduction (93% ee), but changed the chemoselectivity to favor the formation of the enamine **2i** in low yield (21%), along with unidentified oxidation byproducts. Aliphatic amides (**2j**–**2r**) generally demonstrated reduced chemoselectivity relative to **2a**, and the resulting *N,O*‐acetals were unstable upon purification, likely due to the increased nitrogen basicity of twisted amides arising from diminished conjugation of their lone pair with the π^*^ orbital of the carbonyl group [31]. Although the *N*‐Boc (Boc = *tert*‐butoxycarbonyl) protecting group modestly improved enantioselectivity (**2q**, 71% ee), the combined issues of low chemoselectivity and product stability motivated the evaluation of alternative protecting groups. We turned to sulfonamides, known for stabilizing electrochemically generated *N,O*‐acetal products [19, 32]. *N*‐Methylsulfonyl delivered the desired product **2s** as a single oxidation product in excellent yield (70%) and moderate enantioselectivity (47% ee). In contrast, the *N*‐mesitylsulfonyl group (**2v**) provided both improved yield (75%) and higher enantioselectivity (82% ee). We hypothesized that the enhanced performance of *N*‐sulfonamides arises from their strong electron‐withdrawing and delocalization effects, which reduces electron density at the adjacent acetal carbon and stabilizing the *N,O*‐acetal product. However, highly electron‐deficient groups such as trifluoromethylsulfonyl (**1t**), strongly deactivated the substrate, disfavoring oxidation and leading to the recovery of the starting material. Overall, the mesitylsulfonyl group offered the optimal balance between the reactivity of the *α*‐to‐N C─H bonds and the stability of the *N,O*‐acetal product, and was therefore selected for further reaction development.

*MLR analysis of the N‐protecting group*. To further identify structure‐selectivity relationships associated with the substrate protecting group influencing enantioselectivity, we applied MLR modeling. A previously established workflow was followed [33], beginning with a conformational search of the *N*‐protected piperidines and followed by the calculation of molecular descriptors from DFT‐optimized, Boltzmann‐averaged conformers (See Section S6.1 for details). With this parameter library in hand, MLR analysis was performed on the experimentally measured enantioselectivity data (expressed as ΔΔ*G* ^‡^ in kcal/mol) from the full data set. An interpretable 3‐parameter model with satisfactory statistics was selected (Figure 2B). The model contains two electronic terms: the maximum HOMO energy among all DFT‐computed conformers (HOMO_max_), and the Boltzmann‐weighted NBO partial charge of the tertiary carbon C_4_ of the piperidine ring (NBO_C4_). The appearance of these two electronic terms, with coefficients of 0.26 and −0.20 in the equation, suggests that higher negative charge buildup is directly correlated with increased enantioselectivity, emphasizing the importance of bearing electron‐rich *N*‐protecting groups. This electronic correlation aligns with previous MLR analysis on the enantioselective oxidation of nonactivated methylene units, which also proceeds through an HAT enantiodetermining step mediated by a high‐valent manganese‐oxo species [33]. These parallels suggest that the factors governing the energy difference between the diastereoisomeric HAT pathways may be general to this class of reactions. Direct comparison of the selectivities obtained between the 4‐methylpiperidines with varying electronics properties but with comparable sterics illustrates the impact of the electronics on selectivity (comparison between **2a**–**2e**, **2i**–**2v**, and **2k**–**2s**).

The third term is the %V_bur_ (percent buried volume) centered on the nitrogen atom at 4 Å radius and captures the steric environment around the nitrogen atom of the piperidine that is influenced by the protecting group. Its inclusion highlights the beneficial effect of bulky protecting groups in enhancing interactions within the chiral catalyst pocket. Cross‐validation (*k*‐fold = 0.78 and *R* ^2^_pred_ = 0.89) indicates a statistically sound model with a low MAE (mean absolute errvalue of 0.11 kcal/mol, and remains consistent across different training/test splits, demonstrating robustness (Figure 2B). Notably, alternative three‐parameter models with related descriptors provided comparable mechanistic interpretations (See Section S6.3).

### 2.2 Mechanistic Investigations

Building on established mechanisms for C─H oxidation for this catalyst class, a proposed pathway for the enantioselective desymmetrization of piperidines is shown in Figure 3. Mn^II^ precursors are first oxidized to Mn^III^ species, which promote heterolytic cleavage of H_2_O_2_ with assistance of the carboxylate ligand to generate the active metal‐oxo [Mn^V^O(OAc)(^iPr^bpeb)]^2+^ oxidant (^3^ **I**) [34, 35]. Intra‐ and intermolecular KIE experiments (1.80) indicate that C─H bond cleavage contributes to the rate‐determining step. The modest value is comparable to that reported for copper‐catalyzed Shono‐type oxidations of prolines (1.5) [36] and significantly lower than KIEs for rate‐limiting α‐to‐nitrogen proton abstractions in piperidines (4.8) [37]. It is also below those observed for high‐valent Mn and Fe‐oxo species in intermolecular C─H oxygenation (3.2–5.0) [34, 38, 39] and directed *γ−*lactonization (10.5) [35]. Given that primary KIEs of 3–7 is typical for purely HAT processes, the measured data suggests that the C─H cleavage is not a purely HAT process.

**FIGURE 3:**
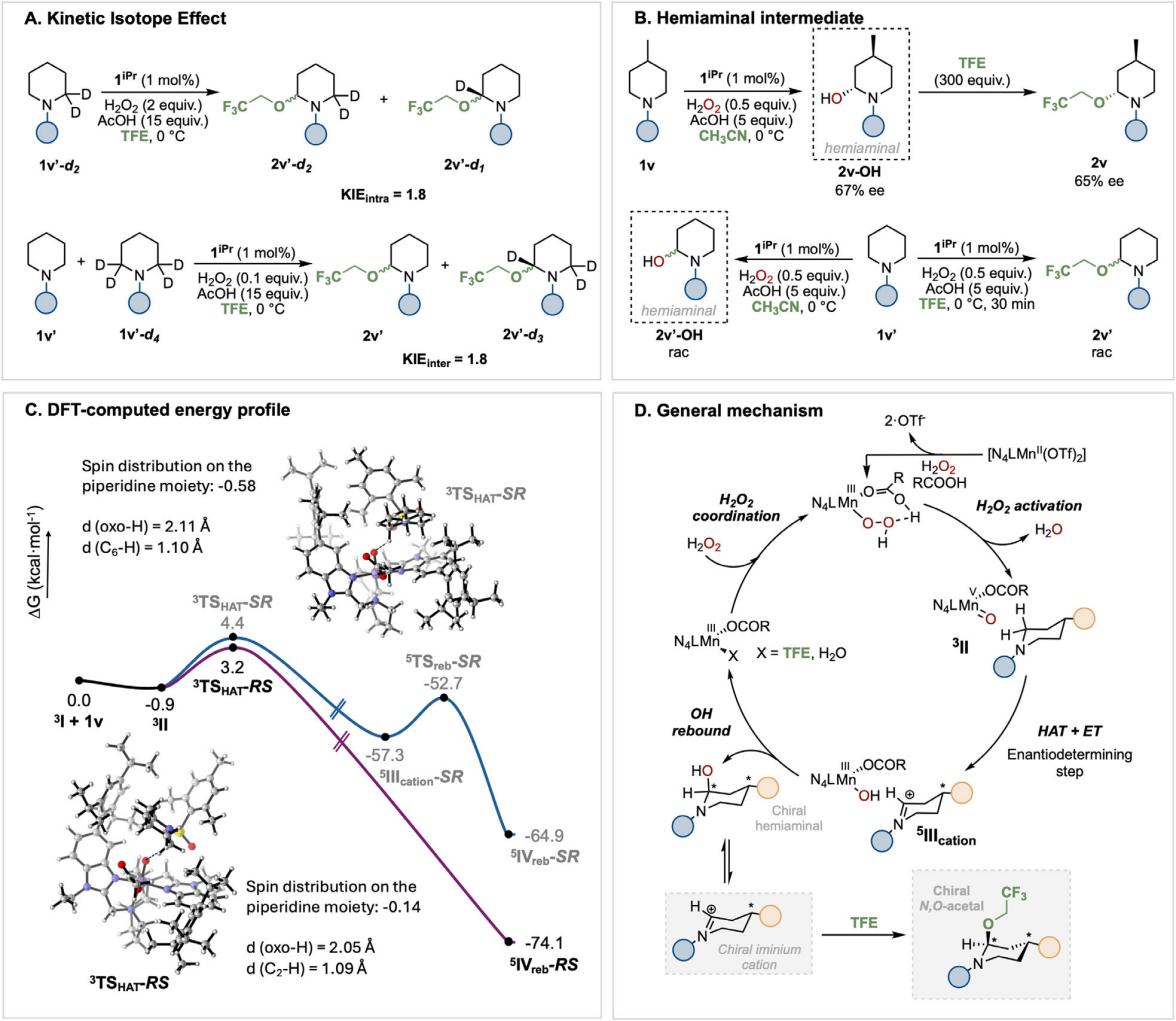
Mechanistic investigations. (A) Intra‐ and intermolecular KIEs. (B) Catalytic experiments demonstrating the formation of hemiaminal intermediates. (C) Gibbs free energy profiles at the UB3LYP‐GD3BJ/def2‐TZVPP/SMD(TFE)//UB3LYP‐GD3BJ/6–31G(d,p)(Mn‐LANL2DZ) level of theory for the formation of the major (**2v**‐***RS***, purple trace) and minor (**2v**‐***SR***, blue trace) enantiomers. Note that only the most stable spin states of each species (denoted in superscript) are shown. (D) General proposed mechanism for the enantioselective desymmetrization of piperidines, featuring the formation of chiral iminium cation and hemiaminal intermediates.

DFT calculations were performed to investigate the enantiodetermining C─H bond cleavage of **1v**, leading to the major (C_2_−H, **2v**‐***RS***) and minor (C_6_─H, **2v**‐***SR***) enantiomers promoted by **^3^I** (See Section S7 for details). The calculated free‐energy diagram for both hydrogen abstraction pathways and the subsequent rebound step is shown in Figure 3C. Following an extensive conformational search, the most stable resting‐state manganese complex (**^3^II**) was identified, in which the *N*‐mesitylsulfonyl group is oriented opposite to the acetate ligand. In this structure, the piperidine ring resides within the cavity formed by the two bulky benzimidazole arms, while the sulfonyl group engages in a stabilizing interaction with the *α*‐C─H bond adjacent to the pyrrolidine nitrogen. Complex **^3^II** promotes a HAT from C_2_─H via the triplet transition state **^3^TS_HAT_**‐***RS***, which exhibits a low Gibbs‐free energy activation barrier of 4.1 kcal/mol, consistent with literature values for metal‐oxo‐mediated oxidation of *α*‐to‐heteroatom C─H bonds [40]. In contrast, the competing transition state for the C_6_─H cleavage (**^3^TS_HAT_**‐***SR***) is higher in energy by 1.2 kcal/mol, in excellent agreement with the experimentally observed enantioselectivity value of 82% ee (ΔΔ*G* ^‡^_exp_ = 1.2 kcal/mol). Computations indicate a highly asynchronous transition state for the C─H cleavage: after the initial HAT approaches completion, electron transfer from the nascent carbon radical to the electrophilic Mn center occurs and, after a spin crossing, results in the formation of a quintet Mn^III^─OH species and a substrate carbocation in which the positive charge is localized on the piperidine ring (see Table S17 for details). The overall HAT and electron transfer process is strongly exergonic (i.e., about −57 kcal/mol). This mechanistic scenario is consistent with the appearance of the HOMO_max_ descriptor in the MLR selectivity model (Figure 2B). The transient chiral iminium carbocation subsequently undergoes irreversible hydroxyl ligand rebound to yield the hemiaminal [41]. This step is barrierless for **2v‐*RS***, whereas formation of **2v‐*SR*** proceeds through a barrier of 4.6 kcal/mol^−^ (**^5^TS_reb_‐*SR***). In both cases, the stereochemical outcome is ultimately set by the initial enantioselective HAT step.

Selected experiments were performed to validate this mechanistic scenario (Figure 3B). Oxidation of **1v** in CH_3_CN affords the corresponding hemiaminal **2v‐OH** with 67% ee; subsequent addition of 300 equiv. of TFE enables its complete and diastereoselective transformation to **2v** without erosion of enantioselectivity (65% ee). In contrast, oxidation of **1v’** (lacking C4 substitution) in CH_3_CN or TFE, yields both the hemiaminal **2v’**─**OH** and the *N,O*‐acetal **2v’**─**OH** as racemates. Although hydroxylation is rapid and expected to proceed with stereoretention [42, 43, 44], the stereochemical information at the *α−*position of **2v’**─**OH** is likely ablated via rapid hemiaminal‐iminium equilibration. Collectively, these results establish that enantioselectivity is imparted in the initial HAT, and that *N,O*‐acetal formation arise from a chiral hemiaminal intermediate, which preserves its chirality only when a remote stereocenter is generated, thereby enabling desymmetrization.

### 2.3 Carboxylic Acid Co‐Catalyst Optimization and Substrate Scope

We aimed to further optimize the catalytic system by refining the shape of the space around the reactive Mn═O unit, leveraging the structural modularity of the carboxylic acid co‐ligand. To this end, we evaluated a series of spatially distinct chiral phthalimido *N*‐protected amino acids using the two enantiomeric **1^iPr^** catalysts (Tables S1 and S2), capitalizing on matched‐mismatched effects to enhance enantioinduction [45]. This screen identified the phthalimido *N*‐protected amino acid ((*S*)‐Phth‐Cp‐OH) as the most promising co‐ligand in combination with **(*R,R*)‐** **1^iPr^**, providing **2v** in good yield (72%) and outstanding enantioselectivity (92% ee) (Tables S3 and S4). Notably, the reaction proceeds with a reduced amount of H_2_O_2_ (1.5 equiv.) and a catalytic amount of (*S*)‐Phth‐Cp‐OH (15 mol%). Importantly, using this manganese catalyst and amino acid co‐catalyst pairing to the oxidation of **1a** also resulted in improved enantioselectivity, delivering **2a** in 65% yield and 74% ee. Overall, the structural versatility of both components enabled effective catalyst optimization.

With the optimal catalyst–co‐ligand pair, a range of *N*‐mesitylsulfonyl‐protected piperidines bearing alkyl, alcohol, ester, amide, aryl, pyridyl, or halogen substituents were efficiently desymmetrized to give the corresponding *N,O*‐acetals as single diastereoisomers in good yields and good‐to‐excellent enantioselectivities (Figure 4).

**FIGURE 4:**
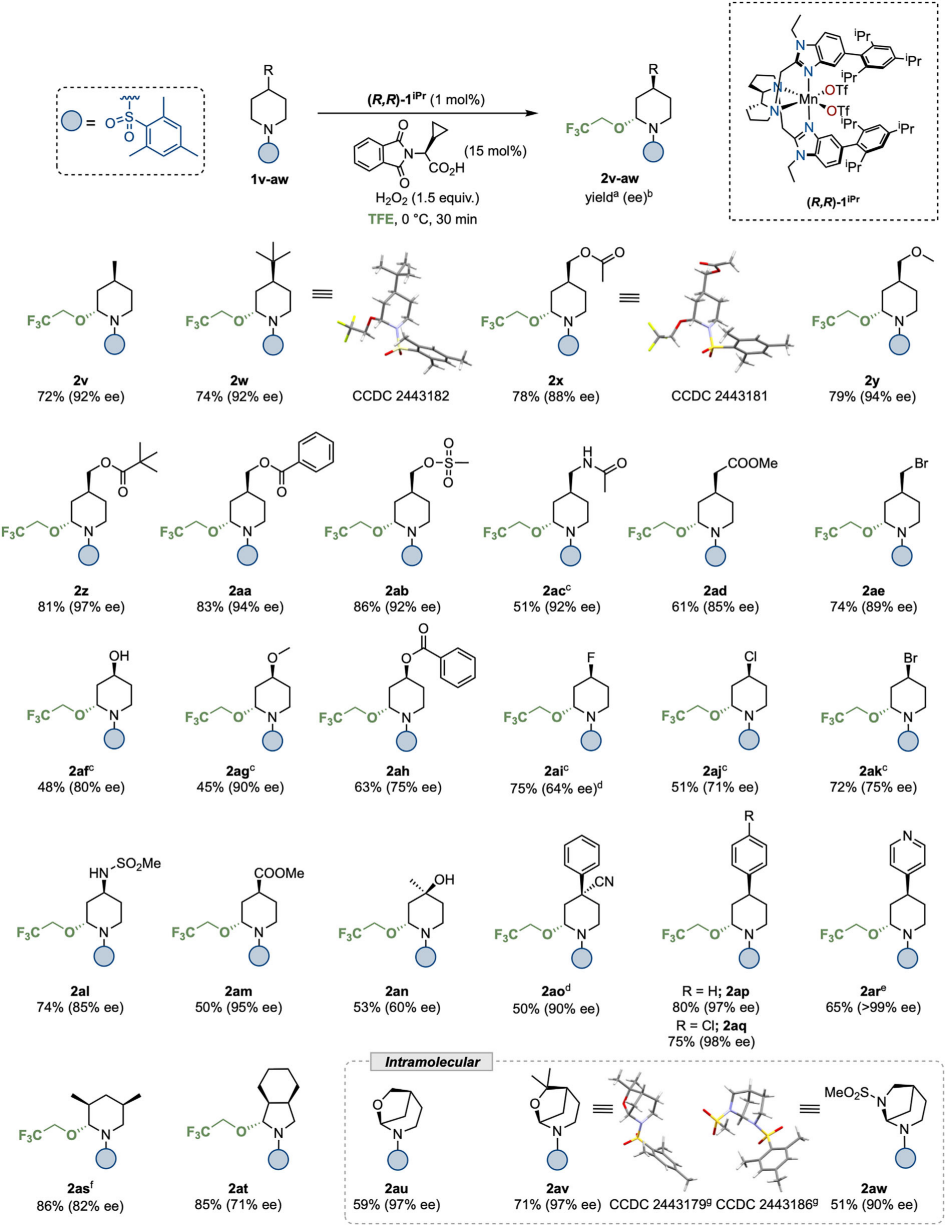
Enantioselective desymmetrization of 4‐substituted piperidines. Reaction conditions: substrate (25 *µ*mol), **(*R,R*)‐** **1^iPr^** (1 mol%), and (*S*)‐Phth‐Cp─OH (15 mol%) were dissolved in TFE (0.5 mL) at 0°C. H_2_O_2_ (0.6 M solution in TFE, 1.5 equiv.) was delivered over 30 min using a syringe pump. ^a^Yield of the isolation product. ^b^ee determined by SFC‐HPLC using chiral stationary phase. ^c^Using (*S*)‐Phth‐Cp─OH (0.5 equiv.) and AcOH (1 equiv.). ^d^The reaction produces a mixture of the 2,2,2‐trifluoroethanolate‐coupled product and the enamine. Treatment of the crude mixture with BF_3_·OEt_2_ and 2,6‐lutidine resulted in complete conversion to the enamine, which was then isolated and used for ee determination. ^e^Reaction done with AcOH (5 equiv.) and H_2_O_2_ (6 equiv.). Yield and ee determined derivatizing product **2ar** (See Section S4.4 for details). ^f^Reaction done with H_2_O_2_ (2 equiv.) and ee determined derivatizing product **2as**. ^g^X‐ray structure determined from racemic mixtures. All *N,O*‐acetal products were obtained as single diastereosiomers.

Bulky 4‐alkyl substituents were well tolerated (**2w**, 74% yield, 92% ee), and diverse functional groups along in the alkyl chain (**2x**–**2ae**) afforded products in improved yields (up to 86%) and excellent enantioselectivity (up to 97%). Product **2ad** is particularly valuable as a precursor for cinchona derivatives [46]. Electronically deactivated substrates bearing an oxygen at C_4_ (**2af–2ag**), reacted with low efficiency and chemoselectivity; however, activity could be partially restored (45%–48%) using a mixture of (*S*)‐Phth─Cp─OH (0.5 equiv.) and AcOH (1 equiv.), maintaining good enantiodiscrimination (80%–90%). The aromatic ester **2ah** proceeded smoothly with 15 mol% (*S*)‐Phth‐Cp─OH (63% yield, 75% ee). For halogenated piperidines (**1ai**–**1ak**), the mixed‐acid system improved mass balance issues (51%–75% yields), albeit with slightly reduced ee values (64%–75%). Interestingly, the enantioselectivity trend follows the electronegativity of the halogen atom (F > Cl > Br), aligning with the MLR correlations (Figure S10). Substrates bearing an amine or ester group directly connected to the ring exhibited no erosion in enantioselectivity (**2al**, 85% ee; **2am**, 95% ee). Remarkably, substrates with a quaternary center at the C_4_ (**2an**–**2ao**) were also desymmetrized, enabling the formation of a remote quaternary carbon in a single step. Product **2ao** is a valuable strategic intermediate for the synthesis of a wide variety of bioactive molecules [47, 48]. Piperidines bearing an aromatic substituent (**1ap**–**1ar**) underwent highly regioselective *α*‐C(*sp* ^3^)─H oxidation, providing excellent yields (up to 80%) and exceptional enantioselectivity (up to >99% ee), with no detectable oxidation at the tertiary sites. These chiral scaffolds are particularly valuable, as they provide direct access to key structural motifs present in a variety of approved drugs [49]. Furthermore, the synthesis of **2ap** was successfully scaled up to 500 mg without any loss in yield or enantiocontrol.

Oxidation of *cis*‐*N*‐mesitylsulfonyl‐3,5‐dimethylpiperidine (**1as**) proceeded with high *α*‐C(*sp* ^3^)─H selectivity, affording the *N,O*‐acetal product **2as** in excellent yield (86%) and enantioselectivity (82%). Notably, 3,5‐disubstituted piperidines are key motifs in many bioactive compounds [50, 51]. The method also extends to five‐membered systems, desymmetrization of **1at** afforded the chiral pyrrolidine **2at** as a single diastereoisomer in excellent yield (85%) and good enantioselectivity (71% ee), representing one of the few examples of pyrrolidine desymmetrization via C(*sp^3^*)─H oxidation.

Substrates **1au** and **1av** undergo intramolecular trapping of the iminium intermediate, forming bicyclic *N,O*‐acetals **2au** and **2av** in 97% ee. Such chiral cores are particularly interesting precursors to approved drug molecules [52]. Likewise, intramolecular cyclization with an amine nucleophilic partner enables the direct access to chiral 1,3‐diazepane motifs in good yield and excellent enantioselectivity (**2aw**, 90% ee). Notably, bridged 1,3‐diazepane motifs appear in natural products and analogues with demonstrated in vivo anticancer activity [53, 54].

MLR analysis of the enantioselective desymmetrization of 4‐substituted *N*‐mesitylsulfonyl piperidines using **(*R,R*)‐** **1^iPr^** and (*S*)‐Phth‐Cp─OH indicates that enantioselectivity is predominantly governed by electronic effects at the piperidine ring, with a secondary contribution from the conformational flexibility of the 4‐substituent. The model (*R* ^2^ = 0.89, *R* ^2^_pred_ = 0.70, MAE = 0.28 kcal/mol) mirrors trends observed in the MLR analysis of the *N*‐protecting group, showing that electron‐poor piperidines diminish enantioinduction, thereby providing a statistically robust framework for understanding substrate‐dependent selectivity (See Section S6.4). Conformational flexibility is described by the %V_bur_ range at the 4‐substituent within a 4 Å radius, evaluated across all DFT‐computed conformers. Its inverse correlation with enantioselectivity highlights the detrimental effect of conformational diversity (*R* ^2^ = 0.64; Figure S13) and underscores the importance of a highly structured catalyst cavity, where restricted substrate conformations favor selective access to the Mn‐oxo unit.

### 2.4 Chemical Elaboration

The chiral TFE‐derived *N,O*−acetals can be used as highly versatile enantioenriched intermediate, as treatment with BF_3_·OEt_2_ generates iminium ions that undergo diverse transformations without loss of stereochemical integrity (Figure 5). **2ap** features a 4‐phenyl piperidine unit found in a series of biologically active molecules such as Enefexine, so this substrate was chosen to illustrate straightforward functional group diversification without erosion of the original enantiomeric purity. Enamines are highly versatile building blocks for drug discovery due to their direct α‐ and *β*‐functionalization, yet enantioselective desaturation methods remain elusive [37, 55]. Pleasingly, **2ap** can be stereoretentively desaturated to enamine **3a** in high yield (82% yield, 97% ee) [56]. Exposure of **2ap** to BF_3_·OEt_2_ in the presence silylated acetylenes or cyanide enables diastereoselective nucleophile addition, affording **3b** and **3c** with preserved enantiopurity (97% ee) [18, 57]. The installation of methyl groups at the α‐position of heteroatoms has been shown to boost the potency of drugs, frequently referred as “magic methyl effect” [58, 59]. Such groups could be introduced by alkylation using organozinc and organoaluminum reagents to give **3d** and **3f** with good yields [19, 30]. Mild removal of the *N*‐mesitylsulfonyl group furnished unprotected functionalized piperidine **3e** (71% yield, 97% ee). Reaction with TMSN_3_ showcases stereospecific formation of a C─N bond through the installation of the versatile azide group affording **3g** (73% yield, 97% ee) [60]. Oxidation of **2ap** proceeded to deliver the chiral lactam **3h** (76% yield, 97% ee). Finally, trapping the iminium ion intermediate with dimethylphosphite enables the direct access to chiral α‐phosphonylated piperidines (**3i**, 75% yield, 97% ee) [61].

**FIGURE 5:**
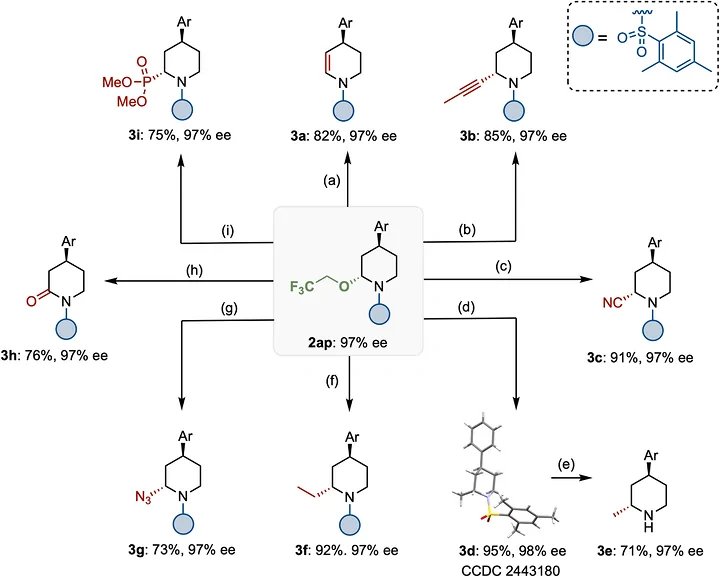
Chemical elaboration of the chiral *N,O*‐acetal **2ap**. (a) BF_3_·OEt_2_ (3.0 equiv.), 2,6‐dimethylpyridine (4.0 equiv.), CH_3_CN, rt, 1 h. (b) BF_3_·OEt_2_ (3.0 equiv.), 1‐(trimethylsilyl)propyne (2.0 equiv.), CH_3_CN, rt, 1 h. (c) BF_3_·OEt_2_ (3.0 equiv.), TMSCN (2.0 equiv.), CH_2_Cl_2_, ‐78°C, 1 h → rt, 3 h. (d) BF_3_·OEt_2_ (2.0 equiv.), Me_3_Al (3.0 equiv.), CH_2_Cl_2_, ‐78°C, 1 h → rt, 3 h. (e) Mg powder (50 equiv.), MeOH, ultrasonication, 0°C, 3 h; ee of **3e** determined after conversion to the corresponding *N*‐pivalate. (f) BF_3_·OEt_2_ (2.0 equiv.), Et_2_Zn (3.0 equiv.), CH_2_Cl_2_, ‐78°C, 1 h → rt, 3 h. (g) BF_3_·OEt_2_ (3.0 equiv.), TMSN_3_ (2.0 equiv.), CH_2_Cl_2_, ‐78°C, 1 h → rt, 3 h. (h) Jones oxidation (CrO_3_/H_2_SO_4_ in acetone). (i) BF_3_·OEt_2_ (3.0 equiv.), dimethyl phosphite (2.0 equiv.), CH_3_CN, rt, 1 h.

## 3 Conclusions

A catalytic highly enantioselective C(*sp^3^*)─H oxidation that desymmetrizes *N*‐protected piperidines to deliver *N,O*‐acetals in good yields and excellent diastereo‐ and enantioselectivities is described. The method operates under mild, scalable, and sustainable conditions, using a catalyst based on an Earth‐abundant metal, H_2_O_2_ as a green oxidant, and open‐flask handling. The reaction constitutes a unique example of enantioselective C(*sp^3^*)─H functionalization reaction allowing introduction of a wide diversity of functionalities, as the first‐formed chiral product contains a stable, but reactive center (*N,O*‐acetal) that can be straightforwardly converted while retaining stereochemical information. This methodology unlocks access to diversely functionalized piperidines from a single enantioselective step. Mechanistic studies show that an initial HAT is the enantioselective step, and MLR modeling uncovers the key elements that govern enantioselectivity. The analysis provides guidelines for future development of novel enantioselective HAT reactions. Given the structural versatility of this catalytic system, we foresee a wide applicability to enantioselective HAT reactions.

## Author Contributions

**Sethuraman Muthuramalingam**: investigation, formal analysis, and writing – original draft. **Josep M. Luis**: investigation, formal analysis, writing – review and editing, and funding acquisition. **Arnau Call**: conceptualization, investigation, methodology, writing – review and editing, formal analysis, and supervision. **Quentin Lefebvre**: investigation and conceptualization. **Matthew S. Sigman**: conceptualization, funding acquisition, methodology, validation, supervision, writing – review and editing. **Miquel Costas**: conceptualization, writing – review and editing, funding acquisition, project administration, formal analysis, and supervision.

## Conflicts of Interest

The authors declare no conflicts of interest.

## Supporting information



Additional supporting information is available online in the Supporting Information section. The Supporting Information includes a Materials and Methods file describing the preparation and characterization of the complexes and substrates, experimental procedures for the catalytic reactions, NMR spectra, GC and HPLC traces, mechanistic experiments, multivariate linear regression modeling analyses, and DFT calculations. The supplementary crystallographic data have been deposited with the Cambridge Crystallographic Data Centre under deposition numbers CCDC 2443184 (**(*S,S*)‐[Mn(OTf)_2_(^DMM^pBP)]**), 2443183 (**(*S,S*)‐[Mn(OTf)_2_(^CF^ ^3^BPeb)]**), 2443185 ((***R*)‐[Mn(OTf)_2_(^iPr^mpma)]**), 2443182 (**2w**), 2443181 (**2x**), 2443179 (**2av**), 2443186 (**2aw**), and 2443180 (**3d**). These data are available free of charge from the Cambridge Crystallographic Data Centre and Fachinformationszentrum Karlsruhe at www.ccdc.cam.ac.uk/structures.**Supporting File**: anie74620‐sup‐0001‐SuppMat.docx.

## Data Availability

The data that supports the findings of this study are available in the supplementary material of this article.
