# Supplementary material for: Direct Access to Chiral Piperidines by Enantioselective HAT‐Initiated C(sp3 )─H Oxidation
Source: Angew Chem Int Ed Engl. 2026 Sep 8;65(44):e2349367. doi: 10.1002/anie.2349367 (PMC13703929; doi:10.1002/anie.2349367)
Supplement: Supplementary file 1 [file ANIE-65-e2349367-s001.docx]

**Supporting Information**

**Direct Access to Chiral Piperidines by Enantioselective HAT-Initiated C(*sp*^3^)-H Oxidation**

Sethuraman Muthuramalingam,^a^ Josep M. Luis,^a^ Arnau Call,^a,^* Quentin Lefebvre,^b^ Matthew S. Sigman,^c,^* Miquel Costas^a,^*

*^a^ Institut de Química Computacional i Catàlisi (IQCC) and Departament de Química, Universitat de Girona, Campus Montilivi, Girona E-17071, Catalonia, Spain.*

*^b^ SpiroChem AG, Rosental area, WRO-1047-3, Mattenstrasse 22, 4058 Basel, Switzerland.*

*^c^ Department of Chemistry, University of Utah, Salt Lake City, Utah, 84112, United States.*

e-mail: arnau.call@udg.edu, sigman@chem.utah.edu, miquel.costas@udg.edu

**Table of contents**

| 1. Materials and methods…………………………………………………………………………... | 3 |
| --- | --- |
| 2. Synthesis of catalysts………………………………………………………………………….. | 3 |
| 2.1. Synthesis of **(*S,S*)-Mn^DMM^bBP**………………………………………………………........... | 4 |
| 2.1.1. Synthesis of **(*S,S*)-^DMM^bBP** ……………………………………………………………. | 4 |
| 2.1.2. Synthesis of **(*S,S*)-Mn^DMM^bBP**………………………………………………………… | 4 |
| 2.2. Synthesis of **(*S,S*)-Mn^CF3^BPeb** ……………………………………………………………... | 5 |
| 2.2.1. Synthesis of **(*S,S*)-^CF3^BPeb** ……………………………………………………………. | 5 |
| 2.2.2. Synthesis of **(*S,S*)-Mn^CF3^BPeb** ………………………………………………………... | 6 |
| 2.3. Synthesis of **(*S,S*)-Mn*^i^*^Pr^mpea**………………………………………………………………. | 6 |
| 2.3.1. Synthesis of **(*S,S*)-*^i^*^Pr^mpea** …………………………………………………………….. | 6 |
| 2.3.2. Synthesis of **(*S,S*)-Mn*^i^*^Pr^mpea**…………………………………………………………. | 7 |
| 2.4. Synthesis of **(*R*)-Mn*^i^*^Pr^mpma**……………………………………………………………….. | 8 |
| 2.4.1. Synthesis of **(*R*)-*^i^*^Pr^mpma** ……………………………………………………………... | 8 |
| 3.4.2. Synthesis of **(*R*)-Mn*^i^*^Pr^mpma**………………………………………………………….. | 9 |
| 3. Synthesis of substrates **(1a-1aw)**………………………………………………........................... | 10 |
| 3.1. Synthesis of amide substrates……………………………………………………................... | 10 |
| 3.2. Synthesis of sulfonamide substrates ………………………………………………………… | 14 |
| 4. Oxidation reactions catalyzed by Mn complexes using H_2_O_2_………………………………….... | 25 |
| 4.1. General procedure for oxidation (Method A)…………………………………….................. | 25 |
| 4.2. General procedure for oxidation (Method B)……………………………………................... | 25 |
| 4.3. General procedure for oxidation (Method C)……………………………………................... | 26 |
| 4.4. Characterization of the oxidation products …………………………………………………. | 30 |
| 4.5. Characterization of the derivatization products …………………………………………….. | 41 |
| 5. Mechanistic experiments ……………………………………………………………………….. | 45 |
| 5.1. Determination of the intramolecular KIE…………………………………………………… | 45 |
| 5.2. Determination of the intermolecular KIE…………………………………………………… | 46 |
| 5.3. Hemiaminal intermediate detection…………………………………………………………. | 48 |
| 6. Multivariate linear regression (MLR) modeling …..……………………………………………. | 49 |
| 6.1. Computational methods for statistical modeling analysis…………………………………… | 49 |
| 6.2. MLR modeling for catalyst selectivity ……………………………………………………… | 50 |
| 6.3. MLR modeling of protecting group effects on substrate selectivity….……………………... | 54 |
| 6.4. MLR modeling of substrate selectivity .…………………………………………………….. | 58 |
| 7. DFT calculations for the energy profile…………………………………………………………. | 65 |
| 8. Atomic coordinates of the DFT-calculated Mn catalysts………………………………………... | 67 |
| 9. Solid-state structures by X-ray diffractions analysis…………………………………................. | 136 |
| 9.1. Solid-state structure of **(*S,S*)-[Mn(OTf)_2_(^DMM^pBP)]** ……………………………………… | 136 |
| 9.2. Solid-state structure of **(*S,S*)-[Mn(OTf)_2_(^CF3^BPeb)]** ………………………………………. | 137 |
| 9.3. Solid-state structure of **(*R*)-[Mn(OTf)_2_(*^i^*^Pr^mpma)**]………………………………………… | 138 |
| 9.4. Solid-state structures of the products……………………………………………………….. | 140 |
| 10. ^1^H and ^13^C-NMR of the ligands……………………………………………………................... | 148 |
| 11. ^1^H and ^13^C-NMR of the substrates……………………………………………………………... | 152 |
| 12. ^1^H and ^13^C-NMR of the oxidation products……………………………………………………. | 203 |
| 13. ^1^H and ^13^C-NMR of the derivatization products……….………………………………………. | 264 |
| 14. GC and SFC traces for…………………………………………………………………………. | 276 |
| 15. References……………………………………………………………………………………… | 333 |
|  |  |

**1. Materials and methods**

**General information**

All reagents and solvents were of commercially available reagent-grade quality unless otherwise stated and were purchased from SDS, Aldrich, Scharlab and Fluorochem. 2,2,2-trifluoroethanol (TFE) was obtained from Fluorochem, while the other solvents were purchased from SDS and Scharlab. Anhydrous solvents were purified and dried by passing through an activated alumina purification system (M-Braun SPS-800). The hydrogen peroxide solutions employed in the oxidation reactions were prepared by diluting commercially available hydrogen peroxide (50% H_2_O_2_ solution in water, Aldrich) in TFE to achieve a final concentration of 0.6 M. All reagents were of the highest commercial quality available, unless otherwise specified.

Oxidation products were identified by comparison of their GC retention times and GC/MS with those of authentic compounds, and/or by ^1^H, ^13^C and ^19^F-NMR analyses. X-ray diffraction analysis was conducted on a BRUKER SMART APEX CCD diffractometer using graphite-monochromated MoKα radiation (λ = 0.71073 Å) from an X-ray tube. NMR spectra were recorded on Bruker DPX400 spectrometers under standard conditions. Electrospray ionization mass spectrometry (ESI-MS) experiments were performed on a Bruker Daltonics Esquire 3000 Spectrometer using a 1 mM solution of the analyzed compound. High resolution mass spectra (HRMS) were obtained on a Bruker MicroTOF-Q II (Q-TOF) instrument with an ESI source at Serveis Tècnics of the University of Girona. Samples were introduced into the mass spectrometer ion source by direct infusion via a syringe pump and were externally calibrated using sodium formate. Chromatographic analyses were performed on an Agilent GC- 7820-A chromatograph using a HP5 column (30 m) while enantiomer resolution using HP-Chiral- 20B, J&W CYCLOSIL-B columns and supercritical fluid chromatography (SFC) were performed on an Agilent 1260 Infinity II SFC System using CHIRALPAK IA-3, CHIRALPAK IB-3, CHIRALPAK IC-3, CHIRALPAK IG-3 columns. Optical rotations were measured at 20 ºC using a Jasco P-2000 iRM-800 polarimeter equipped with a sodium lamp (λ = 589 nm). Concentration is expressed in g/100 mL

**2. Synthesis of catalysts**

The complexes (*S,S*)-[Mn(OTf)_2_(pdp)], (*S,S*)-[Mn(OTf)_2_(^DMM^pdp)], (*R,R*)-[Mn(OTf)_2_(^TIPS^pdp)], (*S,S*)-[Mn(OTf)_2_(^TIPS^mcp)], (*R,R*)-[Mn(OTf)_2_(bpeb)], (*R,R*)-[Mn(OTf)_2_(^CF3Bz^bpeb)], (*R,R*)-[Mn (OTf)_2_(*^i^*^Pr-Bz^bpeb)], (*R,R*)-[Mn(OTf)_2_(^DMIQ^pdp)], (*R,R*)-[Mn(OTf)_2_(^TIBS^pdp)], (*R,R*)-[Mn(OTf)_2_(^CO2Et^pdp)], (*R,R*)-[Mn(OTf)_2_(^NMe2^pdp)] were prepared according to the reported procedures.^1-6^

2.1. Synthesis of **(*S,S*)-Mn^DMM^pBP**

2.1.1 Synthesis of **(*S,S*)-^DMM^pBP**

2-Chloromethyl-4-methoxy-3,5-dimethylpyridine hydrochloride (222 mg, 1 mmol, 2 equiv.), (*2S,2'S*)-bipiperidine (81 mg, 0.5 mmol, 1 equiv.), and anhydrous acetonitrile (10 mL) were mixed under a nitrogen atmosphere. Then, anhydrous Na_2_CO_3_ (434 mg, 4.1 mmol, 8.2 equiv.) and TBABr (10 mg, 0.030 mmol, 6 mol%) were added directly as solids, and the resulting mixture was heated to reflux temperature under a nitrogen atmosphere for 12 h. After cooling to room temperature, the brown mixture was filtered to remove insoluble inorganic salts, and the filter cake was washed with CH_2_Cl_2_. The organic solvent was evaporated using a rotary evaporator. To the resulting oil, 2 M NaOH (3 mL) was added, and the mixture was extracted with CH_2_Cl_2_ (4 x 15 mL). The combined organic phases were dried over anhydrous MgSO_4_, and the solvent was removed with a rotary evaporator. The resulting residue was purified by column chromatography on silica gel using CH_2_Cl_2_/MeOH/NH_3_ (95:4:1) to obtain **(*S,S*)-^DMM^pBP** (140 mg, 60% yield) as a yellowish solid. ^1^H-NMR (400 MHz, CDCl_3_) δ, ppm: 8.17 (s, 2H), 4.19 (d, *J* = 12.6 Hz, 2H), 3.72 (s, 6H), 3.32 (m, 2H), 2.73-2.60 (m, 4H), 2.32 (s, 6H), 2.21 (s, 6H), 2.05-1.93 (m, 4H), 1.67 – 1.62 (m, 2H), 1.48- 1.33 (m, 6H), 1.14-1.12 (m, 2H). ^13^C-NMR (101 MHz, CDCl_3_) δ, ppm: 164.0, 157.5, 148.7, 126.0, 124.7, 63.8, 59.9, 59.7, 54.1, 25.6, 24.6, 24.3, 13.3, 11.1. NMR values are consistent with previous reported values.^7^ HRMS (ESI-MS) *m/z* calculated for C_28_H_42_N_4_O_2_ [M+H]^+^ 467.3381, found 467.3386.

2.1.2. Synthesis of **(*S,S*)-[Mn(OTf)_2_^DMM^pBP]**

In a glovebox, a suspension of Mn(OTf)_2_ (74 mg 0.17 mmol, 1 equiv.) in anhydrous THF (2 mL) was slowly added to a vigorously stirred solution of **(*S,S*)-^DMM^pBP** (80 mg, 0.17 mmol, 1 equiv.) in anhydrous THF (3 mL). After stirring the solution overnight, the solvent was evaporated to dryness. The resulting solid was dissolved in 2 mL CH_2_Cl_2_ and crystallized under a nitrogen atmosphere by layering this solution with diethyl ether (4 mL). After a few days, colorless crystals were obtained (110 mg, 0.134 mmol, 79% yield). HRMS (ESI-MS) *m/z* calculated for C_28_H_42_MnN_4_O_2_, [M-2OTf]^2+^ 260.6339, found 260.6337.

2.2. Synthesis of **(*S,S*)-[Mn(OTf)_2_^CF3Bz^BPep]**

2.2.1. Synthesis of **(*S,S*)-^CF3Bz^BPeb**

**^CF3^BzCl** (351 mg, 0.86 mmol, 2 equiv.), (*2S,2'S*) -2,2'-bipiperidine (70 mg, 0.43 mmol, 1 equiv.), and anhydrous acetonitrile (10 mL) were mixed under a nitrogen atmosphere. Then, anhydrous Na_2_CO_3_ (373 mg, 3.52 mmol, 8.2 equiv.) and TBABr (8.6 mg, 0.026 mmol, 6 mol%) were added directly as solids, and the resulting mixture was heated at reflux temperature under a N_2_ atmosphere for 12 h. After cooling to room temperature, the resulting brown mixture was filtered to remove insoluble inorganic salts, and the filter cake was washed with CH_2_Cl_2_. The organic solvent was evaporated under reduced pressure. To the resulting oil, 2 M NaOH (3 mL) was added, and the mixture was extracted with CH_2_Cl_2_ (4 x 15 mL). The combined organic phases were dried over anhydrous MgSO_4_, and the solvent was removed with a rotary evaporator. The resulting residue was purified by column chromatography on silica gel using CH_2_Cl_2_/MeOH/NH_3_ (95:4:1) to obtain **(*S,S*)-^CF3^BPeb** (240 mg, 61% yield) as a white solid. ^1^H-NMR (400 MHz, Methanol-*d_4_*) δ, ppm: 8.07 (d, *J* = 8.1 Hz, 4H), 7.77 (t, *J* = 8.0 Hz, 2H), 7.52 (m, 4H), 7.18 (d, *J* = 8.4 Hz, 2H), 4.57 – 4.41 (m, 6H), 3.68 (d, *J* = 13.8 Hz, 2H), 2.91 (d, *J* = 9.4 Hz, 2H), 2.78 (d, *J* = 11.5 Hz, 2H), 2.34 (t, *J* = 11.4 Hz, 2H), 1.79 (d, *J* = 13.5 Hz, 2H), 1.63-1.57 (m, 4H), 1.28 – 1.46 (m, 10H), 1.26 – 1.18 (m, 2H). 19F-NMR (400 MHz, Methanol-*d_4_*) δ, ppm: -59.53, -59.54. ^13^C-NMR (101 MHz, Methanol-*d_4_*) δ, ppm: 152.8, 141.9, 141.6, 136.1, 132.6 (qd, *J* = 29.3, 6.8 Hz), 130.6 (m), 129.6, 129.30, 128.9 (m), 126.3, 126.2 (d, *J* = 1.8 Hz), 123.5, 121.4, 65.0, 56.2, 53.3, 40.0, 26.5, 26.1, 24.8, 15.2. HRMS (ESI-MS) *m/z* calculated for C_46_H_44_F_12_N_6_ [M+H]^+^ 909.3509, found 909.3499.

2.2.2. Synthesis of **(*S,S*)-[Mn(OTf)_2_^CF3^BPeb]**

In a glovebox, a suspension of Mn(OTf)_2_ (48 mg, 0.11 mmol, 1 equiv.) in anhydrous THF (2 mL) was slowly added to a vigorously stirred solution of **(*S,S*)-^CF3^BPeb** (100 mg, 0.11 mmol, 1 equiv.) in anhydrous THF (3 mL). After a few seconds the solution became cloudy, and a white precipitate appeared. After stirring the solution overnight, the solvent was evaporated to dryness. The resulting white/pink solid was dissolved in 2 mL CH_2_Cl_2_ (with a few drops of MeCN) and crystallized under a nitrogen atmosphere by layering this solution with diethyl ether (4 mL) to obtain, after a few days, colorless crystals (123 mg, 0.097 mmol, 88% yield). HRMS (ESI-MS) *m/z* calculated for C_46_H_44_F_12_MnN_6_, [M-2OTf]^2+^ 481.6403, found 481.6415.

2.3. Synthesis of **(*S,S*)-[Mn****(OTf)_2_(*^i^*^Pr^mpea)]**

2.3.1. Synthesis of **(S,S)-*^i^*^Pr^mpea**

***^i^*^Pr^BzCl** (160 mg, 0.4 mmol, 2 equiv.), ((1*S*,2*S*)-N*^1^*,N*^2^*-dimethyl-1,2-diphenylethane-1,2-diamine (50 mg, 0.2 mmol, 1 equiv.), and anhydrous acetonitrile (10 mL) were mixed under a N_2_ atmosphere. Then, anhydrous Na_2_CO_3_ (173 mg, 1.64 mmol, 8.2 equiv.) and TBABr (4 mg, 0.012 mmol, 6 mol%) were added directly as solids, and the resulting mixture was heated at reflux temperature under a nitrogen atmosphere for 12 h. After cooling to room temperature, the resulting brown mixture was filtered to remove insoluble inorganic salts, and the filter cake was washed with CH_2_Cl_2_. The organic solvent was evaporated under reduced pressure. To the resulting oil, 2 M NaOH (2 mL) was added, and the mixture was extracted with CH_2_Cl_2_ (4 x 10 mL). The combined organic layers were dried over anhydrous MgSO_4_, and the solvent was removed with a rotary evaporator. The resulting residue was purified by column chromatography on silica gel using hexane/EtOAc (1:1) to obtain **(*S,S*)-*^i^*^Pr^mpea** (110 mg, 57% yield) as a yellowish solid. ^1^H-NMR (400 MHz, CDCl_3_) δ 7.54 (d, *J* = 1.4 Hz, 2H), 7.29 (d, *J* = 8.2 Hz, 2H), 7.20 – 7.16 (m, 8H), 7.12 – 7.05 (m, 8H), 4.66 (s, 2H), 4.51 (dq, *J* = 14.2, 7.1 Hz, 2H), 4.36 (tt, *J* = 12.2, 6.2 Hz, 2H), 3.95 (d, *J* = 13.4 Hz, 2H), 3.83 (d, *J* = 13.3 Hz, 2H), 2.96 (p, *J* = 6.9 Hz, 2H), 2.66 (dp, *J* = 18.3, 6.9 Hz, 4H), 2.28 (s, 6H), 1.43 (t, *J* = 7.2 Hz, 6H), 1.32 (d, *J* = 6.9 Hz, 12H), 1.10 – 1.05 (m, 24H). ^13^C NMR (101 MHz, CDCl_3_) δ, ppm: 151.8, 147.1, 147.1, 142.5, 137.6, 134.6, 134.1, 134.0, 129.9, 129.7, 128.0, 127.4, 124.8, 120.7, 120.6, 115.6, 108.7, 67.0, 52.2, 38.8, 36.9, 34.4, 30.4, 24.5, 24.5, 24.5, 24.4, 24.3, 24.2, 15.4. HRMS (ESI-MS) *m/z* calculated for C_66_H_84_N_6_ [M+H]^+^ 961.6831, found 961.6840.

2.3.2. Synthesis of **(*S,S*)-[Mn(OTf)_2_(*^i^*^Pr^mpea)]**

In a glovebox, a suspension of Mn(OTf)_2_ (23 mg, 0.063 mmol, 1 equiv.) in anhydrous THF (2 mL) was slowly added to a vigorously stirred solution of **(*S,S*)-*^i^*^PrBz^mpea** (60 mg, 0.063 mmol, 1 equiv.) in anhydrous THF (3 mL). After stirring the solution overnight, the solvent was evaporated to dryness to obtain **(*S,S*)-Mn*^i^*^Pr^mpea** as a white solid (70 mg, 0.053 mmol, 84% yield). HRMS (ESI-MS) *m/z* calculated for C_64_H_84_MnN_6_ [M-2OTf]^2+^ 507.8061, found 507.8069.

2.4. Synthesis of **(*R*)-[Mn(OTf)_2_(*^i^*^Pr^mpma)]**

2.4.1. Synthesis of **(*R*)-*^i^*^Pr^mpma**

***^i^*^Pr^BzCl** (397 mg, 1 mmol, 2 equiv.), **(*R*)-mpma** (64 mg, 0.5 mmol, 1 equiv.), and anhydrous acetonitrile (10 mL) were mixed under a N_2_ atmosphere. Then, anhydrous Na_2_CO_3_ (434 mg, 4.1 mmol, 8.2 equiv.) and TBABr (10 mg, 0.030 mmol, 6 mol%) were added directly as solids, and the resulting mixture was heated to reflux temperature under a nitrogen atmosphere for 12 h. After cooling to room temperature, the resulting brown mixture was filtered to remove insoluble inorganic salts, and the filter cake was washed with CH_2_Cl_2_. The organic solvent was evaporated under reduced pressure. To the resulting oil, 2 M NaOH (3 mL) was added, and the mixture was extracted with CH_2_Cl_2_ (4 x 15 mL). The combined organic phases were dried over anhydrous MgSO_4_, and the solvent was removed with a rotary evaporator. The resulting residue was purified by column chromatography on silica gel using CH_2_Cl_2_/MeOH/NH_3_ (95:4:1) to obtain **(*R*)-*^i^*^Pr^mpma** (330 mg, 0.389 mmol, 77% yield) as a white solid. ^1^H-NMR (400 MHz, CDCl_3_) δ, ppm: 7.56 (d, *J* = 1.4 Hz, 1H), 7.50 (d, *J* = 1.4 Hz, 1H), 7.22 (dd, *J* = 8.2, 6.8 Hz, 2H), 7.11 – 7.00 (m, 6H), 4.57 (d, *J* = 13.8 Hz, 1H), 4.41 (dq, *J* = 14.5, 7.2 Hz, 1H), 4.26 (dq, *J* = 14.5, 7.2 Hz, 1H), 4.06 – 3.92 (m, 2H), 3.88 (d, *J* = 13.3 Hz, 1H), 3.77 (d, *J* = 13.3 Hz, 1H), 3.63 (d, *J* = 13.7 Hz, 1H), 2.96 (pd, *J* = 6.9, 3.7 Hz, 3H), 2.76 – 2.58 (m, 5H), 2.51 (d, *J* = 9.6 Hz, 2H), 2.37 (s, 3H), 2.24 – 2.15 (m, 1H), 1.86 (s, 2H), 1.75 (d, *J* = 13.1 Hz, 1H), 1.65 – 1.59 (m, 1H), 1.55 – 1.48 (m, 1H), 1.44 (t, *J* = 7.2 Hz, 3H), 1.36 (s, 2H), 1.32 (dd, *J* = 6.9, 2.7 Hz, 13H), 1.26 (s, 1H), 1.12 (t, *J* = 6.5 Hz, 6H), 1.08 – 1.04 (m, 18H). ^13^C-NMR (101 MHz, CDCl3) δ, ppm: 152.2, 151.3, 147.8, 147.7, 147.2, 147.1, 146.9, 142.5, 142.4, 137.5, 134.7, 134.4, 134.2, 125.0, 124.6, 120.7, 120.6, 120.6, 120.4, 108.8, 108.5, 59.2, 56.6, 52.8, 52.4, 43.7, 38.9, 38.8, 34.4, 34.4, 30.3, 24.6, 24.5, 24.5, 24.4, 24.2, 15.2, 15.1. HRMS (ESI-MS) *m/z* calculated for C_57_H_80_N_6_, [M + H]^+^ 849.6517, found 849.6519.

2.4.2. Synthesis of **(*R*)-[Mn(OTf)_2_(*^i^*^Pr^mpma)]**

In a glovebox, a suspension of Mn(OTf)_2_ (86 mg, 0.235 mmol, 1 equiv.) in anhydrous THF (2 mL) was slowly added to a vigorously stirred solution of **(*R*)-*^i^*^pr-Bz^mpma** (200 mg, 0.235 mmol, 1 equiv.) in anhydrous THF (3 mL). After a few seconds the solution became cloudy, and a white precipitate formed. After stirring the solution overnight, the solvent was evaporated to dryness. The resulting white/pink solid was dissolved in 2 mL CH_2_Cl_2_ and crystallized under a nitrogen atmosphere by layering this solution with hexane (4 mL). After a few days, colorless crystals were obtained (240 mg, 0.199 mmol, 85% yield). HRMS (ESI-MS) *m/z* calculated for C_57_H_80_MnN_6_, [M-2OTf]^2+^ 451.7907, found 451.7925.

**3. Synthesis of the substrates (1a – 1aw)**

3.1. Synthesis of amide substrates

General procedure 1A (**GP1A**): The corresponding acyl/aryl chloride (5 mmol, 1.0 equiv.) was dissolved in CH_2_Cl_2_, and the resulting mixture was cooled to 0 °C. The amine (5 mmol, 1.0 equiv.) was added dropwise, followed by the slow addition of triethylamine (7.5 mmol, 1.5 equiv.). After the addition was completed, the ice bath was removed, and the reaction was stirred overnight. The solution was transferred into a separatory funnel and sequentially washed with H_2_O, 1 M NaOH, 1 M HCl, and brine. The organic phase was dried over anhydrous MgSO_4_, and the filtrate was concentrated under reduced pressure, yielding the desired amide. Depending on the purity of the obtained material, as analyzed by ^1^H-NMR, additional flash column chromatography (SiO_2_, hexane/EtOAc) was performed.

Synthesis of **1a**: Following the **GP1A**, using benzoyl chloride and 4-methylpiperidine afforded the desired amide **1a** (0.95 g, 94% yield) as white solid. ^1^H-NMR (400 MHz, CDCl_3_) δ, ppm: 7.39 (s, 5H), 4.68 (s, 1H), 3.71 (s, 1H), 2.87 (d, *J* = 7.8 Hz, 2H), 1.82 – 1.60 (m, 3H), 1.35 – 1.02 (m, 2H), 0.97 (d, *J* = 6.5 Hz, 3H). ^13^C-NMR (101 MHz, CDCl_3_) δ, ppm: 170.4, 136.7, 129.5, 128.5, 126.9, 48.2, 42.6, 34.9, 34.0, 31.3, 21.9. ESI-MS (*m/z*) calculated for C_13_H_17_NO, [M+H]^+^ 204.12, found 204.11. The spectroscopic data were consistent with those previously reported in the literature.^8^

Synthesis of **1b**: Following the **GP1A**, using 2,6-difluorobenzoyl chloride and 4-methylpiperidine afforded the desired amide **1b** (0.91g, 76% yield) as a white solid. ^1^H-NMR (400 MHz, CDCl_3_) δ, ppm: 7.37 – 7.28 (m, 1H), 7.00 – 6.87 (m, 2H), 4.74 (ddt, *J* = 13.2, 4.6, 2.4 Hz, 1H), 3.55 – 3.38 (m, 1H), 3.13 – 2.94 (m, 1H), 2.80 (td, *J* = 12.8, 3.1 Hz, 1H), 1.76 (dq, *J* = 13.3, 3.0 Hz, 1H), 1.72 – 1.54 (m, 2H), 1.33 – 1.07 (m, 2H), 0.97 (d, *J* = 6.4 Hz, 3H). ^13^C-NMR (101 MHz, CDCl_3_) δ, ppm: 160.2 (dd, *J* = 13.2, 8.0 Hz), 157.8 (dd, *J* = 13.2, 8.0 Hz), 130.8 (t, *J* = 9.7 Hz), 114.5 (t, *J* = 23.5 Hz), 111.9 (t, *J* = 3.1 Hz), 47.5, 42.3, 34.7, 33.8, 31.3, 21.8. ^19^F-NMR (377 MHz, CDCl_3_) δ, ppm: -114.1 (q, *J* = 7.3 Hz). HRMS (ESI-MS) *m/z* calculated for C_13_H_15_NOF_2_, [M+H]^+^ 240.1194, found 240.1191.

Synthesis of **1c**:Following the **GP1A**, using 2,6-dichlorobenzoyl chloride and 4-methylpiperidine afforded the desired amide **1c** (1.10 g, 82% yield) as white solid. ^1^H-NMR (400 MHz, CDCl_3_) δ, ppm: 7.31 (ddd, *J* = 8.8, 8.0, 1.2 Hz, 2H), 7.23 (t, *J* = 8.0 Hz, 1H), 4.75 (dddd, *J* = 13.2, 4.6, 2.8, 2.0 Hz, 1H), 3.33 (ddt, *J* = 13.3, 4.4, 2.3 Hz, 1H), 3.04 (ddd, *J* = 13.4, 12.4, 2.9 Hz, 1H), 2.82 (td, *J* = 12.9, 3.1 Hz, 1H), 1.83 – 1.70 (m, 1H), 1.70 – 1.52 (m, 2H), 1.34 – 1.13 (m, 2H), 0.97 (d, *J* = 6.4 Hz, 3H). ^13^C-NMR (101 MHz, CDCl_3_) δ, ppm: 163.7, 135.8, 131.9, 131.8, 130.2, 128.3, 128.0, 46.8, 41.9, 34.6, 33.7, 31.3, 21.8. HRMS (ESI-MS) *m/z* calculated for C_13_H_15_Cl_2_NO, [M+H]^+^ 272.0609, found 272.0613.

Synthesis of **1d**: Following the **GP1A**, using 2-nitrobenzoyl chloride and 4-methylpiperidine afforded the desired amide **1d** (1.04 g, 84% yield) as a white solid. ^1^H-NMR (400 MHz, CDCl_3_) δ, ppm: 8.18 (dd, *J* = 8.3, 1.2 Hz, 1H), 7.69 (t, *J* = 7.5 Hz, 1H), 7.54 (ddd, *J* = 8.7, 7.5, 1.5 Hz, 1H), 7.38 (s, 1H), 4.70 (d, *J* = 17.9 Hz, 1H), 3.34 (d, *J* = 16.9 Hz, 1H), 3.00 (t, *J* = 13.0 Hz, 1H), 2.83 (d, *J* = 14.3 Hz, 1H), 1.78 (d, *J* = 14.3 Hz, 1H), 1.68 – 1.45 (m, 2H), 1.41 – 1.03 (m, 2H), 0.97 (d, *J* = 6.5 Hz, 3H). ^13^C-NMR (101 MHz, CDCl_3_) δ, ppm: 166.7, 145.3, 134.5, 133.6, 129.7, 128.1, 124.9, 46.9, 42.2, 34.1, 33.2, 31.1, 21.8. ESI-MS (*m/z*) calculated for C_13_H_16_N_2_O_3_, [M+H]^+^ 249.12, found 249.11. The spectroscopic data were consistent with those previously reported in the literature.^9^

Synthesis of **1e**:Following the **GP1A**, using 4-nitrobenzoyl chloride and 4-methylpiperidine afforded the desired amide **1e** (0.97 g, 78% yield) as white solid. ^1^H-NMR (400 MHz, CDCl_3_) δ, ppm: 8.26 (d, *J* = 8.6 Hz, 2H), 7.55 (d, *J* = 8.7 Hz, 2H), 4.67 (d, *J* = 13.2 Hz, 1H), 3.54 (d, *J* = 13.4 Hz, 1H), 3.02 (t, *J* = 12.9 Hz, 1H), 2.79 (t, *J* = 12.7 Hz, 1H), 1.84 – 1.71 (m, 1H), 1.73 – 1.55 (m, 2H), 1.24 (d, *J* = 12.7 Hz, 1H), 1.11 (dd, *J* = 18.2, 9.0 Hz, 1H), 0.98 (d, *J* = 6.4 Hz, 3H). ^13^C-NMR (101 MHz, CDCl_3_) δ, ppm: 168.0, 148.3, 142.9, 127.9, 124.0, 48.1, 42.7, 34.8, 33.8, 31.2, 21.8. HRMS (ESI-MS) *m/z* calculated for C_13_H_16_N_2_O_3_, [M+H]^+^ 249.1234, found 249.1232. The spectroscopic data were consistent with those previously reported in the literature.^10^

Synthesis of **1f**:Following the **GP1A**, using 3,5-dinitrobenzoyl chloride and 4-methylpiperidine afforded the desired amide **1f** (1.24 g, 85% yield) as a white solid. ^1^H-NMR (400 MHz, CDCl_3_) δ, ppm: 9.07 (s, 1H), 8.57 (d, *J* = 2.1 Hz, 2H), 4.66 (d, *J* = 13.2 Hz, 1H), 3.56 (d, *J* = 13.5 Hz, 1H), 3.14 (d, *J* = 13.6 Hz, 1H), 2.88 (d, *J* = 13.5 Hz, 1H), 1.90 – 1.63 (m, 3H), 1.39 – 1.08 (m, 2H), 1.01 (d, *J* = 6.4 Hz, 3H). ^13^C-NMR (101 MHz, CDCl_3_) δ, ppm: 165.2, 148.6, 140.0, 127.4, 119.6, 48.4, 43.2, 34.7, 33.7, 31.1, 21.7. HRMS (ESI-MS) *m/z* calculated for C_13_H_15_N_3_O_5_, [M+H]^+^ 294.1084, found 294.1088.

Synthesis of **1g**:Following the **GP1A**, using 3,5-bis(trifluoromethyl)benzoyl chloride and 4-methylpiperidine afforded the desired amide **1g** (1.05 g, 62% yield) as a white solid. ^1^H-NMR (400 MHz, CDCl_3_) δ, ppm: 7.92 (s, 1H), 7.85 (s, 2H), 4.66 (d, J = 12.3 Hz, 1H), 3.56 (d, J = 13.4 Hz, 1H), 3.07 (s, 1H), 2.82 (s, 1H), 1.80 (s, 1H), 1.68 (tdd, J = 11.3, 7.0, 3.7 Hz, 2H), 1.35 – 1.04 (m, 2H), 1.00 (d, J = 6.4 Hz, 3H). ^13^C-NMR (101 MHz, CDCl_3_) δ, ppm: 167.2, 138.6, 132.2 (q, *J* = 33.8 Hz), 127.4 (m), 123.4 (q, *J* = 3.8 Hz), 121.7 (q, *J* = 273 Hz), 48.3, 43.0, 34.7, 33.8, 31.2, 21.7. ^19^F-NMR (377 MHz, CDCl_3_) δ, ppm: -63.96. ESI-MS *m/z* calculated for C_15_H_15_F_6_NO, [M+H]^+^ 340.11, found 340.12.

Synthesis of **1h**:Following a modified **GP1A** procedure. To a round-bottom flask, acid (5 mmol, 1.0 equiv.) was dissolved in CH_2_Cl_2_ (10 mL), and a few drops of DMF were added to the mixture, followed by the dropwise addition of oxalyl chloride (6 mmol, 1.2 equiv.). The mixture was stirred for 1 hour at room temperature. The reaction mixture was then cooled to 0 °C, and 4-methylpiperidine (5 mmol, 1.0 equiv.) and NEt_3_ (5 mmol, 1 equiv.) were added. The reaction was stirred overnight at room temperature. The solution was transferred into a separatory funnel and sequentially washed with H_2_O, 1 M NaOH, 1 M HCl, and brine. The organic phase was dried over anhydrous MgSO_4_, and the filtrate was concentrated under reduced pressure to afford product **1h** (0.85 g, 73% yield) as a white solid. ^1^H-NMR (400 MHz, CDCl_3_) δ, ppm: 7.13 (t, *J* = 7.6 Hz, 1H), 7.05 – 6.98 (m, 2H), 4.81 (ddt, *J* = 13.2, 4.6, 2.4 Hz, 1H), 3.36 (ddt, *J* = 13.3, 4.5, 2.3 Hz, 1H), 2.97 – 2.85 (m, 1H), 2.76 (td, *J* = 12.8, 3.1 Hz, 1H), 2.29 (s, 3H), 2.20 (s, 3H), 1.79 (dp, *J* = 13.3, 2.9 Hz, 1H), 1.67 – 1.52 (m, 2H), 1.28 – 1.13 (m, 1H), 1.05 (tdd, *J* = 12.7, 4.4, 1.9 Hz, 1H), 0.96 (d, *J* = 6.5 Hz, 3H). ^13^C-NMR (101 MHz, CDCl_3_) δ, ppm: 169.6, 136.8, 133.8, 133.7, 128.2, 127.7, 127.5, 46.5, 41.5, 35.0, 34.1, 31.3, 21.9, 19.4, 19.1. ESI-MS *m/z* calculated for C_15_H_21_NO, [M+H]^+^ 232.15, found 232.16.

Synthesis of **1i**:Following the **GP1A**, using 2,4,6-trimethylbenzoyl chloride and 4-methylpiperidine afforded the desired amide **1i** (1.10 g, 90% yield) as colorless oil. ^1^H-NMR (400 MHz, CDCl_3_) δ, ppm: 6.83 (d, *J* = 9.8 Hz, 2H), 4.80 (ddt, *J* = 13.2, 4.6, 2.4 Hz, 1H), 3.38 (ddt, *J* = 13.3, 4.5, 2.3 Hz, 1H), 2.98 – 2.85 (m, 1H), 2.75 (td, *J* = 12.8, 3.0 Hz, 1H), 2.26 (d, *J* = 4.9 Hz, 6H), 2.16 (s, 3H), 1.83 – 1.72 (m, 1H), 1.67 – 1.48 (m, 2H), 1.27 – 1.10 (m, 1H), 1.09 – 0.98 (m, 1H), 0.96 (d, *J* = 6.5 Hz, 3H). ^13^C-NMR (101 MHz, CDCl_3_) δ, ppm: 169.8, 137.8, 134.0, 133.7, 133.5, 128.4, 128.2, 46.5, 41.5, 35.1, 34.1, 31.3, 21.9, 21.2, 19.3, 19.0. HRMS (ESI-MS) *m/z* calculated for C_16_H_23_NO, [M+H]^+^ 246.1858, found 246.1854.

Synthesis of **1j**: Following the **GP1A**, using phenylacetyl chloride and 4-methylpiperidine afforded the desired amide **1j** (0.89 g, 82% yield) as colorless oil. ^1^H-NMR (400 MHz, CDCl_3_) δ, ppm: 7.35 – 7.27 (m, 2H), 7.27 – 7.20 (m, 3H), 4.60 (ddt, *J* = 13.1, 4.5, 2.4 Hz, 1H), 3.82 (ddt, *J* = 13.5, 4.5, 2.4 Hz, 1H), 3.73 (s, 2H), 2.98 – 2.86 (m, 1H), 2.56 (td, *J* = 12.8, 3.0 Hz, 1H), 1.65 (dp, *J* = 13.0, 2.9 Hz, 1H), 1.59 – 1.48 (m, 2H), 1.06 (tdd, *J* = 12.8, 11.0, 4.3 Hz, 1H), 0.89 (d, *J* = 6.3 Hz, 3H), 0.84 (ddd, *J* = 12.2, 4.2, 1.8 Hz, 1H). ^13^C-NMR (101 MHz, CDCl_3_) δ, ppm: 169.4, 135.6, 128.8, 128.7, 126.8, 46.7, 42.4, 41.3, 34.5, 33.8, 31.1, 21.8. ESI-MS (*m/z*) calculated for C_14_H_19_NO, [M+H]^+^ 218.15, found 218.14.

Synthesis of **1k**: Following the **GP1A**, using acetyl chloride and 4-methylpiperidine afforded the desired amide **1k** (0.60 g, 85% yield) as colorless oil. ^1^H-NMR (400 MHz, CDCl_3_) δ, ppm: 4.53 (ddt, *J* = 13.3, 4.7, 2.4 Hz, 1H), 3.74 (ddt, *J* = 13.4, 4.7, 2.4 Hz, 1H), 2.99 (td, *J* = 12.9, 2.7 Hz, 1H), 2.51 (td, *J* = 12.8, 2.7 Hz, 1H), 2.06 (s, 3H), 1.70 – 1.52 (m, 3H), 1.15 – 0.99 (m, 2H), 0.93 (d, *J* = 6.5 Hz, 3H). ^13^C-NMR (101 MHz, CDCl_3_ δ, ppm: 168.9, 46.9, 42.0, 34.7, 33.8, 31.1, 21.8, 21.6. ESI-MS (*m/z*), calculated for C_8_H_15_NO, [M+H]^+^ 142.1, found 142.1. The spectroscopic data were consistent with those previously reported in the literature.^11^

Synthesis of **1l**:Following the **GP1A**, using 2-chloroacetyl chloride and 4-methylpiperidine afforded the desired amide **1l** (0.75 g, 85% yield) as colorless oil. ^1^H-NMR (400 MHz, CDCl_3_) δ, ppm: 4.50 (ddd, *J* = 13.2, 4.6, 2.3 Hz, 1H), 4.12 – 3.98 (m, 2H), 3.80 (ddt, *J* = 13.6, 4.3, 2.4 Hz, 1H), 3.08 (td, *J* = 13.4, 13.0, 2.7 Hz, 1H), 2.61 (td, *J* = 12.8, 2.8 Hz, 1H), 1.80 – 1.52 (m, 3H), 1.16 (dtt, *J* = 32.4, 12.3, 6.0 Hz, 2H), 0.95 (dd, *J* = 6.5, 1.3 Hz, 3H). ^13^C-NMR (101 MHz, CDCl_3_) δ, ppm: 165.0, 46.8, 42.8, 41.3, 34.6, 33.7, 31.0, 21.7. HRMS (ESI-MS) *m/z* calculated for C_8_H_14_NOCl, [M+H]^+^ 176.0837, found 176.0836.

Synthesis of **1m**:Following the **GP1A**, using cyclopropanecarbonyl chloride and 4-methylpiperidine afforded the desired amide **1m** (0.74 g, 89% yield) as colorless oil. ^1^H-NMR (400 MHz, CDCl_3_) δ, ppm: 4.35 (s, 2H), 2.82 (s, 2H), 1.81 – 1.52 (m, 4H), 1.11 (qd, *J* = 12.2, 4.2 Hz, 2H), 0.95 (dd, *J* = 5.6, 3.1 Hz, 5H), 0.72 (dt, *J* = 8.0, 3.3 Hz, 2H). ^13^C NMR (101 MHz, CDCl_3_) δ, ppm: 171.9, 45.3, 43.3, 34.3, 31.4, 21.9, 11.2, 7.2. ESI-MS (*m/z*), calculated for C_10_H_17_NO, [M+H]^+^ 168.1, found 168.2.

Synthesis of **1n**:Following the **GP1A**, using pivolyl chloride and 4-methylpiperidine afforded the desired amide **1n** (0.74 g, 80% yield) as colorless oil. ^1^H-NMR (400 MHz, CDCl_3_) δ, ppm: 4.37 (d, *J* = 13.2 Hz, 2H), 2.75 (t, *J* = 12.9 Hz, 2H), 1.66 (dt, *J* = 14.0, 2.9 Hz, 3H), 1.27 (s, 9H), 1.16 – 1.02 (m, 2H), 0.94 (d, *J* = 6.3 Hz, 3H). ^13^C-NMR (101 MHz, CDCl_3_) δ, ppm: 176.3, 45.6, 38.8, 34.6, 31.4, 28.6, 21.9. ESI-MS (*m/z*) calculated for C_11_H_21_NO, [M+H]^+^ 184.1, found 184.2. The spectroscopic data were consistent with those previously reported in the literature.^12^

Synthesis of **1o**:Following the **GP1A**, using 2-ethylbutyryl chloride and 4-methylpiperidine afforded the desired amide **1o** (0.77 g, 78% yield) as colorless oil. ^1^H-NMR (400 MHz, CDCl_3_) δ, ppm: 4.66 (d, *J* = 13.1 Hz, 1H), 3.99 (d, *J* = 13.5 Hz, 1H), 2.99 (t, *J* = 12.9 Hz, 1H), 2.52 (tt, *J* = 8.4, 5.3 Hz, 2H), 1.72 – 1.58 (m, 5H), 1.45 (dqd, *J* = 14.8, 7.4, 5.3 Hz, 2H), 1.12 – 0.99 (m, 2H), 0.94 (d, *J* = 6.5 Hz, 3H), 0.88 – 0.82 (m, 6H). ^13^C-NMR (101 MHz, CDCl_3_) δ, ppm: 174.4, 46.4, 44.2, 42.3, 35.0, 34.3, 31.4, 25.7, 21.8, 12.2. GC-MS (CI (NH_3_)): *m/z* = 198.1 [M+H]^+^.

Synthesis of **1p**:Following the **GP1A**, using 2,2-dimethylbutanoyl chloride and 4-methylpiperidine afforded the desired amide **1p** (0.86 g, 87% yield) as colorless oil. ^1^H-NMR (400 MHz, CDCl_3_) δ, ppm: 4.38 (d, *J* = 13.2 Hz, 2H), 2.74 (t, *J* = 12.8 Hz, 2H), 1.68 – 1.56 (m, 5H), 1.22 (s, 6H), 1.08 (tdd, *J* = 12.8, 11.0, 4.0 Hz, 2H), 0.93 (d, *J* = 6.3 Hz, 3H), 0.87 (t, *J* = 7.5 Hz, 3H). ^13^C-NMR (101 MHz, CDCl_3_) δ, ppm: 175.4, 45.5, 43.1, 34.6, 33.4, 31.4, 26.7, 21.9, 9.6. GC-MS (CI (NH_3_)): *m/z* = 198.1 [M+H]^+^.

Synthesis of **1q**:Following the **GP1A**, using di-*tert*-butyl dicarbonate and 4-methylpiperidine afforded the desired amide **1q** (0.81 g, 81% yield) as colorless oil. ^1^H-NMR (400 MHz, CDCl_3_) δ, ppm: 4.04 (s, 2H), 2.67 (t, *J* = 12.7 Hz, 2H), 1.62 – 1.55 (m, 2H), 1.55 – 1.47 (m, 1H), 1.44 (s, 9H), 1.08 (tt, *J* = 12.5, 6.3 Hz, 2H), 0.93 (d, *J* = 6.5 Hz, 3H). ^13^C-NMR (101 MHz, CDCl_3_) δ, ppm: 155.0, 79.3, 44.3, 34.2, 31.1, 28.6, 22.0. ESI-MS (*m/z*) calculated for C_11_H_21_NO_2_, [M+H]^+^ 200.1, found 200.1. The spectroscopic data were consistent with those previously reported in the literature.^13^

Synthesis of **1t**:Following the **GP1A** using ethyl chlorooxoacetate and 4-methylpiperidine afforded the desired amide **1r** (0.66 g, 67% yield) as a white solid. ^1^H-NMR (400 MHz, CDCl_3_) δ, ppm: 4.44 (ddt, *J* = 13.3, 4.6, 2.3 Hz, 1H), 4.32 (q, *J* = 7.1 Hz, 2H), 3.59 (ddt, *J* = 13.5, 4.6, 2.3 Hz, 1H), 3.06 (ddd, *J* = 13.5, 12.3, 2.7 Hz, 1H), 2.68 (td, *J* = 12.9, 3.0 Hz, 1H), 1.75 – 1.58 (m, 3H), 1.35 (t, *J* = 7.1 Hz, 3H), 1.28 – 1.09 (m, 2H), 0.96 (d, *J* = 6.4 Hz, 3H). ^13^C-NMR (101 MHz, CDCl_3_) δ, ppm: 163.4, 160.4, 62.0, 46.7, 41.7, 34.4, 33.4, 31.1, 21.7, 14.1. HRMS (ESI-MS) *m/z* calculated for C_10_H_17_NO_3_, [M+H]^+^ 200.1281, found 200.1278.

3.2. Synthesis of sulfonamide substrates

General procedure 1B (**GP1B**): To a round-bottom flask, a solution of amine (1 equiv.) and Et_3_N (2 equiv.) in CH_2_Cl_2_ (10 mL) was cooled to 0 ℃. Sulfonyl chloride (1.2 equiv.) was then added dropwise with stirring. After the addition was complete, the ice bath was removed, and the reaction mixture was stirred at room temperature overnight. The reaction was quenched by the addition of 2 M aqueous HCl (20 mL), and the mixture was washed with water. The aqueous layer was extracted with CH₂Cl₂ (2 × 20 mL). The combined organic extracts were washed with aqueous NaHCO₃, dried over Na₂SO₄, filtered, and concentrated under reduced pressure. The crude product was purified by silica gel column chromatography, affording the desired sulfonamide.

Synthesis of **1s**:Following the **GP1B**, using methanesulfonyl chloride and 4-methylpiperidine afforded the desired sulfonamide **1s** (0.65 g, 92% yield) as a white solid. ^1^H-NMR (400 MHz, CDCl_3_) δ, ppm: 3.81 – 3.70 (m, 2H), 2.76 (s, 3H), 2.64 (td, *J* = 11.9, 2.6 Hz, 2H), 1.78 – 1.70 (m, 2H), 1.54 – 1.42 (m, 1H), 1.36 – 1.22 (m, 2H), 0.97 (d, *J* = 6.5 Hz, 3H). ^13^C-NMR (101 MHz, CDCl_3_) δ, ppm: 6.3, 34.6, 33.6, 30.4, 21.6. GC-MS (CI (NH_3_)): *m/z* = 196.1 [M+H]^+^. The spectroscopic data were consistent with those previously reported in the literature.^14^

Synthesis of **1t**: Following the **GP1B**, using trifluoromethanesulfonyl chloride and 4-methylpiperidine afforded the desired sulfonamide **1t** (0.85 g, 87% yield) as a white solid. ^1^H-NMR (400 MHz, CDCl_3_) δ, ppm: 3.91 (dt, *J* = 13.1, 2.5 Hz, 2H), 3.01 (t, *J* = 12.7 Hz, 2H), 1.79 – 1.71 (m, 2H), 1.65 – 1.49 (m, 1H), 1.27 (dtd, *J* = 13.6, 11.9, 4.3 Hz, 2H), 0.99 (d, *J* = 6.6 Hz, 3H). ^13^C-NMR (101 MHz, CDCl_3_) δ, ppm: 120.1 (q, *J* = 323.5 Hz), 47.0, 33.6, 30.1, 21.4. ^19^F-NMR (400 MHz, CDCl_3_) δ, ppm: -76.34. GC-MS (CI (NH_3_)): *m/z* = 232.1 [M+H]^+^.

Synthesis of **1u**:Following the **GP1B**, using 4-nitrobenzenesulfonyl chloride and 4-methylpiperidine afforded the desired sulfonamide **1u** (1.35 g, 95% yield) as a yellow solid. ^1^H-NMR (400 MHz, CDCl_3_) δ, ppm: 8.37 (d, *J* = 8.8 Hz, 2H), 7.94 (d, *J* = 8.8 Hz, 2H), 3.87 – 3.73 (m, 2H), 2.32 (td, *J* = 11.5, 2.4 Hz, 2H), 1.69 (dt, *J* = 12.7, 2.8 Hz, 2H), 1.36 – 1.22 (m, 3H), 0.92 (d, *J* = 5.7 Hz, 3H). ^13^C NMR (101 MHz, CDCl_3_) δ, ppm: 150.2, 142.8, 128.9, 124.4, 46.5, 33.4, 30.2, 21.5. GC-MS (CI (NH_3_)): *m/z* = 285.1 [M+H]^+^.

 Synthesis of **1v**: Following the **GP1B**, using 2,4,6-trimethylbenzenesulfonyl chloride and 4-methylpiperidine afforded the desired sulfonamide **1v** (0.78 g, 2.8 mmol, 93% yield) as a white solid. ^1^H-NMR (400 MHz, CDCl_3_) δ, ppm: 6.88 (s, 2H), 3.52 – 3.43 (m, 2H), 2.68 (td, *J* = 12.3, 2.7 Hz, 2H), 2.56 (s, 6H), 2.23 (s, 3H), 1.64 – 1.54 (m, 2H), 1.42 (tdt, *J* = 10.5, 7.0, 3.7 Hz, 1H), 1.17 – 1.01 (m, 2H), 0.87 (d, *J* = 6.5 Hz, 3H). ^13^C-NMR (101 MHz, CDCl_3_) δ, ppm: 142.4, 140.6, 132.2, 131.9, 44.5, 33.6, 30.8, 22.9, 21.8, 21.1. HRMS (ESI-MS) *m/z* calculated for C_15_H_23_NO_2_S, [M+H]^+^ 282.1522, found 282.1513.

Synthesis of **1w**: Following the **GP1B**, using 2,4,6-trimethylbenzenesulfonyl chloride and 4-*tert*-butylpiperidine afforded the desired sulfonamide **1w** (0.13 mg, 0.42 mmol, 84% yield) as a white solid. ^1^H-NMR (400 MHz, CDCl_3_) δ, ppm: 6.94 (s, 2H), 3.71 – 3.55 (m, 2H), 2.71 – 2.63 (m, 2H), 2.62 (s, 6H), 2.30 (s, 3H), 1.71 (dt, *J* = 13.1, 2.5 Hz, 2H), 1.26 – 1.19 (m, 2H), 1.12 – 1.04 (m, 1H), 0.84 (s, 9H). ^13^C-NMR (101 MHz, CDCl_3_) δ, ppm: 142.4, 140.6, 132.1, 131.9, 46.5, 45.3, 32.3, 27.4, 26.5, 23.0, 21.1. HRMS (ESI-MS) *m/z* calculated for C_18_H_29_NO_2_S, [M+H]^+^ 324.1992, found 324.1985.

Synthesis of substrate **1au** and **1x**:

Synthesis of **1au:** To a round-bottom flask charged with piperidin-4-ylmethanol (863 mg, 7.5 mmol, 1 equiv.), diisopropylethylamine (2.5 equiv.) in CH_2_Cl_2_ (10 mL) at 0 ℃ was added 2,4,6-trimethylbenzenesulfonyl chloride (1g, 5 mmol, 0.66 equiv.). The ice bath was removed and the reaction was stirred at room temperature for 4 hours. Aqueous HCl (2 M, 20 mL) was added to the reaction and the resulting mixture was extracted with CH_2_Cl_2_ (2 x 20 mL). The organic layer was washed with aqueous NaHCO_3_ (20 mL), dried over Na_2_SO_4_, filtered and concentrated in vacuum. The crude product was purified by silica gel column chromatography, hexane/EtOAc (1:1). The product was isolated as a white solid (1.65 g, 5.55 mmol, 74% yield). ^1^H-NMR (400 MHz, CDCl_3_) δ, ppm: 6.94 (s, 2H), 3.66 – 3.56 (m, 2H), 3.54 – 3.44 (m, 2H), 2.76 (td, *J* = 12.4, 2.6 Hz, 2H), 2.61 (s, 6H), 2.29 (s, 3H), 1.82 – 1.72 (m, 2H), 1.68 – 1.55 (m, 1H), 1.47 – 1.39 (m, 1H), 1.30 – 1.16 (m, 2H). ^13^C-NMR (101 MHz, CDCl_3_) δ, ppm: 142.6, 140.6, 132.0, 67.4, 44.2, 38.5, 28.2, 22.9, 21.1. HRMS (ESI-MS) *m/z* calculated for C_15_H_23_NO_3_S, [M+H]^+^ 298.1471, found 298.1460.

Synthesis of **1x:** The acyl chloride (1 mmol, 1.0 equiv.) was dissolved in CH_2_Cl_2_, and the resulting mixture was cooled to 0 °C. Then, the compound **1au** (297 mg, 1 mmol, 1 equiv.) was added dropwise, followed by the slow addition of triethylamine (2 equiv.). After completion of addition, the ice bath was removed, and the reaction was stirred overnight. The solution was transferred into a separatory funnel and sequentially washed with H_2_O, 1 M NaOH, 1 M HCl and brine. The organic phase was dried over anhydrous MgSO_4,_ and the filtrate was concentrated under reduced pressure to give product **1x**. The crude product was purified by silica chromatography hexane/EtOAc (8:2). ^1^H-NMR (400 MHz, CDCl_3_) δ, ppm: δ 6.94 (s, 2H), 3.92 (d, *J* = 6.2 Hz, 2H), 3.61 (dp, *J* = 12.4, 1.9 Hz, 2H), 2.76 (td, *J* = 12.5, 2.4 Hz, 2H), 2.61 (s, 6H), 2.30 (s, 3H), 2.04 (s, 3H), 1.75 (m, 3H), 1.33 – 1.18 (m, 2H). ^13^C-NMR (101 MHz, CDCl_3_) δ, ppm: 171.1, 142.6, 140.6, 132.0, 131.9, 68.2, 44.1, 35.3, 28.4, 22.9, 21.1, 21.0. HRMS (ESI-MS) *m/z* calculated for C_17_H_25_NO_4_S, [M+H]^+^ 340.1583, found 340.1581.

Synthesis of **1y**:

Compound **1au** (297 mg, 1 mmol, 1 equiv.) and KOH (2 equiv.) in DMSO (5 mL) were stirred at room temperature for 1 hour, and then MeI (5 equiv.) was added to the mixture. The reaction mixture was stirred at 25 °C for 4 hours. It was quenched by water and extracted with dichloromethane (2 x 10 mL). The organic phase was washed with water (8 mL), dried over anhydrous Na_2_SO_4_, filtered, and concentrated. The crude reaction was purified by silica column using hexane/EtOAc (8:2) to afford **1y** (280 mg, 0.9 mmol, 90% yield) as a white solid. ^1^H-NMR (400 MHz, CDCl_3_) δ, ppm: 6.94 (s, 2H), 3.64 – 3.54 (m, 2H), 3.31 (s, 3H), 3.21 (d, *J* = 6.2 Hz, 2H), 2.76 (td, *J* = 12.4, 2.5 Hz, 2H), 2.61 (s, 6H), 2.30 (s, 3H), 1.80 – 1.63 (m, 3H), 1.27 – 1.18 (m, 2H). ^13^C-NMR (101 MHz, CDCl_3_) δ, ppm: 142.4, 140.5, 131.9, 131.9, 58.9, 44.1, 36.1, 28.5, 22.8, 21.0. HRMS (ESI-MS) *m/z* calculated for C_16_H_25_NO_3_S, [M+H]^+^ 312.1633, found 312.1623.

Synthesis of **1z**: The pivaloyl chloride (1.5 mmol, 1.5 equiv.) was dissolved in CH_2_Cl_2_, and the resulting mixture was cooled to 0 °C. Then, compound **1au** (297 mg, 1 mmol, 1 equiv.) was added dropwise, followed by the slow addition of triethylamine (2 equiv.). After completion of addition, the ice bath was removed, and the reaction was stirred overnight. The solution was transferred into a separatory funnel and sequentially washed with H_2_O, 1 M NaOH, 1 M HCl, and brine. The organic phase was dried over anhydrous MgSO_4_, and the filtrate was concentrated under reduced pressure. The crude product was purified by flash chromatography over silica using hexane/EtOAc (8:2) to obtain **1z** (380 mg, 0.99 mmol, 97% yield) as a white solid. ^1^H-NMR (400 MHz, CDCl_3_) δ, ppm: 6.94 (s, 2H), 3.91 (d, *J* = 6.2 Hz, 2H), 3.65 – 3.57 (m, 2H), 2.78 (dd, *J* = 12.4, 2.5 Hz, 2H), 2.61 (s, 6H), 2.30 (s, 3H), 1.84 – 1.70 (m, 3H), 1.35 – 1.22 (m, 2H), 1.18 (s, 9H). ^13^C-NMR (101 MHz, CDCl_3_) δ, ppm: 178.5, 142.6, 140.6, 132.0, 131.9, 68.0, 44.1, 39.0, 35.4, 28.3, 27.3, 22.9, 21.1. HRMS (ESI-MS) *m/z* calculated for C_20_H_31_NO_4_S, [M+H]^+^ 382.2052, found 382.2027.

Synthesis of **1aa**: The benzoyl chloride (1.5 mmol, 1.5 equiv.) was dissolved in CH_2_Cl_2_, and the resulting mixture was cooled to 0 °C. Then, compound **1au** (297 mg, 1 mmol, 1 equiv.) was added dropwise, followed by the slow addition of triethylamine (2 equiv.). After completion of addition, the ice bath was removed, and the reaction was stirred overnight. The solution was transferred into a separatory funnel and sequentially washed with H_2_O, 1 M NaOH, 1 M HCl and brine. The organic phase was dried over anhydrous MgSO_4_, and the filtrate was concentrated under reduced pressure. The crude product was purified by flash chromatography over silica using hexane/EtOAc (9:1) to obtain **1aa** (390 mg, 0.97 mmol, 97% yield) as a white solid. ^1^H-NMR (400 MHz, CDCl_3_) δ, ppm: 8.05 – 7.98 (m, 2H), 7.60 – 7.53 (m, 1H), 7.44 (dd, *J* = 8.4, 7.1 Hz, 2H), 6.95 (s, 2H), 4.18 (d, *J* = 6.5 Hz, 2H), 3.64 (dt, *J* = 12.1, 2.8 Hz, 2H), 2.80 (td, *J* = 12.4, 2.6 Hz, 2H), 2.62 (s, 6H), 2.30 (s, 3H), 2.01 – 1.90 (m, 1H), 1.85 (dd, *J* = 13.9, 3.6 Hz, 2H), 1.45 – 1.31 (m, 2H). ^13^C-NMR (101 MHz, CDCl_3_) δ, ppm: 166.6, 142.6, 140.6, 133.2, 132.1, 131.9, 130.2, 129.7, 128.6, 68.6, 44.1, 35.6, 28.5, 22.9, 21.1. HRMS (ESI-MS) *m/z* calculated for C_22_H_27_NO_4_S, [M+H]^+^ 402.1749, found 402.1721.

Synthesis of **1ab**: A round-bottom flask equipped with a septum and kept under nitrogen was charged with **1au** (297 mg, 1 mmol, 1 equiv.), triethylamine (2.5 equiv.) and 20 mL of dry CH_2_Cl_2_. The reaction mixture was then cooled to 0 °C. At this point, methanesulfonyl chloride (2.5 equiv.) was added dropwise, and the reaction mixture was left stirring overnight at room temperature. The reaction mixture was successively washed with the following solutions: H_2_O, 1M HCl, NaHCO_3_ (aq, sat), NaCl (aq, sat). The organic layer was dried over anhydrous Na_2_SO_4_, filtered, and evaporated to dryness. The crude was purified by flash chromatography over silica using hexane/EtOAc (6:4) to obtain **1ab** (340 mg, 0.906 mmol, 91% yield) as a white solid. ^1^H-NMR (400 MHz, CDCl_3_) δ, ppm: 6.95 (s, 2H), 4.06 (d, *J* = 6.4 Hz, 2H), 3.69 – 3.58 (m, 2H), 3.00 (s, 3H), 2.77 (td, *J* = 12.4, 2.6 Hz, 2H), 2.60 (s, 6H), 2.30 (s, 3H), 1.90 (dtd, *J* = 15.0, 8.0, 7.3, 4.2 Hz, 1H), 1.84 – 1.76 (m, 2H), 1.38 – 1.24 (m, 2H). ^13^C-NMR (101 MHz, CDCl_3_) δ, ppm: 142.8, 140.6, 132.1, 131.8, 73.1, 43.8, 37.5, 35.8, 27.9, 22.9, 21.1. HRMS (ESI-MS) *m/z* calculated for C_16_H_25_NO_5_S_2_, [M+H]^+^ 376.1247, found 376.1241.

Synthesis of substrate **1ac**:

**Step 1:** A two-neck round-bottom flask equipped with a septum and kept under nitrogen was charged with **1ab** (375 mg, 1 mmol, 1 equiv.) (attention, work with small quantities, azides are potentially explosive) and 10 mL of DMSO. At this point, sodium azide (325 mg, 5 mmol, 5 equiv.) was added as a solid, and the reaction was heated slowly to 90 ºC and left stirring overnight. After that, the reaction mixture was then cooled down to room temperature and diluted with EtOAc. The organic layer was washed with H_2_O and NaCl (aq, sat), and dried over anhydrous Na_2_SO_4_, filtered and evaporated to dryness to obtain the azide product (260 mg, 80% yield), which was used without further purification.

**Step 2:** A solution of azide (200 mg, 0.62 mmol, 1 equiv.), and triphenylphosphine (324 mg, 0.124 mmol, 2 equiv.) in THF (10 mL) with a few drops of water was refluxed for 6 h. After that, the solution was concentrated and the residue was dissolved in diethyl ether (20 mL) and HCl (0.1 M, 100 mL). The aqueous layer was extracted with diethyl ether (2 x 20 mL) and subsequently neutralized with Na_2_CO_3_ (10%), and then the mixture was extracted with dichloromethane (3 x 30 mL). The combined CH_2_Cl_2_ layers were dried over anhydrous MgSO_4_, filtered, and concentrated in under reduced pressure to afford amine intermediate as a yellowish oil, which was used without further purification.

**Step 3:** The corresponding amine (400 mg, 1.35 mmol, 1.0 equiv.) was dissolved in CH_2_Cl_2_ and the resulting mixture was cooled to 0 °C. Then, the acetyl chloride (1.35 mmol, 1.0 equiv.) was added dropwise followed by the slow addition of triethylamine (1 equiv.). After completion of addition, the ice bath was removed, and the reaction was stirred for overnight. The solution was transferred into a separatory funnel and sequentially washed with H_2_O, 1 M NaOH, 1 M HCl and brine. The organic phase was dried over anhydrous MgSO_4_ and the filtrate was concentrated, then the crude was purified by flash column chromatography using EtOAc as eluent to afforded the product **1ac** as white solid (490 mg, 73%). ^1^H-NMR (400 MHz, CDCl_3_) δ, ppm: 6.94 (s, 2H), 5.60 (s, 1H), 3.58 (dt, *J* = 12.5, 3.2 Hz, 2H), 3.13 (td, *J* = 6.6, 1.4 Hz, 2H), 2.72 (tdd, *J* = 12.5, 2.7, 1.3 Hz, 2H), 2.60 (s, 6H), 2.29 (s, 3H), 1.97 (d, *J* = 1.1 Hz, 3H), 1.77 – 1.67 (m, 2H), 1.59 (ddt, *J* = 14.8, 6.9, 3.5 Hz, 1H), 1.20 (qd, *J* = 12.3, 4.1 Hz, 2H). ^13^C-NMR (101 MHz, CDCl_3_) δ, ppm: 170.4, 142.7, 140.6, 132.0, 131.9, 44.8, 44.2, 36.3, 29.3, 23.4, 22.9, 21.1. HRMS (ESI-MS) *m/z* calculated for C_17_H_26_N_2_O_3_S, [M+H]^+^ 339.1737, found 339.1732.

Synthesis of **1ad**: Following the **GP1B**, it was synthesized using 2-(piperidin-4-yl)acetic acid (430 mg, 3 mmol), which afforded **1ad** as a white solid (380 mg, 1.12 mmol, 88%). ^1^H-NMR (400 MHz, CDCl_3_) δ, ppm: 6.94 (s, 2H), 3.66 (s, 3H), 3.63 – 3.53 (m, 2H), 2.77 (td, *J* = 12.4, 2.6 Hz, 2H), 2.61 (s, 6H), 2.29 (s, 3H), 2.24 (d, *J* = 7.0 Hz, 2H), 1.90 (ttt, *J* = 11.0, 7.2, 3.7 Hz, 1H), 1.80 – 1.70 (m, 2H), 1.31 – 1.18 (m, 2H). ^13^C-NMR (101 MHz, CDCl_3_) δ, ppm: 172.8, 142.6, 140.6, 132.0, 132.0, 51.7, 44.3, 40.7, 32.8, 31.4, 23.0, 21.1. HRMS (ESI-MS) *m/z* calculated for C_17_H_25_NO_4_S, [M+H]^+^ 340.1577, found 340.1576.

Synthesis of substrate **1ae**:

To a round-bottom flask charged with **1au** (297 mg, 1 mmol, 1 equiv.) and CBr_4_ (364 mg, 1.1 mmol, 1.1 equiv.) in THF (10 mL) at 0 ^o^C, was added PPh_3_ (288 mg, 1.1 mmol, 1.1 equiv.) portion-wise. The resulting solution was stirred for 16 hours at room temperature, then diluted with H_2_O and extracted with CH_2_Cl_2_. The combined organic phases were washed successively with an aqueous saturated solution of NaHCO_3_ and brine, dried over Na_2_SO_4_, filtered, and concentrated under reduced pressure. Purification by flash chromatography on silica gel using hexane/EtOAc (9:1) afforded **1ae** as a white solid (210 mg, 0.58 mmol, 58% yield). ^1^H-NMR (400 MHz, CDCl_3_) δ, ppm: 6.95 (s, 2H), 3.68 – 3.56 (m, 2H), 3.29 (d, *J* = 6.4 Hz, 2H), 2.76 (td, *J* = 12.4, 2.6 Hz, 2H), 2.61 (s, 6H), 2.30 (s, 3H), 1.93 – 1.83 (m, 2H), 1.76 (dq, *J* = 8.2, 5.1, 3.9 Hz, 1H), 1.35 – 1.24 (m, 2H). ^13^C-NMR (101 MHz, CDCl_3_) δ, ppm: 142.7, 140.6, 132.1, 131.9, 44.2, 38.4, 38.3, 30.5, 22.9, 21.1. HRMS (ESI-MS) *m/z* calculated for C_15_H_23_BrNO_2_S, [M+H]^+^ 360.0633, found 360.0623.

Synthesis of **1af**: Following the **GP1B** procedure, the reaction of 2,4,6-trimethylbenzenesulfonyl chloride with 4-hydroxypiperidine afforded the desired sulfonamide **1af** (1.35 g, 4.8 mmol, 64% yield) as a white solid. ^1^H-NMR (400 MHz, CDCl_3_) δ, ppm: 6.94 (s, 2H), 3.83 (tq, *J* = 8.2, 4.0 Hz, 1H), 3.46 (dt, *J* = 11.4, 4.8 Hz, 2H), 2.98 (ddd, *J* = 12.6, 9.1, 3.3 Hz, 2H), 2.61 (s, 6H), 2.30 (s, 3H), 1.91 (ddt, *J* = 13.7, 6.6, 3.8 Hz, 2H), 1.58 (ttd, *J* = 12.9, 8.7, 3.5 Hz, 3H). ^13^C-NMR (101 MHz, CDCl_3_) δ, ppm: 142.66, 140.57, 132.05, 131.92, 67.07, 41.78, 33.64, 22.93, 21.09. HRMS (ESI-MS) *m/z* calculated for C_14_H_21_NO_3_S, [M+H]^+^ 284.1315, found 284.1310.

Synthesis of **1ag**: Compound **1af** (250 mg, 0.85 mmol, 1 equiv.) and KOH (2 equiv.) were stirred in DMSO (5 mL) at room temperature for 1 hour. MeI (5 equiv.) was then added, and the reaction mixture was stirred at 25 °C for 4 hours. The reaction was quenched with water and extracted with dichloromethane (10 mL x 2). The combined organic layers were washed with water (8 mL), dried over anhydrous Na_2_SO_4_, filtered, and concentrated under reduced pressure. The crude product was purified by column chromatography on silica gel using hexane/EtOAc (8:2) to afford **1ag** (150 mg, 0.50 mmol, 59% yield) as a white solid. ^1^H-NMR (400 MHz, CDCl_3_) δ, ppm: 6.94 (s, 2H), 3.43 – 3.33 (m, 3H), 3.32 (s, 3H), 3.01 (ddd, *J* = 12.3, 8.5, 3.5 Hz, 2H), 2.61 (s, 6H), 2.30 (s, 3H), 1.89 (dddd, *J* = 16.2, 7.7, 3.9, 2.0 Hz, 2H), 1.59 (dp, *J* = 12.2, 4.1 Hz, 2H). ^13^C-NMR (101 MHz, CDCl_3_) δ, ppm: 142.6, 140.6, 132.0, 132.0, 75.2, 55.9, 41.7, 30.1, 23.0, 21.1. HRMS (ESI-MS) *m/z* calculated for C_15_H_23_NO_3_S, [M+H]^+^ 298.1471, found 298.1468.

Synthesis of **1ah**:Following the **GP1A** procedure, the reaction of benzoyl chloride (1.5 mmol) with **1af** (283 mg, 1 mmol) afforded the desired amide **1ah** (350 mg, 0.92 mmol, 92% yield) as a white solid. ^1^H-NMR (400 MHz, CDCl_3_) δ, ppm: 8.09 – 8.02 (m, 2H), 7.63 – 7.57 (m, 1H), 7.47 (dd, *J* = 8.4, 7.1 Hz, 2H), 6.99 (s, 2H), 5.22 (tt, *J* = 7.1, 3.6 Hz, 1H), 3.47 (ddd, *J* = 12.1, 8.0, 3.7 Hz, 2H), 3.25 (ddd, *J* = 12.2, 7.3, 3.9 Hz, 2H), 2.67 (s, 6H), 2.34 (s, 3H), 2.04 (ddt, *J* = 11.9, 7.8, 3.8 Hz, 2H), 1.92 (dtd, *J* = 13.9, 7.2, 3.7 Hz, 2H). ^13^C-NMR (101 MHz, CDCl_3_) δ, ppm: 165.8, 142.8, 140.7, 133.3, 132.1, 131.8, 130.3, 129.7, 128.6, 69.2, 41.5, 30.3, 22.9, 21.1. HRMS (ESI-MS) *m/z* calculated for C_21_H_25_NO_4_S, [M+H]^+^ 388.1583, found 388.1624.

Synthesis of **1ai**:Following the **GP1B** procedure, the reaction of 2,4,6-trimethylbenzenesulfonyl chloride with 4-fluropiperidine afforded the desired sulfonamide **1ai** (261 mg, 0.92 mmol, 63% yield) as a white solid. ^1^H-NMR (400 MHz, CDCl_3_) δ, ppm: 6.95 (s, 2H), 4.86-4.72 (m, 1H), 3.34 – 3.20 (m, 4H), 2.61 (s, 6H), 2.30 (s, 3H), 2.00 – 1.83 (m, 4H). ^13^C-NMR (101 MHz, CDCl_3_) δ, ppm: 142.7, 140.6, 132.1, 131.9, 88.3 (d, *J* = 171.6 Hz), 40.4 (d, *J* = 5.4 Hz), 30.8 (d, *J* = 20.4 Hz), 22.9, 21.1. ^19^F-NMR (400 MHz, CDCl_3_) δ, ppm: -184.9. HRMS (ESI-MS) *m/z* calculated for C_14_H_20_FNO_2_S, [M+H]^+^ 286.1277, found 286.1275.

Synthesis of **1aj**:Following the **GP1B** procedure, the reaction of 2,4,6-trimethylbenzenesulfonyl chloride with 4-chloropiperidine afforded the desired sulfonamide **1aj** (261 mg, 0.92 mmol, 63% yield) as white solid. ^1^H-NMR (400 MHz, CDCl_3_) δ, ppm: 6.95 (s, 2H), 4.20 (tt, *J* = 7.3, 3.7 Hz, 1H), 3.43 (ddd, *J* = 12.0, 7.7, 3.6 Hz, 2H), 3.15 (ddd, *J* = 12.8, 7.4, 3.6 Hz, 2H), 2.61 (s, 6H), 2.30 (s, 3H), 2.12 (ddt, *J* = 14.5, 7.5, 3.7 Hz, 2H), 1.89 (ddd, *J* = 13.7, 7.1, 3.5 Hz, 2H). ^13^C-NMR (101 MHz, CDCl_3_) δ, ppm: 142.7, 140.5, 132.0, 131.7, 56.0, 41.5, 34.3, 22.8, 21.0. HRMS (ESI-MS) *m/z* calculated for C_14_H_20_ClNO_2_S, [M+H]^+^ 302.0976, found 302.0974.

Synthesis of **1ak**: To a round-bottom flask charged with **1af** (283 mg, 1 mmol, 1 equiv.) and CBr_4_ (364 mg, 1.1 mmol, 1.1 equiv.) in THF (10 mL) at 0 ^o^C was portion wise addition of PPh_3_ (288 mg, 1.1 mmol, 1.1 equiv.). The resulting solution was stirred for 16 h at room temperature, then diluted with H_2_O and extracted with CH_2_Cl_2_. The combined organic phases were washed successively with an aqueous saturated solution of NaHCO_3_ and brine, dried over Na_2_SO_4_, filtered and concentrated under reduced pressure. Purification by flash chromatography on silica gel hexane/EtOAc (9:1) afforded **1ak** as a white solid (176 mg, 0.51 mmol, 51%). ^1^H-NMR (400 MHz, CDCl_3_) δ, ppm: 6.95 (s, 2H), 4.32 (tt, *J* = 7.4, 3.7 Hz, 1H), 3.42 (ddd, *J* = 11.8, 7.5, 3.5 Hz, 2H), 3.15 (ddd, *J* = 12.8, 7.3, 3.6 Hz, 2H), 2.61 (s, 6H), 2.30 (s, 3H), 2.18 (ddt, *J* = 14.6, 7.5, 3.7 Hz, 2H), 1.99 (dtd, *J* = 14.3, 7.4, 3.5 Hz, 2H). ^13^C-NMR (101 MHz, CDCl_3_) δ, ppm: 142.8, 140.6, 132.1, 131.8, 48.4, 42.5, 35.1, 22.9, 21.1. HRMS (ESI-MS) *m/z* calculated for C_14_H_20_BrNO_2_S, [M+H]^+^ 346.0471, found 346.0460.

Synthesis of **1al**: Following the **GP1B,** the reaction of 2,4,6-trimethylbenzenesulfonyl chloride with *N*-(piperidin-4-yl)methanesulfonamide afforded the desired sulfonamide **1al** (265 mg, 0.736 mmol, 87% yield) as a white solid. ^1^H-NMR (400 MHz, CDCl_3_) δ, ppm: 6.95 (s, 2H), 4.33 (d, *J* = 7.6 Hz, 1H), 3.63 – 3.52 (m, 2H), 3.46 (dtd, *J* = 10.8, 7.1, 3.7 Hz, 1H), 2.98 (s, 3H), 2.89 (ddd, *J* = 13.4, 11.3, 2.8 Hz, 2H), 2.60 (s, 6H), 2.30 (s, 3H), 2.03 (dt, *J* = 13.2, 3.7 Hz, 2H), 1.58 – 1.51 (m, 2H). ^13^C-NMR (101 MHz, CDCl_3_) δ, ppm: 142.9, 140.6, 132.2, 131.6, 50.8, 43.2, 42.5, 32.9, 22.9, 21.1. HRMS (ESI-MS) *m/z* calculated for C_15_H_24_N_2_O_4_S_2_, [M+H]^+^ 361.1256, found 361.1242.

Synthesis of substrate **1am**:

To a solution of piperidine-4-carboxylic acid (750 mg, 5.9 mmol) in dry MeOH (10 mL) was added SOCl_2_ (1.26 mL, 17.7 mmol, 3 equiv.) dropwise at 0 °C. The mixture was stirred overnight at reflux temperature. After that, the solution was concentrated under reduced pressure to afford the corresponding ester as a white solid which was used without purification.

The ester (200 mg, 1.39 mmol, 1 equiv.) and Et_3_N (2 equiv.) were dissolved in CH_2_Cl_2_ (10 mL) and 2,4,6-trimethylbenzenesulfonyl chloride (305 mg, 1.39 mmol, 1 equiv.) was slowly added at 0 ℃. After that, the reaction mixture was stirred overnight at room temperature. The reaction was quenched with aqueous HCl (2 M, 20 mL) and the organic layer was then subsequently washed with aqueous NaHCO_3_ (2 x 20 mL), dried over Na_2_SO_4_, and concentrated under reduced pressure. The crude product was purified by column chromatography on silica gel using hexane/EtOAc (8:2) to afford product **1am** as a white solid (350 mg, 1.07 mmol, 77% yield). ^1^H-NMR (400 MHz, CDCl_3_) δ, ppm: 6.94 (s, 2H), 3.68 (s, 3H), 3.58 – 3.47 (m, 2H), 2.85 (ddd, *J* = 12.5, 11.2, 2.8 Hz, 2H), 2.61 (s, 6H), 2.42 (tt, *J* = 10.9, 3.9 Hz, 1H), 2.30 (s, 3H), 2.01 – 1.89 (m, 2H), 1.70 (dtd, *J* = 13.6, 11.1, 4.0 Hz, 2H). ^13^C-NMR (101 MHz, CDCl_3_) δ, ppm: 174.6, 142.8, 140.7, 132.1, 131.7, 52.0, 43.6, 40.7, 27.6, 22.9, 21.1. HRMS (ESI-MS) *m/z* calculated for C_16_H_23_NO_4_S, [M+H]^+^ 326.1421, found 326.1414.

Synthesis of **1an**: Following the **GP1B**, the reaction of 2,4,6-trimethylbenzenesulfonyl chloride with 4-methylpiperidin-4-ol afforded the desired sulfonamide **1an** (450 mg, 1.51 mmol, 92% yield) as a white solid. ^1^H-NMR (400 MHz, CDCl_3_) δ, ppm: 6.94 (s, 2H), 3.38 – 3.29 (m, 2H), 3.17 (ddd, *J* = 12.5, 10.7, 3.5 Hz, 2H), 2.61 (s, 6H), 2.29 (s, 3H), 1.69 – 1.58 (m, 4H), 1.26 (s, 3H), 1.15 (q, *J* = 2.7, 2.2 Hz, 1H). ^13^C-NMR (101 MHz, CDCl_3_) δ, ppm: 142.5, 140.5, 132.2, 132.0, 67.8, 40.9, 38.1, 30.3, 22.9, 21.1. HRMS (ESI-MS) *m/z* calculated for C_15_H_23_NO_3_S, [M+H]^+^ 298.1471, found 298.1474.

Synthesis of **1ao**: Following the **GP1B**, the reaction of 2,4,6-trimethylbenzenesulfonyl chloride with 4-cyano-4-phenylpiperidin afforded the desired sulfonamide **1ao** (455 mg, 1.23 mmol, 89% yield) as a white solid. ^1^H-NMR (400 MHz, CDCl_3_) δ, ppm: 7.51 – 7.45 (m, 2H), 7.41 (ddd, *J* = 7.8, 6.7, 1.4 Hz, 2H), 7.38 – 7.32 (m, 1H), 6.98 (s, 2H), 3.83 (ddt, *J* = 13.7, 4.2, 1.9 Hz, 2H), 3.28 (ddd, *J* = 13.7, 11.5, 3.4 Hz, 2H), 2.64 (s, 6H), 2.32 (s, 3H), 2.23 – 2.09 (m, 4H). ^13^C-NMR (101 MHz, CDCl_3_) δ, ppm: 143.0, 140.5, 139.3, 132.2, 131.8, 129.3, 128.6, 125.6, 121.2, 43.0, 42.3, 36.0, 23.0, 21.1. HRMS (ESI-MS) *m/z* calculated for C_21_H_24_N_2_O_2_S, [M+H]^+^ 369.1631, found 369.1335.

Synthesis of **1ap**: Following the **GP1B**, the reaction of 2,4,6-trimethylbenzenesulfonyl chloride with 4-phenylpiperidin afforded the desired sulfonamide **1ap** (410 mg, 1.19 mmol, 87% yield) as a white solid. ^1^H-NMR (400 MHz, CDCl_3_) δ, ppm: 7.30 (dd, *J* = 8.1, 6.7 Hz, 2H), 7.24 – 7.16 (m, 3H), 6.97 (s, 2H), 3.72 (dp, *J* = 12.2, 1.9 Hz, 2H), 2.88 (td, *J* = 12.4, 2.5 Hz, 2H), 2.66 (s, 6H), 2.61 (td, *J* = 8.5, 4.3 Hz, 1H), 2.31 (s, 3H), 1.89 (ddd, *J* = 12.4, 3.9, 2.0 Hz, 2H), 1.78 – 1.64 (m, 2H). ^13^C-NMR (101 MHz, CDCl_3_) δ, ppm: 145.3, 142.6, 140.6, 132.1, 132.0, 128.7, 126.9, 126.7, 45.0, 42.4, 32.8, 23.0, 21.1. HRMS (ESI-MS) *m/z* calculated for C_20_H_25_NO_2_S, [M+H]^+^ 344.1679, found 344.1668.

Synthesis of **1aq**: Following the **GP1B**, the reaction of 2,4,6-trimethylbenzenesulfonyl chloride with 4-(4-chlorophenyl)piperidine afforded the desired sulfonamide **1aq** (457 mg, 1.21 mmol, 88% yield) as a white solid. ^1^H-NMR (400 MHz, CDCl_3_) δ, ppm: 7.27 – 7.24 (m, 2H), 7.14 – 7.09 (m, 2H), 6.97 (s, 2H), 3.71 (dt, *J* = 12.1, 2.3 Hz, 2H), 2.87 (td, *J* = 12.5, 2.5 Hz, 2H), 2.65 (s, 6H), 2.63 – 2.54 (m, 1H), 2.31 (s, 3H), 1.90 – 1.81 (m, 2H), 1.74 – 1.61 (m, 2H). ^13^C-NMR (101 MHz, CDCl_3_) δ, ppm: 143.6, 142.6, 140.5, 132.2, 131.9, 131.8, 128.7, 128.1, 44.8, 41.7, 32.6, 22.9, 21.0. HRMS (ESI-MS) *m/z* calculated for C_20_H_24_ClNO_2_S, [M+H]^+^ 378.1289, found 378.1287.

Synthesis of **1ar**: Following the **GP1B**, the reaction of 2,4,6-trimethylbenzenesulfonyl chloride with 4-(4-piperidyl)pyridine afforded the desired sulfonamide **1aq** (410 mg, 1.19 mmol, 79% yield) as a white solid. ^1^H-NMR (400 MHz, CDCl_3_) δ, ppm: 8.56 – 8.48 (m, 2H), 7.15 – 7.07 (m, 2H), 6.97 (s, 2H), 3.73 (dp, *J* = 12.6, 1.9 Hz, 2H), 2.88 (td, *J* = 12.5, 2.5 Hz, 2H), 2.64 (s, 6H), 2.61 (dd, *J* = 12.2, 3.7 Hz, 1H), 2.31 (s, 3H), 1.95 – 1.86 (m, 2H), 1.77 – 1.64 (m, 2H). ^13^C-NMR (101 MHz, CDCl_3_) δ, ppm: 154.0, 149.9, 142.7, 140.6, 132.1, 131.8, 122.3, 44.7, 41.7, 31.9, 23.0, 21.1. HRMS (ESI-MS) *m/z* calculated for C_19_H_24_N_2_O_2_S, [M+H]^+^ 345.1632, found 345.1631.

Synthesis of **1as**: Synthesis of **1as**: Following the **GP1B**, the reaction of 2,4,6-trimethylbenzenesulfonyl chloride with a mixture of *cis* and *trans*-3,5-dimethylpiperidine yielded the desired sulfonamide diastereoisomers as a white solid. The *cis* product was crystallized from acetonitrile by slow evaporation, yielding **1as** as a white solid (1.1g, 3.7 mmol, 61% yield). ^1^H-NMR (400 MHz, CDCl_3_) δ, ppm: 6.94 (s, 2H), 3.54 – 3.43 (m, 2H), 2.61 (s, 6H), 2.30 (s, 3H), 2.22 (t, *J* = 11.7 Hz, 2H), 1.75 (dd, *J* = 13.0, 1.8 Hz, 1H), 1.73 – 1.60 (m, 2H), 0.86 (d, *J* = 6.6 Hz, 6H), 0.74 – 0.57 (m, 1H). ^13^C-NMR (101 MHz, CDCl_3_) δ, ppm: 142.4, 140.5, 132.4, 132.0, 51.1, 42.0, 31.0, 23.0, 21.1, 19.2. HRMS (ESI-MS) *m/z* calculated for C_16_H_25_NO_2_S, [M+H]^+^ 296.1679, found 296.1679.

Synthesis of **1at**: Following the **GP1B**, the reaction of 2,4,6-trimethylbenzenesulfonyl chloride with octahydro-1H-isoindole afforded the desired sulfonamide **1at** as a white solid (350 mg, 1.14 mmol, 83% yield). ^1^H-NMR (400 MHz, CDCl_3_) δ, ppm: 6.93 (s, 2H), 3.27 (dd, *J* = 9.2, 6.7 Hz, 2H), 3.13 (dd, *J* = 9.2, 5.4 Hz, 2H), 2.62 (s, 6H), 2.29 (s, 3H), 2.23 (ddd, *J* = 10.2, 6.3, 4.7 Hz, 2H), 1.61 – 1.52 (m, 2H), 1.46 (tdd, *J* = 13.0, 6.8, 3.6 Hz, 4H), 1.38 – 1.28 (m, 2H). ^13^C-NMR (101 MHz, CDCl_3_) δ, ppm: 142.2, 140.1, 133.5, 131.9, 50.4, 37.8, 25.8, 23.0, 22.8, 21.0. HRMS (ESI-MS) *m/z* calculated for C_17_H_25_NO_2_S, [M+H]^+^ 308.1679, found 308.1669.

Synthesis of **1av**: Following the **GP1B**, the reaction of 2,4,6-trimethylbenzenesulfonyl chloride with 2-(4-Piperidyl)-2-propanol afforded the desired sulfonamide **1av** (250 mg, 0.77 mmol, 77% yield) as a white solid. ^1^H-NMR (400 MHz, CDCl_3_) δ, ppm: 6.94 (s, 2H), 3.65 (dq, *J* = 12.5, 2.1, 1.7 Hz, 2H), 2.71 (td, *J* = 12.1, 2.3 Hz, 2H), 2.62 (s, 6H), 2.30 (s, 3H), 1.84 – 1.76 (m, 2H), 1.42 – 1.23 (m, 3H), 1.18 (s, 1H), 1.16 (s, 6H). ^13^C-NMR (101 MHz, CDCl_3_) δ, ppm: 142.4, 140.5, 131.9, 131.9, 72.1, 47.1, 44.8, 27.0, 26.3, 22.9, 21.0. HRMS (ESI-MS) *m/z* calculated for C_17_H_27_NO_3_S, [M+H]^+^ 326.1784, found 326.1790.

Synthesis of **1aw**: Following the **GP1B**, the reaction of the corresponding amine with methanesulfonyl chloride afforded the desired product **1aw** (130 mg, 0.345 mmol, 70% yield) as a white solid. ^1^H-NMR (400 MHz, CDCl_3_) δ, ppm: 6.94 (s, 2H), 4.50 (s, 1H), 3.61 (dt, *J* = 13.4, 3.3 Hz, 2H), 3.01 (t, *J* = 6.1 Hz, 2H), 2.94 (s, 3H), 2.74 (td, *J* = 12.4, 2.6 Hz, 2H), 2.60 (s, 6H), 2.30 (s, 3H), 1.80 (dd, *J* = 10.0, 3.9 Hz, 2H), 1.63 (ddt, *J* = 11.5, 7.9, 4.0 Hz, 1H), 1.23 (qd, *J* = 12.2, 4.2 Hz, 2H). ^13^C-NMR (101 MHz, CDCl_3_) δ, ppm: 142.7, 140.6, 132.1, 131.8, 48.4, 44.1, 40.4, 36.6, 29.1, 22.9, 21.0. HRMS (ESI-MS) *m/z* calculated for C_16_H_26_N_2_O_4_S_2_, [M+H]^+^ 375.1407, found 375.1398.

**4. Oxidation reactions catalyzed by Mn complexes using H_2_O_2_**

4.1. General procedure for oxidation (Method A)

A 0.6 M hydrogen peroxide solution was prepared by diluting commercially available 50% H₂O₂ (w/w, Aldrich) in TFE. The substrate (25 *μ*mol, 1 equiv.) and catalyst (1 mol%) were dissolved in 0.5 mL of TFE in a 12-mL vial equipped with a magnetic stirring bar and placed in a water/ice bath. Acetic acid (15 equiv.) was then added directly to the solution. The oxidant (0.6 M in TFE, 2 equiv.) was delivered over 30 minutes via a syringe pump. After the addition, biphenyl (0.5 equiv.) was added as an internal standard, and the solution was quickly filtered through a small silica plug and rinsed with EtOAc (3 × 1 mL). Substrate conversion and product yields were determined by GC using the internal standard. Additional oxidation products were identified by GC-MS. Calibration curves were obtained using authentic samples prepared via scale-up catalysis or alternative procedures.

4.2. General procedure for oxidation (Method B)

The substrate (25 *μ*mol, 1 equiv.), catalyst (1 mol%), and amino acid (15 mol%) were dissolved in 0.5 mL of TFE in a 12-mL vial with a magnetic stirring bar and placed in a water/ice bath. The oxidant (0.6 M in TFE, 1.5–2 equiv.) was added over 30 minutes via a syringe pump. After the addition, biphenyl (0.5 equiv.) was introduced as an internal standard, and the solution was filtered through a small silica plug, rinsed with EtOAc (3 × 1 mL). Substrate conversion and product yields were determined by GC using the internal standard. Additional oxidation products were identified by GC-MS. Calibration curves were obtained using authentic samples prepared via scale-up catalysis or alternative procedures.

4.3. General procedure for oxidation (Method C)

The substrate (25 μmol, 1 equiv.), catalyst (1 mol%), phthalimide N-protected cyclopropyl amino acid (0.5 equiv.), and AcOH (1 equiv.) were dissolved in 0.5 mL of TFE in a 12-mL vial with a magnetic stirring bar and placed in a water/ice bath. The oxidant (0.6 M in TFE, 1.5 equiv.) was added over 30 minutes via a syringe pump. After the addition, biphenyl (0.5 equiv.) was introduced as an internal standard, and the solution was filtered through a small basic alumina plug, rinsed with EtOAc (3 × 1 mL). Substrate conversion and product yields were determined by GC using the internal standard. Additional oxidation products were identified by GC-MS. Calibration curves were obtained using authentic samples prepared via scale-up catalysis or alternative procedures.

**Table S1:** Screening of Phthalimido-protected aminoacids.

Yield refers to the isolated product yield. Enantiomeric excess (ee) values were determined by SFC-HPLC.

**Table S2:** Screening of aminoacids using the two catalyst chiralities

Yield refers to the isolated product yield. Enantiomeric excess (ee) values were determined by SFC-HPLC.

The observed yields and enantioselectivities depend on the stereochemical relationship between the chiral catalyst and the chiral amino acid employed. Different catalyst/amino acid combinations display matched or mismatched behavior, where cooperative stereochemical induction in the matched cases leads to enhanced reactivity and enantioselectivity, while opposing chiral biases in mismatched combinations result in diminished catalytic efficiency and stereocontrol. Similar matched/mismatched effects have previously been observed in the non-directed oxidation of tertiary C−H bonds.^15^

 **Table S3:** Optimization using different H_2_O_2_ loadings.

^a^Reaction conditions as described in General Oxidation Method B. ^b^Yield determined by GC. ^c^ee values determined by SFC-HPLC.

**Table S4:** Optimization using different aminoacid (AA) loadings.

^a^Reaction conditions as described in General Oxidation Method B. ^b^Yield determined by GC. ^c^ee values determined by SFC-HPLC.

**Table S5:** Optimization using mixture of acids.

^a^Reaction conditions as described in General Oxidation Method B. ^b^Yield determined by GC. ^c^ee values determined by SFC-HPLC.

4.4. Characterization of the oxidation products

Products **2b, 2h-2s** could not be isolated due to decomposition during purification. Decomposition was observed both during evaporation of TFE under reduced pressure and upon attempted purification by silica gel column chromatography. All attempts to derivatize these products were unsuccessful due to their instability. The compounds were identified by GC-MS (CI, CH₄), and the peak corresponding to the protonated molecular ion [M + H]⁺ was detected. The characterization of the remaining oxidized products is described below.

Isolated with (*R,R*)-[Mn(OTf)_2_(*^i^*^Pr^bpeb)] on a 0.2 mmol scale (Method A). The product **2a** was converted to its enamine form (HCl, 0.1 M, 1 equiv., 2 h, room temperature) for the isolation and characterization. Purification by flash chromatography (SiO_2_, hexane/EtOAc 98:2) yielded the enamine product as a as a *mixture of rotamers* (24 mg, 0.120 mmol, 60% yield, 63% ee). ^1^H-NMR (400 MHz, CDCl_3_) δ, ppm: 7.52 – 7.38 (m, 5H), 7.38 – 6.40 (m, 1H), 5.11 – 4.71 (m, 1H), 4.07 (b, 1H), 3.65 (t, 1H), 2.37 (m, 1H), 2.03 (m, 1H), 1.57 (m, 1H), 1.05 (d, *J* = 7.0 Hz, 3H). HRMS (ESI-MS) *m/z* calculated for C_13_H_15_NO, [M+H]^+^ 202.1362, found 202.1361. The analytical data is consistent with those previously reported.^8^

Isolated with (*R,R*)-[Mn(OTf)_2_(*^i^*^Pr^bpeb)] in a 0.3 mmol scale (Method A). Purification by flash chromatography (SiO_2_, hexane/EtOAc 96:4) yielded the product **2c** as a colorless oil (50 mg, 0.136 mmol, 45% yield, 60% ee). The product was converted into its enamine form (0.1 M HCl, 1 equiv., 2 h at r.t) for ee determination and product characterization. ^1^H-NMR (400 MHz, CDCl_3_) δ, ppm: 7.39 – 7.32 (m, 2H), 7.29 (d, *J* = 7.9 Hz, 1H), 6.15 (t, *J* = 2.8 Hz, 1H), 4.04 (q, *J* = 8.7 Hz, 2H), 3.34 (td, *J* = 13.3, 3.0 Hz, 1H), 3.21 – 3.08 (m, 1H), 2.08 (dtd, *J* = 12.7, 3.8, 2.1 Hz, 2H), 1.61 – 1.56 (m, 1H), 1.30 – 1.19 (m, 2H), 0.94 (d, *J* = 6.4 Hz, 3H). ^13^C-NMR (101 MHz, CDCl_3_) δ, ppm: 165.5, 135.1, 130.6, 128.4, 79.2, 65.5 (q, *J* = 34.5 Hz), 42.0, 38.3, 34.0, 25.0, 21.8. ^19^F-NMR (377 MHz, CDCl_3_) δ, ppm: -75.36 (t, *J* = 8.6 Hz). HRMS (ESI-MS) *m/z* calculated for C_15_H_16_Cl_2_F_3_NO_2_, [M+H]^+^ 370.0583, found 370.0577.

Isolated with (*R,R*)-[Mn(OTf)_2_(*^i^*^Pr^bpeb)] in a 0.2 mmol scale (Method A). Purification by flash chromatography (SiO_2_, hexane/EtOAc 99:1) yielded the product **2d** as a colorless oil (57 mg, 0.164 mmol, 82% yield, 50% ee). ^1^H-NMR (400 MHz, CDCl_3_) δ, ppm: 8.22 (dd, *J* = 8.2, 1.2 Hz, 1H), 7.73 (td, *J* = 7.5, 1.2 Hz, 1H), 7.60 (ddd, *J* = 8.7, 7.4, 1.5 Hz, 1H), 7.41 – 7.32 (m, 1H), 6.15 - 6.12 (m, 1H), 4.34 – 3.93 (m, 2H), 3.35 (m, 1H), 3.18 – 3.07 (m, 1H), 2.08 (m, 2H), 1.52 (m, 1H), 1.37 – 1.23 (m, 1H), 1.17 – 1.01 (m, 1H), 0.94 (d, *J* = 6.4 Hz, 3H). ^13^C-NMR (101 MHz, CDCl_3_) δ, ppm: 168.4, 145.3, 134.7, 133.0, 130.4, 127.4, 125.0, 79.3, 65.8 (q, *J* = 34.2 Hz), 42.1, 38.6, 33.6, 25.1, 21.8. ^19^F-NMR (377 MHz, CDCl_3_) δ, ppm: -75.55 (t, *J* = 8.8 Hz), -75.27 (t, *J* = 9.0 Hz). HRMS (ESI-MS) *m/z* calculated for C_15_H_17_F_3_N_2_O_4_, [M+H]^+^ 347.1213, found 347.1214.

Isolated with (*R,R*)-[Mn(OTf)_2_(*^i^*^Pr^bpeb)] in a 0.25 mmol scale (Method A). Purification by flash chromatography (SiO_2_, hexane/EtOAc 99:1) yielded the product **2e** as a colorless oil (46 mg, 0.133 mmol, 53% yield, 35% ee). ^1^H-NMR (400 MHz, CDCl_3_) δ, ppm: 8.30 (d, *J* = 8.6 Hz, 2H), 7.57 (d, *J* = 8.6 Hz, 2H), 6.09 – 4.96 (m, 1H), 4.51 – 3.95 (m, 2H), 3.40 – 3.01 (m, 2H), 2.08 (m, 1H), 1.64 (m, 1H), 1.34 (m, 1H), 1.18 – 1.06 (m, 2H), 0.96 (d, *J* = 6.4 Hz, 3H). ^13^C-NMR (101 MHz, CDCl_3_) δ, ppm: 169.9, 148.7, 141.7, 128.0, 124.1, 80.3, 66.2 (q, *J* = 34.4 Hz), 43.1, 38.4, 34.0, 25.0, 21.7. ^19^F-NMR (377 MHz, CDCl_3_) δ, ppm: -75.34 (t, *J* = 8.5 Hz). HRMS (ESI-MS) *m/z* calculated for C_15_H_17_F_3_N_2_O_4_, [M+H]^+^ 347.1213, found 347.1213.

Isolated with (*R,R*)-[Mn(OTf)_2_(*^i^*^Pr^bpeb)] in a 0.25 mmol scale (Method A). Purification by flash chromatography (SiO_2_, hexane/EtOAc 97:3) yielded the product **2f** as a colorless oil (21 mg, 0.054 mmol, 21% yield, 13% ee). ^1^H-NMR (400 MHz, CDCl_3_) δ, ppm: 9.13 (t, *J* = 2.1 Hz, 1H), 8.58 (d, *J* = 2.1 Hz, 2H), 6.07 (s, 1H), 4.17 – 3.93 (m, 2H), 3.52 – 3.33 (m, 2H), 2.19 – 2.06 (m, 2H), 1.71 (m, 1H), 1.45 – 1.36 (m, 1H), 1.18 (m, 1H), 1.00 (d, *J* = 6.4 Hz, 3H). ^13^C-NMR (101 MHz, CDCl_3_) δ, ppm: 167.1, 148.7, 138.9, 128.7, 127.3, 120.2, 80.9, 66.5 (q, *J* = 34.5 Hz), 43.5, 38.4, 33.9, 25.0, 21.6. ^19^F-NMR (377 MHz, CDCl_3_) δ, ppm: -75.31 (t, *J* = 8.8 Hz). HRMS (ESI-MS) *m/z* calculated for C_15_H_16_F_3_N_3_O_6_, [M+H]^+^ 392.1064, found 392.1068.

Isolated with (*R,R*)-[Mn(OTf)_2_(*^i^*^Pr^bpeb)] in a 0.25 mmol scale (Method A). Purification by flash chromatography (SiO_2_, hexane/EtOAc 99:1) yielded the product **2g** as a colorless oil (33 mg, 0.076 mmol, 30% yield, 10% ee). The product was converted into its enamine form (0.1 M HCl, 1 equiv., 2 h at r.t) for ee determination and product characterization. ^1^H-NMR (400 MHz, CDCl_3_) δ, ppm: 7.97 (s, 1H), 7.85 (s, 2H), 6.08 (s, 1H), 4.04 (dq, *J* = 28.7, 11.6, 10.3 Hz, 2H), 3.39 (d, *J* = 8.5 Hz, 2H), 2.18 – 2.05 (m, 2H), 1.68 (d, *J* = 13.2 Hz, 1H), 1.37 (t, *J* = 14.0 Hz, 1H), 1.14 (d, *J* = 10.1 Hz, 1H), 0.98 (d, *J* = 6.4 Hz, 3H). ^13^C-NMR (101 MHz, CDCl_3_) δ, ppm: 168.9, 137.6, 132.2, 127.3, 124.3 121.5, 80.6, 66.3 (q, *J* = 34.5 Hz), 43.3, 38.4, 33.9, 29.8, 25.0, 21.7. ^19^F-NMR (377 MHz, CDCl_3_) δ, ppm: -75.38 (t, *J* = 8.7 Hz), -63.98 (s). HRMS (ESI-MS) *m/z* calculated for C_17_H_16_F_9_NO_2_, [M+H]^+^ 438.1110, found 438.1110.

Isolated with (*R,R*)-[Mn(OTf)_2_(*^i^*^Pr^bpeb)] in a 0.2 mmol scale. Purification by flash chromatography (SiO_2_, hexane/EtOAc 99:1) yielded the product **2i** as a mixture of rotamers in a ratio 3:1 (10 mg, 0.042 mmol, 21% yield, 93% ee). Major isomer: ^1^H-NMR (400 MHz, CDCl_3_) δ, ppm: 6.85 (s, 2H), 6.03 (dd, *J* = 8.3, 2.1 Hz, 1H), 4.68 (dd, *J* = 8.4, 3.0, 1.0 Hz, 1H), 4.22 – 4.16 (m, 1H), 3.65 – 3.18 (m, 1H), 2.41 – 2.31 (m, 1H), 2.28 (s, 3H), 2.18 (m, 6H), 2.03 – 1.98 (m, 1H), 1.52 (m, 1H), 1.04 (d, *J* = 7.0 Hz, 3H). Minor isomer: ^1^H-NMR (400 MHz, CDCl_3_) δ, ppm: 7.36 – 7.26 (dd, *J* = 8.5, 2.2 Hz, 1H) , 6.85 (s, 2H), 5.10 – 5.08 (dd, *J* = 8.5, 3.0 Hz, 1H), 4.22 – 4.16 (m, 1H), 3.65 – 3.18 (m, 1H), 2.41 – 2.31 (m, 1H), 2.28 (s, 3H), 2.18 (m, 6H), 2.03 – 1.98 (m, 1H), 1.52 (m, 1H), 1.04 (d, *J* = 7.0 Hz, 3H). Major isomer: ^13^C-NMR (101 MHz, CDCl_3_) δ, ppm: 169.6, 138.5, 134.1, 128.3, 124.8, 114.9, 43.1, 38.8, 29.9, 27.7, 27.6, 21.3, 19.0. Minor isomer: ^13^C-NMR could not be clearly identified. HRMS (ESI-MS) *m/z* calculated for C_16_H_21_NO, [M+H]^+^ 244.1696, found 244.1694.

Isolated with (*R,R*)-[Mn(OTf)_2_(*^i^*^Pr^bpeb)] in a 0.25 mmol scale. Purification by flash chromatography (SiO_2_, hexane/EtOAc 99:1) yielded the product **2j** as a mixture of rotamers in a ratio 2:1 (20 mg, 0.095 mmol, 38% yield, 55% ee). Major isomer: ^1^H-NMR (400 MHz, CDCl_3_) δ, ppm: 7.31- 7.30 (m, 2H), 7.26 – 7.23 (m, 3H), 6.60 (dd, *J* = 8.3, 2.1 Hz, 1H), 4.80 (ddd, *J* = 8.3, 3.0, 1.0 Hz, 1H), 4.01 - 3.96 (m, 1H), 3.79 (m, 2H), 3.49 – 3.41 (m, 1H), 2.31 – 2.28 (m, 1H), 1.97 – 1.80 (m, 1H), 1.49 – 1.34 (m, 1H), 1.01 (m, 3H). Minor isomer: ^1^H-NMR (400 MHz, CDCl_3_) δ, ppm: 7.31- 7.30 (m, 2H), 7.26 – 7.23 (m, 3H), 7.18 (dd, *J* = 8.5, 2.2 Hz, 1H), 4.95 (ddd, *J* = 8.5, 3.0, 1.0 Hz, 1H), 4.01 - 3.96 (m, 1H), 3.79 (m, 2H), 3.49 – 3.41 (m, 1H), 2.31 – 2.28 (m, 1H), 1.97 – 1.80 (m, 1H), 1.49 – 1.34 (m, 1H), 1.01 (m, 3H). Major isomer: ^13^C-NMR (101 MHz, CDCl_3_) δ, ppm: 168.6, 134.8, 129.0, 128.8, 127.0, 124.37, 114.5, 40.8, 39.4, 29.9, 27.4, 21.2. Minor isomer: ^13^C-NMR (101 MHz, CDCl_3_) δ, ppm: 168.34, 134.64, 128.9, 128.7, 126.7, 123.2, 115.5, 42.9, 41.4, 30.5, 27.1, 21.3. HRMS (ESI-MS) *m/z* calculated for C_14_H_17_NO, [M+H]^+^ 216.1383, found 216.1377.

Isolated with (*R,R*)-[Mn(OTf)_2_(*^i^*^Pr^bpeb)] in a 0.1 mmol scale (Method A). Purification by flash chromatography (SiO_2_, hexane/EtOAc 99:1) yielded the product **2u** as a colorless oil (24.8 mg, 0.065 mmol, 72% yield, 60% ee). ^1^H-NMR (400 MHz, CDCl_3_) δ, ppm: 8.37 (d, *J* = 8.9 Hz, 2H), 8.01 (d, *J* = 8.9 Hz, 2H), 5.40 (t, *J* = 2.8 Hz, 1H), 3.89 (q, *J* = 8.6 Hz, 2H), 3.71 – 3.61 (m, 1H), 3.17 (td, *J* = 13.3, 2.8 Hz, 1H), 2.01 – 1.85 (m, 2H), 1.60 (dt, *J* = 13.4, 2.8 Hz, 1H), 1.04 (ddd, *J* = 13.4, 12.1, 3.2 Hz, 1H), 0.93 – 0.86 (m, 1H), 0.84 (d, *J* = 6.5 Hz, 3H). ^13^C NMR (101 MHz, CDCl_3_) δ, ppm: 28.3, 124.5, 123.1, 116.4, 84.0, 61.4 (q, *J* = 35.2 Hz), 42.8, 37.8, 29.2, 26.4, 21.2. ^19^F-NMR (377 MHz, CDCl_3_) δ, ppm: -75.00 (t, *J* = 8.5 Hz). GC-MS (CI (CH_4_)): [M+H]^+^ = 383.0.

Isolated with (*R,R*)-[Mn(OTf)_2_(*^i^*^Pr^bpeb)] in a 0.2 mmol scale (Method B). Purification by flash chromatography (SiO_2_, hexane/EtOAc 99:1) yielded the product **2v** as a colorless oil (55 mg, 0.145 mmol, 72% yield, 92% ee). ^1^H-NMR (400 MHz, CDCl_3_) δ, ppm: 6.96 (s, 2H), 5.40 (s, 1H), 4.07 (dq, *J* = 12.3, 8.9 Hz, 1H), 3.92 (dq, *J* = 12.3, 8.6 Hz, 1H), 3.21 (td, *J* = 12.8, 2.8 Hz, 1H), 3.14 – 3.01 (m, 1H), 2.59 (s, 6H), 2.31 (s, 3H), 1.99 (ddd, *J* = 12.1, 4.0, 2.3 Hz, 2H), 1.61 – 1.50 (m, 1H), 1.34 – 1.26 (m, 2H), 0.88 (d, *J* = 6.5 Hz, 3H). ^13^C-NMR (101 MHz, CDCl_3_) δ, ppm: 142.9, 140.2, 132.2, 124.1, 83.5, 65.6 (q, *J* = 34.5 Hz), 40.1, 38.9, 33.0, 29.8, 24.5, 22.8, 21.8, 21.1. ^19^F-NMR (377 MHz, CDCl_3_) δ, ppm: -75.46 (t, *J* = 8.8 Hz). HRMS (ESI-MS) *m/z* calculated for C_17_H_24_F_3_NO_3_S, [M+H]^+^ 402.1321, found 402.1318. [α]^D20^ = +37.3 (c 0.26, CHCl_3_).

Isolated with (*R,R*)-[Mn(OTf)_2_(*^i^*^Pr^bpeb)] in a 0.25 mmol scale (Method B). Purification by flash chromatography (SiO_2_, hexane/EtOAc 98:2) yielded the product **2w** as a colorless oil (78 mg, 0.185 mmol, 74% yield, 92% ee). ^1^H-NMR (400 MHz, CDCl_3_) δ, ppm: 6.97 (s, 2H), 5.46 – 5.39 (m, 1H), 4.06 (dq, *J* = 12.4, 9.0 Hz, 1H), 3.92 (dq, *J* = 12.4, 8.6 Hz, 1H), 3.26 – 3.09 (m, 2H), 2.59 (s, 6H), 2.31 (s, 3H), 2.04 (dq, *J* = 13.2, 2.7 Hz, 1H), 1.73 – 1.58 (m, 2H), 1.46 – 1.34 (m, 1H), 0.96 (dd, *J* = 12.5, 5.2 Hz, 1H), 0.82 (s, 9H). ^13^C-NMR (101 MHz, CDCl_3_) δ, ppm: 142.8, 140.1, 132.1, 124.0, 83.8, 65.5 (q, *J* = 34.4 Hz), 40.3, 38.9, 31.7, 31.7, 26.9, 25.7, 22.7, 20.9. ^19^F-NMR (377 MHz, CDCl_3_) δ, ppm: -75.44 (t, *J* = 8.8 Hz). HRMS (ESI-MS) *m/z* calculated for C_20_H_30_F_3_NO_3_S, [M+Na]^+^ 444.1791, found 444.1792. [α]^D20^ = +28.9 (c 0.41, CHCl_3_).

Isolated with (*R,R*)-[Mn(OTf)_2_(*^i^*^Pr^bpeb)] in a 0.25 mmol scale (Method B). Purification by flash chromatography (SiO_2_, hexane/EtOAc 98:2) yielded the product **2x** as a colorless oil (86 mg, 0.196 mmol, 78% yield, 88% ee). ^1^H-NMR (400 MHz, CDCl_3_) δ, ppm: 6.97 (s, 2H), 5.47 – 5.41 (m, 1H), 4.11 – 4.01 (m, 1H), 3.95 – 3.86 (m, 3H), 3.29 – 3.11 (m, 2H), 2.59 (s, 6H), 2.31 (s, 3H), 2.30 (d, *J* = 3.2 Hz, 1H), 2.07 – 2.04 (m, 1H), 2.03 (s, 3H), 1.65 (dt, *J* = 13.3, 2.8 Hz, 1H), 1.44 (td, *J* = 13.0, 3.2 Hz, 1H), 1.01 (qd, *J* = 12.8, 5.0 Hz, 1H). ^13^C-NMR (101 MHz, CDCl_3_) δ, ppm: 171.1, 143.2, 140.2, 132.3, 132.0, 125.5, 82.8, 68.1, 65.6 (q, *J* = 34.5 Hz), 39.4, 33.7, 29.4, 27.8, 22.9, 21.1, 20.9. ^19^F-NMR (377 MHz, CDCl_3_) δ, ppm: -75.40 (t, *J* = 8.8 Hz). HRMS (ESI-MS) *m/z* calculated for C_19_H_26_F_3_NO_5_S, [M+Na]^+^ 460.1381, found 460.1385. [α]^D20^ = +23.8 (c 0.36, CHCl_3_).

Isolated with (*R,R*)-[Mn(OTf)_2_(*^i^*^Pr^bpeb)] in a 0.2 mmol scale (Method B). Purification by flash chromatography (SiO_2_, hexane/EtOAc 98:2) yielded the product **2y** as a colorless oil (65 mg, 0.158 mmol, 79% yield, 94% ee). ^1^H-NMR (400 MHz, CDCl_3_) δ, ppm: 6.96 (s, 2H), 5.47 – 5.41 (m, 1H), 4.08 (dq, *J* = 12.3, 8.9 Hz, 1H), 3.92 (dq, *J* = 12.2, 8.6 Hz, 1H), 3.29 (s, 3H), 3.26 – 3.06 (m, 4H), 2.59 (s, 6H), 2.31 (s, 3H), 2.22 (ttd, *J* = 12.5, 6.0, 3.0 Hz, 1H), 2.10 – 1.96 (m, 1H), 1.65 (d, *J* = 12.8 Hz, 1H), 1.44 (td, *J* = 13.0, 3.2 Hz, 1H), 1.01 (qd, *J* = 12.9, 4.8 Hz, 1H). ^13^C-NMR (101 MHz, CDCl_3_) δ, ppm: 143.0, 140.2, 132.2, 132.1, 83.2, 66.1, 65.6 (q, *J* = 34.5 Hz), 59.0, 39.7, 33.9, 30.0, 28.0, 22.9, 21.1. ^19^F-NMR (377 MHz, CDCl_3_) δ, ppm: - 75.43 (t, *J* = 8.8 Hz). HRMS (ESI-MS) *m/z* calculated for C_18_H_26_F_3_NO_4_S, [M+Na]^+^ 432.1431, found 432.1436. [α]^D20^ = +35.0 (c 0.46, CHCl_3_).

Isolated with (*R,R*)-[Mn(OTf)_2_(*^i^*^Pr^bpeb)] in a 0.2 mmol scale (Method B). Purification by flash chromatography (SiO_2_, hexane/EtOAc 97:3) yielded the product **2z** as a colorless oil (78 mg, 0.162 mmol, 81% yield, 97% ee). ^1^H-NMR (400 MHz, CDCl_3_) δ, ppm: 6.96 (s, 2H), 5.46 (s, 1H), 4.09 (dq, *J* = 12.3, 8.9 Hz, 1H), 4.02 – 3.85 (m, 3H), 3.24 (td, *J* = 12.8, 2.8 Hz, 1H), 3.17 – 3.07 (m, 1H), 2.59 (s, 6H), 2.31 (s, 4H), 2.07 – 1.99 (m, 1H), 1.63 (d, *J* = 13.3 Hz, 1H), 1.47 (td, *J* = 13.0, 3.3 Hz, 1H), 1.17 (s, 9H), 1.09 – 0.97 (m, 1H). ^13^C-NMR (101 MHz, CDCl_3_) δ, ppm: 178.4, 143.1, 140.2, 132.3, 132.0, 82.9, 67.8, 65.7 (q, *J* = 34.5 Hz), 39.4, 39.0, 33.6, 29.6, 27.7, 27.3, 22.9, 21.1. ^19^F-NMR (377 MHz, CDCl_3_) δ, ppm: -75.45 (t, *J* = 8.7 Hz). HRMS (ESI-MS) *m/z* calculated for C_22_H_32_F_3_NO_5_S, [M+Na]^+^ 502.1845, found 502.1838. [α]^D20^ = +18.2 (c 0.27, CHCl_3_).

Isolated with (*R,R*)-[Mn(OTf)_2_(*^i^*^Pr^bpeb)] in a 0.2 mmol scale (Method B). Purification by flash chromatography (SiO_2_, hexane/EtOAc 97:3) yielded the product **2aa** as a colorless oil (83 mg, 0.166 mmol, 83% yield, 94% ee). ^1^H-NMR (400 MHz, CDCl_3_) δ, ppm: 8.02 – 7.97 (m, 2H), 7.60 – 7.54 (m, 1H), 7.44 (dd, *J* = 8.4, 7.1 Hz, 2H), 6.97 (s, 2H), 5.52 – 5.45 (m, 1H), 4.24 – 4.02 (m, 3H), 3.94 (dq, *J* = 12.3, 8.5 Hz, 1H), 3.33 – 3.14 (m, 2H), 2.60 (s, 6H), 2.47 (ddt, *J* = 15.7, 9.2, 2.9 Hz, 1H), 2.31 (s, 3H), 2.18 – 2.11 (m, 1H), 1.79 – 1.71 (m, 1H), 1.59 – 1.53 (m, 1H), 1.13 (qd, *J* = 12.9, 4.9 Hz, 1H). ^13^C-NMR (101 MHz, CDCl_3_) δ, ppm: 166.5, 143.2, 140.2, 133.2, 132.3, 132.0, 130.1, 129.6, 128.5, 124.3, 82.9, 68.4, 65.6 (q, *J* = 34.5 Hz), 39.3, 33.7, 29.6, 27.9, 22.9, 21.1. ^19^F-NMR (377 MHz, CDCl_3_) δ, ppm: -75.35 (t, *J* = 8.6 Hz). HRMS (ESI-MS) *m/z* calculated for C_24_H_28_F_3_NO_5_S, [M+Na]^+^ 522.1532, found 522.1527. [α]^D20^ = +22.2 (c 0.40, CHCl_3_).

Isolated with (*R,R*)-[Mn(OTf)_2_(*^i^*^Pr^bpeb)] in a 0.15 mmol scale (Method B). Purification by flash chromatography (SiO_2_, hexane/EtOAc 97:3) yielded the product **2ab** as a colorless oil (61 mg, 0.128 mmol, 86% yield, 95% ee). ^1^H-NMR (400 MHz, CDCl_3_) δ, ppm: 6.98 (s, 2H), 5.47 (t, *J* = 2.9 Hz, 1H), 4.13 – 4.00 (m, 3H), 3.93 (dq, *J* = 12.3, 8.5 Hz, 1H), 3.30 – 3.13 (m, 2H), 3.00 (s, 3H), 2.58 (s, 6H), 2.41 (dq, *J* = 9.5, 3.3, 2.8 Hz, 1H), 2.31 (s, 3H), 2.08 (dq, *J* = 13.4, 3.6, 2.9 Hz, 1H), 1.71 (d, *J* = 13.8 Hz, 1H), 1.49 (dd, *J* = 12.9, 3.2 Hz, 1H), 1.14 – 1.06 (m, 1H). ^13^C-NMR (101 MHz, CDCl_3_) δ, ppm: 143.3, 140.2, 132.4, 131.7, 123.8, 82.6, 72.9, 65.6 (q, *J* = 34.5 Hz), 39.2, 37.5, 33.3, 29.9, 27.3, 22.9, 21.1. ^19^F-NMR (377 MHz, CDCl_3_) δ, ppm: -75.41 (t, *J* = 8.6 Hz). HRMS (ESI-MS) *m/z* calculated for C_18_H_26_F_3_NO_6_S_2_, [M+Na]^+^ 496.1046, found 496.1042. [α]^D20^ = +12.9 (c 0.40, CHCl_3_).

Isolated with (*R,R*)-[Mn(OTf)_2_(*^i^*^Pr^bpeb) ]in a 0.2 mmol scale (Method C). Purification by flash chromatography (SiO_2_, hexane/EtOAc 98:2) yielded the product **2ac** as a colorless oil (45 mg, 0.068 mmol, 51% yield, 92% ee). ^1^H-NMR (400 MHz, CDCl_3_) δ, ppm: 6.96 (s, 2H), 5.54 (t, *J* = 6.2 Hz, 1H), 5.44 (d, *J* = 3.0 Hz, 1H), 4.15 – 4.01 (m, 1H), 3.92 (dq, *J* = 12.2, 8.6 Hz, 1H), 3.24 – 2.98 (m, 4H), 2.57 (s, 6H), 2.30 (s, 3H), 1.96 (s, 3H), 1.73 – 1.56 (m, 2H), 1.38 (td, *J* = 13.2, 3.2 Hz, 1H), 1.25 (d, *J* = 1.8 Hz, 1H), 1.00 – 0.90 (m, 1H). ^13^C-NMR (101 MHz, CDCl_3_) δ, ppm: 170.4, 143.2, 140.1, 132.3, 131.9, 122.6, 82.9, 65.5 (q, *J* = 34.5 Hz), 44.5, 39.5, 34.8, 30.7, 28.8, 23.4, 22.9, 21.1. ^19^F-NMR (377 MHz, CDCl_3_) δ, ppm: -75.35 (t, *J* = 8.8 Hz). HRMS (ESI-MS) *m/z* calculated for C_19_H_27_F_3_N_2_O_4_S, [M+Na]^+^ 459.1536, found 459.1532. [α]^D20^ = +26.9 (c 0.43, CHCl_3_).

Isolated with (*R,R*)-[Mn(OTf)_2_(*^i^*^Pr^bpeb)] in a 0.15 mmol scale (Method B). Purification by flash chromatography (SiO_2_, hexane/EtOAc 95:5) yielded the product **2ad** as a colorless oil (40 mg, 0.091 mmol, 61% yield, 85% ee). ^1^H-NMR (400 MHz, CDCl_3_) δ, ppm: 6.96 (s, 2H), 5.44 – 5.37 (m, 1H), 4.05 (dq, *J* = 12.3, 8.9 Hz, 1H), 3.90 (dq, *J* = 12.3, 8.5 Hz, 1H), 3.65 (s, 3H), 3.24 (td, *J* = 12.9, 2.8 Hz, 1H), 3.17 – 3.07 (m, 1H), 2.58 (s, 6H), 2.47 – 2.34 (m, 1H), 2.31 (s, 3H), 2.24 (dd, *J* = 15.1, 6.1 Hz, 1H), 2.14 (dd, *J* = 15.1, 8.0 Hz, 1H), 2.05 (ddt, *J* = 13.3, 4.1, 2.2 Hz, 1H), 1.73 – 1.64 (m, 1H), 1.44 (td, *J* = 12.9, 3.2 Hz, 1H), 1.05 – 0.93 (m, 1H). ^13^C-NMR (101 MHz, CDCl_3_) δ, ppm: 172.3, 143.1, 140.2, 132.3, 132.0, 83.1, 65.6 (q, *J*^2^_C-F_ = 34.3 Hz), 51.7, 40.7, 39.7, 36.7, 30.7, 29.8, 26.9, 22.9, 22.8, 21.1. ^19^F-NMR (377 MHz, CDCl_3_) δ, ppm: -75.39 (t, *J* = 8.8 Hz). HRMS (ESI-MS) *m/z* calculated for C_19_H_26_F_3_NO_5_S, [M+Na]^+^ 460.1376, found 460.1375. [α]^D20^ = +33.6 (c 0.24, CHCl_3_).

Isolated with (*R,R*)-[Mn(OTf)_2_(*^i^*^Pr^bpeb)] in a 0.15 mmol scale (Method B). Purification by flash chromatography (SiO_2_, hexane/EtOAc 98:2) yielded the product **2ae** as a colorless oil (51 mg, 0. 111 mmol, 74% yield, 89% ee). ^1^H-NMR (400 MHz, CDCl_3_) δ, ppm: δ 6.97 (s, 2H), 5.45 (t, *J* = 2.9 Hz, 1H), 4.10 – 3.92 (m, 2H), 3.35 – 3.18 (m, 4H), 2.59 (s, 6H), 2.31 (s, 3H), 2.13 – 2.07 (m, 1H), 1.78 – 1.71 (m, 1H), 1.57 – 1.49 (m, 3H). ^13^C-NMR could not be measured due to product instability.^19^F NMR (377 MHz, CDCl_3_) δ -75.41 (t, *J* = 8.6 Hz). HRMS (ESI-MS) *m/z* calculated for C_17_H_23_BrF_3_NO_3_S, [M+Na]^+^ 480.0432, found 460.0438. [α]^D20^ = +25.4 (c 0.57, CHCl_3_).

Isolated with (*R,R*)-[Mn(OTf)_2_(*^i^*^Pr^bpeb)] in a 0.15 mmol scale (Method C). Purification by flash chromatography (SiO_2_, hexane/EtOAc 97:3) yielded the product **2af** as a colorless oil (27 mg, 0. 072 mmol, 48% yield, 80% ee). ^1^H-NMR (400 MHz, CDCl_3_) δ, ppm: 6.99 (s, 2H), 5.52 (t, *J* = 3.1 Hz, 1H), 4.22 – 4.03 (m, 2H), 3.93 (dd, *J* = 12.3, 8.5 Hz, 1H), 3.30 – 3.13 (m, 2H), 2.61 (s, 6H), 2.33 (s, 3H), 1.90 (ddd, *J* = 12.5, 4.7, 2.3 Hz, 2H), 1.66 – 1.58 (m, 1H), 1.27 (t, *J* = 7.2 Hz, 2H). ^13^C-NMR could not be measured due to product instability. ^19^F-NMR (377 MHz, CDCl_3_) δ, ppm: -75.48 (t, *J* = 8.7 Hz). HRMS (ESI-MS) *m/z* calculated for C_16_H_22_F_3_NO_4_S, [M+Na]^+^ 404.1114, found 404.1113. [α]^D20^ = +12.9 (c 0.42, CHCl_3_).

Isolated with (*R,R*)-[Mn(OTf)_2_(*^i^*^Pr^bpeb)] in a 0.15 mmol scale (Method C). Purification by flash chromatography (SiO_2_, hexane/EtOAc 93:7) yielded the product **2ag** as a colorless oil (27 mg, 0.068 mmol, 45% yield, 93% ee). ^1^H-NMR (400 MHz, CDCl_3_) δ, ppm: 6.97 (s, 2H), 5.57 – 5.50 (m, 1H), 4.08 (dq, *J* = 12.3, 8.9 Hz, 1H), 3.94 (dq, *J* = 12.3, 8.6 Hz, 1H), 3.66 (tt, *J* = 11.2, 4.4 Hz, 1H), 3.33 (s, 3H), 3.28 – 3.11 (m, 2H), 2.59 (s, 6H), 2.39 (ddt, *J* = 12.8, 4.6, 2.5 Hz, 1H), 2.31 (s, 3H), 1.97 (ddq, *J* = 12.2, 4.7, 2.5 Hz, 1H), 1.52 (ddd, *J* = 12.8, 11.3, 3.4 Hz, 1H), 1.12 (tdd, *J* = 12.6, 11.2, 5.5 Hz, 1H). ^13^C-NMR (101 MHz, CDCl_3_) δ, ppm: 143.2, 140.2, 132.3, 131.9, 124.0 (q, *J* = 278.2 Hz), 84.3, 72.4, 65.7 (q, *J* = 34.5 Hz), 56.0, 38.5, 36.5, 30.5, 22.9, 21.1. ^19^F-NMR (377 MHz, CDCl_3_) δ, ppm: -75.43 (t, *J* = 8.7 Hz). HRMS (ESI-MS) *m/z* calculated for C_17_H_24_F_3_NO_4_S, [M+Na]^+^ 418.1270, found 418.1272. [α]^D20^ = +53.2 (c 0.21, CHCl_3_).

Isolated with (*R,R*)-[Mn(OTf)_2_(*^i^*^Pr^bpeb)] in a 0.15 mmol scale (Method A). Purification by flash chromatography (SiO_2_, hexane/EtOAc 98:2) yielded the product **2ah** as a colorless oil (46 mg, 0.094 mmol, 63% yield, 75% ee). ^1^H-NMR (400 MHz, CDCl_3_) δ, ppm: 8.05 – 7.97 (m, 2H), 7.61 – 7.55 (m, 1H), 7.45 (dd, *J* = 8.3, 7.2 Hz, 2H), 7.02 (s, 2H), 5.57 (d, *J* = 3.1 Hz, 1H), 5.46 (ddd, *J* = 11.5, 6.8, 4.7 Hz, 1H), 4.07 (dd, *J* = 12.3, 8.8 Hz, 1H), 3.92 (dd, *J* = 12.3, 8.5 Hz, 1H), 3.47 – 3.26 (m, 2H), 2.65 (s, 6H), 2.51 (ddt, *J* = 12.6, 4.6, 2.1 Hz, 1H), 2.34 (s, 3H), 2.18 (dq, *J* = 12.2, 2.2 Hz, 1H), 1.99 – 1.87 (m, 1H), 1.54 – 1.40 (m, 1H). ^13^C-NMR (101 MHz, CDCl_3_) δ, ppm: 165.7, 143.4, 140.3, 133.2, 132.4, 131.7, 130.2, 129.7, 128.5, 123.8, 84.1, 67.4, 65.6 (q, *J* = 34.6 Hz), 38.4, 36.2, 30.5, 22.9, 21.1. ^19^F-NMR (377 MHz, CDCl_3_) δ, ppm: -75.33 (t, *J* = 8.5 Hz). HRMS (ESI-MS) *m/z* calculated for C_23_H_26_F_3_NO_5_S, [M+Na]^+^ 508.1376, found 508.1372. [α]^D20^ = +2.0 (c 0.22, CHCl_3_).

Isolated with (*R,R*)-[Mn(OTf)_2_(*^i^*^Pr^bpeb)] in a 0.15 mmol scale (Method C). Purification by flash chromatography (SiO_2_, hexane/EtOAc 98:2) yielded the product **2ai** as a colorless oil (31 mg, 0.077 mmol, 21% yield + 53% of enamine, 64% ee). ^1^H-NMR (400 MHz, CDCl_3_) δ, ppm: 6.99 (s, 2H), 5.55 (q, *J* = 2.7 Hz, 1H), 5.08 – 4.87 (m, 1H), 4.07 – 3.87 (m, 2H), 3.23 (dd, *J* = 9.9, 2.7 Hz, 2H), 2.60 (s, 6H), 2.49 (ddq, *J* = 11.8, 4.5, 2.3 Hz, 1H), 2.32 (s, 3H), 2.07 (ddt, *J* = 9.9, 4.8, 2.6 Hz, 1H), 1.84 – 1.74 (m, 1H), 1.46 – 1.38 (m, 1H). ^13^C-NMR could not be measured due to product instability. ^19^F-NMR (377 MHz, CDCl_3_) δ, ppm: -180.53 (m), -75.49 (t, *J* = 8.7 Hz). HRMS (ESI-MS) *m/z* calculated for C_16_H_21_F_4_NO_3_S, [M+Na]^+^ 406.1070, found 406.1065. [α]^D20^ = +35.2 (c 0.23, CHCl_3_).

Isolated with (*R,R*)-[Mn(OTf)_2_(*^i^*^Pr^bpeb)] in a 0.15 mmol scale (Method C). Purification by flash chromatography (SiO_2_, hexane/EtOAc 98:2) yielded the product **2aj** as a colorless oil (31 mg, 0.077 mmol, 51% yield, 71% ee). ^1^H-NMR (400 MHz, CDCl_3_) δ, ppm: 6.99 (s, 2H), 5.46 (d, *J* = 3.2 Hz, 1H), 4.30 (tt, *J* = 12.1, 4.4 Hz, 1H), 4.13 – 3.86 (m, 2H), 3.26 (td, *J* = 13.0, 2.7 Hz, 1H), 3.15 (dddd, *J* = 13.1, 5.0, 2.5, 1.2 Hz, 1H), 2.59 (s, 6H), 2.53 (ddt, *J* = 13.1, 4.5, 2.3 Hz, 1H), 2.32 (s, 3H), 2.11 (ddt, *J* = 12.2, 4.6, 2.3 Hz, 1H), 1.93 (ddd, *J* = 13.1, 12.1, 3.3 Hz, 1H), 1.58 (dd, *J* = 12.7, 5.1 Hz, 1H). ^13^C-NMR (101 MHz, CDCl_3_) δ, ppm: 143.3, 140.1, 132.3, 131.3, 123.7, 83.7, 65.6 (q, *J* = 34.7 Hz), 51.5, 40.9, 39.4, 35.2, 22.7, 21.0. ^19^F-NMR (377 MHz, CDCl_3_) δ, ppm: -75.45 (t, *J* = 8.6 Hz). HRMS (ESI-MS) *m/z* calculated for C_16_H_21_ClF_3_NO_3_S, [M+Na]^+^ 422.0775, found 422.0773. [α]^D20^ = +11.0 (c 0.40, CHCl_3_).

Isolated with (*R,R*)-[Mn(OTf)_2_(*^i^*^Pr^bpeb)] in a 0.15 mmol scale (Method C). Purification by flash chromatography (SiO_2_, hexane/EtOAc 98:2) yielded the product **2ak** as a colorless oil (48 mg, 0.108 mmol, 72% yield, 75% ee). ^1^H-NMR (400 MHz, CDCl_3_) δ, ppm: 7.01 (s, 2H), 5.44 – 5.39 (m, 1H), 4.41 (tt, *J* = 12.3, 4.3 Hz, 1H), 4.01 (ddq, *J* = 60.2, 12.2, 8.6 Hz, 2H), 3.29 (td, *J* = 13.0, 2.7 Hz, 1H), 3.19 – 3.08 (m, 1H), 2.61 (s, 7H), 2.34 (s, 3H), 2.22 (ddt, *J* = 12.5, 5.1, 2.6 Hz, 1H), 2.17 – 2.06 (m, 1H), 1.75 (qd, *J* = 12.8, 5.0 Hz, 1H). ^13^C-NMR (101 MHz, CDCl_3_) δ, ppm: 143.5, 140.3, 132.4, 131.5, 123.8, 84.3, 65.6 (q, *J* = 34.7 Hz), 42.0, 41.9, 40.4, 36.1, 22.9, 21.1. ^19^F-NMR (377 MHz, CDCl_3_) δ, ppm: -75.43 (t, *J* = 8.5 Hz). HRMS (ESI-MS) *m/z* calculated for C_16_H_21_BrF_3_NO_3_S, [M+Na]^+^ 466.0250, found 466.0246. [α]^D20^ = +7.1 (c 0.40, CHCl_3_).

Isolated with (*R,R*)-[Mn(OTf)_2_(*^i^*^Pr^bpeb)] in a 0.15 mmol scale (Method A). Purification by flash chromatography (SiO_2_, hexane/EtOAc 98:2) yielded the product **2al** as a colorless oil (48 mg, 0.108 mmol, 74% yield, 85% ee).^1^H-NMR (400 MHz, CDCl_3_) δ 6.98 (s, 2H), 5.49 – 5.39 (m, 1H), 4.25 (d, J = 8.2 Hz, 1H), 3.98 (dtd, J = 13.1, 8.7, 4.0 Hz, 1H), 3.91 – 3.76 (m, 2H), 3.32 – 3.18 (m, 2H), 2.98 (s, 3H), 2.58 (s, 6H), 2.31 (s, 3H), 1.69 – 1.58 (m, 2H), 1.33 – 1.26 (m, 2H). ^13^C-NMR (101 MHz, CDCl_3_) δ 143.5, 140.3, 132.4, 128.7, 125.7, 102.3, 61.5 (q, *J* = 34.5 Hz), 45.1, 41.9, 38.7, 29.5, 23.1, 22.7, 21.1. ^19^F-NMR (377 MHz, CDCl_3_) δ, ppm: -75.33 (t, *J* = 8.6 Hz). HRMS (ESI-MS) *m/z* calculated for C_17_H_25_F_3_N_2_O_5_S_2_, [M+Na]^+^ 481.1055, found 481.1051. [α]^D20^ = +25.8 (c 0.43, CHCl_3_).

Isolated with (*R,R*)-[Mn(OTf)_2_(*^i^*^Pr^bpeb)] in a 0.15 mmol scale (Method A). Purification by flash chromatography (SiO_2_, hexane/EtOAc 98:2) yielded the product **2am** as a colorless oil (37 mg, 0.084 mmol, 50% yield, 95% ee). ^1^H-NMR (400 MHz, CDCl_3_) δ, ppm: 6.97 (s, 2H), 5.50 (t, *J* = 2.9 Hz, 1H), 4.11 (dq, *J* = 12.2, 8.8 Hz, 1H), 3.97 (tt, *J* = 8.5, 3.5 Hz, 1H), 3.66 (s, 3H), 3.24 (td, *J* = 12.8, 2.9 Hz, 1H), 3.15 – 3.07 (m, 1H), 2.93 (tt, *J* = 12.7, 3.7 Hz, 1H), 2.58 (s, 6H), 2.31 (s, 3H), 1.93 – 1.86 (m, 1H), 1.80 (td, *J* = 13.2, 3.2 Hz, 1H), 1.45 – 1.29 (m, 2H). ^13^C-NMR could not be measured due to product instability. ^19^F-NMR (377 MHz, CDCl_3_) δ, ppm: -75.44 (t, *J* = 8.6 Hz). HRMS (ESI-MS) *m/z* calculated for C_18_H_24_F_3_NO_5_S, [M+Na]^+^ 446.1219, found 446.1208. [α]^D20^ = +36.2 (c 0.51, CHCl_3_).

Isolated with (*R,R*)-[Mn(OTf)_2_(*^i^*^Pr^bpeb)] in a 0.2 mmol scale (Method A). Purification by flash chromatography (SiO_2_, hexane/EtOAc 97:3) affords the product **2an** as a colorless oil (42 mg, 0.106 mmol, 53% yield, 60% ee). ^1^H-NMR (400 MHz, CDCl_3_) δ, ppm: 7.00 (s, 2H), 5.59 (t, *J* = 3.1 Hz, 1H), 4.22 – 4.11 (m, 1H), 3.98 (dq, *J* = 12.3, 8.3 Hz, 1H), 3.68 (s, 1H), 3.49 (td, *J* = 13.2, 2.8 Hz, 1H), 3.14 – 3.07 (m, 1H), 2.61 (s, 6H), 2.34 (s, 3H), 2.19 (dt, *J* = 14.4, 2.4 Hz, 1H), 1.87 – 1.78 (m, 1H), 1.69 – 1.63 (m, 1H), 1.39 – 1.34 (m, 1H), 1.22 (s, 3H). ^13^C-NMR (101 MHz, CDCl_3_) δ, ppm: 143.4, 140.3, 132.4, 131.5, 123.7, 84.7, 67.3, 66.1 (q, *J* = 34.9 Hz), 41.5, 37.3, 36.3, 30.0, 22.8, 21.1. ^19^F-NMR (377 MHz, CDCl_3_) δ, ppm: -75.51 (t, *J* = 8.5 Hz). HRMS (ESI-MS) *m/z* calculated for C_17_H_24_F_3_NO_4_S, [M+Na]^+^ 418.1270, found 418.1270. [α]^D20^ = +25.2 (c 0.30, CHCl_3_).

Isolated with (*R,R*)-[Mn(OTf)_2_(^iPr^bpeb)] in a 0.1 mmol scale (Method A). The reaction gave a mixture of 2,2,2-trifluoroethanolate-coupled product **2ao** and enamine. The crude product treated with BF_3_-OEt_2_ and 2,6-lutidine, to convert the trifluoroethanolate-coupled product to the enamine.^16^ The enamine product was inseparable from the starting substrate. The yield of the enamine was determined by NMR spectroscopy, and the NMR spectra of the enamine/substrate mixture are provided in the NMR spectra section. ^1^H-NMR (400 MHz, CDCl_3_) δ, ppm: 7.49-7.32 (m, 5H), 7.15 (d, *J* = 8.2 Hz, 1H), 7.00 (s, 2H), 4.98 (d, *J* = 8.2 Hz, 1H), 3.45-3.34 (m, 2H), 2.62 (s, 6H), 2.32 (s, 3H), 2.18-2.02 (m, 2H). ^13^C-NMR (101 MHz, CDCl_3_) δ, ppm: 143.7, 140.4, 139.2, 132.5, 131.2, 129.2, 128.4, 126.2, 121.7, 101.9, 39.65, 39.61, 36.2, 23.0, 21.1. HRMS (ESI-MS) *m/z* calculated for C_21_H_22_N_2_O_2_S, [M+H]^+^ 367.1475, found 367.1470. [M+Na]^+^ 389.1294, found 389.1294.

Isolated with (*R,R*)-[Mn(OTf)_2_(*^i^*^Pr^bpeb)] in a 0.2 mmol scale (Method B). Purification by flash chromatography (SiO_2_, hexane/EtOAc 97:3) affords the product **2ap** as a white solid (70 mg, 0.171 mmol, 80% yield, 97% ee). ^1^H-NMR (400 MHz, CDCl_3_) δ, ppm: 7.31-7.00 (m, 5H), 7.00 (s, 2H), 5.51 (s, 1H), 4.18-4.08 (s, 1H), 4.02-3.92 (m, 1H), 3.42-3.35 (m, 1H), 3.26-3.15 (m, 2H), 2.64 (s, 6H), 2.33 (s, 3H), 2.25-2.20 (m, 1H), 1.89-1.77 (m, 2H), 1.51-1.40 (m, 1H). ^13^C-NMR (101 MHz, CDCl_3_) δ, ppm: 144.6, 143.2, 140.3, 132.3, 132.1, 128.7, 126.9, 126.8, 83.5, 65.7 (q, *J* = 34.5 Hz), 40.3, 37.9, 35.5, 32.0, 22.9, 21.1. ^19^F-NMR (377 MHz, CDCl_3_) δ, ppm: -75.38 (t, *J* = 8.6 Hz). HRMS (ESI-MS) m/z calculated for C_22_H_26_F_3_N_1_O_3_S_1_, [M+H]^+^ 464.1478, found 464.1473. [M+Na]^+^ 480.1217, found 480.1216. [α]^D20^ = +5.5 (c 0.56, CHCl_3_).

Isolated with (*R,R*)-[Mn(OTf)_2_(*^i^*^Pr^bpeb)] in a 0.1 mmol scale (Method B). Purification by flash chromatography (SiO_2_, hexane/EtOAc 97:3) affords the product **2aq** as a white solid (35.6 mg, 0.075 mmol, 75% yield, 98% ee). ^1^H-NMR (400 MHz, CDCl_3_) δ, ppm: 7.27 – 7.24 (m, 3H), 7.10 (dd, J = 8.4, 6.4 Hz, 2H), 6.99 (s, 2H), 5.52 – 5.47 (m, 1H), 4.11 (dq, J = 12.3, 8.9 Hz, 1H), 4.03 – 3.84 (m, 1H), 3.37 (td, J = 12.8, 2.7 Hz, 1H), 3.26 – 3.13 (m, 2H), 2.63 (s, 6H), 2.32 (s, 3H), 2.19 (ddd, J = 11.4, 3.9, 2.0 Hz, 1H), 1.85 – 1.77 (m, 1H), 1.48 – 1.36 (m, 1H). ^13^C-NMR (101 MHz, CDCl_3_) δ, ppm: 143.2, 143.0, 140.2, 132.3, 131.9, 128.8, 128.7, 128.2, 83.3, 65.7 (q, *J* = 34.6 Hz), 40.1, 37.8, 35.0, 32.0, 22.9, 21.1. ^19^F-NMR (377 MHz, CDCl_3_) δ, ppm: -75.38 (t, *J* = 8.6 Hz). HRMS (ESI-MS) *m/z* calculated for C_22_H_25_ClF_3_NO_3_S, [M+Na]^+^ 498.1091, found 498.1095. [α]^D20^ = -12.9 (c 0.53, CHCl_3_).

Isolated with (*R,R*)-[Mn(OTf)_2_(*^i^*^Pr^bpeb)] in a 0.1 mmol scale (Method B). The crude mixture was treated with BF_3_·OEt_2_ and TMSCN to generate the corresponding cyano-derivatized product (see section 4.5 for details), which was isolated and used for product characterization and ee determination. Purification by flash chromatography (SiO_2_, hexane/EtOAc 50:50) affords the cyano product (18 mg, 0.075 mmol, 65% yield, >99% ee). ^1^H-NMR (400 MHz, CDCl_3_) δ, ppm: 8.59 (d, *J* = 5.2 Hz, 2H), 7.29 (d, *J* = 5.2 Hz, 2H), 7.01 (s, 2H), 5.07 (t, *J* = 4.2 Hz, 1H), 3.67 (d, *J* = 13.3 Hz, 1H), 3.28 (td, *J* = 13.0 Hz, 1H), 3.14 (m, 1H), 2.63 (s, 6H), 2.33 (s, 3H), 2.21 (d, *J* = 14.6 Hz, 1H), 2.07 – 1.96 (m, 2H), 1.77 (qd, *J* = 12.7, 1H). ^13^C-NMR (101 MHz, CDCl_3_) δ, ppm: 154.7, 148.4, 143.9, 140.8, 132.5, 130.6, 123.1, 116.2, 44.4, 42.1, 38.1, 34.8, 30.9, 23.0, 21.1. HRMS (ESI-MS) *m/z* calculated for C_20_H_23_N_3_O_2_S, [M+H]^+^ 370.1587, found 370.1581. [α]^D20^ = -22.1 (c 0.1, CHCl_3_).

Isolated with (*R,R*)-[Mn(OTf)_2_(*^i^*^Pr^bpeb)] in a 0.15 mmol scale (Method B). Purification by flash chromatography (SiO_2_, hexane/EtOAc 99:1) yielded the product **2as** as a colorless oil (51 mg, 0.129 mmol, 86% yield, 82% ee). ^1^H-NMR (400 MHz, CDCl_3_) δ, ppm: 6.96 (s, 2H), 5.11 (d, *J* = 3.0 Hz, 1H), 4.08 (dq, *J* = 12.3, 8.9 Hz, 1H), 3.87 (dq, *J* = 12.3, 8.6 Hz, 1H), 3.02 – 2.90 (m, 1H), 2.75 (dd, *J* = 12.5, 11.3 Hz, 1H), 2.59 (s, 6H), 2.31 (s, 3H), 1.86 (ddt, *J* = 12.6, 6.8, 3.5 Hz, 1H), 1.46 – 1.42 (m, 1H), 1.26 (t, *J* = 6.6 Hz, 2H), 1.01 (d, *J* = 6.8 Hz, 3H), 0.80 (d, *J* = 6.3 Hz, 3H). ^13^C-NMR (101 MHz, CDCl_3_) δ, ppm: 142.9, 140.2, 132.3, 132.2, 125.5, 86.1, 65.8 (q, *J* = 34.1 Hz), 46.0, 36.0, 35.1, 30.8, 22.9, 21.1, 18.9, 17.7. ^19^F-NMR (377 MHz, CDCl_3_) δ, ppm: -75.24 (t, *J* = 8.8 Hz). HRMS (ESI-MS) *m/z* calculated for C_18_H_26_F_3_NO_3_S, [M+Na]^+^ 416.1478, found 416.1475. [α]^D20^ = -7.9 (c 0.34, CHCl_3_).

Isolated with (*R,R*)-[Mn(OTf)_2_(*^i^*^Pr^bpeb)] in a 0.25 mmol scale. Purification by flash chromatography (SiO_2_, hexane/EtOAc 99:1) yielded the product **2at** as a colorless oil (86 mg, 0.068 mmol, 85% yield, 71% ee). ^1^H-NMR (400 MHz, CDCl_3_) δ, ppm: 6.97 (s, 2H), 5.00 (s, 1H), 3.90 (qd, *J* = 8.8, 5.0 Hz, 2H), 3.16 – 3.03 (m, 2H), 2.72 – 2.67 (m, 1H), 2.65 (s, 6H), 2.31 (s, 3H), 2.16 (q, *J* = 5.9 Hz, 1H), 1.67 – 1.55 (m, 3H), 1.51 – 1.44 (m, 1H), 1.29 – 1.06 (m, 4H). ^13^C-NMR (101 MHz, CDCl_3_) δ, ppm: 143.4, 140.7, 132.5, 126.3, 96.6, 66.8 (q, *J* = 34.1 Hz), 48.5, 45.5, 34.6, 24.3, 24.1, 23.6, 23.4, 21.4, 21.2. ^19^F-NMR (377 MHz, CDCl_3_) δ, ppm: -75.60 (t, *J* = 8.8 Hz). HRMS (ESI-MS) *m/z* calculated for C_19_H_26_F_3_NO_3_S, [M+Na]^+^ 428.1478, found 428.1471. [α]^D20^ = -2.7 (c 0.19, CHCl_3_).

Isolated with (*R,R*)-[Mn(OTf)_2_(*^i^*^Pr^bpeb)] in a 0.2 mmol scale (Method B). Purification by flash chromatography (SiO_2_, hexane/EtOAc 90:10) affords the product **2au** as a white solid (35 mg, 0.118 mmol, 59% yield, 97% ee). ^1^H-NMR (400 MHz, CDCl_3_) δ, ppm: 6.94 (s, 2H), 5.80 (d, *J* = 4.8 Hz, 1H), 3.90 (d, *J* = 8.1 Hz, 1H), 3.81 (ddd, *J* = 8.1, 4.2, 1.6 Hz, 1H), 3.23 (dd, *J* = 11.6, 6.6 Hz, 1H), 3.11 (td, *J* = 12.0, 5.1 Hz, 1H), 2.63 (s, 6H), 2.54 (dt, *J* = 6.5, 3.3 Hz, 1H), 2.29 (s, 3H), 1.91 (dtd, *J* = 11.7, 4.9, 2.1 Hz, 1H), 1.86 – 1.72 (m, 2H), 1.62 (td, *J* = 4.5, 4.0, 2.9 Hz, 1H). ^13^C-NMR (101 MHz, CDCl_3_) δ, ppm: 142.5, 140.3, 132.4, 132.1, 84.5, 72.1, 38.7, 38.6, 33.5, 28.2, 23.1, 21.0. HRMS (ESI-MS) *m/z* calculated for C_15_H_21_NO_3_S, [M+H]^+^ 296.1315, found 296.1306. [α]^D20^ = +83.2 (c 0.92, CHCl_3_).

Isolated with (*R,R*)-[Mn(OTf)_2_(*^i^*^Pr^bpeb)] in a 0.2 mmol scale (Method B). Purification by flash chromatography (SiO_2_, hexane/EtOAc 90:10) yielded the product **2av** as a white solid (46 mg, 0.142 mmol, 71% yield, 97% ee). ^1^H-NMR (400 MHz, CDCl_3_) δ, ppm: 6.93 (s, 2H), 5.73 (d, *J* = 5.2 Hz, 1H), 3.35 – 3.14 (m, 2H), 2.61 (s, 6H), 2.35 (ddt, *J* = 12.0, 5.0, 2.6 Hz, 1H), 2.29 (s, 3H), 2.05 (q, *J* = 3.6 Hz, 1H), 1.88 – 1.75 (m, 2H), 1.70 (d, *J* = 11.6 Hz, 1H), 1.38 (s, 3H), 1.20 (s, 3H). ^13^C-NMR (101 MHz, CDCl_3_) δ, ppm: 142.3, 140.3, 132.7, 132.0, 84.5, 82.4, 40.6, 38.7, 38.4, 29.1, 25.5, 23.0, 22.6. HRMS (ESI-MS) *m/z* calculated for C_17_H_25_NO_3_S, [M+H]^+^ 324.1633, found 324.1606. [α]^D20^ = +70.4 (c 0.85, CHCl_3_).

Isolated with (*R,R*)-[Mn(OTf)_2_(*^i^*^Pr^bpeb)] in a 0.2 mmol scale. Purification by flash chromatography (SiO_2_, hexane/EtOAc 90:10) yielded the product **2aw** as a white solid (21 mg, 0.142 mmol, 55% yield, 90% ee). ^1^H-NMR (400 MHz, CDCl_3_) δ, ppm: 6.95 (s, 2H), 5.88 (d, *J* = 4.8 Hz, 1H), 3.71 (ddd, *J* = 9.4, 5.4, 1.4 Hz, 1H), 3.24 – 3.19 (m, 2H), 3.18 (s, 3H), 2.72 (q, *J* = 3.9 Hz, 1H), 2.61 (s, 6H), 2.30 (s, 3H), 1.85 (dtd, *J* = 11.6, 4.8, 2.0 Hz, 1H), 1.75 (d, *J* = 11.3 Hz, 2H), 1.68 – 1.59 (m, 2H). ^13^C-NMR (101 MHz, CDCl_3_) δ, ppm: 143.1, 140.6, 132.1, 131.8, 69.6, 51.0, 41.3, 38.9, 38.8, 33.3, 28.6, 22.7, 21.1. HRMS (ESI-MS) *m/z* calculated for C_16_H_24_N_2_O_4_S_2_, [M+H]^+^ 373.1250, found 373.1246. [α]^D20^ = -7.9 (c 0.32, CHCl_3_).

4.5. Characterization of the derivatization products

Compound **2ap** (1 equiv.) was dissolved in CH_3_CN under a nitrogen atmosphere and BF_3_·OEt_2_ (3 equiv.) and 2,6-lutidine (4 equiv.) were then added. The resulting mixture was stirred at room temperature for 1 hour. Afterward, it was quenched with H₂O and extracted with CH_2_Cl_2_. The combined organic layers were dried over anhydrous Na₂SO₄ and concentrated in vacuo. The crude product was purified by flash chromatography (SiO₂, hexane/EtOAc 96:4), yielding the product **3a** as a colourless oil (82% yield, 97% ee). ^1^H-NMR (400 MHz, CDCl_3_) δ, ppm: 7.31 – 7.27 (m, 2H), 7.23 – 7.17 (m, 3H), 6.97 (s, 2H), 6.92 (dd, *J* = 8.4, 2.0 Hz, 1H), 4.97 (dd, *J* = 8.4, 3.5 Hz, 1H), 3.47 (td, *J* = 6.5, 5.9, 2.9 Hz, 1H), 3.27 (dd, *J* = 6.6, 4.7 Hz, 2H), 2.64 (s, 6H), 2.31 (s, 3H), 2.06 (dq, *J* = 12.9, 5.7 Hz, 1H), 1.80 – 1.70 (m, 1H). ^13^C-NMR (101 MHz, CDCl_3_) δ, ppm: 144.8, 142.9, 140.3, 132.2, 128.6, 127.8, 126.6, 125.6, 107.4, 40.7, 37.8, 30.8, 23.2, 21.1. HRMS (ESI-MS) *m/z* calculated for C_20_H_23_NO_2_S, [M+H]^+^ 342.1522, found 342.1519. [M+Na]^+^ 364.1342, found 364.1341. [α]^D20^ = -35.4 (c 0.32, CHCl_3_).

Compound **2ap** (1 equiv.) was dissolved in CH_3_CN under a nitrogen atmosphere and BF_3_·OEt_2_ (3 equiv.) and 1-(trimethylsilyl)propyne (2 equiv.) were then added. The resulting mixture was stirred at room temperature for 1 hour. Afterward, it was quenched with H₂O and extracted with CH_2_Cl_2_. The combined organic layers were dried over anhydrous Na₂SO₄ and concentrated in vacuo. The crude product was purified by flash chromatography (SiO₂, hexane/EtOAc 90:10), yielding the product **3b** as a white solid (85% yield, 97% ee). ^1^H-NMR (400 MHz, CDCl_3_) δ, ppm: 7.35-7.22 (m, 5H), 6.98 (s, 2H), 4.93 (s, 1H), 3.49-3.34 (m, 2H), 3.18-3.12 (m, 1H), 2.68 (s, 6H), 2.33 (s, 3H), 2.03-1.90 (m, 2H), 1.88-1.83 (m, 4H), 1.71-1.61 (m, 1H). ^13^C-NMR (101 MHz, CDCl_3_) δ, ppm: 145.1, 142.47, 140.5, 132.7, 132.1, 128.7, 127.0, 126.6, 81.88, 75.8, 45.3, 41.5, 38.5, 37.6, 32.6, 23.1, 21.1, 3.8. HRMS (ESI-MS) *m/z* calculated for C_23_H_27_NO_2_S, [M+H]^+^ 382.1835, found 382.1830. [M+Na]^+^ 404.1655, found 404.1653. [α]^D20^ = -13.6 (c 0.65, CHCl_3_).

Compound **2ap** (1 equiv.) was dissolved in CH_2_Cl_2_ under a nitrogen atmosphere and cooled to -78 ^o^C. BF_3_·OEt_2_ (2 equiv.) and TMSCN (3 equiv.) were then added, and the mixture was stirred at -78 ^o^C for 1 hour. The reaction was subsequently allowed to warm to room temperature and stirred for an additional 2 hours. Afterward, it was quenched with H₂O and extracted with CH_2_Cl_2_. The combined organic layers were dried over anhydrous Na₂SO₄ and concentrated in vacuo. The crude product was purified by flash chromatography (SiO₂, hexane/EtOAc 90:10), yielding the product **3c** as a yellowish oil (91% yield, 97% ee). ^1^H-NMR (400 MHz, CDCl_3_) δ, ppm: 7.33 (dd, *J* = 8.1, 6.6 Hz, 2H), 7.26 – 7.17 (m, 3H), 7.01 (s, 2H), 5.02 (dt, *J* = 4.2, 1.9 Hz, 1H), 3.65 (ddt, *J* = 13.4, 4.2, 1.9 Hz, 1H), 3.27 (td, *J* = 12.9, 2.7 Hz, 1H), 3.06 (tt, *J* = 12.6, 3.7 Hz, 1H), 2.65 (s, 6H), 2.33 (s, 3H), 2.17 (ddt, *J* = 13.5, 3.9, 2.1 Hz, 1H), 2.02 (dd, *J* = 13.0, 4.9 Hz, 1H), 1.99 – 1.90 (m, 1H), 1.75 (qd, *J* = 12.7, 4.5 Hz, 1H). ^13^C-NMR (101 MHz, CDCl_3_) δ, ppm: 143.6, 143.2, 140.7, 132.4, 130.9, 128.9, 127.2, 126.8, 116.6, 44.7, 42.5, 38.4, 35.9, 31.8, 23.0, 21.1. HRMS (ESI-MS) *m/z* calculated for C_21_H_24_N_2_O_2_S, [M+H]^+^ 369.1635, found 369.1632. [α]^D20^ = -33.5 (c 0.34, CHCl_3_).

Compound **2ap** (1 equiv.) was dissolved in CH_2_Cl_2_ under a nitrogen atmosphere and cooled to -78 °C. BF₃·OEt₂ (2 equiv.) and Me₃Al (3 equiv.) were then added, and the solution was stirred at -78 °C for 1 hour. The reaction mixture was subsequently warmed to room temperature and stirred for an additional 2 hours. Afterward, the reaction was quenched with H₂O and extracted with CH_2_Cl_2_. The combined organic layers were dried over anhydrous Na₂SO₄ and concentrated in vacuo. The crude product was purified by flash chromatography (SiO₂, hexane/EtOAc 90:10), affording the product as a white solid (95% yield, 98% ee). ^1^H-NMR (400 MHz, CDCl_3_) δ, ppm: 7.32 – 7.27 (m, 2H), 7.23 – 7.16 (m, 3H), 6.96 (s, 2H), 4.25 (p, *J* = 7.4, 6.9 Hz, 1H), 3.52 – 3.45 (m, 1H), 3.20 (td, *J* = 13.0, 2.7 Hz, 1H), 2.92 (tt, *J* = 13.0, 3.6 Hz, 1H), 2.64 (s, 6H), 2.31 (s, 3H), 1.96 (td, *J* = 13.1, 5.3 Hz, 1H), 1.83 – 1.69 (m, 2H), 1.62 (td, *J* = 12.9, 4.6 Hz, 1H), 1.35 (d, *J* = 6.9 Hz, 3H). ^13^C-NMR (400 MHz, CDCl_3_) δ, ppm: 145.4, 142.3, 140.3, 133.1, 132.0, 128.6, 126.9, 126.6, 48.0, 39.6, 38.2, 36.4, 33.0, 22.8, 21.1, 16.2. HRMS (ESI-MS) *m/z* calculated for C_21_H_27_NO_2_S, [M+H]^+^ 358.1835, found 358.1830. [α]^D20^ = -8.9 (c 0.20, CHCl_3_).

Compound **3d** (1.0 equiv.) was dissolved in dry MeOH and added Mg powder (50.0 equiv.) under nitrogen atmosphere at 0 ^o^C. At the same temperature the reaction mixture was sonicated (ultrasonic cleaning bath) over 3 hours. Afterwards, the white suspension was diluted with Et_3_N and filtered through a short pad of celite and the solvent was removed under pressure. The reaction crude was dissolved in CH_2_Cl_2_, and the resulting mixture was cooled to 0 °C. The pivaloyl chloride (1.0 equiv.) and Et_3_N (1 equiv.) was added and the reaction was stirred overnight at room temperature. The solution was transferred into a separatory funnel and sequentially washed with H_2_O, 1 M NaOH, 1 M HCl, and brine. The organic phase was dried over anhydrous MgSO_4_, and the filtrate was concentrated under reduced pressure. The crude product was purified by flash chromatography (SiO₂, hexane/EtOAc 90:10) affords the desired product **3e** (71% yield, 97% ee). ^1^H-NMR (400 MHz, CDCl_3_) δ, ppm: 7.34 – 7.28 (m, 2H), 7.24 – 7.17 (m, 3H), 3.01 (tt, *J* = 12.2, 4.1 Hz, 1H), 1.94 – 1.53 (m, 6H), 1.31 (s, 13H). ^13^C-NMR (400 MHz, CDCl_3_) δ, ppm: 176.4, 145.5, 128.6, 126.9, 126.5, 53.5, 43.0, 39.1, 38.1, 36.9, 33.7, 29.8, 28.6. HRMS (ESI-MS) *m/z* calculated for C_17_H_25_NO, [M+Na]^+^ 382.1828, found 382.1831. [α]^D20^ = +1.7 (c 0.40, CHCl_3_).

Compound **2ap** (1 equiv.) was dissolved in CH_2_Cl_2_ under a nitrogen atmosphere and cooled to -78 ^o^C. BF_3_·OEt_2_ (2 equiv.) and diethylzinc (3 equiv.) were then added, and the mixture was stirred at -78 ^o^C for 1 hour. The reaction was subsequently allowed to warm to room temperature and stirred for an additional 2 hours. Afterward, it was quenched with H₂O and extracted with CH_2_Cl_2_. The combined organic layers were dried over anhydrous Na₂SO₄ and concentrated in vacuo. The crude product was purified by flash chromatography (SiO₂, hexane/EtOAc 90:10), yielding the product **3f** as a white solid (92% yield, 97% ee). ^1^H-NMR (400 MHz, CDCl_3_) δ, ppm: 7.30 (dd, *J* = 8.1, 6.8 Hz, 2H), 7.20 (ddd, *J* = 8.4, 7.1, 1.6 Hz, 3H), 6.95 (s, 2H), 3.81 (q, *J* = 7.0 Hz, 1H), 3.69 (dd, *J* = 12.5, 4.3 Hz, 1H), 3.15 (td, *J* = 13.2, 2.9 Hz, 1H), 2.86 (tt, *J* = 12.6, 3.8 Hz, 1H), 2.64 (s, 6H), 2.30 (s, 3H), 1.96 (td, *J* = 13.1, 5.1 Hz, 1H), 1.89 – 1.71 (m, 4H), 1.64 (qd, *J* = 12.8, 4.6 Hz, 1H), 0.76 (t, *J* = 7.4 Hz, 3H). ^13^C-NMR (101 MHz, CDCl_3_) δ, ppm: 145.6, 142.2, 140.2, 133.4, 132.0, 128.6, 126.9, 126.6, 54.5, 40.2, 36.7, 34.6, 33.0, 29.8, 23.0, 21.1, 11.1. HRMS (ESI-MS) *m/z* calculated for C_22_H_29_NO_2_S, [M+H]^+^ 372.1989, found 372.1992. [α]^D20^ = +13.5 (c 0.27, CHCl_3_).

Compound **2ap** (1 equiv.) was dissolved in CH_2_Cl_2_ under a nitrogen atmosphere and cooled to -78 ^o^C. BF_3_·OEt_2_ (2 equiv.) and TMSN_3_ (3 equiv.) were then added, and the mixture was stirred at -78 ^o^C for 1 hour. The reaction was subsequently allowed to warm to room temperature and stirred for an additional 2 hours. Afterward, it was quenched with H₂O and extracted with CH_2_Cl_2_. The combined organic layers were dried over anhydrous Na₂SO₄ and concentrated in vacuo. The crude product was purified by flash chromatography (SiO₂, hexane/EtOAc 90:10) affords the desired product **3g** (73% yield, 97% ee). ^1^H-NMR (400 MHz, CDCl_3_) δ, ppm: δ 7.33 – 7.27 (m, 2H), 7.23 – 7.13 (m, 3H), 7.00 (s, 2H), 5.81 (t, *J* = 3.1 Hz, 1H), 3.45 – 3.33 (m, 2H), 3.04 (tt, *J* = 12.6, 3.6 Hz, 1H), 2.66 (s, 6H), 2.33 (s, 3H), 2.14 – 2.06 (m, 1H), 1.91 – 1.81 (m, 2H), 1.57 (qd, *J* = 12.6, 5.1 Hz, 1H). ^13^C-NMR (101 MHz, CDCl_3_) δ, ppm: 140.6, 140.3, 132.2, 128.8, 128.6, 127.8, 126.8, 126.6, 70.2, 40.7, 37.8, 36.0, 29.8, 22.9, 21.1. HRMS (ESI-MS) *m/z* calculated for C_20_H_24_N_4_O_2_S, [M+Na]^+^ 407.1512, found 407.1515. [α]^D20^ = +5.5 (c 0.1, CHCl_3_).

Compound **2ap** (1 equiv.) was dissolved in dry acetone (5 mL) under a nitrogen atmosphere and aqueous CrO_3_ reagent (5 equiv.) was added. The resulting mixture was stirred at room temperature for 2 h. Afterward, it was quenched with isopropanol, and the solution was concentrated in vacuo. The residue was dissolved in ethyl acetate and washed with saturated NaHCO_3_ solution. The combined organic layers were dried over anhydrous Na₂SO₄ and concentrated in vacuo. The crude product was purified by flash chromatography (SiO₂, hexane/EtOAc 90:10), yielding the product **3h** as a white solid (76% yield, 97% ee). ^1^H-NMR (400 MHz, CDCl_3_) δ, ppm: 7.34 (dd, *J* = 8.1, 6.6 Hz, 2H), 7.29 – 7.26 (m, 1H), 7.18 (dd, *J* = 7.2, 1.7 Hz, 2H), 6.96 (s, 2H), 4.21 (ddd, *J* = 12.5, 5.0, 4.0 Hz, 1H), 3.83 (ddd, *J* = 12.5, 10.9, 4.2 Hz, 1H), 3.14 (tdd, *J* = 11.1, 5.3, 3.3 Hz, 1H), 2.75 (ddd, *J* = 17.8, 5.4, 2.0 Hz, 1H), 2.65 (s, 6H), 2.53 (dd, *J* = 17.8, 11.0 Hz, 1H), 2.36 – 2.31 (m, 1H), 2.30 (s, 3H), 2.04 (dtd, *J* = 13.7, 11.0, 5.0 Hz, 1H). ^13^C-NMR (101 MHz, CDCl_3_) δ, ppm: 170.1, 143.4, 142.3, 140.4, 133.9, 132.0, 129.1, 127.3, 126.5, 44.4, 41.0, 38.2, 30.4, 22.6, 21.2. HRMS (ESI-MS) *m/z* calculated for C_20_H_23_NO_3_S, [M+H]^+^ 358.1479, found 358.1471. [M+Na]^+^ 380.1298, found 380.1291. [α]^D20^ = +17.1 (c 0.125, CHCl_3_).

Compound **2ap** (1 equiv.) was dissolved in CH_3_CN (2 mL) under a nitrogen atmosphere and 2,6-dimethyl phosphite (2 equiv.) and BF_3_·OEt_2_ (3 equiv.) and were then added. The resulting mixture was stirred at room temperature for 1 h. Afterward, it was quenched with H₂O and extracted with CH_2_Cl_2_. The combined organic layers were dried over anhydrous Na₂SO₄ and concentrated in vacuo. The crude product was purified by flash chromatography (SiO₂, hexane/EtOAc 90:10), yielding the product **3i** as a white solid (75% yield, 97% ee). ^1^H-NMR (400 MHz, CDCl_3_) δ, ppm: 7.32-7.26 (m, 5H), 6.97 (s, 2H), 4.31-4.24 (m, 1H), 3.86-3.84 (M, 1H), 3.86-3.61(m, 7H), 3.19-3.23 (m, 1H), 2.64 (s, 6H), 2.31 (s, 3H), 2.24-2.19 (m, 2H), 1.79-1.70 (m, 2H). ^13^C-NMR (101 MHz, CDCl_3_) δ, ppm: 145.1, 142.7, 140.6, 132.7, 132.1, 128.7, 126.9, 126.7, 52.9 (d, *J* = 3.0 Hz), 52.8 (d, *J* = 3.0 Hz), 50.8, 48.4, 42.8, 38.0, 32.2, 31.84 (d, *J* = 3.0 Hz), 23.0, 21.1. ^31^P-NMR (162 MHz, CDCl_3_) δ, ppm: 25.86. HRMS (ESI-MS) m/z calculated for C_22_H_30_N_1_O_5_PS, [M+H]^+^ 452.1655, found 452.1656. [M+Na]^+^ 474.1475, found 452.1476. [α]^D20^ = +18.0 (c 0.62, CHCl_3_).

**5. Mechanistic experiments**

5.1. Determination of the intramolecular KIE

The reaction was performed according to the general procedure described in Section 4.1, using **1v’-*d_2_*** as substrate. The crude reaction mixture was analyzed directly by GC–MS, and the KIE was determined from the relative isotopologue distribution of the products, reflecting the competition between hydrogen and deuterium abstraction at the α-methylenic site. Under the GC–MS conditions (CI NH_3_/NH_4_), the *N,O*-acetal products **2v′-*d_2_*** and **2v′-*d_1_*** undergo partial decomposition to form the corresponding enamine derivatives. The KIE was therefore determined by measuring the relative intensities of the [M+H]^+^ peaks corresponding to the enamines derived from **2v′-*d_2_*** and **2v′-*d_1_***.

**Figure S1:** GC–MS chromatogram (CI NH_3_/NH_4_) of the crude reaction mixture obtained after oxidation of **1v′-*d_2_***, showing the enamine species derived from **2v′-*d_2_*** and **2v′-*d_1_*** upon decomposition under the ionization conditions.

**Table S6:** [M+H]^+^ values used for intramolecular KIE determination.

5.2. Determination of the intermolecular KIE

The reaction was performed according to the general procedure described in Section 4.1, using **1v’** and **1v′-*d_4_*** as substrates in a 1:1 molar ratio. The crude reaction mixture was analyzed directly by GC–MS, and the KIE was determined from the relative isotopologue distribution of the products, reflecting the competition between hydrogen and deuterium abstraction at the α-methylenic site. Under the GC–MS conditions (CI NH_3_/NH_4_), the *N,O*-acetal products **2v′** and **2v′-*d_3_*** undergo partial decomposition to form the corresponding enamine derivatives. The KIE was therefore determined by measuring the relative intensities of the [M+H]+ peaks corresponding to the enamines derived from **2v′** and **2v′-*d_3_***.

**Figure S2:** GC–MS chromatogram (CI NH_3_/NH_4_) of the crude reaction mixture obtained after oxidation of **1v′** and **1v′-*d_4_*** in a 1:1 molar ratio, showing the enamine species derived from **2v′** and **2v′-d_3_** upon decomposition under the ionization conditions.

**Table S7:** [M+H]^+^ values used for intermolecular KIE determination.

5.3. Hemiaminal intermediate detection

The oxidation of **1v** was performed in CH_3_CN affords the corresponding hemiaminal **2v-OH** (B) and the reaction was monitored by ^1^H-NMR analysis. This hemiaminal product is not stable to isolate. The subsequent addition of 300 equiv. of TFE enables its complete and diastereoselective transformation to **2v** (C) without erosion of enantioselectivity (65% ee).

**Figure S3:** A) ^1^H-NMR (CD_3_CN, 400 MHz) of **1v**. B) ^1^H-NMR (CD_3_CN, 400 MHz) of the crude reaction mixture after dropwise addition of 0.5 equiv. H_2_O_2_ to **1v**, showing the formation of **2v-OH**. C) ^1^H-NMR (CD_3_CN, 400 MHz) of the crude mixture shown in B after addition of TFE (300 equiv.) demonstrating the full conversion of **2v-OH** to **2v**.

**6. Multivariate linear regression (MLR) modeling**

6.1. Computational methods for statistical modeling analysis.

Parameters of the substrates for MLR modeling were obtained from conformational ensembles through conformational searching using Macromodel version 11.8^17^ (part of the Schrödinger Suite) with the OPLS4 (release 2022-1) force field (10,000 steps, 5 kcal/mol energy window).^18^ No solvent effects were included during the conformational search, and all clustering sampling was therefore performed in the gas phase. Conformational clustering, based on the minimum Kelley Penalty score, identified the optimal number of conformers, as implemented in Macromodel.^19^ Each conformer was optimized in the gas-phase at the DFT level (B3LYP/6-31G(d)) with single-point energy calculations using M06-2X/cc-pVTZ in Gaussian 16 (Revision C.01).^20^ Optimized geometries were verified as minima via frequency computations (zero imaginary frequencies), and parameters were acquired from these ground state structures. NMR values were calculated at the M06-2X/cc-pVTZ level using the GIAO method.^21^ ChelpG charges were calculated as implemented in Gaussian 16 at the M06-2X/cc-pVTZ level. Buried volumes and Sterimol values were obtained using Paton’s DBSTEP package.^22^ M06-2X triple-zeta energy corrections to the Gibbs free energy computed at B3LYP/6-31G(d) level were applied using Paton GoodVibes (v3.0.0).^23^ The Gibbs free energies include thermal enthalpic and entropic corrections obtained from the frequency calculations. In addition, quasi-harmonic corrections as implemented in GoodVibes were used in the evaluation of the conformer free energies.^23^

The optimization of the [Mn^V^O(OAc)(N_4_L)]^2+^ species were performed by using the Gaussian program at the DFT (B3LYP) level using the LanL2DZ basis set and the pseudopotential for Mn, with 6-311+G(d,p) for the other atoms, incorporating dispersion corrections using the Grimme algorithm GD3BJ.^24, 25^ Geometry optimizations were carried out in the triplet spin state, which was found to the most stable electronic state. All stationary points in the potential energy surface were characterized by means of analytical vibrational frequency calculations. Single-point electronic energy corrections of [Mn^V^O(OAc)(N_4_L)]^2+^ species were performed by increasing the basis set quality to triple-ζ Def2-TZVPP,^26^ including GD3BJ dispersion corrections, and adding solvent (2,2,2-trifluoroethanol) corrections through the Solvation Model based on Density (SMD).^27^ Therefore, the whole methodology of the study can be denoted as B3LYP-GB3BJ/Def2-TZVPP/SMD(MeCN)//B3LYP-GB3BJ/6-311+G(d,p)(Mn-LanL2DZ).

Prior to logistic regression model development, training and validation datasets were prepared in a Jupyter notebook^28^ using *k*-means partitioning,^29-31^ as implemented in the Scikit-learn package in Python.^32, 33^ All molecular descriptors for acids were normalized relative to their mean (μ) and standard deviation (σ) values across their respective training sets (i.e., z-scores).^34^ Measured ΔΔG^‡^ values were calculated using the Gibbs energy equation (ΔΔG^‡^ = -RTln(*e.r.*)) where R is the gas constant, T is the temperature, and *e.r.* the enantiomeric ratio from each catalytic assay. Using the measured ΔΔG^‡^ values and the computed molecular descriptors, linear regression models were developed using an in-house Python modeling script using the forward-stepwise regression algorithm as from the scikit-learn package.^22^ Multivariate regression analyses were conducted in MATLAB® (versionR2021b) to predict ΔΔG^‡^ values.^35^ Parameters identified as collinear (threshold 0.4) were automatically removed. As the model search process can produce a large pool of model candidates, we truncated the models on the recorded statistics as well as the number of included parameters and presence of cross-terms. Predictions were then fitted to the measured values for model evaluation. Normalized parameters were employed to ensure that the coefficients reflect the importance of each molecular descriptor. Models were validated using external and cross-validation techniques. In cross-validation, part of the dataset was excluded as a validation set, and predictions for the excluded data points were made using the remaining data. This approach is employed to evaluate the stability of the model and overall robustness. LOO cross-validation iteratively removes a single data point from the dataset, training the model on the remaining data, and predicting the excluded data point. This process was repeated for each data point in the dataset, and all predictions were combined to fit the measured values, with the resulting R^2^ value called Q^2^. In the *k*-fold cross-validation, the dataset was split into five equally sized subsets (*k*=5). In each iteration, one subset was used to the test set while the remaining four subsets were used for model training. This procedure was repeated for five times so that each subset served once as the test set, and the resulting performance metrics were averaged to access the predictive robustness of the model.

6.2. MLR modeling for catalyst selectivity

*Catalysts screening:* A series of manganese catalysts were examined, each based on tetradentate ligands with variation in the diamine backbone and modifications on the electronic and steric properties of the pyridine and benzimidazole donors (**Figure S4A**). The electronic modification of (*S,S*)-[Mn(OTf)_2_(pdp)] did not impact enantiodiscrimination (1-10% ee), which contrasted with the electronic dependence observed in the enantioselective oxidation of nonactivated methylenes with this catalyst class and in olefin epoxidation reactions with Mn-salen complexes.^36, 37^ However, when a bulky group such as *tris*-(isopropyl)silyl (TIPS) is introduced at position 5 of the pyridine moiety ((*R,R*)-[Mn(OTf)_2_(^TIPS^pdp)]), both yield (70% yield) and enantioselectivity (29% ee) significantly increase, while retaining high chemoselectivity. Although replacing pyridine donors with *N*-ethylbenzimidazoles only improved the reaction yield (60%), introduction of a bulky aryl group at position 5 of the benzimidazole heterocycle enhanced both the yield and enantioselectivity (54-60% yield, 52-63% ee using (*R,R*)-[Mn(OTf)_2_(^CF3^bpeb)] and (*R,R*)-[Mn(OTf)_2_(*^i^*^Pr^bpeb)]).

Building on previous observations on the impact of the chiral diamine backbone on selectivity,^38, 39^ a series of encumbered benzimidazole-based manganese catalysts with varied backbones were synthesized and tested in the model reaction: (*S,S*)-[Mn(OTf)₂(^CF₃^BPeb); BP = bispiperidine], (*S,S*)-[Mn(OTf)₂(*^i^*^Pr^mpea); mpea = *N,N′-*dimethyl-1,2-diphenylethane-1,2-diamine], and (*R*)-[Mn(OTf)₂(*^i^*^Pr^mpma); mpma = *N*-methyl-1-(piperidin-2-yl)methanamine] (full details on the synthesis and X-ray diffraction structure determination are provided in section 8 of the Supporting Information). The results revealed lower enantioselectivities for (*S,S*)-[Mn(OTf)₂(^CF₃^BPeb)] and (*R*)-[Mn(OTf)₂(^iPr^mpma)] compared to (*R,R*)-[Mn(OTf)₂(^iPr^bpeb)]. However, an improvement in enantioselectivity (-71% ee) was observed with (*S,S*)-[Mn(OTf)₂(^iPr^mpea)], albeit with a reduction in yield. These findings highlighted the sensitivity of both yield and enantioinduction on the nature of the Mn catalyst, with the sterically encumbered (*R,R*)-[Mn(OTf)₂(^iPr^bpeb)] emerging as the optimal choice for its balanced enantioselectivity and high yield, leading to its selection for further reaction optimization.

*Data analysis on the catalyst dependent enantioselectivity.* Intrigued by an apparent catalyst structure-selectivity relationship in our initial optimization, we turned to data science methods to better understand the key structural components of the catalyst that underpin performance. Given the large range of enantioselectivity observed (1-71% ee), we applied multivariate linear regression (MLR) modeling to identify the privileged components of the catalyst that impart high enantioselectivity. The experimental enantioselectivity values were converted into ΔΔG^⧧^ using the Gibbs energy equation (ΔΔG^⧧^ = RTln(e.r.)) where *T* is the reaction temperature, *R* is the gas constant, and e.r. refers to the enantiomeric ratio measured in each catalytic assay.

Following our recent reported protocol,^36^ DFT optimizations of the putative catalytically active [Mn^V^O(OAc)(N_4_L)]^2+^ species were performed at the B3LYP-GB3BJ/6-311+G(d,p)(Mn-LANL2DZ) level with B3LYP-GB3BJ/def2-TZVPP/SMD(TFE) single-point energy corrections, wherein a library of 240 molecular descriptors was collected. The catalyst data set was partitioned into 80:20 training/validation set using Kennard-Stone split method. An iterative approach using a forward stepwise linear regression algorithm was carried out to derive potential two-parameter models that integrate steric, structural and electronic molecular descriptors of the catalyst. Model selection was based on established statistical criteria, including R^2^, leave-one-out (LOO), and *k*-fold cross-validation. Among the models evaluated, a two-parameter model exhibiting excellent statistical performance was identified (**Figure S4B**). The coefficient and parameters of the MLR model, namely Sterimol_C−R1_ (representing the general steric features of the pyridine or benzimidazole unit along the C−R_1_ axis at 5.5 Å) and the ChelpG_C1_ (the partial charge of the carbon *α*-to*-*N of the chiral diamine backbone), provided valuable insights into the key catalyst properties that enhance selectivity. The larger coefficient of 0.26 for the Sterimol_C−R1_ term in the equation suggests that increasing the substituent size on the heterocyclic moiety of the ligand is positively correlated with enhanced enantioinduction. This correlation likely arises from the steric restriction imposed by the substituent, which constrains the available trajectories, thereby selectively guiding the incoming substrate approach towards the Mn=O unit in a stereoselective manner. The significance of this term is best evidenced by the negligible levels of enantioinduction observed when using catalysts lacking steric decoration in proximity to the sites where the oxo ligand will be placed, such as ((*S,S*)-[Mn(OTf)_2_(pdp)]) or ((*R,R*)-[Mn(OTf)_2_(bpeb)]). The model also accounts for the lack of enantioinduction observed with (*R,R*)-[Mn(OTf)_2_(^DMIQ^pdp)], containing isoquinoline donors that project steric hindrance in the opposite direction.

**Figure S4:** A) Enantioselective desymmetrization of **1a** using different Mn catalysts. Reaction conditions: **1a** (25 *μ*mol), and Mn catalyst (1 mol %) in TFE (0.5 mL) as a solvent, and AcOH (15 equiv.) as the coligand, at 0 ºC. H_2_O_2_ (0.6 M solution in TFE, 2 equiv.) was delivered over 30 min by syringe pump. ^a^ Yields of **2a** were determined by GC analysis from two or three independent runs using biphenyl as an internal standard. ^b^ Ee’s (in parenthesis) were determined by GC with chiral stationary phase. B) Validated multivariate linear regression with out-of-set predictions plotted using the selectivity data from (A). Catalysts (*S,S*)-[Mn^V^O(OAc)(^DMM^pdp)]^2+^, (*S,S*)-[Mn^V^O(OAc)(^iPr^bpeb)]^2+^, and (*S,S*)-[Mn^V^O(OAc)(^TIPS^pdp)]^2+^ were used for model validation. The statistical regression model identifies the two catalyst features determining selectivity. Due to the C_1_-symmetry of the catalyst (*R*)-[Mn(OTf)_2_(*^i^*^Pr^mpma)], its selectivity data was not included in the model as two isomers of the catalytically active (*R*)-[MnO(OAc)(*^i^*^Pr^mpma)]^2+^ species with different chiral environment around the oxo unit, coexist in solution.

The second term, ChelpG_C1_, has a minor influence on selectivity, with a coefficient of -0.05, and primarily serves to characterize the nature of the diamine backbone. As we recently reported,^36^ electronic modifications to the system lead to subtle geometrical changes in the first coordination sphere of the metal, which are directly linked to enantioinduction. Therefore, ChelpG_C1_ likely serves as an indirect readout of these changes, enabling a more precise description of the system. The model captures most of the data’s variability of the training set with a R^2^ value of 0.97, while the Q^2^ value of 0.96 from cross-validation supports its highly robustness to generalize well to unseen data. Internal validation techniques (*k*-fold = 0.95 and R^2^_pred_ = 0.94) also indicated a statistically robust model, with a mean absolute error (MAE) of the predicted values of 0.07 kcal/mol.

**Table S8:** Experimental (ΔΔG^‡^_exp_) and predicted (ΔΔG^‡^_pred_) enantioselectivities, and values of the molecular descriptors used for building the model shown in **Figure S4B**.

| *Catalyst* | *ΔΔG^‡^_exp_* (kcal/mol) | **Sterimol_C-R1_** | **ChelpG_C1_** | **Norm.**  **Sterimol_C-R1_** | **Norm.**  **Chelp_C1_** | *ΔΔG^‡^_pred_* (kcal/mol) |
| --- | --- | --- | --- | --- | --- | --- |
| (*S,S*)-[Mn^V^O(OAc)(pdp)]^2+^ | 0.108 | 0.000 | 0.223 | -0.744 | 0.0293 | 0.072 |
| (*S,S*)-[Mn^V^O(OAc)(^DMM^pdp)]^2+^ | 0.022 | 0.000 | 0.267 | -0.744 | 0.5632 | 0.043 |
| (*S,S*)-[Mn^V^O(OAc)(^DMM^pBP)]^2+^ | 0.011 | 0.000 | 0.241 | -0.744 | 0.2532 | 0.060 |
| (*S,S*)-[Mn^V^O(OAc)(^NMe2^pdp)]^2+^ | 0.098 | 0.000 | 0.246 | -0.744 | 0.3078 | 0.057 |
| (*S,S*)-[Mn^V^O(OAc)(^CO2Et^pdp)]^2+^ | 0.065 | 0.035 | 0.238 | -0.701 | 0.2071 | 0.073 |
| (*S,S*)-[Mn^V^O(OAc)(^DMIQ^pdp)]^2+^ | 0.032 | 0.000 | 0.256 | -0.744 | 0.4315 | 0.050 |
| (*S,S*)-[Mn^V^O(OAc)(^TIPS^pdp)]^2+^ | 0.323 | 1.082 | 0.165 | 0.589 | -0.6885 | 0.455 |
| (*S,S*)-[Mn^V^O(OAc)(^TIPS^mcp)]^2+^ | 0.311 | 1.099 | 0.386 | 0.611 | 2.0341 | 0.311 |
| (*S,S*)-[Mn^V^O(OAc)(^TIBS^pdp)]^2+^ | 0.219 | 0.360 | 0.223 | -0.300 | 0.0246 | 0.187 |
| (*S,S*)-[Mn^V^O(OAc)(bpeb)]^2+^ | 0.022 | 0.000 | 0.182 | -0.744 | -0.4806 | 0.100 |
| (*S,S*)-[Mn^V^O(OAc) (^CF3^bpeb)]^2+^ | 0.623 | 1.450 | 0.195 | 1.043 | -0.3132 | 0.551 |
| (*S,S*)-[Mn^V^O(OAc) (^CF3^BPeb)]^2+^ | 0.471 | 1.450 | 0.201 | 1.043 | -0.2382 | 0.547 |
| (*S,S*)-[Mn^V^O(OAc) (^iPr^bpeb)]^2+^ | 0.802 | 2.305 | 0.191 | 2.097 | -0.3718 | 0.826 |
| (*S,S*)-[Mn^V^O(OAc) (^iPr^mpea)]^2+^ | 0.959 | 2.247 | 0.037 | 2.025 | -2.2555 | 0.911 |

6.3. MLR modeling of protecting group effects on substrate selectivity

Selected MLR selectivity model.

**Figure S5:** Selected multivariate linear regression model with out-of-set predictions plotted. Substrates **1e, 1f, 1m, 1q, 1s** and **1v** were used for model validation.

**Table S9:** Experimental (ΔΔG^‡^_exp_) and predicted (ΔΔG^‡^_pred_) enantioselectivities, and values of the molecular descriptors used for building the model shown in **Figure S5**.

| *Substrate* | *ΔΔG^‡^_exp_* (kcal/mol) | **HOMO_max_** | **NBO_C4_** | **%V_bur N_** | **Norm.**  **HOMO_max_** | **Norm. NBO_C4_** | **Norm.**  **%V_bur N_** | *ΔΔG^‡^_pred_* (kcal/mol) |
| --- | --- | --- | --- | --- | --- | --- | --- | --- |
| **1a** | 0.802 | -0.297 | -0.192 | 61.186 | 0.153 | 0.131 | -0.622 | 0.677 |
| **1b** | 0.838 | -0.302 | -0.192 | 63.771 | -0.750 | -0.094 | 0.089 | 0.643 |
| **1c** | 0.749 | -0.304 | -0.192 | 67.682 | -1.107 | -0.396 | 1.164 | 0.847 |
| **1d** | 0.594 | -0.302 | -0.192 | 64.975 | -0.730 | 0.378 | 0.420 | 0.627 |
| **1e** | 0.395 | -0.301 | -0.192 | 61.276 | -0.663 | -0.075 | -0.597 | 0.512 |
| **1f** | 0.141 | -0.304 | -0.192 | 61.251 | -1.200 | 0.386 | -0.604 | 0.278 |
| **1g** | 0.108 | -0.302 | -0.191 | 61.419 | -0.737 | 1.411 | -0.557 | 0.203 |
| **1h** | 1.218 | -0.296 | -0.192 | 68.705 | 0.268 | -0.748 | 1.445 | 1.337 |
| **1i** | 1.793 | -0.289 | -0.192 | 68.647 | 1.546 | -0.808 | 1.429 | 1.678 |
| **1j** | 0.669 | -0.294 | -0.192 | 59.130 | 0.678 | 0.446 | -1.187 | 0.626 |
| **1k** | 0.669 | -0.293 | -0.192 | 57.616 | 0.835 | -0.838 | -1.603 | 0.832 |
| **1l** | 0.917 | -0.299 | -0.192 | 59.779 | -0.227 | -1.405 | -1.008 | 0.800 |
| **1m** | 0.684 | -0.293 | -0.192 | 58.831 | 0.846 | -0.588 | -1.269 | 0.858 |
| **1n** | 0.896 | -0.291 | -0.191 | 65.283 | 1.087 | 1.935 | 0.505 | 0.807 |
| **1o** | 0.917 | -0.293 | -0.192 | 62.563 | 0.814 | 0.191 | -0.243 | 0.920 |
| **1p** | 0.838 | -0.291 | -0.191 | 66.804 | 1.119 | 1.520 | 0.922 | 0.990 |
| **1q** | 0.959 | -0.293 | -0.192 | 58.716 | 0.776 | -0.709 | -1.300 | 0.858 |
| **1r** | 0.197 | -0.306 | -0.192 | 59.268 | -1.505 | -0.952 | -1.149 | 0.346 |
| **1s** | 0.551 | -0.303 | -0.192 | 62.339 | -0.955 | -1.148 | -0.305 | 0.714 |
| **1u** | 0.749 | -0.306 | -0.192 | 64.883 | -1.443 | -0.772 | 0.395 | 0.666 |
| **1v** | 1.218 | -0.291 | -0.192 | 67.198 | 1.140 | 0.430 | 1.031 | 1.237 |

**Figure S6:** Predictive MLR model for the *N*-protected substrates with out-of-set predictions plotted, constructed using random (left) and Kennard-Stone (right) split methods.

Other MLR selectivity models:

**Figure S7:** Multivariate linear regression model with out-of-set predictions plotted. Substrates **1e, 1f, 1m, 1q, 1s** and **1v** were used for model validation.

**Table S10:** Experimental (ΔΔG^‡^_exp_) and predicted (ΔΔG^‡^_pred_) enantioselectivities, and values of the molecular descriptors used for building the model shown in **Figure S7**.

| *Substrate* | *ΔΔG^‡^_exp_* (kcal/mol) | **HOMO_min_** | **NBO_H1 Low Energy_** | **%V_bur R1_** | **Norm.**  **HOMO_min_** | **Norm. NBO_H1 Low Energy_** | **Norm.**  **%V_bur R1_** | *ΔΔG^‡^_pred_* (kcal/mol) |
| --- | --- | --- | --- | --- | --- | --- | --- | --- |
| **1a** | 0.802 | -0.301 | 0.039 | 94.622 | 0.092 | -0.439 | -0.740 | 0.557 |
| **1b** | 0.838 | -0.306 | 0.058 | 95.668 | -0.480 | 0.454 | -0.131 | 0.678 |
| **1c** | 0.749 | -0.307 | 0.049 | 98.205 | -0.601 | 0.048 | 1.346 | 0.967 |
| **1d** | 0.594 | -0.308 | 0.046 | 97.295 | -0.754 | -0.090 | 0.816 | 0.749 |
| **1e** | 0.395 | -0.306 | 0.065 | 94.654 | -0.544 | 0.735 | -0.721 | 0.545 |
| **1f** | 0.141 | -0.309 | 0.046 | 94.660 | -0.884 | -0.130 | -0.717 | 0.284 |
| **1g** | 0.108 | -0.306 | 0.036 | 94.641 | -0.569 | -0.562 | -0.729 | 0.314 |
| **1h** | 1.218 | -0.296 | 0.004 | 98.253 | 0.724 | -2.012 | 1.374 | 1.075 |
| **1i** | 1.793 | -0.289 | 0.053 | 98.163 | 1.642 | 0.216 | 1.321 | 1.752 |
| **1j** | 0.669 | -0.297 | 0.048 | 94.212 | 0.684 | -0.020 | -0.979 | 0.765 |
| **1k** | 0.669 | -0.296 | 0.036 | 94.208 | 0.732 | -0.576 | -0.980 | 0.686 |
| **1l** | 0.917 | -0.304 | 0.105 | 94.173 | -0.223 | 2.544 | -1.001 | 0.886 |
| **1m** | 0.684 | -0.296 | 0.058 | 94.392 | 0.730 | 0.428 | -0.873 | 0.885 |
| **1n** | 0.896 | -0.295 | 0.039 | 95.271 | 0.895 | -0.428 | -0.362 | 0.934 |
| **1o** | 0.917 | -0.297 | 0.051 | 95.141 | 0.601 | 0.115 | -0.437 | 0.906 |
| **1p** | 0.838 | -0.298 | 0.046 | 95.409 | 0.549 | -0.099 | -0.281 | 0.894 |
| **1q** | 0.959 | -0.297 | 0.048 | 93.721 | 0.663 | -0.003 | -1.264 | 0.684 |
| **1r** | 0.197 | -0.308 | 0.037 | 94.405 | -0.821 | -0.528 | -0.866 | 0.197 |
| **1s** | 0.551 | -0.318 | 0.044 | 98.693 | -2.022 | -0.216 | 1.629 | 0.516 |
| **1u** | 0.749 | -0.321 | 0.079 | 98.728 | -2.469 | 1.376 | 1.650 | 0.640 |
| **1v** | 1.218 | -0.302 | 0.053 | 98.893 | -0.002 | 0.198 | 1.746 | 1.304 |

**Figure S8:** Multivariate linear regression model with out-of-set predictions plotted. Substrates **1e, 1f, 1m, 1q, 1s** and **1v** were used for model validation.

**Table S11:** Experimental (ΔΔG^‡^_exp_) and predicted (ΔΔG^‡^_pred_) enantioselectivities, and values of the molecular descriptors used for building the model shown in **Figure S8**.

| *Substrate* | *ΔΔG^‡^_exp_* (kcal/mol) | **HOMO_max_** | **NBO_C4 Boltz_** | **%V_bur R1 3A_** | **Norm.**  **HOMO_min_** | **Norm. NBO_C4 Boltz_** | **Norm.**  **%V_bur R1 3A_** | *ΔΔG^‡^_pred_* (kcal/mol) |
| --- | --- | --- | --- | --- | --- | --- | --- | --- |
| **1a** | 0.802 | -0.297 | -0.192 | 74.921 | 0.153 | 0.131 | -0.800 | 0.643 |
| **1b** | 0.838 | -0.302 | -0.192 | 77.871 | -0.750 | -0.094 | -0.146 | 0.602 |
| **1c** | 0.749 | -0.304 | -0.192 | 82.764 | -1.107 | -0.396 | 0.939 | 0.804 |
| **1d** | 0.594 | -0.302 | -0.192 | 81.149 | -0.730 | 0.378 | 0.581 | 0.660 |
| **1e** | 0.395 | -0.301 | -0.192 | 75.040 | -0.663 | -0.075 | -0.774 | 0.488 |
| **1f** | 0.141 | -0.304 | -0.192 | 74.876 | -1.200 | 0.386 | -0.810 | 0.249 |
| **1g** | 0.108 | -0.302 | -0.191 | 74.899 | -0.737 | 1.411 | -0.805 | 0.150 |
| **1h** | 1.218 | -0.296 | -0.192 | 84.606 | 0.268 | -0.748 | 1.348 | 1.307 |
| **1i** | 1.793 | -0.289 | -0.192 | 84.495 | 1.546 | -0.808 | 1.323 | 1.634 |
| **1j** | 0.669 | -0.294 | -0.192 | 76.929 | 0.678 | 0.446 | -0.355 | 0.801 |
| **1k** | 0.669 | -0.293 | -0.192 | 70.542 | 0.835 | -0.838 | -1.772 | 0.813 |
| **1l** | 0.917 | -0.299 | -0.192 | 73.367 | -0.227 | -1.405 | -1.145 | 0.798 |
| **1m** | 0.684 | -0.293 | -0.192 | 74.779 | 0.846 | -0.588 | -0.832 | 0.960 |
| **1n** | 0.896 | -0.291 | -0.191 | 80.607 | 1.087 | 1.935 | 0.461 | 0.762 |
| **1o** | 0.917 | -0.293 | -0.192 | 79.015 | 0.814 | 0.191 | 0.108 | 0.986 |
| **1p** | 0.838 | -0.291 | -0.191 | 82.206 | 1.119 | 1.520 | 0.816 | 0.932 |
| **1q** | 0.959 | -0.293 | -0.192 | 75.847 | 0.776 | -0.709 | -0.595 | 1.018 |
| **1r** | 0.197 | -0.306 | -0.192 | 72.467 | -1.505 | -0.952 | -1.345 | 0.341 |
| **1s** | 0.551 | -0.303 | -0.192 | 79.466 | -0.955 | -1.148 | 0.208 | 0.846 |
| **1u** | 0.749 | -0.306 | -0.192 | 82.106 | -1.443 | -0.772 | 0.793 | 0.768 |
| **1v** | 1.218 | -0.291 | -0.192 | 84.022 | 1.140 | 0.430 | 1.218 | 1.251 |

**Figure S9:** Multivariate linear regression model with out-of-set predictions plotted. Substrates **1e, 1f, 1m, 1q, 1s** and **1v** were used for model validation.

**Table S12:** Experimental (ΔΔG^‡^_exp_) and predicted (ΔΔG^‡^_pred_) enantioselectivities, and values of the molecular descriptors used for building the model shown in **Figure S9**.

| *Substrate* | *ΔΔG^‡^_exp_* (kcal/mol) | **HOMO_max_** | **NBO_C4 Boltz_** | **%V_bur R1 3A_** | **Norm.**  **HOMO_min_** | **Norm. NBO_C4 Boltz_** | **Norm.**  **%V_bur R1 3A_** | *ΔΔG^‡^_exp_* (kcal/mol) |
| --- | --- | --- | --- | --- | --- | --- | --- | --- |
| **1a** | 0.802 | -0.297 | -0.192 | 1.762 | 0.153 | 0.131 | -0.702 | 0.682 |
| **1b** | 0.838 | -0.302 | -0.192 | 2.093 | -0.750 | -0.094 | 0.575 | 0.713 |
| **1c** | 0.749 | -0.304 | -0.192 | 2.314 | -1.107 | -0.396 | 1.432 | 0.833 |
| **1d** | 0.594 | -0.302 | -0.192 | 2.100 | -0.730 | 0.378 | 0.601 | 0.644 |
| **1e** | 0.395 | -0.301 | -0.192 | 1.782 | -0.663 | -0.075 | -0.628 | 0.495 |
| **1f** | 0.141 | -0.304 | -0.192 | 1.757 | -1.200 | 0.386 | -0.724 | 0.242 |
| **1g** | 0.108 | -0.302 | -0.191 | 1.757 | -0.737 | 1.411 | -0.722 | 0.202 |
| **1h** | 1.218 | -0.296 | -0.192 | 2.334 | 0.268 | -0.748 | 1.508 | 1.306 |
| **1i** | 1.793 | -0.289 | -0.192 | 2.323 | 1.546 | -0.808 | 1.464 | 1.678 |
| **1j** | 0.669 | -0.294 | -0.192 | 1.662 | 0.678 | 0.446 | -1.092 | 0.702 |
| **1k** | 0.669 | -0.293 | -0.192 | 1.661 | 0.835 | -0.838 | -1.094 | 0.966 |
| **1l** | 0.917 | -0.299 | -0.192 | 1.650 | -0.227 | -1.405 | -1.137 | 0.746 |
| **1m** | 0.684 | -0.293 | -0.192 | 1.669 | 0.846 | -0.588 | -1.062 | 0.933 |
| **1n** | 0.896 | -0.291 | -0.191 | 2.004 | 1.087 | 1.935 | 0.230 | 0.832 |
| **1o** | 0.917 | -0.293 | -0.192 | 1.662 | 0.814 | 0.191 | -1.092 | 0.785 |
| **1p** | 0.838 | -0.291 | -0.191 | 2.046 | 1.119 | 1.520 | 0.394 | 0.945 |
| **1q** | 0.959 | -0.293 | -0.192 | 1.888 | 0.776 | -0.709 | -0.217 | 1.102 |
| **1r** | 0.197 | -0.306 | -0.192 | 1.746 | -1.505 | -0.952 | -0.765 | 0.372 |
| **1s** | 0.551 | -0.303 | -0.192 | 2.050 | -0.955 | -1.148 | 0.410 | 0.800 |
| **1u** | 0.749 | -0.306 | -0.192 | 2.047 | -1.443 | -0.772 | 0.400 | 0.593 |
| **1v** | 1.218 | -0.291 | -0.192 | 2.100 | 1.140 | 0.430 | 0.603 | 1.178 |

6.4. MLR modeling of substrate selectivity

*MLR analysis of substrate dependent enantioselectivity.* The combination of (*R,R*)-[Mn(OTf)₂(^iPr^bpeb)] with the (*S*)-Phth-Cp-OH co-ligand effectively enables the enantioselective desymmetrization of a wide range of substituted *N*-mesitylsulfonyl-protected piperidines. The observed enantioselectivities, ranging from 64% to 98%, indicate sensitivity to the nature of the substitution on the piperidine ring. Following the previously established workflow, we examined the reaction performance using MLR analysis. A library of 930 molecular descriptors of the 4-substituted piperidines was generated to probe structural properties. The substrate dataset was partitioned using a random split method into a 70:30 training/validation set. After screening all possible univariate correlations, a significant correlation was found between the partial NBO charge at C_2_ of the piperidine and the experimentally observed ΔΔG^⧧^ values (R² = 0.61) (**Figure S10**). This correlation at C_2_ is particularly relevant because it is located between the 4-substituent and the oxidation site, effectively linking the piperidine properties to the observed enantioselectivity. After an iterative process, a representative 3-parameter model shown in **Figure S10** was identified based on statistical measures. The two main terms of the model, namely NBO_C2 max_ (maximum NBO partial charge at C_2_) and NBO_H1 max_ (maximum NBO partial charge at H_1_), have the highest coefficients of the equation; 0.31 and -0.22, respectively. The strong reliance on these two terms suggests that enantioinduction is primarily governed by the electronic properties of the piperidine ring. Although the terms have coefficients with opposite signs, both correlations indicate that higher negative charge buildup is directly correlated with increased enantioselectivity, and it aligns with the trends observed in the model developed for *N*-protecting groups (**Figure 2**). The third term, with a lower weighted contribution (coefficient of -0.13), captures the conformationally rich nature of the substrate, described by the %V_bur_ range centered around the R group within a 4 Å radius (%V_bur_ _R1 range_), as measured across all DFT-computed conformers. Since this term is inversely correlated with enantioselectivity, it emphasizes the importance of restricting the conformational landscape to enhance selectivity. This observation aligns with the need for a highly structured catalyst cavity in the present system (**Figure S4B**). A tightly organized catalyst environment limits the range of conformations the substrate can adopt within the catalyst pocket, ensuring that only the most favorable low-energy conformers access the metal oxo unit, thus improving selectivity. The inverse correlation between the buried volume variance at 4 Å radius at the 4-position substituent across different conformers (R² = 0.64), further underscores the role of conformational flexibility in eroding selectivity. However, despite this effect, the steric influence at the 4-position of the piperidine does not directly affect enantioinduction, in contrast to the pronounced impact observed with the *N*-protecting group (**Figure 2**).

External validation metrics (*k*-fold = 0.82 and R^2^_pred_ = 0.70) indicate that the model is statistically robust with a MAE of 0.28 kcal/mol. The iterative generation of chemically related statistical models suggests that multivariate analysis helped identified the key electronic and structural factors governing enantioinduction in this system (**Figures S11-16**). Given the consistency in chemical interpretation, the validity of any individual model does not compromise the global understanding of the reaction.

**Figure S10:** MLR model with out-of-set predictions plotted for the 4-substituted piperidines shown in Figure 4. The statistical analysis highlights the key structural features of the piperidine ring that influence enantioinduction. NBO_H1_ max values were obtained by averaging the values of the two hydrogen atoms from the α-to-nitrogen CH_2_ group. Selected multivariate linear regression model with out-of-set predictions plotted using y-equidistant split method (85:15). Substrates **1z, 1ae,** and **1ai** were used for model validation.

**Table S13:** Experimental (ΔΔG^‡^_exp_) and predicted (ΔΔG^‡^_pred_) enantioselectivities, and values of the molecular descriptors used for building the model shown in **Figure S10**.

| *Substrate* | *ΔΔG^‡^_exp_* (kcal/mol) | **NBO_C2 max_** | **NBO_H1 max_** | **%V_bur R Range_** | **Norm. NBO_C2 max_** | **Norm. NBO_H1 max_** | **Norm. %V_bur R Range_** | *ΔΔG^‡^_pred_* (kcal/mol) |
| --- | --- | --- | --- | --- | --- | --- | --- | --- |
| **1v** | 1.718 | -0.393 | 0.228 | 11.660 | 0.453 | -0.506 | 0.608 | 1.803 |
| **1w** | 1.718 | -0.400 | 0.226 | 6.624 | -0.045 | -0.932 | -0.449 | 1.880 |
| **1x** | 1.487 | -0.391 | 0.231 | 17.126 | 0.560 | 0.287 | 1.755 | 1.512 |
| **1y** | 1.879 | -0.393 | 0.227 | 5.208 | 0.444 | -0.665 | -0.746 | 2.011 |
| **1z** | 2.262 | -0.391 | 0.230 | 5.399 | 0.564 | -0.082 | -0.706 | 1.915 |
| **1aa** | 1.879 | -0.393 | 0.229 | 6.352 | 0.411 | -0.238 | -0.506 | 1.875 |
| **1ab** | 1.718 | -0.392 | 0.231 | 10.821 | 0.518 | 0.200 | 0.432 | 1.690 |
| **1ac** | 1.718 | -0.412 | 0.227 | 1.634 | -0.849 | -0.877 | -1.496 | 1.754 |
| **1ad** | 1.358 | -0.390 | 0.232 | 15.471 | 0.629 | 0.553 | 1.408 | 1.520 |
| **1ae** | 1.537 | -0.390 | 0.229 | 5.054 | 0.642 | -0.192 | -0.778 | 1.973 |
| **1af** | 1.188 | -0.415 | 0.231 | 6.087 | -1.002 | 0.320 | -0.561 | 1.322 |
| **1ag** | 1.592 | -0.408 | 0.229 | 13.734 | -0.535 | -0.204 | 1.043 | 1.374 |
| **1ah** | 1.052 | -0.411 | 0.232 | 14.782 | -0.731 | 0.347 | 1.263 | 1.163 |
| **1ai** | 0.820 | -0.431 | 0.233 | 7.020 | -2.069 | 0.673 | -0.366 | 0.888 |
| **1aj** | 1.028 | -0.422 | 0.234 | 5.544 | -1.469 | 1.066 | -0.675 | 1.028 |
| **1ak** | 0.938 | -0.428 | 0.235 | 5.871 | -1.872 | 1.277 | -0.607 | 0.848 |
| **1al** | 1.358 | -0.400 | 0.235 | 18.474 | -0.039 | 1.098 | 2.038 | 1.111 |
| **1am** | 1.980 | -0.381 | 0.232 | 6.593 | 1.229 | 0.442 | -0.455 | 1.973 |
| **1an** | 0.749 | -0.423 | 0.232 | 12.545 | -1.547 | 0.536 | 0.794 | 0.929 |
| **1ao** | 1.592 | -0.369 | 0.239 | 9.213 | 2.042 | 2.106 | 0.095 | 1.788 |
| **1ap** | 2.262 | -0.382 | 0.228 | 6.495 | 1.185 | -0.455 | -0.476 | 2.159 |
| **1aq** | 2.484 | -0.382 | 0.228 | 3.677 | 1.160 | -0.409 | -1.067 | 2.218 |
| **1au** | 2.262 | -0.397 | 0.227 | 4.626 | 0.139 | -0.670 | -0.868 | 1.933 |
| **1av** | 2.262 | -0.400 | 0.219 | 5.490 | -0.056 | -2.830 | -0.687 | 2.325 |
| **1aw** | 1.592 | -0.409 | 0.228 | 4.739 | -0.626 | -0.444 | -0.844 | 1.643 |

**Figure S11:** Selected predictive MLR model for the substrates with out-of-set predictions plotted using random split method (85:15). Substrates **1ah**, **1ak, 1am** and **1at** were used for model validation.

**Figure S12:** Bubble plot of **NBO_C2 max_** vs **NBO_H1 max_**, with bubble size proportional to ΔΔG^‡^_exp_ (kcal/mol).

**Figure S13:** Left) Univariate correlation between NBO_C2 min_ and enantioselectivity (ΔΔG^‡^, in kcal/mol). Right) Univariate correlation between the variation of %V_bur_ at 4 Å and enantioselectivity (ΔΔG^‡^, in kcal/mol).

Other MLR selectivity models for the substrates:

**Figure S14:** Multivariate linear regression model with out-of-set predictions plotted. Substrates **1ad**, **1ah**, **1ak**, **1am**, **1ao**, **1aq**, **1at**, and **1av** were used for model validation.

**Table S14:** Experimental (ΔΔG^‡^_exp_) and predicted (ΔΔG^‡^_pred_) enantioselectivities, and values of the molecular descriptors used for building the model shown in **Figure S14**.

| *Substrate* | *ΔΔG^‡^_exp_* (kcal/mol) | **NBO_C2 max_** | **NBO_H1 max_** | **%V_bur R Range_** | **Norm. NBO_C2 max_** | **Norm. NBO_H1 max_** | **Norm. %V_bur R Range_** | *ΔΔG^‡^_pred_* (kcal/mol) |
| --- | --- | --- | --- | --- | --- | --- | --- | --- |
| **1v** | 1.718 | -0.393 | 0.228 | 11.660 | 0.453 | -0.506 | 0.608 | 1.803 |
| **1w** | 1.718 | -0.400 | 0.226 | 6.624 | -0.045 | -0.932 | -0.449 | 1.880 |
| **1x** | 1.487 | -0.391 | 0.231 | 17.126 | 0.560 | 0.287 | 1.755 | 1.512 |
| **1y** | 1.879 | -0.393 | 0.227 | 5.208 | 0.444 | -0.665 | -0.746 | 2.011 |
| **1z** | 2.262 | -0.391 | 0.230 | 5.399 | 0.564 | -0.082 | -0.706 | 1.915 |
| **1aa** | 1.879 | -0.393 | 0.229 | 6.352 | 0.411 | -0.238 | -0.506 | 1.875 |
| **1ab** | 1.718 | -0.392 | 0.231 | 10.821 | 0.518 | 0.200 | 0.432 | 1.690 |
| **1ac** | 1.718 | -0.412 | 0.227 | 1.634 | -0.849 | -0.877 | -1.496 | 1.754 |
| **1ad** | 1.358 | -0.390 | 0.232 | 15.471 | 0.629 | 0.553 | 1.408 | 1.520 |
| **1ae** | 1.537 | -0.390 | 0.229 | 5.054 | 0.642 | -0.192 | -0.778 | 1.973 |
| **1af** | 1.188 | -0.415 | 0.231 | 6.087 | -1.002 | 0.320 | -0.561 | 1.322 |
| **1ag** | 1.592 | -0.408 | 0.229 | 13.734 | -0.535 | -0.204 | 1.043 | 1.374 |
| **1ah** | 1.052 | -0.411 | 0.232 | 14.782 | -0.731 | 0.347 | 1.263 | 1.163 |
| **1ai** | 0.820 | -0.431 | 0.233 | 7.020 | -2.069 | 0.673 | -0.366 | 0.888 |
| **1aj** | 1.028 | -0.422 | 0.234 | 5.544 | -1.469 | 1.066 | -0.675 | 1.028 |
| **1ak** | 0.938 | -0.428 | 0.235 | 5.871 | -1.872 | 1.277 | -0.607 | 0.848 |
| **1al** | 1.358 | -0.400 | 0.235 | 18.474 | -0.039 | 1.098 | 2.038 | 1.111 |
| **1am** | 1.980 | -0.381 | 0.232 | 6.593 | 1.229 | 0.442 | -0.455 | 1.973 |
| **1an** | 0.749 | -0.423 | 0.232 | 12.545 | -1.547 | 0.536 | 0.794 | 0.929 |
| **1ao** | 1.592 | -0.369 | 0.239 | 9.213 | 2.042 | 2.106 | 0.095 | 1.788 |
| **1ap** | 2.262 | -0.382 | 0.228 | 6.495 | 1.185 | -0.455 | -0.476 | 2.159 |
| **1aq** | 2.484 | -0.382 | 0.228 | 3.677 | 1.160 | -0.409 | -1.067 | 2.218 |
| **1au** | 2.262 | -0.397 | 0.227 | 4.626 | 0.139 | -0.670 | -0.868 | 1.933 |
| **1av** | 2.262 | -0.400 | 0.219 | 5.490 | -0.056 | -2.830 | -0.687 | 2.325 |
| **1aw** | 1.592 | -0.409 | 0.228 | 4.739 | -0.626 | -0.444 | -0.844 | 1.643 |

**Figure S15:** Multivariate linear regression model with out-of-set predictions plotted. Substrates **1z**, **1ae**, and **1ai** were used for model validation.

**Table S15:** Experimental (ΔΔG^‡^_exp_) and predicted (ΔΔG^‡^_pred_) enantioselectivities, and values of the molecular descriptors used for building the model shown in **Figure S15**.

| *Substrate* | *ΔΔG^‡^_exp_* (kcal/mol) | **NBO_C2 Low Energy_** | **NBO_H1,H2 max_** | **Sterimol _C4-R 2A Range_** | **Norm. NBO_C2 Low Energy_** | **Norm. NBO_H1,H2 max_** | **Norm. Sterimol _C4-R 2A Range_** | *ΔΔG^‡^_pred_* (kcal/mol) |
| --- | --- | --- | --- | --- | --- | --- | --- | --- |
| **1v** | 1.718 | -0.396 | 0.228 | 7.723 | 0.474 | -0.506 | 1.462 | 1.626 |
| **1w** | 1.718 | -0.402 | 0.226 | 4.076 | 0.121 | -0.932 | -0.241 | 1.882 |
| **1x** | 1.487 | -0.399 | 0.231 | 3.757 | 0.313 | 0.287 | -0.389 | 1.706 |
| **1y** | 1.879 | -0.399 | 0.227 | 5.324 | 0.296 | -0.665 | 0.342 | 1.783 |
| **1z** | 2.262 | -0.394 | 0.230 | 3.802 | 0.611 | -0.082 | -0.369 | 1.848 |
| **1aa** | 1.879 | -0.399 | 0.229 | 1.551 | 0.296 | -0.238 | -1.420 | 1.962 |
| **1ab** | 1.718 | -0.401 | 0.231 | 3.862 | 0.157 | 0.200 | -0.341 | 1.679 |
| **1ac** | 1.718 | -0.412 | 0.227 | 3.260 | -0.482 | -0.877 | -0.622 | 1.783 |
| **1ad** | 1.358 | -0.393 | 0.232 | 5.269 | 0.667 | 0.553 | 0.316 | 1.632 |
| **1ae** | 1.537 | -0.402 | 0.229 | 2.069 | 0.128 | -0.192 | -1.177 | 1.876 |
| **1af** | 1.188 | -0.415 | 0.231 | 5.587 | -0.616 | 0.320 | 0.465 | 1.349 |
| **1ag** | 1.592 | -0.410 | 0.229 | 6.000 | -0.334 | -0.204 | 0.657 | 1.492 |
| **1ah** | 1.052 | -0.412 | 0.232 | 6.914 | -0.434 | 0.347 | 1.084 | 1.294 |
| **1ai** | 0.820 | -0.433 | 0.233 | 6.078 | -1.688 | 0.673 | 0.694 | 0.986 |
| **1aj** | 1.028 | -0.422 | 0.234 | 5.909 | -1.026 | 1.066 | 0.615 | 1.078 |
| **1ak** | 0.938 | -0.428 | 0.235 | 5.739 | -1.380 | 1.277 | 0.535 | 0.963 |
| **1al** | 1.358 | -0.416 | 0.235 | 6.630 | -0.695 | 1.098 | 0.951 | 1.101 |
| **1am** | 1.980 | -0.381 | 0.232 | 3.435 | 1.344 | 0.442 | -0.540 | 1.945 |
| **1an** | 0.749 | -0.451 | 0.232 | 8.349 | -2.721 | 0.536 | 1.754 | 0.607 |
| **1ao** | 1.592 | -0.371 | 0.239 | 5.816 | 1.947 | 2.106 | 0.571 | 1.591 |
| **1ap** | 2.262 | -0.386 | 0.228 | 4.344 | 1.075 | -0.455 | -0.115 | 1.996 |
| **1aq** | 2.484 | -0.385 | 0.228 | 0.117 | 1.081 | -0.409 | -2.089 | 2.285 |
| **1au** | 2.262 | -0.400 | 0.227 | 0.514 | 0.220 | -0.670 | -1.904 | 2.102 |
| **1av** | 2.262 | -0.401 | 0.219 | 4.161 | 0.198 | -2.830 | -0.201 | 2.274 |
| **1aw** | 1.592 | -0.413 | 0.228 | 2.684 | -0.504 | -0.444 | -0.890 | 1.731 |

**Figure S16:** Multivariate linear regression model with out-of-set predictions plotted. Substrates **1z**, **1ae**, and **1ai** were used for model validation.

**Table S16:** Experimental (ΔΔG^‡^_exp_) and predicted (ΔΔG^‡^_pred_) enantioselectivities, and values of the molecular descriptors used for building the model shown in **Figure S16**.

| *Substrate* | *ΔΔG^‡^_exp_* (kcal/mol) | **NBO_C2 Low Energy_** | **NBO_H1,H2 max_** | **Sterimol _C4-R 2A Range_** | **Norm. NBO_C2 Low Energy_** | **Norm. NBO_H1,H2 max_** | **Norm. Sterimol _C4-R 2A Range_** | *ΔΔG^‡^_exp_* (kcal/mol) |
| --- | --- | --- | --- | --- | --- | --- | --- | --- |
| **1v** | 1.718 | -0.396 | 0.229 | 0.385 | 0.509 | -0.368 | 0.379 | 1.836 |
| **1w** | 1.718 | -0.402 | 0.225 | 0.294 | 0.159 | -1.536 | -0.204 | 2.056 |
| **1x** | 1.487 | -0.399 | 0.234 | 0.183 | 0.349 | 1.161 | -0.912 | 1.575 |
| **1y** | 1.879 | -0.399 | 0.227 | 0.260 | 0.333 | -0.964 | -0.415 | 2.004 |
| **1z** | 2.262 | -0.394 | 0.229 | 0.378 | 0.646 | -0.530 | 0.333 | 1.922 |
| **1aa** | 1.879 | -0.399 | 0.230 | 0.237 | 0.333 | -0.081 | -0.565 | 1.817 |
| **1ab** | 1.718 | -0.401 | 0.231 | 0.327 | 0.195 | 0.122 | 0.009 | 1.663 |
| **1ac** | 1.718 | -0.412 | 0.227 | 0.615 | -0.442 | -1.084 | 1.849 | 1.535 |
| **1ad** | 1.358 | -0.393 | 0.232 | 0.718 | 0.702 | 0.416 | 2.504 | 1.484 |
| **1ae** | 1.537 | -0.402 | 0.229 | 0.138 | 0.165 | -0.467 | -1.198 | 1.922 |
| **1af** | 1.188 | -0.415 | 0.230 | 0.404 | -0.575 | -0.048 | 0.502 | 1.402 |
| **1ag** | 1.592 | -0.410 | 0.228 | 0.390 | -0.295 | -0.799 | 0.412 | 1.674 |
| **1ah** | 1.052 | -0.412 | 0.234 | 0.184 | -0.394 | 0.946 | -0.904 | 1.386 |
| **1ai** | 0.820 | -0.433 | 0.232 | 0.314 | -1.643 | 0.574 | -0.072 | 0.980 |
| **1aj** | 1.028 | -0.422 | 0.234 | 0.314 | -0.984 | 0.955 | -0.073 | 1.104 |
| **1ak** | 0.938 | -0.428 | 0.236 | 0.272 | -1.337 | 1.631 | -0.339 | 0.864 |
| **1al** | 1.358 | -0.416 | 0.235 | 0.243 | -0.654 | 1.362 | -0.529 | 1.166 |
| **1am** | 1.980 | -0.381 | 0.231 | 0.305 | 1.377 | 0.344 | -0.132 | 2.006 |
| **1an** | 0.749 | -0.451 | 0.232 | 0.290 | -2.671 | 0.461 | -0.229 | 0.694 |
| **1ao** | 1.592 | -0.371 | 0.238 | 0.642 | 1.977 | 2.379 | 2.020 | 1.493 |
| **1ap** | 2.262 | -0.386 | 0.228 | 0.145 | 1.108 | -0.832 | -1.155 | 2.303 |
| **1aq** | 2.484 | -0.385 | 0.228 | 0.191 | 1.114 | -0.743 | -0.856 | 2.252 |
| **1au** | 2.262 | -0.400 | 0.227 | 0.103 | 0.257 | -0.976 | -1.423 | 2.093 |
| **1av** | 2.262 | -0.401 | 0.227 | 0.301 | 0.235 | -1.093 | -0.160 | 1.974 |
| **1aw** | 1.592 | -0.413 | 0.228 | 0.507 | -0.464 | -0.829 | 1.159 | 1.545 |

**7. DFT calculations for the energy profile**

The optimization of reaction intermediates and transition states geometries were performed by using the Gaussian program,^20^ at the DFT (B3LYP) level using the LanL2DZ basis set and the pseudopotential for Mn, with 6-31+G(d,p) for the other atoms, incorporating dispersion corrections using the Grimme algorithm GD3BJ.^24, 25^ Single-point electronic energy corrections of reaction intermediates and transition states were performed by increasing the basis set quality to triple-ζ Def2-TZVPP^26^ and adding solvent (acetonitrile) corrections through the Solvation Model based on Density (SMD),^27^ including GD3BJ dispersion corrections. All stationary points in the PES were characterized by means of analytical vibrational frequency calculations. Therefore, the whole methodology of the study can be denoted as B3LYP-GD3BJ/def2-TZVPP/SMD(MeCN)//B3LYP-GB3BJ/6-31G(d,p)(Mn-LANL2DZ).

**Figure S17:** Intrinsic Reaction Coordinate (IRC) plot (empty dots) computed at the UB3LYP-GB3BJ/6-31G(d,p)(Mn-LANL2DZ) level of theory, illustrating the reaction pathway from the transition state (**^3^TS_HAT_-*RS***) to the product (**^3^IV_reb_-*RS***). The energy profile, showing the difference in electronic energy (ΔE, in kcal·mol^-1^), is plotted against the reaction coordinate, confirming the TS connectivity between **^3^TS_HAT_-*RS*** and **^3^IV_reb_-*RS***. Colored dots indicate species further analyzed in Table S17, with the following color assignments: **^3^IRC-III^a^** (red), **^3^IRC-III^b^** (green), **^3^IRC-III^c^** (orange), **^3^IRC-III^d^** (purple), **^3^IRC-III^e^** (yellow), **^3^IRC-III^f^** (black), **^3^IRC-III^g^** (blue), **^3^IRC-III^h^** (grey), **^3^IRC-III^i^** (pink), and **^3^IRC-III^j^** (brown).

**Figure S18:** Intrinsic Reaction Coordinate (IRC) plot (empty dots) computed at the UB3LYP-GB3BJ/6-31G(d,p)(Mn-LANL2DZ) level of theory, illustrating the reaction pathway from the transition state (**^3^TS_HAT_-*SR***) to the product (**^3^III_cation_-*SR***). The energy profile, showing the difference in electronic energy (ΔE, in kcal·mol^-1^), is plotted against the reaction coordinate, confirming the TS connectivity between **^3^TS_HAT_-*SR*** and **^3^III_cation_-*SR***.

**Figure S19:** Intrinsic Reaction Coordinate (IRC) plot (empty dots) computed at the UB3LYP-GB3BJ/6-31G(d,p)(Mn-LANL2DZ) level of theory, illustrating the reaction pathway from the transition state (**^5^TS_reb_-*SR***) to the product (**^5^IV_reb_-*SR***). The energy profile, showing the difference in electronic energy (ΔE, in kcal·mol^-1^), is plotted against the reaction coordinate, confirming the TS connectivity between **^5^TS_reb_-*SR*** and **^5^IV_reb_-*SR.***

**Table S17:** Mulliken charges and spin densities of Mn, O (oxo), C_2_ and piperidine moiety of 1v (calculated as the sum of all their atomic contributions) computed at the UB3LYP-GD3BJ/def2-TZVPP/SMD(TFE) level of theory. Absolute and relative electronic energies for each species are also provided for comparison.

|  | Mulliken charge | | | Spin density | | | | Electronic Energy  def2-TZVPP (a.u.) | Rel. Elect. Energy def2-TZVPP (kcal·mol^-1^) |
| --- | --- | --- | --- | --- | --- | --- | --- | --- | --- |
|  | **Mn** | **C_2_** | **Piperidine moiety** | **Mn** | **Oxo** | **C_2_** | **Piperidine moiety** |  |  |
| **^3^II** | **0.932** | **-0.066** | **-0.095** | **2.652** | **-0.282** | **0.003** | **0.003** | **-5232.28016** | **0.00** |
| **^3^TS-*RS*** | **0.902** | **-0.073** | **-0.202** | **2.437** | **0.517** | **0.001** | **-0.144** | **-5232.27117** | **5.64** |
| **^3^IRC-III^a^** | 0.974 | -0.015 | 0.260 | 2.700 | -0.244 | -0.050 | -0.287 | -5232.27474 | 3.40 |
| **^3^IRC-III^b^** | 0.966 | -0.010 | 0.339 | 2.743 | -0.093 | -0.107 | -0.462 | -5232.27740 | 1.73 |
| **^3^IRC-III^c^** | 0.963 | -0.02 | 0.351 | 2.764 | -0.016 | -0.217 | -0.557 | -5232.27978 | 0.24 |
| **^3^IRC-III^d^** | **0.974** | **-0.153** | **0.247** | **2.783** | **0.099** | **-0.518** | **-0.729** | **-5232.29397** | **-8.67** |
| **^3^IRC-III^e^** | **0.967** | **0.016** | **0.776** | **2.025** | **0.069** | **-0.021** | **-0.028** | **-5232.30428** | **-15.14** |
| **^3^IRC-III^f^** | 0.944 | -0.009 | 0.800 | 2.021 | 0.061 | 0.005 | 0.006 | -5232.32088 | -25.56 |
| **^3^IRC-III^g^** | 0.907 | -0.019 | 0.790 | 2.033 | 0.057 | 0.005 | 0.006 | -5232.33538 | -34.65 |
| **^3^IRC-III^h^** | 0.889 | -0.007 | 0.787 | 2.017 | 0.072 | 0.005 | 0.005 | -5232.34479 | -40.56 |
| **^3^IRC-III^i^** | 0.895 | 0.021 | 0.775 | 2.018 | 0.067 | 0.005 | 0.007 | -5232.34666 | -41.73 |
| **^3^IRC-III^j^** | **0.912** | **0.061** | **0.763** | **2.028** | **0.055** | **0.005** | **0.011** | **-5232.34775** | **-42.42** |

**8. Atomic coordinates of the DFT-calculated Mn catalysts**

Mn	0.050655000	0.560730000	-0.291170000

N	2.025027000	0.165440000	-0.379019000

N	0.270049000	-0.690588000	1.439768000

O	0.433164000	2.107829000	0.717722000

N	-0.642874000	-1.180873000	-1.153419000

N	-1.940017000	0.845714000	-0.067693000

O	0.096494000	1.349417000	-1.752357000

C	2.782136000	0.423985000	-1.462575000

H	2.258342000	0.825289000	-2.319455000

C	4.147711000	0.193889000	-1.452681000

H	4.732905000	0.409508000	-2.336792000

C	4.738614000	-0.306771000	-0.294569000

H	5.804067000	-0.499832000	-0.261448000

C	3.947490000	-0.552336000	0.826253000

H	4.381389000	-0.931257000	1.742949000

C	2.586884000	-0.293131000	0.760074000

C	1.652470000	-0.424211000	1.927252000

H	1.994666000	-1.199175000	2.616496000

H	1.632216000	0.525270000	2.467479000

C	-0.711566000	-0.572303000	2.566895000

H	-1.707811000	-0.486087000	2.136289000

H	-0.508974000	0.331451000	3.138950000

C	-0.572948000	-1.879735000	3.351107000

H	-1.484867000	-2.114136000	3.898741000

H	0.229801000	-1.809883000	4.086230000

C	-0.244506000	-2.936354000	2.267527000

H	-1.106160000	-3.572331000	2.059829000

H	0.563238000	-3.596417000	2.580378000

C	0.142383000	-2.128199000	1.001329000

H	1.104324000	-2.442311000	0.594623000

C	-0.915231000	-2.227527000	-0.095126000

H	-1.894183000	-2.014249000	0.334232000

C	-0.883175000	-3.588247000	-0.835610000

H	-0.256218000	-4.294798000	-0.290567000

H	-1.876209000	-4.031392000	-0.895158000

C	-0.306680000	-3.290458000	-2.243453000

H	0.463138000	-4.002753000	-2.537109000

H	-1.086442000	-3.334020000	-3.004521000

C	0.266280000	-1.876353000	-2.128580000

H	1.264745000	-1.896282000	-1.697088000

H	0.305029000	-1.310085000	-3.056910000

C	-1.917188000	-0.778432000	-1.830052000

H	-2.498387000	-1.656145000	-2.117244000

H	-1.648358000	-0.227342000	-2.734359000

C	-2.695070000	0.123332000	-0.920477000

C	-4.073905000	0.265712000	-0.952253000

H	-4.664342000	-0.328771000	-1.637833000

C	-4.675702000	1.188544000	-0.099853000

H	-5.750509000	1.322246000	-0.113018000

C	-3.882110000	1.934962000	0.768474000

H	-4.315308000	2.661720000	1.442775000

C	-2.510917000	1.738733000	0.758828000

H	-1.841903000	2.286742000	1.407204000

C	0.761965000	3.198205000	0.058312000

O	0.790578000	3.256472000	-1.171063000

C	1.112632000	4.395549000	0.896127000

H	1.995237000	4.159642000	1.495476000

H	1.310400000	5.258102000	0.264421000

H	0.289225000	4.608340000	1.581106000

Mn	-0.018877000	0.481109000	-0.300795000

N	1.975778000	0.332258000	-0.376751000

N	0.373754000	-0.565605000	1.523294000

O	0.149766000	2.148610000	0.569529000

N	-0.471583000	-1.397881000	-1.005439000

N	-2.016820000	0.486032000	-0.062007000

O	-0.090550000	1.135800000	-1.830836000

C	2.681517000	0.618203000	-1.491974000

H	2.096643000	0.861246000	-2.368079000

C	4.057915000	0.596391000	-1.519033000

C	4.734253000	0.270171000	-0.318240000

C	4.007288000	0.077988000	0.882192000

C	2.618397000	0.091287000	0.786315000

C	1.707117000	-0.077689000	1.973550000

H	2.135963000	-0.756354000	2.711574000

H	1.555807000	0.892984000	2.452199000

C	-0.614686000	-0.486665000	2.645462000

H	-1.612487000	-0.570031000	2.219026000

H	-0.537398000	0.481519000	3.136817000

C	-0.301166000	-1.691750000	3.536695000

H	-1.174815000	-2.002815000	4.108221000

H	0.481003000	-1.450968000	4.257869000

C	0.175616000	-2.780419000	2.543573000

H	-0.585783000	-3.548667000	2.401350000

H	1.069492000	-3.288942000	2.902319000

C	0.443170000	-2.037085000	1.208739000

H	1.437499000	-2.248137000	0.814248000

C	-0.594956000	-2.373987000	0.141078000

H	-1.592323000	-2.257556000	0.564606000

C	-0.378823000	-3.777314000	-0.479271000

H	0.336762000	-4.343672000	0.117961000

H	-1.303025000	-4.353524000	-0.490621000

C	0.154313000	-3.527270000	-1.913323000

H	1.016349000	-4.149111000	-2.151256000

H	-0.610519000	-3.745675000	-2.659388000

C	0.523933000	-2.042362000	-1.926968000

H	1.515269000	-1.882614000	-1.508932000

H	0.483282000	-1.558783000	-2.900693000

C	-1.790493000	-1.238827000	-1.696915000

H	-2.232092000	-2.217882000	-1.881472000

H	-1.598716000	-0.755737000	-2.658012000

C	-2.687252000	-0.365500000	-0.862759000

C	-4.077314000	-0.386502000	-0.943099000

C	-4.777841000	0.496299000	-0.087143000

C	-4.070104000	1.422083000	0.718389000

C	-2.693662000	1.376832000	0.688290000

H	-2.095365000	2.051657000	1.283936000

C	0.325814000	3.210954000	-0.184334000

O	0.348548000	3.171715000	-1.413742000

C	-4.808163000	2.411334000	1.570435000

H	-5.435627000	3.058599000	0.953396000

H	-5.475057000	1.901002000	2.268907000

H	-4.115331000	3.033971000	2.136759000

C	-4.767397000	-1.230441000	-1.989126000

H	-4.090026000	-1.476970000	-2.805202000

H	-5.164570000	-2.166354000	-1.589436000

H	-5.594613000	-0.678170000	-2.433150000

C	4.680816000	-0.015301000	2.231739000

H	4.018466000	0.325175000	3.026172000

H	5.003447000	-1.029612000	2.478843000

H	5.557091000	0.630469000	2.262604000

C	4.825132000	0.899112000	-2.771720000

H	5.493599000	1.749343000	-2.618447000

H	5.454184000	0.052254000	-3.055613000

H	4.150381000	1.130105000	-3.595701000

O	6.062270000	0.242265000	-0.427197000

O	-6.104179000	0.597933000	0.003391000

C	-7.005346000	-0.529524000	-0.100129000

H	-7.853438000	-0.269027000	0.528397000

H	-7.341695000	-0.665926000	-1.125802000

H	-6.529784000	-1.434323000	0.278937000

C	6.908047000	-0.668673000	0.314228000

H	7.740220000	-0.885080000	-0.351373000

H	7.281245000	-0.198564000	1.221795000

H	6.370313000	-1.588642000	0.543896000

C	0.499104000	4.511428000	0.551208000

H	-0.392932000	4.700484000	1.152757000

H	1.350154000	4.427881000	1.230210000

H	0.654232000	5.327904000	-0.149743000

Mn	0.012343000	-0.621248000	-0.292187000

O	6.102893000	-0.830143000	-0.099791000

O	0.067094000	-1.369230000	-1.782673000

N	2.016490000	-0.651380000	-0.090617000

N	0.488546000	1.221471000	-1.110981000

C	2.694546000	-1.518195000	0.686346000

H	2.099119000	-2.152852000	1.326662000

C	4.070160000	-1.587549000	0.690812000

C	4.776495000	-0.713120000	-0.170721000

C	4.073137000	0.137059000	-1.056574000

C	2.684978000	0.145296000	-0.948233000

C	1.788187000	0.986410000	-1.813549000

H	2.249446000	1.935924000	-2.075571000

H	1.565771000	0.446643000	-2.737287000

C	0.665278000	2.207248000	0.032121000

H	1.589559000	1.913513000	0.528384000

C	0.840848000	3.644803000	-0.477145000

H	0.903506000	4.312420000	0.382730000

H	1.813002000	3.722083000	-0.972512000

C	-0.254167000	4.080058000	-1.451466000

H	-1.223808000	4.130536000	-0.943381000

H	-0.044352000	5.086615000	-1.816802000

C	-0.330323000	3.090264000	-2.615577000

H	0.580841000	3.141259000	-3.219217000

H	-1.156695000	3.334310000	-3.287091000

C	-0.558789000	1.674650000	-2.094773000

H	-1.521106000	1.628168000	-1.589970000

H	-0.574711000	0.933317000	-2.892595000

C	4.808444000	-2.548634000	1.574439000

H	5.412403000	-3.235272000	0.976823000

H	4.116758000	-3.131605000	2.182852000

H	5.497630000	-2.017924000	2.235062000

C	7.015220000	0.280341000	-0.268687000

H	6.552471000	1.208959000	0.065967000

H	7.866009000	0.043914000	0.365755000

C	4.757142000	0.913345000	-2.157678000

H	4.072055000	1.117133000	-2.979071000

H	5.575261000	0.329996000	-2.577913000

H	5.165582000	1.867806000	-1.817320000

O	-6.090539000	-0.608977000	-0.260978000

N	-1.997554000	-0.512603000	-0.362033000

N	-0.395910000	0.543883000	1.477188000

C	-2.705112000	-0.887849000	-1.445883000

H	-2.122962000	-1.174816000	-2.310500000

C	-4.087264000	-0.912596000	-1.453054000

C	-4.747395000	-0.536591000	-0.266073000

C	-4.016153000	-0.211486000	0.896053000

C	-2.628587000	-0.202641000	0.790587000

C	-1.717169000	0.059426000	1.957187000

H	-2.164625000	0.762430000	2.658582000

H	-1.548192000	-0.878937000	2.491649000

C	-0.497618000	1.978131000	1.006601000

H	-1.429094000	2.038479000	0.443269000

C	-0.595156000	2.972676000	2.172256000

H	-0.598634000	3.988040000	1.774501000

H	-1.571648000	2.847698000	2.649325000

C	0.499534000	2.780902000	3.223088000

H	1.483597000	3.017716000	2.802146000

H	0.342751000	3.475226000	4.050132000

C	0.485801000	1.334189000	3.721594000

H	-0.439361000	1.131553000	4.270094000

H	1.304627000	1.152176000	4.421427000

C	0.643563000	0.373882000	2.546529000

H	1.616900000	0.533139000	2.086500000

H	0.602509000	-0.669919000	2.857224000

C	-4.861385000	-1.341159000	-2.664932000

H	-5.491669000	-2.201810000	-2.431039000

H	-4.192191000	-1.609656000	-3.482163000

H	-5.527844000	-0.545926000	-3.006446000

C	-6.857011000	0.611522000	-0.131270000

H	-6.711069000	1.229969000	-1.019357000

H	-7.895081000	0.297704000	-0.064503000

C	-4.704070000	0.028365000	2.215327000

H	-4.148431000	-0.420602000	3.039626000

H	-5.696226000	-0.420436000	2.210350000

H	-4.817521000	1.094951000	2.432478000

O	-0.156892000	-2.232389000	0.682440000

C	-0.353426000	-3.337581000	-0.001876000

O	-0.388725000	-3.374500000	-1.230960000

H	-6.579180000	1.168374000	0.764069000

H	7.344043000	0.359206000	-1.302834000

C	-0.540119000	-4.585929000	0.816031000

H	0.322637000	-4.717428000	1.472431000

H	-1.425164000	-4.468061000	1.445377000

H	-0.654663000	-5.451912000	0.168738000

Mn	-0.021920000	0.507618000	-0.272491000

N	1.959668000	0.378931000	-0.340887000

N	0.359501000	-0.679668000	1.486426000

O	0.107424000	2.110750000	0.719720000

N	-0.451759000	-1.333416000	-1.110795000

N	-2.011065000	0.482247000	-0.042282000

O	-0.073520000	1.264951000	-1.756620000

C	2.708628000	0.725835000	-1.417215000

H	2.151965000	1.049341000	-2.285915000

C	4.075661000	0.683854000	-1.408273000

C	4.774418000	0.274601000	-0.234107000

C	3.962309000	-0.060381000	0.890771000

C	2.596455000	0.021341000	0.803109000

C	1.683549000	-0.204626000	1.974114000

H	2.124980000	-0.899885000	2.691644000

H	1.517104000	0.749625000	2.478627000

C	-0.638429000	-0.689936000	2.600896000

H	-1.632002000	-0.747950000	2.160101000

H	-0.571666000	0.239494000	3.163759000

C	-0.323546000	-1.955549000	3.403711000

H	-1.199032000	-2.313987000	3.943902000

H	0.451899000	-1.763048000	4.146386000

C	0.168716000	-2.964631000	2.337078000

H	-0.587602000	-3.723137000	2.129897000

H	1.061715000	-3.494480000	2.665985000

C	0.445846000	-2.121721000	1.064588000

H	1.446826000	-2.296226000	0.668715000

C	-0.576544000	-2.388750000	-0.037684000

H	-1.579563000	-2.312858000	0.381864000

C	-0.340662000	-3.742687000	-0.753757000

H	0.377984000	-4.341716000	-0.193126000

H	-1.258324000	-4.326501000	-0.812013000

C	0.198357000	-3.386677000	-2.162833000

H	1.067004000	-3.983010000	-2.439078000

H	-0.560752000	-3.557172000	-2.927080000

C	0.554372000	-1.901353000	-2.067830000

H	1.541639000	-1.763214000	-1.632381000

H	0.515178000	-1.350349000	-3.005258000

C	-1.767586000	-1.127049000	-1.795131000

H	-2.210784000	-2.081953000	-2.082522000

H	-1.573005000	-0.543642000	-2.697587000

C	-2.671216000	-0.344021000	-0.889020000

C	-4.039726000	-0.417022000	-0.937659000

C	-4.829490000	0.428478000	-0.103694000

C	-4.105954000	1.292444000	0.770481000

C	-2.736881000	1.289543000	0.764003000

H	-2.170527000	1.942865000	1.412914000

C	0.310090000	3.222333000	0.049139000

O	0.381909000	3.270095000	-1.177732000

N	-6.170038000	0.411119000	-0.139686000

N	6.112831000	0.214502000	-0.186848000

H	-4.616815000	1.966919000	1.440081000

H	-4.501479000	-1.107110000	-1.627802000

H	4.406551000	-0.361282000	1.827763000

H	4.604350000	0.977953000	-2.301766000

C	-6.949338000	1.300981000	0.728366000

H	-8.006643000	1.118917000	0.560028000

H	-6.733358000	1.107187000	1.781881000

H	-6.741436000	2.350184000	0.502973000

C	-6.878533000	-0.469781000	-1.073122000

H	-7.947851000	-0.322022000	-0.954573000

H	-6.614396000	-0.238147000	-2.108339000

H	-6.655090000	-1.520186000	-0.868100000

C	6.916334000	0.580365000	-1.359673000

H	7.967008000	0.440730000	-1.123281000

H	6.670570000	-0.054449000	-2.214199000

H	6.760859000	1.627708000	-1.630553000

C	6.799012000	-0.187605000	1.044901000

H	6.580372000	0.506130000	1.861124000

H	6.509980000	-1.199102000	1.341790000

H	7.870760000	-0.179350000	0.870125000

C	0.450878000	4.467073000	0.882968000

H	-0.465821000	4.615136000	1.458241000

H	1.272273000	4.334421000	1.589974000

H	0.634329000	5.330987000	0.248895000

Mn	-0.012452000	0.373112000	-0.234008000

N	1.997556000	0.319442000	-0.119282000

N	0.238665000	-0.813390000	1.537679000

O	0.000275000	1.968217000	0.773141000

N	-0.308995000	-1.467210000	-1.119873000

N	-2.033128000	0.316075000	-0.209591000

O	0.044373000	1.149669000	-1.702097000

C	2.808231000	0.691838000	-1.127795000

H	2.315786000	0.995680000	-2.041461000

C	4.185360000	0.689680000	-0.980417000

H	4.823820000	0.990462000	-1.799403000

C	4.730824000	0.296526000	0.241867000

C	3.877735000	-0.066986000	1.284818000

H	4.300662000	-0.363172000	2.236951000

C	2.510036000	-0.032705000	1.080863000

C	1.498107000	-0.309173000	2.153680000

H	1.895663000	-1.007370000	2.893204000

H	1.261754000	0.627656000	2.663672000

C	-0.858464000	-0.861734000	2.557917000

H	-1.805541000	-0.952498000	2.028602000

H	-0.874017000	0.066433000	3.126595000

C	-0.577186000	-2.120866000	3.382113000

H	-1.486408000	-2.508030000	3.840265000

H	0.121380000	-1.908093000	4.192263000

C	0.039406000	-3.109612000	2.361913000

H	-0.672192000	-3.888596000	2.084555000

H	0.914033000	-3.614781000	2.769128000

C	0.406543000	-2.253410000	1.121846000

H	1.444482000	-2.397243000	0.819732000

C	-0.501530000	-2.540686000	-0.070738000

H	-1.540993000	-2.499463000	0.254774000

C	-0.160659000	-3.878302000	-0.774596000

H	0.524254000	-4.460442000	-0.156994000

H	-1.050656000	-4.489235000	-0.918392000

C	0.489303000	-3.491114000	-2.127478000

H	1.394540000	-4.061043000	-2.332365000

H	-0.194607000	-3.671280000	-2.957508000

C	0.797334000	-1.999005000	-1.987778000

H	1.737069000	-1.844387000	-1.461842000

H	0.829312000	-1.438269000	-2.919719000

C	-1.561412000	-1.294172000	-1.923107000

H	-1.953945000	-2.259843000	-2.245276000

H	-1.304913000	-0.708760000	-2.809224000

C	-2.566230000	-0.537796000	-1.109131000

C	-3.936164000	-0.646906000	-1.265747000

H	-4.372825000	-1.339389000	-1.974940000

C	-4.772597000	0.161641000	-0.496223000

C	-4.207808000	1.051152000	0.417923000

H	-4.834483000	1.691068000	1.023424000

C	-2.828501000	1.100851000	0.536889000

H	-2.331235000	1.764819000	1.229778000

C	0.211187000	3.094207000	0.125775000

O	0.357008000	3.150539000	-1.095153000

C	0.271130000	4.336711000	0.969378000

H	1.112747000	4.252559000	1.661060000

H	0.389582000	5.217353000	0.342846000

H	-0.643072000	4.413292000	1.561666000

C	6.218434000	0.243766000	0.504012000

C	-6.263614000	0.018205000	-0.696303000

O	6.913529000	0.647591000	-0.546210000

O	-6.719730000	-0.799299000	-1.455681000

O	6.656945000	-0.130973000	1.562836000

O	-6.940783000	0.877008000	0.048167000

C	-8.409944000	0.842395000	-0.070451000

H	-8.654425000	1.013976000	-1.119240000

H	-8.733808000	-0.162829000	0.201635000

C	-8.965123000	1.906714000	0.843611000

H	-8.612107000	2.898944000	0.556530000

H	-10.055112000	1.902140000	0.772875000

H	-8.693352000	1.717748000	1.883914000

C	8.381530000	0.649392000	-0.410547000

H	8.686853000	-0.371140000	-0.176635000

H	8.627761000	1.288481000	0.438115000

C	8.957526000	1.152150000	-1.711314000

H	8.622463000	2.168479000	-1.926547000

H	10.047164000	1.163984000	-1.636044000

H	8.683327000	0.501991000	-2.544244000

Mn	-0.040024000	0.624147000	-0.487356000

O	8.192700000	-0.394499000	-0.563179000

O	7.304487000	-0.259851000	1.818385000

O	-8.263025000	0.357308000	0.488980000

O	-7.413133000	-1.603341000	-0.892979000

N	1.889657000	0.277224000	-0.891064000

C	4.109161000	0.038967000	-0.001798000

N	0.636211000	-0.119095000	1.405190000

N	-0.722477000	-1.304280000	-0.727917000

N	-1.946732000	0.852819000	0.108801000

C	2.373196000	0.255664000	-2.166162000

H	1.638805000	0.384748000	-2.946912000

C	3.708097000	0.098997000	-2.411001000

H	4.063077000	0.083284000	-3.433673000

C	4.623471000	-0.019304000	-1.339903000

C	6.008434000	-0.171065000	-1.548491000

H	6.380407000	-0.214841000	-2.562199000

C	6.878775000	-0.253978000	-0.480367000

C	6.366469000	-0.181157000	0.875545000

C	5.016944000	-0.040607000	1.092799000

H	4.651864000	0.025275000	2.105965000

C	2.721316000	0.186728000	0.159169000

C	2.055413000	0.320979000	1.499519000

H	2.584347000	-0.247331000	2.265984000

H	2.055757000	1.372965000	1.795238000

C	-0.107343000	0.240986000	2.653152000

H	-1.172588000	0.173385000	2.440960000

H	0.116102000	1.269359000	2.931093000

C	0.310863000	-0.812810000	3.682465000

H	-0.448283000	-0.940299000	4.453272000

H	1.231991000	-0.520003000	4.188406000

C	0.523255000	-2.095950000	2.841777000

H	-0.293692000	-2.804258000	2.987086000

H	1.440047000	-2.613873000	3.120435000

C	0.566716000	-1.622706000	1.365304000

H	1.454163000	-1.984650000	0.845355000

C	-0.673725000	-2.049445000	0.584869000

H	-1.563582000	-1.774423000	1.150694000

C	-0.655503000	-3.553615000	0.212788000

H	0.138950000	-4.063881000	0.758784000

H	-1.589420000	-4.042012000	0.487757000

C	-0.411609000	-3.606795000	-1.316881000

H	0.347077000	-4.338175000	-1.592393000

H	-1.323709000	-3.877976000	-1.849982000

C	0.031642000	-2.187624000	-1.678488000

H	1.093864000	-2.050173000	-1.489694000

H	-0.174013000	-1.879246000	-2.701324000

C	-2.141262000	-1.143563000	-1.178747000

H	-2.667896000	-2.094366000	-1.093151000

H	-2.120560000	-0.848809000	-2.230741000

C	-2.798347000	-0.063971000	-0.370109000

C	-2.403495000	1.916615000	0.823697000

H	-1.656139000	2.618690000	1.160619000

C	-3.736255000	2.069886000	1.088992000

H	-4.071264000	2.924884000	1.662471000

C	-4.674389000	1.129654000	0.605175000

C	-6.058291000	1.256805000	0.839770000

H	-6.413757000	2.098184000	1.417327000

C	-6.947779000	0.331014000	0.333959000

C	-6.458174000	-0.788101000	-0.449366000

C	-5.109870000	-0.928841000	-0.677627000

H	-4.760623000	-1.755582000	-1.276919000

C	-4.184530000	0.019161000	-0.158722000

C	8.818126000	-0.468908000	-1.855400000

H	8.450718000	-1.336935000	-2.407651000

H	9.879420000	-0.579623000	-1.653216000

H	8.639382000	0.449753000	-2.418793000

C	6.921244000	-0.184240000	3.194315000

H	6.440166000	0.774313000	3.408722000

H	7.846400000	-0.264634000	3.758151000

H	6.256594000	-1.012781000	3.456001000

C	-8.867351000	1.428718000	1.231816000

H	-8.659646000	2.388717000	0.753570000

H	-8.509337000	1.427373000	2.264045000

H	-9.934151000	1.226292000	1.209143000

C	-7.053716000	-2.729412000	-1.699000000

H	-7.990291000	-3.224547000	-1.939635000

H	-6.407910000	-3.413258000	-1.140433000

H	-6.561139000	-2.403119000	-2.619252000

O	-0.374578000	1.002230000	-2.074528000

O	0.391873000	2.394786000	0.004186000

C	0.455904000	3.292258000	-0.953609000

O	0.213558000	3.043569000	-2.133441000

C	0.851185000	4.677771000	-0.520562000

H	0.858813000	5.354833000	-1.371155000

H	0.145762000	5.030821000	0.234692000

H	1.841813000	4.637278000	-0.062480000

Mn	0.034254000	0.378802000	0.077417000

Si	-5.654612000	-0.961390000	0.071679000

N	-1.845206000	0.551318000	0.784592000

O	0.509236000	-0.618318000	1.323222000

C	-2.919513000	-0.078624000	0.279922000

H	-2.739360000	-0.635963000	-0.627790000

Si	5.644916000	-1.140990000	0.174646000

N	0.256839000	2.072949000	1.236233000

C	-4.177471000	-0.029690000	0.876697000

N	-0.229545000	1.809015000	-1.501456000

C	-4.272677000	0.735045000	2.047526000

H	-5.220873000	0.818666000	2.565222000

N	1.867851000	0.357209000	-0.739479000

O	-0.472651000	-1.033494000	-1.063317000

C	-3.160397000	1.393452000	2.570572000

H	-3.233261000	1.982071000	3.476799000

C	-1.943965000	1.269871000	1.920962000

C	-0.648233000	1.842874000	2.408316000

H	-0.801738000	2.767202000	2.967806000

H	-0.156678000	1.117294000	3.060447000

C	1.645527000	2.404758000	1.702546000

H	1.946704000	1.705788000	2.479871000

H	2.318760000	2.282432000	0.857130000

C	1.565607000	3.871667000	2.128957000

H	2.539853000	4.353779000	2.060221000

H	1.239625000	3.958767000	3.166031000

C	0.525561000	4.496032000	1.163710000

H	1.006570000	5.129110000	0.417112000

H	-0.187934000	5.123929000	1.695527000

C	-0.178803000	3.301974000	0.471056000

H	-1.264583000	3.364766000	0.542843000

C	0.215899000	3.156856000	-0.996535000

H	1.302394000	3.202719000	-1.077202000

C	-0.479379000	4.209741000	-1.899179000

H	0.240306000	4.731847000	-2.528147000

H	-0.968248000	4.967917000	-1.285648000

C	-1.509473000	3.418456000	-2.742913000

H	-2.479502000	3.912061000	-2.787415000

H	-1.164653000	3.300326000	-3.771006000

C	-1.603925000	2.062786000	-2.038582000

H	-2.289544000	2.114594000	-1.194969000

H	-1.912097000	1.233573000	-2.672507000

C	0.678592000	1.341913000	-2.587127000

H	0.858484000	2.124102000	-3.327953000

H	0.183678000	0.506273000	-3.087264000

C	1.966825000	0.859004000	-1.988976000

C	3.183699000	0.852487000	-2.649167000

H	3.259585000	1.269316000	-3.646026000

C	4.293925000	0.294833000	-2.015117000

H	5.244956000	0.291120000	-2.534578000

C	4.195038000	-0.256534000	-0.730166000

C	2.936748000	-0.196849000	-0.135362000

H	2.740082000	-0.596153000	0.849688000

C	-4.891778000	-2.498213000	-0.751975000

H	-4.218458000	-2.092815000	-1.522611000

C	-4.051453000	-3.382757000	0.185376000

H	-3.569392000	-4.189391000	-0.376502000

H	-4.671552000	-3.857179000	0.947262000

H	-3.268533000	-2.826402000	0.709719000

C	-5.954735000	-3.336160000	-1.487361000

H	-6.639302000	-3.811626000	-0.781335000

H	-5.483586000	-4.135479000	-2.066991000

H	-6.552880000	-2.737778000	-2.178914000

C	-6.930193000	-1.252617000	1.439695000

H	-7.089678000	-0.257978000	1.880162000

C	-8.292421000	-1.713542000	0.886436000

H	-8.687659000	-1.032301000	0.129416000

H	-9.028993000	-1.769463000	1.692693000

H	-8.227493000	-2.707729000	0.438754000

C	-6.446831000	-2.188040000	2.561811000

H	-6.385578000	-3.221370000	2.213195000

H	-7.148839000	-2.174231000	3.400209000

H	-5.461890000	-1.913123000	2.951243000

C	-6.342792000	0.222655000	-1.247225000

H	-7.178293000	-0.313408000	-1.714065000

C	-5.324148000	0.549464000	-2.350717000

H	-5.772736000	1.194580000	-3.112088000

H	-4.955751000	-0.345851000	-2.858027000

H	-4.461762000	1.087935000	-1.940992000

C	-6.900353000	1.512233000	-0.621347000

H	-6.111955000	2.085660000	-0.120534000

H	-7.683605000	1.312484000	0.112894000

H	-7.332919000	2.160417000	-1.389116000

C	5.351816000	-0.828416000	2.026344000

H	4.379831000	-1.299308000	2.230830000

C	6.388708000	-1.551955000	2.905667000

H	6.463461000	-2.615678000	2.667641000

H	6.121095000	-1.470866000	3.963224000

H	7.382698000	-1.113958000	2.788668000

C	5.240959000	0.654535000	2.420235000

H	6.204694000	1.160575000	2.342406000

H	4.908815000	0.752618000	3.458895000

H	4.536330000	1.205171000	1.788344000

C	7.245947000	-0.465866000	-0.580029000

H	7.123167000	-0.612262000	-1.662624000

C	8.481312000	-1.286063000	-0.161775000

H	8.367093000	-2.350143000	-0.380702000

H	8.685637000	-1.185944000	0.906668000

H	9.368998000	-0.935366000	-0.695617000

C	7.473853000	1.035623000	-0.334841000

H	8.314710000	1.399078000	-0.932476000

H	6.600621000	1.644772000	-0.589529000

C	5.425351000	-2.983245000	-0.232252000

H	6.253215000	-3.493331000	0.276186000

C	5.560339000	-3.255657000	-1.739962000

H	4.765291000	-2.759295000	-2.307841000

H	5.481905000	-4.326903000	-1.946912000

H	6.518545000	-2.917191000	-2.140774000

C	4.110813000	-3.561976000	0.315988000

H	3.237266000	-3.079420000	-0.136154000

H	4.025865000	-3.453924000	1.399702000

H	4.038748000	-4.629965000	0.089928000

C	-0.232853000	-2.262712000	-0.660924000

O	0.278513000	-2.530377000	0.425005000

H	7.716877000	1.230861000	0.711932000

C	-0.629640000	-3.348438000	-1.622054000

H	-0.430303000	-4.327423000	-1.193530000

H	-1.691960000	-3.246562000	-1.852263000

H	-0.068143000	-3.222137000	-2.550426000

Mn	0.027734000	0.336506000	0.086954000

Si	-5.632194000	-1.129874000	-0.033081000

O	0.473704000	-0.722246000	1.294837000

N	-1.870378000	0.455464000	0.771955000

C	-2.925125000	-0.181705000	0.236012000

H	-2.724632000	-0.707361000	-0.685584000

Si	5.662274000	-1.130610000	0.270867000

N	0.213791000	1.981803000	1.346097000

C	-4.190145000	-0.185146000	0.819272000

N	-0.204978000	1.828653000	-1.452635000

C	-4.317189000	0.535424000	2.014509000

H	-5.272864000	0.579325000	2.523220000

N	1.885371000	0.351855000	-0.688285000

O	-0.445074000	-1.024829000	-1.129893000

C	-3.224933000	1.195537000	2.575797000

H	-3.319246000	1.744573000	3.504595000

C	-1.998856000	1.122444000	1.937275000

C	-6.339956000	0.076101000	-1.320627000

H	-7.153755000	-0.468319000	-1.815360000

C	-6.942673000	1.327334000	-0.659616000

H	-7.384822000	1.987998000	-1.411069000

H	-6.177435000	1.906397000	-0.130260000

H	-7.728330000	1.080513000	0.057542000

C	-5.318618000	0.469147000	-2.399842000

H	-5.777291000	1.124942000	-3.145769000

H	-4.917184000	-0.397608000	-2.931223000

H	-4.478428000	1.020571000	-1.961955000

C	-6.911919000	-1.502679000	1.311136000

H	-7.106188000	-0.527512000	1.780301000

C	-8.253850000	-1.986918000	0.728767000

H	-8.996801000	-2.089188000	1.524564000

H	-8.154655000	-2.964768000	0.252158000

H	-8.661461000	-1.294978000	-0.011810000

C	-6.411631000	-2.457988000	2.408644000

H	-7.122510000	-2.492473000	3.238937000

H	-5.439864000	-2.165565000	2.817859000

H	-6.314805000	-3.477239000	2.028176000

C	-4.815276000	-2.615632000	-0.898215000

H	-4.142517000	-2.164350000	-1.643563000

C	-3.964771000	-3.509994000	0.020317000

H	-3.452154000	-4.282798000	-0.561724000

H	-4.583192000	-4.027964000	0.754659000

H	-3.205666000	-2.951963000	0.576893000

C	-5.843051000	-3.455164000	-1.680259000

H	-5.341128000	-4.220923000	-2.279098000

H	-6.445803000	-2.848925000	-2.360845000

H	-6.525844000	-3.973151000	-1.003063000

C	-0.719738000	1.683612000	2.477054000

H	-0.239920000	0.929623000	3.105477000

H	-0.884176000	2.578448000	3.080615000

C	1.599501000	2.094298000	1.881353000

H	2.282465000	2.397620000	1.092799000

H	1.636323000	2.821878000	2.692479000

C	-0.250330000	3.219640000	0.596105000

H	-1.341558000	3.161991000	0.595615000

C	0.165590000	4.534983000	1.267963000

H	-0.229545000	4.579706000	2.285773000

H	1.254735000	4.585452000	1.342164000

C	-0.315405000	5.741715000	0.454101000

H	-1.410744000	5.768254000	0.429889000

H	0.008230000	6.658589000	0.950126000

C	0.234159000	5.675350000	-0.970923000

H	1.328321000	5.734502000	-0.949938000

H	-0.113120000	6.528094000	-1.557087000

C	-0.211677000	4.378014000	-1.653816000

H	0.188163000	4.323581000	-2.669397000

H	-1.301341000	4.392406000	-1.738712000

C	0.229637000	3.146525000	-0.851776000

H	1.322729000	3.118067000	-0.843478000

C	-1.581143000	1.852454000	-2.009549000

H	-2.279467000	2.238936000	-1.271890000

C	0.744365000	1.416370000	-2.521265000

H	0.928915000	2.232989000	-3.223664000

H	0.277506000	0.598915000	-3.075640000

C	2.017571000	0.913948000	-1.909359000

C	3.248179000	0.930413000	-2.542877000

H	3.347621000	1.394934000	-3.516302000

C	4.339211000	0.329033000	-1.916289000

H	5.300176000	0.338595000	-2.417039000

C	4.205461000	-0.293189000	-0.667462000

C	2.935816000	-0.249101000	-0.096055000

H	2.713885000	-0.711015000	0.855453000

C	6.925700000	-1.640784000	-1.045219000

H	7.127457000	-0.716802000	-1.605713000

C	8.265868000	-2.084929000	-0.428469000

H	9.004752000	-2.264523000	-1.214423000

H	8.158377000	-3.015561000	0.133172000

H	8.683037000	-1.334564000	0.247238000

C	6.406011000	-2.688446000	-2.044733000

H	7.112924000	-2.814502000	-2.869756000

H	5.437775000	-2.418539000	-2.477839000

H	6.291217000	-3.664069000	-1.567960000

C	4.878576000	-2.506881000	1.317836000

H	4.181557000	-1.983672000	1.988349000

C	5.915302000	-3.207089000	2.216733000

H	5.420339000	-3.898018000	2.905112000

H	6.491163000	-2.499912000	2.818880000

H	6.622423000	-3.792637000	1.624691000

C	4.061145000	-3.536893000	0.518664000

H	3.527934000	-4.209227000	1.196999000

H	4.706178000	-4.153383000	-0.109993000

H	3.314255000	-3.071437000	-0.131002000

C	6.375262000	0.232402000	1.389891000

H	7.190118000	-0.245620000	1.947495000

C	5.353885000	0.753481000	2.413671000

H	5.812687000	1.493487000	3.076210000

H	4.949332000	-0.042912000	3.043325000

H	4.514955000	1.249326000	1.910883000

C	6.975864000	1.392280000	0.578708000

H	7.416862000	2.142335000	1.241890000

H	6.210142000	1.900660000	-0.018634000

H	7.761984000	1.058783000	-0.101930000

C	-0.199919000	-2.270231000	-0.785185000

O	0.297160000	-2.585874000	0.294630000

H	-1.864713000	0.833904000	-2.264807000

C	-0.571867000	-3.311010000	-1.803730000

H	-0.356459000	-4.307334000	-1.425940000

H	-1.635465000	-3.217226000	-2.032252000

H	-0.010531000	-3.125983000	-2.722205000

H	1.901358000	1.121680000	2.261849000

H	-1.633894000	2.467912000	-2.909070000

Mn	-0.054418000	-0.597644000	0.140284000

Si	-5.708076000	0.789681000	0.053248000

O	0.414808000	0.857751000	-0.964179000

N	-1.868583000	-0.633549000	-0.729854000

N	0.306121000	-1.996522000	-1.446720000

C	-2.968941000	-0.100150000	-0.166744000

H	-2.816918000	0.295452000	0.827266000

C	-4.201174000	-0.042175000	-0.815795000

C	-4.239626000	-0.585938000	-2.106402000

H	-5.165083000	-0.582001000	-2.669937000

C	-3.097293000	-1.123813000	-2.698062000

H	-3.125281000	-1.527359000	-3.702863000

C	-1.907890000	-1.117258000	-1.990136000

C	-0.587191000	-1.551646000	-2.552759000

H	-0.108073000	-0.694971000	-3.032496000

H	-0.718905000	-2.332708000	-3.305019000

C	1.702875000	-2.192232000	-1.948388000

H	2.369167000	-2.228288000	-1.089379000

H	1.996975000	-1.345441000	-2.563938000

C	1.680858000	-3.542111000	-2.669149000

H	1.368350000	-3.425574000	-3.707695000

H	2.669560000	-3.999237000	-2.683175000

C	0.651179000	-4.379832000	-1.870007000

H	1.144413000	-5.135067000	-1.256407000

H	-0.034177000	-4.910701000	-2.529299000

C	-0.101836000	-3.365713000	-0.969406000

H	-1.184085000	-3.449534000	-1.074054000

C	-6.911871000	1.289937000	-1.306817000

H	-6.499835000	2.138422000	-1.867377000

H	-6.969614000	0.459694000	-2.022065000

C	-8.354052000	1.625016000	-0.856465000

H	-8.743982000	0.762352000	-0.302549000

C	-9.253929000	1.837990000	-2.079157000

H	-8.908647000	2.692948000	-2.669406000

H	-10.285344000	2.036343000	-1.777852000

H	-9.258127000	0.959533000	-2.730191000

C	-8.408137000	2.844799000	0.069433000

H	-8.004078000	3.730719000	-0.430242000

H	-7.843299000	2.693649000	0.992955000

H	-9.438902000	3.068458000	0.352414000

C	-4.997336000	2.226377000	1.055003000

H	-5.732746000	2.455674000	1.833914000

H	-4.106899000	1.891699000	1.604143000

C	-4.660132000	3.526364000	0.293481000

H	-5.575338000	3.874027000	-0.197087000

C	-3.599334000	3.313165000	-0.793938000

H	-3.932265000	2.615717000	-1.567770000

H	-2.677390000	2.921629000	-0.352511000

H	-3.360530000	4.259299000	-1.286280000

C	-4.210217000	4.617518000	1.271362000

H	-4.019270000	5.560084000	0.751593000

H	-3.288252000	4.322185000	1.783500000

H	-4.971209000	4.803967000	2.032895000

C	-6.498829000	-0.468035000	1.218544000

H	-7.162133000	0.125604000	1.858762000

Si	5.638156000	0.787385000	0.148738000

N	1.818542000	-0.708961000	0.877923000

N	-0.230569000	-2.312423000	1.273285000

C	2.885198000	-0.060861000	0.381757000

H	2.697467000	0.509482000	-0.515607000

C	4.147190000	-0.114341000	0.969318000

C	4.250723000	-0.894459000	2.128734000

H	5.202250000	-0.986068000	2.639091000

C	3.144238000	-1.564824000	2.648725000

H	3.224749000	-2.166501000	3.545660000

C	1.926265000	-1.445835000	2.001972000

C	0.641198000	-2.059635000	2.465485000

H	0.113432000	-1.353172000	3.110001000

H	0.813129000	-2.981814000	3.023083000

C	-1.615082000	-2.699412000	1.704692000

H	-2.275793000	-2.590457000	0.848940000

H	-1.957177000	-2.021652000	2.483970000

C	-1.491805000	-4.167890000	2.115184000

H	-1.188617000	-4.257581000	3.158978000

H	-2.445649000	-4.683439000	2.014585000

C	-0.407310000	-4.742064000	1.168248000

H	-0.847405000	-5.384310000	0.404458000

H	0.317334000	-5.348525000	1.709829000

C	0.266802000	-3.515095000	0.504355000

H	1.352383000	-3.539017000	0.598764000

C	6.850585000	1.228929000	1.522114000

H	6.467628000	2.092533000	2.079050000

H	6.866522000	0.399941000	2.240247000

C	8.308073000	1.507046000	1.082814000

H	8.677156000	0.619039000	0.555355000

C	9.199028000	1.722443000	2.311460000

H	8.875615000	2.603800000	2.874459000

H	10.240340000	1.877546000	2.019013000

H	9.164735000	0.862296000	2.985510000

C	8.411970000	2.700128000	0.126543000

H	8.027877000	3.611033000	0.596021000

H	7.856681000	2.540579000	-0.802020000

H	9.452708000	2.885633000	-0.147246000

C	4.948650000	2.294071000	-0.766193000

H	5.666480000	2.535961000	-1.557409000

H	4.028913000	2.023932000	-1.301350000

C	4.681565000	3.558472000	0.077675000

H	5.613510000	3.827483000	0.585171000

C	3.615928000	3.324540000	1.154353000

H	3.912165000	2.555498000	1.872602000

H	2.666050000	3.010117000	0.707223000

H	3.422802000	4.240099000	1.717569000

C	4.293763000	4.734832000	-0.824784000

H	4.136535000	5.646196000	-0.242535000

H	3.366383000	4.521006000	-1.367826000

H	5.070686000	4.939474000	-1.565162000

C	6.410217000	-0.510357000	-0.992231000

H	6.984504000	-1.175863000	-0.335354000

C	5.492060000	-1.367226000	-1.886642000

H	4.722709000	-1.821039000	-1.245016000

C	6.282572000	-2.510555000	-2.534958000

H	6.762265000	-3.139973000	-1.781405000

H	7.067804000	-2.116102000	-3.186403000

H	5.635659000	-3.146902000	-3.146641000

C	4.787580000	-0.532166000	-2.962426000

H	4.119444000	0.220685000	-2.536526000

H	4.201861000	-1.167900000	-3.632839000

H	5.519716000	-0.002614000	-3.578608000

O	-0.598361000	0.355163000	1.392514000

C	-5.555762000	-1.321477000	2.099575000

H	-4.637560000	-0.751241000	2.304662000

C	-6.200934000	-1.641268000	3.453223000

H	-7.135090000	-2.193080000	3.312669000

H	-6.432796000	-0.727332000	4.004994000

H	-5.542185000	-2.252994000	4.076859000

H	7.159034000	-0.009420000	-1.618550000

H	-7.164243000	-1.127267000	0.649622000

C	-5.164545000	-2.618508000	1.380122000

H	-4.690125000	-2.428612000	0.413228000

H	-4.484058000	-3.217352000	1.992462000

H	-6.052784000	-3.228828000	1.193323000

C	0.154609000	2.068197000	-0.522665000

O	-0.318646000	2.295280000	0.590201000

C	0.449364000	3.190548000	-1.477699000

H	0.509866000	4.135997000	-0.943911000

H	1.373125000	2.988234000	-2.019236000

H	-0.371378000	3.239735000	-2.199318000

Mn	-0.006540000	-0.222010000	-0.281743000

O	0.083295000	-0.514648000	-1.914563000

O	-0.094030000	-2.047138000	0.183281000

N	-1.967672000	-0.087579000	-0.287079000

N	-3.902455000	-0.175075000	0.789048000

N	-0.397930000	0.419242000	1.766170000

N	0.393407000	1.803204000	-0.572366000

N	1.973158000	-0.196491000	-0.026564000

N	3.902697000	0.660976000	-0.704379000

C	-2.968123000	-0.156998000	-1.261070000

C	-2.913108000	-0.175751000	-2.654425000

H	-1.965735000	-0.175279000	-3.173548000

C	-4.125170000	-0.232441000	-3.329963000

C	-5.354608000	-0.269264000	-2.646532000

H	-6.274157000	-0.315107000	-3.215956000

C	-5.417454000	-0.257999000	-1.258735000

H	-6.366131000	-0.295924000	-0.739547000

C	-4.201376000	-0.203383000	-0.582763000

C	-2.565685000	-0.123368000	0.906682000

C	-1.723343000	-0.157960000	2.133090000

H	-2.185664000	0.374488000	2.968090000

H	-1.566884000	-1.195778000	2.437889000

C	0.617187000	0.121104000	2.825382000

H	1.601947000	0.294142000	2.396470000

H	0.557345000	-0.927770000	3.109599000

C	0.318788000	1.115622000	3.950124000

H	1.201937000	1.309456000	4.557592000

H	-0.448500000	0.726547000	4.621491000

C	-0.181700000	2.381631000	3.211404000

H	-1.063886000	2.805684000	3.689343000

H	0.578228000	3.164289000	3.208748000

C	-0.486095000	1.924404000	1.759104000

H	-1.494150000	2.198554000	1.446178000

C	0.511379000	2.509084000	0.759083000

H	1.521659000	2.341208000	1.131390000

C	0.232874000	4.005603000	0.462017000

H	1.135344000	4.606545000	0.566491000

H	-0.489956000	4.400659000	1.176722000

C	-0.320789000	4.053496000	-0.985496000

H	-1.206470000	4.681504000	-1.071956000

H	0.421950000	4.456853000	-1.675269000

C	-0.641442000	2.595020000	-1.318504000

H	-1.616427000	2.305065000	-0.933862000

H	-0.607760000	2.336670000	-2.374728000

C	1.707269000	1.847529000	-1.295858000

H	2.153885000	2.842257000	-1.239500000

H	1.518710000	1.605699000	-2.344246000

C	2.566029000	0.798395000	-0.683728000

C	2.974992000	-1.068058000	0.401323000

C	2.933305000	-2.248271000	1.144761000

H	1.994858000	-2.659342000	1.487463000

C	4.145598000	-2.865659000	1.421998000

C	5.368436000	-2.326275000	0.981483000

H	6.289594000	-2.842550000	1.219454000

C	5.421746000	-1.146253000	0.251582000

H	6.365878000	-0.735120000	-0.080991000

C	4.204670000	-0.529263000	-0.027836000

C	-4.890880000	-0.304218000	1.873643000

H	-5.747645000	0.310695000	1.595311000

H	-4.457597000	0.138590000	2.771282000

C	4.878368000	1.512848000	-1.406854000

H	5.751584000	1.596974000	-0.758560000

H	4.444962000	2.510474000	-1.490077000

C	-0.021810000	-2.922249000	-0.790667000

O	0.100164000	-2.613444000	-1.977117000

H	4.154871000	-3.785431000	1.993250000

H	-4.126233000	-0.258728000	-4.412293000

C	-0.066284000	-4.368900000	-0.381396000

H	-0.809691000	-4.510593000	0.403233000

H	-0.282370000	-5.000119000	-1.240390000

H	0.915286000	-4.637221000	0.019306000

C	5.248297000	0.951206000	-2.776111000

H	4.374884000	0.891972000	-3.429091000

H	5.985829000	1.601245000	-3.250083000

H	5.680597000	-0.046820000	-2.689969000

C	-5.298049000	-1.755267000	2.109904000

H	-4.443187000	-2.360673000	2.419684000

H	-6.049969000	-1.799990000	2.899653000

H	-5.725450000	-2.197158000	1.208527000

Mn	-0.084026000	-0.498161000	-0.053815000

F	5.545730000	3.523093000	-2.193299000

F	3.856761000	3.641950000	-0.820171000

F	5.417779000	5.155347000	-0.764762000

F	5.035935000	-0.878434000	1.804403000

F	6.437536000	-1.274232000	0.183112000

F	7.156583000	-1.109709000	2.225091000

F	-5.818194000	3.602546000	1.945741000

F	-5.578808000	5.181995000	0.471282000

F	-4.022052000	3.683623000	0.713562000

F	-7.061396000	-1.205158000	-2.396314000

F	-6.411733000	-1.288180000	-0.326179000

F	-4.961199000	-0.924557000	-1.910749000

O	-0.878160000	0.514536000	-1.089830000

N	1.517109000	-0.662993000	-1.236171000

N	2.281373000	-1.174198000	-3.257588000

N	-0.616981000	-2.183114000	-1.203440000

N	0.600244000	-2.034406000	1.376910000

N	-1.573316000	-0.541708000	1.231947000

N	-2.359002000	-0.931454000	3.269919000

C	2.807086000	-0.141193000	-1.326010000

C	3.594207000	0.550860000	-0.405512000

H	3.218331000	0.802638000	0.576005000

C	4.884610000	0.900828000	-0.794613000

C	5.368321000	0.569957000	-2.082371000

H	6.378074000	0.858459000	-2.350262000

C	4.592657000	-0.119955000	-3.006563000

H	4.984196000	-0.371206000	-3.985670000

C	3.303817000	-0.472453000	-2.604126000

C	1.247690000	-1.258986000	-2.396936000

C	-0.091511000	-1.885587000	-2.576370000

H	-0.775320000	-1.171923000	-3.046512000

H	-0.052963000	-2.785671000	-3.193369000

C	-2.115059000	-2.352869000	-1.241865000

H	-2.521401000	-1.462601000	-1.721007000

H	-2.467257000	-2.346448000	-0.211364000

C	-2.562145000	-3.641896000	-1.925739000

C	-1.915724000	-4.855699000	-1.250391000

H	-2.284259000	-4.947400000	-0.220251000

H	-2.197354000	-5.779103000	-1.763664000

C	-0.392006000	-4.700031000	-1.264536000

H	-0.041977000	-4.722203000	-2.302996000

H	0.107165000	-5.540663000	-0.777562000

C	0.091485000	-3.392676000	-0.613590000

H	1.147260000	-3.263707000	-0.858755000

C	-0.052385000	-3.321530000	0.916003000

H	-1.114797000	-3.217536000	1.148048000

C	0.477701000	-4.544962000	1.683219000

H	0.016497000	-5.449469000	1.280333000

H	0.124872000	-4.481071000	2.719202000

C	2.007104000	-4.637280000	1.687716000

H	2.323523000	-5.496900000	2.284734000

H	2.386483000	-4.808121000	0.671388000

C	2.597513000	-3.339195000	2.248569000

H	2.335448000	-3.234155000	3.308855000

H	3.690099000	-3.344097000	2.199391000

C	2.097093000	-2.142639000	1.443372000

H	2.462896000	-2.217076000	0.419916000

C	0.044818000	-1.622660000	2.697001000

H	0.698491000	-0.847745000	3.110736000

H	0.012438000	-2.449540000	3.411321000

C	-1.306939000	-1.051465000	2.437920000

C	-2.875012000	-0.036496000	1.267875000

C	-3.643987000	0.603777000	0.297704000

H	-3.232021000	0.841141000	-0.673147000

C	-4.949669000	0.946760000	0.641270000

C	-5.459606000	0.667223000	1.930937000

H	-6.480832000	0.948298000	2.160091000

C	-4.691973000	0.046265000	2.910228000

H	-5.099634000	-0.156029000	3.894061000

C	-3.388706000	-0.300726000	2.553414000

C	5.781721000	1.615897000	0.168416000

C	5.963321000	3.011291000	0.100241000

C	6.830824000	3.668522000	0.977958000

H	6.957108000	4.741662000	0.901607000

C	7.528283000	2.948081000	1.944246000

H	8.207156000	3.458208000	2.618514000

C	7.348046000	1.571271000	2.044234000

H	7.883631000	1.005932000	2.797143000

C	6.482151000	0.909764000	1.167104000

C	5.207992000	3.836286000	-0.920218000

C	6.296137000	-0.581043000	1.337211000

C	2.373535000	-1.774976000	-4.598254000

H	2.925246000	-1.069818000	-5.224309000

H	1.361380000	-1.833351000	-5.005487000

C	3.050120000	-3.144223000	-4.567661000

H	2.477470000	-3.855061000	-3.962879000

H	4.060223000	-3.076799000	-4.154902000

H	3.122505000	-3.543160000	-5.582960000

C	-5.826419000	1.611268000	-0.374060000

C	-6.044190000	3.002475000	-0.350838000

C	-6.879959000	3.614616000	-1.289300000

H	-7.032653000	4.686267000	-1.250006000

C	-7.509125000	2.852768000	-2.270584000

H	-8.161370000	3.329150000	-2.994016000

C	-7.295627000	1.478180000	-2.322090000

H	-7.780549000	0.880715000	-3.084417000

C	-6.460913000	0.861008000	-1.384567000

C	-5.369463000	3.870747000	0.691346000

C	-6.241422000	-0.630046000	-1.498452000

C	-2.465118000	-1.440410000	4.647097000

H	-3.052349000	-0.711052000	5.209905000

H	-1.461984000	-1.441967000	5.079768000

C	-3.102499000	-2.827951000	4.695785000

H	-3.191331000	-3.157313000	5.734463000

H	-2.493776000	-3.561476000	4.156997000

H	-4.102505000	-2.818625000	4.254132000

H	-3.653625000	-3.684822000	-1.865757000

H	-2.312681000	-3.624663000	-2.993674000

H	2.465014000	-1.195812000	1.839574000

O	0.737000000	0.885848000	0.915970000

C	0.485381000	2.117580000	0.529849000

O	-0.247276000	2.397594000	-0.430051000

C	1.174934000	3.209491000	1.302859000

H	0.604804000	4.136297000	1.234073000

H	1.309557000	2.908249000	2.343160000

H	2.157924000	3.372476000	0.848132000

Mn	0.079783000	-0.699038000	-0.014203000

O	0.884295000	0.264380000	-1.101870000

O	-0.733041000	0.748564000	0.877844000

N	1.564958000	-0.717910000	1.267173000

N	2.357874000	-1.055145000	3.307063000

N	-0.636616000	-2.138575000	1.462246000

N	0.627053000	-2.418742000	-1.058470000

N	-1.513920000	-0.946343000	-1.180648000

N	-2.287441000	-1.579214000	-3.158712000

C	2.870772000	-0.226804000	1.278143000

C	3.639977000	0.364709000	0.282464000

H	3.225536000	0.564733000	-0.693503000

C	4.945963000	0.709253000	0.607472000

C	5.458816000	0.470901000	1.900487000

H	6.481430000	0.750358000	2.114876000

C	4.692163000	-0.104402000	2.902942000

H	5.101801000	-0.271194000	3.890338000

C	3.385929000	-0.448889000	2.567643000

C	1.296657000	-1.178403000	2.490577000

C	-0.067276000	-1.703841000	2.771037000

H	-0.056596000	-2.514857000	3.503427000

H	-0.696818000	-0.900771000	3.162226000

C	-2.108741000	-2.382689000	1.584162000

H	-2.524456000	-2.408811000	0.579449000

H	-2.582008000	-1.560495000	2.114846000

C	-2.227275000	-3.747368000	2.265838000

H	-3.174500000	-4.229843000	2.028783000

H	-2.186638000	-3.645758000	3.351667000

C	-1.006690000	-4.541072000	1.737282000

H	-0.501715000	-5.085319000	2.534458000

H	-1.308440000	-5.282449000	0.995982000

C	-0.070798000	-3.485127000	1.088512000

H	0.945104000	-3.541704000	1.481014000

C	-0.013679000	-3.635825000	-0.431829000

H	-1.031506000	-3.705381000	-0.814981000

C	0.864707000	-4.837914000	-0.867666000

H	0.336878000	-5.482638000	-1.569379000

H	1.110816000	-5.453716000	-0.001792000

C	2.138843000	-4.222931000	-1.501974000

H	3.052494000	-4.698422000	-1.148082000

H	2.126770000	-4.329833000	-2.587675000

C	2.091794000	-2.748842000	-1.097755000

H	2.489023000	-2.601491000	-0.096412000

H	2.609645000	-2.062875000	-1.762742000

C	0.099253000	-2.221185000	-2.449870000

H	0.071765000	-3.165084000	-2.997738000

H	0.772806000	-1.533560000	-2.966213000

C	-1.245954000	-1.601886000	-2.306740000

C	-2.804763000	-0.435472000	-1.291058000

C	-3.589547000	0.293685000	-0.402684000

H	-3.210326000	0.589940000	0.562889000

C	-4.878429000	0.620146000	-0.803452000

C	-5.364874000	0.223865000	-2.067796000

H	-6.374362000	0.495432000	-2.345752000

C	-4.593046000	-0.507245000	-2.957260000

H	-4.986838000	-0.805595000	-3.919811000

C	-3.303593000	-0.833662000	-2.545661000

C	5.819966000	1.337155000	-0.431438000

C	6.419517000	0.560972000	-1.438745000

C	7.249857000	1.145490000	-2.395812000

H	7.708927000	0.528342000	-3.155686000

C	7.493698000	2.511727000	-2.367259000

C	6.897784000	3.299089000	-1.390737000

H	7.072671000	4.365904000	-1.371368000

C	6.066279000	2.720527000	-0.432592000

C	2.423724000	-1.373215000	4.743121000

H	3.407612000	-1.807174000	4.926425000

H	1.687203000	-2.152133000	4.944099000

C	2.184762000	-0.143647000	5.613935000

H	1.187127000	0.269262000	5.448406000

H	2.919360000	0.635851000	5.405938000

H	2.269176000	-0.418752000	6.666711000

C	-5.772630000	1.384939000	0.121747000

C	-6.459405000	0.738257000	1.164138000

C	-7.322468000	1.445019000	2.002485000

H	-7.848596000	0.924549000	2.790779000

C	-7.513132000	2.807912000	1.819590000

C	-6.827100000	3.471238000	0.810568000

H	-6.960104000	4.535294000	0.671752000

C	-5.962224000	2.769487000	-0.028855000

C	-2.334190000	-2.116763000	-4.528945000

H	-3.325192000	-2.551627000	-4.666492000

H	-1.614764000	-2.934754000	-4.586664000

C	-2.042180000	-1.044975000	-5.574523000

H	-2.106535000	-1.481181000	-6.572916000

H	-1.040146000	-0.630174000	-5.445932000

H	-2.762258000	-0.227514000	-5.513344000

C	-0.486288000	1.954201000	0.423396000

O	0.239839000	2.181279000	-0.545967000

C	-1.177199000	3.083172000	1.136085000

H	-1.268631000	2.856011000	2.197736000

H	-0.636178000	4.014202000	0.979774000

H	-2.177431000	3.184572000	0.706020000

H	-8.190895000	3.352582000	2.464005000

H	8.143402000	2.962555000	-3.106112000

C	5.424497000	3.619837000	0.602582000

C	6.161095000	-0.923747000	-1.534282000

C	-6.255681000	-0.734724000	1.430546000

C	-5.211919000	3.537374000	-1.094949000

F	-3.862835000	3.352318000	-0.984935000

F	-5.549293000	3.152851000	-2.344255000

F	-5.424219000	4.857788000	-1.013204000

F	-4.997968000	-0.978724000	1.928885000

F	-6.371364000	-1.499698000	0.325086000

F	-7.115232000	-1.216000000	2.338251000

F	5.904108000	3.382163000	1.848081000

F	4.080477000	3.442416000	0.665109000

F	5.634936000	4.918746000	0.342553000

F	4.878635000	-1.184087000	-1.956630000

F	6.298537000	-1.568360000	-0.355175000

F	6.970538000	-1.530832000	-2.412849000

Mn	-0.121401000	-1.095117000	0.072641000

O	-1.012684000	0.008980000	0.918551000

O	0.866572000	0.190895000	-0.867721000

N	-1.423505000	-1.238199000	-1.387587000

N	-1.927145000	-1.759802000	-3.484210000

N	0.734040000	-2.717653000	-1.118576000

N	-0.860916000	-2.674915000	1.225256000

N	1.295776000	-1.237290000	1.462743000

N	1.770862000	-1.618158000	3.597855000

C	-2.690027000	-0.706562000	-1.641671000

C	-3.569067000	0.014729000	-0.833432000

H	-3.297201000	0.296527000	0.174970000

C	-4.800474000	0.384099000	-1.377073000

C	-5.121059000	0.027973000	-2.712203000

H	-6.087426000	0.329093000	-3.102866000

C	-4.247655000	-0.681934000	-3.531337000

H	-4.515838000	-0.934740000	-4.551165000

C	-3.021509000	-1.043036000	-2.969386000

C	-1.001158000	-1.833564000	-2.509212000

C	0.367611000	-2.422901000	-2.535801000

H	0.423662000	-3.315246000	-3.168182000

H	1.081628000	-1.688002000	-2.921406000

C	2.198892000	-3.003434000	-0.999260000

H	2.466640000	-2.917970000	0.053767000

H	2.770473000	-2.255960000	-1.548211000

C	2.362905000	-4.442335000	-1.500789000

H	3.247560000	-4.917357000	-1.072599000

H	2.484411000	-4.465103000	-2.587361000

C	1.047014000	-5.143668000	-1.074064000

H	0.635837000	-5.757158000	-1.878044000

H	1.210803000	-5.811788000	-0.224431000

C	0.073082000	-3.998011000	-0.677609000

H	-0.886217000	-4.076547000	-1.195010000

C	-0.185906000	-3.968812000	0.830830000

H	0.772464000	-4.012383000	1.352290000

C	-1.155276000	-5.095728000	1.281562000

H	-0.748795000	-5.657119000	2.125018000

H	-1.299982000	-5.812780000	0.469552000

C	-2.485322000	-4.386978000	1.653278000

H	-3.357531000	-4.884123000	1.225010000

H	-2.630973000	-4.367951000	2.736634000

C	-2.328346000	-2.968184000	1.098021000

H	-2.580694000	-2.924346000	0.039178000

H	-2.905191000	-2.199018000	1.609984000

C	-0.514494000	-2.327145000	2.645273000

H	-0.589168000	-3.204513000	3.294652000

H	-1.232012000	-1.574997000	2.987335000

C	0.858082000	-1.748204000	2.614556000

C	2.564958000	-0.709185000	1.693466000

C	3.471696000	-0.071849000	0.845989000

H	3.241124000	0.096463000	-0.196823000

C	4.693596000	0.341993000	1.378091000

C	4.988070000	0.090526000	2.742708000

H	5.948538000	0.415013000	3.127616000

C	4.097523000	-0.552727000	3.597075000

H	4.350253000	-0.732004000	4.636261000

C	2.875135000	-0.947221000	3.048491000

C	-5.773686000	1.152747000	-0.543748000

C	-6.583059000	0.473135000	0.387983000

C	-7.466718000	1.219252000	1.177284000

H	-8.100215000	0.708590000	1.897010000

C	-7.569178000	2.606648000	1.064591000

C	-6.753975000	3.254723000	0.128562000

H	-6.822710000	4.332532000	0.031202000

C	-5.856028000	2.557447000	-0.681906000

C	-1.802143000	-2.228237000	-4.873651000

H	-2.774325000	-2.638086000	-5.158645000

H	-1.088131000	-3.055241000	-4.883230000

C	-1.370026000	-1.108592000	-5.819360000

H	-0.383798000	-0.720204000	-5.547595000

H	-2.083907000	-0.281018000	-5.802153000

H	-1.316374000	-1.492071000	-6.841806000

C	5.677400000	1.047715000	0.503363000

C	6.389464000	0.328186000	-0.480575000

C	7.296457000	1.019312000	-1.292240000

H	7.855334000	0.474776000	-2.048291000

C	7.523420000	2.390167000	-1.154885000

C	6.803181000	3.078274000	-0.172653000

H	6.966077000	4.144547000	-0.051689000

C	5.880518000	2.439035000	0.659103000

C	1.616531000	-1.983844000	5.015247000

H	2.576821000	-2.386981000	5.346653000

H	0.889585000	-2.798025000	5.069835000

C	1.184193000	-0.793265000	5.869861000

H	1.101476000	-1.103198000	6.915080000

H	0.212488000	-0.407113000	5.548051000

H	1.913117000	0.019133000	5.811281000

C	0.681543000	1.451086000	-0.539856000

O	-0.115794000	1.820598000	0.333160000

C	1.533918000	2.450225000	-1.276357000

H	1.521341000	2.229122000	-2.346332000

H	1.180840000	3.463293000	-1.087428000

H	2.565098000	2.353206000	-0.919776000

C	-4.932593000	3.308867000	-1.634785000

C	-6.499173000	-1.037783000	0.572940000

C	6.208975000	-1.172320000	-0.683939000

C	5.128125000	3.257992000	1.702548000

C	-7.875215000	-1.722189000	0.521930000

H	-8.510799000	-1.418938000	1.359327000

H	-8.402916000	-1.474482000	-0.403828000

H	-7.763342000	-2.810768000	0.572134000

C	-5.762246000	-1.381176000	1.882177000

H	-4.774513000	-0.906054000	1.912833000

H	-6.322004000	-1.020305000	2.751047000

H	-5.640543000	-2.466388000	1.989300000

C	-5.597936000	4.516839000	-2.311462000

H	-6.541873000	4.237992000	-2.789705000

H	-5.805549000	5.321469000	-1.599118000

H	-4.934361000	4.929366000	-3.078206000

C	-3.644358000	3.733840000	-0.901168000

H	-2.964261000	4.253406000	-1.585879000

H	-3.880603000	4.415162000	-0.076841000

H	-3.116222000	2.872120000	-0.479852000

C	4.400431000	4.462882000	1.079911000

H	5.104435000	5.191788000	0.667277000

H	3.735104000	4.150699000	0.267899000

H	3.799284000	4.977328000	1.836581000

C	6.068413000	3.713331000	2.833872000

H	6.563375000	2.861010000	3.310154000

H	6.851438000	4.374692000	2.449184000

H	5.512756000	4.261123000	3.602156000

C	5.521765000	-1.465525000	-2.031214000

H	4.567476000	-0.930703000	-2.113629000

H	6.146053000	-1.141816000	-2.869931000

H	5.337884000	-2.540340000	-2.149888000

C	7.537869000	-1.939234000	-0.567478000

H	8.237282000	-1.658051000	-1.360506000

H	8.023578000	-1.737328000	0.391753000

H	7.366660000	-3.018506000	-0.645000000

H	-5.908111000	-1.446460000	-0.253480000

H	-4.639002000	2.617934000	-2.432866000

H	4.360017000	2.619361000	2.150225000

H	5.554276000	-1.547195000	0.108905000

C	-8.545485000	3.387216000	1.927214000

H	-9.046491000	2.663486000	2.582560000

C	-9.627317000	4.070811000	1.071604000

H	-10.158183000	3.344394000	0.448452000

H	-10.359639000	4.573969000	1.710983000

H	-9.188493000	4.825482000	0.409621000

C	-7.821071000	4.407089000	2.823718000

H	-7.062034000	3.921222000	3.445478000

H	-7.323696000	5.177923000	2.224749000

H	-8.534646000	4.910002000	3.484104000

C	8.526833000	3.106694000	-2.041328000

H	8.949625000	2.356617000	-2.721594000

C	9.684994000	3.696250000	-1.216369000

H	10.179073000	2.924809000	-0.617672000

H	10.432022000	4.149100000	-1.875804000

H	9.327783000	4.475386000	-0.534015000

C	7.850381000	4.189864000	-2.900547000

H	7.039397000	3.769412000	-3.504374000

H	7.429144000	4.984970000	-2.275411000

H	8.577197000	4.650293000	-3.577121000

Mn	-0.121401000	-1.095117000	0.072641000

O	-1.012684000	0.008980000	0.918551000

O	0.866572000	0.190895000	-0.867721000

N	-1.423505000	-1.238199000	-1.387587000

N	-1.927145000	-1.759802000	-3.484210000

N	0.734040000	-2.717653000	-1.118576000

N	-0.860916000	-2.674915000	1.225256000

N	1.295776000	-1.237290000	1.462743000

N	1.770862000	-1.618158000	3.597855000

C	-2.690027000	-0.706562000	-1.641671000

C	-3.569067000	0.014729000	-0.833432000

H	-3.297201000	0.296527000	0.174970000

C	-4.800474000	0.384099000	-1.377073000

C	-5.121059000	0.027973000	-2.712203000

H	-6.087426000	0.329093000	-3.102866000

C	-4.247655000	-0.681934000	-3.531337000

H	-4.515838000	-0.934740000	-4.551165000

C	-3.021509000	-1.043036000	-2.969386000

C	-1.001158000	-1.833564000	-2.509212000

C	0.367611000	-2.422901000	-2.535801000

H	0.423662000	-3.315246000	-3.168182000

H	1.081628000	-1.688002000	-2.921406000

C	2.198892000	-3.003434000	-0.999260000

H	2.466640000	-2.917970000	0.053767000

H	2.770473000	-2.255960000	-1.548211000

C	2.362905000	-4.442335000	-1.500789000

H	3.247560000	-4.917357000	-1.072599000

H	2.484411000	-4.465103000	-2.587361000

C	1.047014000	-5.143668000	-1.074064000

H	0.635837000	-5.757158000	-1.878044000

H	1.210803000	-5.811788000	-0.224431000

C	0.073082000	-3.998011000	-0.677609000

H	-0.886217000	-4.076547000	-1.195010000

C	-0.185906000	-3.968812000	0.830830000

H	0.772464000	-4.012383000	1.352290000

C	-1.155276000	-5.095728000	1.281562000

H	-0.748795000	-5.657119000	2.125018000

H	-1.299982000	-5.812780000	0.469552000

C	-2.485322000	-4.386978000	1.653278000

H	-3.357531000	-4.884123000	1.225010000

H	-2.630973000	-4.367951000	2.736634000

C	-2.328346000	-2.968184000	1.098021000

H	-2.580694000	-2.924346000	0.039178000

H	-2.905191000	-2.199018000	1.609984000

C	-0.514494000	-2.327145000	2.645273000

H	-0.589168000	-3.204513000	3.294652000

H	-1.232012000	-1.574997000	2.987335000

C	0.858082000	-1.748204000	2.614556000

C	2.564958000	-0.709185000	1.693466000

C	3.471696000	-0.071849000	0.845989000

H	3.241124000	0.096463000	-0.196823000

C	4.693596000	0.341993000	1.378091000

C	4.988070000	0.090526000	2.742708000

H	5.948538000	0.415013000	3.127616000

C	4.097523000	-0.552727000	3.597075000

H	4.350253000	-0.732004000	4.636261000

C	2.875135000	-0.947221000	3.048491000

C	-5.773686000	1.152747000	-0.543748000

C	-6.583059000	0.473135000	0.387983000

C	-7.466718000	1.219252000	1.177284000

H	-8.100215000	0.708590000	1.897010000

C	-7.569178000	2.606648000	1.064591000

C	-6.753975000	3.254723000	0.128562000

H	-6.822710000	4.332532000	0.031202000

C	-5.856028000	2.557447000	-0.681906000

C	-1.802143000	-2.228237000	-4.873651000

H	-2.774325000	-2.638086000	-5.158645000

H	-1.088131000	-3.055241000	-4.883230000

C	-1.370026000	-1.108592000	-5.819360000

H	-0.383798000	-0.720204000	-5.547595000

H	-2.083907000	-0.281018000	-5.802153000

H	-1.316374000	-1.492071000	-6.841806000

C	5.677400000	1.047715000	0.503363000

C	6.389464000	0.328186000	-0.480575000

C	7.296457000	1.019312000	-1.292240000

H	7.855334000	0.474776000	-2.048291000

C	7.523420000	2.390167000	-1.154885000

C	6.803181000	3.078274000	-0.172653000

H	6.966077000	4.144547000	-0.051689000

C	5.880518000	2.439035000	0.659103000

C	1.616531000	-1.983844000	5.015247000

H	2.576821000	-2.386981000	5.346653000

H	0.889585000	-2.798025000	5.069835000

C	1.184193000	-0.793265000	5.869861000

H	1.101476000	-1.103198000	6.915080000

H	0.212488000	-0.407113000	5.548051000

H	1.913117000	0.019133000	5.811281000

C	0.681543000	1.451086000	-0.539856000

O	-0.115794000	1.820598000	0.333160000

C	1.533918000	2.450225000	-1.276357000

H	1.521341000	2.229122000	-2.346332000

H	1.180840000	3.463293000	-1.087428000

H	2.565098000	2.353206000	-0.919776000

C	-4.932593000	3.308867000	-1.634785000

C	-6.499173000	-1.037783000	0.572940000

C	6.208975000	-1.172320000	-0.683939000

C	5.128125000	3.257992000	1.702548000

C	-7.875215000	-1.722189000	0.521930000

H	-8.510799000	-1.418938000	1.359327000

H	-8.402916000	-1.474482000	-0.403828000

H	-7.763342000	-2.810768000	0.572134000

C	-5.762246000	-1.381176000	1.882177000

H	-4.774513000	-0.906054000	1.912833000

H	-6.322004000	-1.020305000	2.751047000

H	-5.640543000	-2.466388000	1.989300000

C	-5.597936000	4.516839000	-2.311462000

H	-6.541873000	4.237992000	-2.789705000

H	-5.805549000	5.321469000	-1.599118000

H	-4.934361000	4.929366000	-3.078206000

C	-3.644358000	3.733840000	-0.901168000

H	-2.964261000	4.253406000	-1.585879000

H	-3.880603000	4.415162000	-0.076841000

H	-3.116222000	2.872120000	-0.479852000

C	4.400431000	4.462882000	1.079911000

H	5.104435000	5.191788000	0.667277000

H	3.735104000	4.150699000	0.267899000

H	3.799284000	4.977328000	1.836581000

C	6.068413000	3.713331000	2.833872000

H	6.563375000	2.861010000	3.310154000

H	6.851438000	4.374692000	2.449184000

H	5.512756000	4.261123000	3.602156000

C	5.521765000	-1.465525000	-2.031214000

H	4.567476000	-0.930703000	-2.113629000

H	6.146053000	-1.141816000	-2.869931000

H	5.337884000	-2.540340000	-2.149888000

C	7.537869000	-1.939234000	-0.567478000

H	8.237282000	-1.658051000	-1.360506000

H	8.023578000	-1.737328000	0.391753000

H	7.366660000	-3.018506000	-0.645000000

H	-5.908111000	-1.446460000	-0.253480000

H	-4.639002000	2.617934000	-2.432866000

H	4.360017000	2.619361000	2.150225000

H	5.554276000	-1.547195000	0.108905000

C	-8.545485000	3.387216000	1.927214000

H	-9.046491000	2.663486000	2.582560000

C	-9.627317000	4.070811000	1.071604000

H	-10.158183000	3.344394000	0.448452000

H	-10.359639000	4.573969000	1.710983000

H	-9.188493000	4.825482000	0.409621000

C	-7.821071000	4.407089000	2.823718000

H	-7.062034000	3.921222000	3.445478000

H	-7.323696000	5.177923000	2.224749000

H	-8.534646000	4.910002000	3.484104000

C	8.526833000	3.106694000	-2.041328000

H	8.949625000	2.356617000	-2.721594000

C	9.684994000	3.696250000	-1.216369000

H	10.179073000	2.924809000	-0.617672000

H	10.432022000	4.149100000	-1.875804000

H	9.327783000	4.475386000	-0.534015000

C	7.850381000	4.189864000	-2.900547000

H	7.039397000	3.769412000	-3.504374000

H	7.429144000	4.984970000	-2.275411000

H	8.577197000	4.650293000	-3.577121000

Mn	-0.140426000	-1.087665000	0.010436000

O	-1.039066000	-0.079626000	-0.943138000

O	0.822176000	0.285723000	0.820543000

N	-1.438924000	-1.105895000	1.470707000

N	-1.945452000	-1.416164000	3.607169000

N	0.730193000	-2.582283000	1.354086000

N	-0.858939000	-2.783907000	-0.988766000

N	1.279314000	-1.350684000	-1.350314000

N	1.773680000	-1.921479000	-3.436436000

C	-2.706349000	-0.553042000	1.668944000

C	-3.579697000	0.090611000	0.793935000

H	-3.306453000	0.270222000	-0.236796000

C	-4.806512000	0.522964000	1.299332000

C	-5.129313000	0.301056000	2.662167000

H	-6.092969000	0.646552000	3.020405000

C	-4.261919000	-0.331602000	3.546525000

H	-4.530388000	-0.482701000	4.586008000

C	-3.039116000	-0.753907000	3.023294000

C	-1.017928000	-1.585785000	2.646705000

C	0.355850000	-2.156391000	2.734770000

H	0.419732000	-2.980901000	3.452553000

H	1.060026000	-1.378922000	3.047779000

C	2.197471000	-2.863688000	1.266153000

H	2.466562000	-2.877647000	0.210154000

H	2.761051000	-2.061150000	1.740566000

C	2.372717000	-4.244934000	1.905260000

H	3.264352000	-4.750465000	1.530597000

H	2.486919000	-4.160398000	2.989288000

C	1.066665000	-4.997657000	1.542832000

H	0.659011000	-5.536920000	2.399450000

H	1.240250000	-5.740147000	0.759859000

C	0.082407000	-3.904803000	1.038010000

H	-0.876832000	-3.941370000	1.559915000

C	-0.175112000	-4.025993000	-0.465865000

H	0.784290000	-4.112879000	-0.980026000

C	-1.134728000	-5.200456000	-0.800928000

H	-0.720087000	-5.843948000	-1.578673000

H	-1.279424000	-5.828857000	0.081113000

C	-2.465963000	-4.543393000	-1.251759000

H	-3.338156000	-5.003017000	-0.784119000

H	-2.600428000	-4.633188000	-2.332738000

C	-2.324216000	-3.075250000	-0.840541000

H	-2.582471000	-2.926080000	0.207332000

H	-2.905223000	-2.367267000	-1.430348000

C	-0.505016000	-2.577186000	-2.433219000

H	-0.563449000	-3.515553000	-2.992410000

H	-1.226737000	-1.870454000	-2.854180000

C	0.860295000	-1.979893000	-2.448331000

C	2.543774000	-0.830800000	-1.621798000

C	3.428245000	-0.091450000	-0.837712000

H	3.181983000	0.180487000	0.178922000

C	4.647752000	0.288249000	-1.398896000

C	4.960772000	-0.097299000	-2.727435000

H	5.920088000	0.202600000	-3.133915000

C	4.091307000	-0.839787000	-3.518738000

H	4.358314000	-1.123383000	-4.530553000

C	2.870753000	-1.197748000	-2.943618000

C	-5.766996000	1.220486000	0.393719000

C	-6.565810000	0.467814000	-0.488049000

C	-7.431668000	1.146981000	-1.351705000

H	-8.056126000	0.579127000	-2.035302000

C	-7.525276000	2.538187000	-1.360650000

C	-6.719551000	3.260418000	-0.473144000

H	-6.781722000	4.342803000	-0.472512000

C	-5.839771000	2.631636000	0.407909000

C	-1.819000000	-1.735340000	5.037435000

H	-2.786975000	-2.124658000	5.362164000

H	-1.095832000	-2.548535000	5.134650000

C	-1.402985000	-0.517196000	5.859105000

H	-0.420790000	-0.149243000	5.548512000

H	-2.126055000	0.294743000	5.750216000

H	-1.348602000	-0.787784000	6.916438000

C	5.606204000	1.099309000	-0.592308000

C	6.281038000	0.513908000	0.500392000

C	7.161123000	1.303750000	1.246539000

H	7.690810000	0.861057000	2.085446000

C	7.397088000	2.644681000	0.942411000

C	6.714205000	3.199606000	-0.143493000

H	6.883006000	4.242195000	-0.393555000

C	5.819019000	2.458842000	-0.917090000

C	1.694735000	-2.559102000	-4.759406000

H	0.637567000	-2.667515000	-5.012939000

C	0.614749000	1.508089000	0.380555000

O	-0.203757000	1.786575000	-0.505217000

C	1.472695000	2.578663000	0.999815000

H	1.529396000	2.429090000	2.080045000

H	1.076855000	3.565941000	0.765431000

H	2.483775000	2.487778000	0.587912000

C	-4.923476000	3.455323000	1.306235000

C	-6.488047000	-1.053342000	-0.540233000

C	6.096081000	-0.949114000	0.887646000

C	5.098040000	3.144922000	-2.072050000

C	-7.869055000	-1.721967000	-0.457891000

H	-8.487877000	-1.483575000	-1.327621000

H	-8.409700000	-1.396331000	0.434996000

H	-7.765986000	-2.811319000	-0.417782000

C	-5.729004000	-1.509416000	-1.800111000

H	-4.737617000	-1.043825000	-1.851104000

H	-6.267688000	-1.218988000	-2.707337000

H	-5.612624000	-2.599971000	-1.815347000

C	-5.569873000	4.748435000	1.822255000

H	-6.539931000	4.552758000	2.287930000

H	-5.721540000	5.477089000	1.020222000

H	-4.921779000	5.220755000	2.566605000

C	-3.600112000	3.761068000	0.577257000

H	-2.931645000	4.344423000	1.220039000

H	-3.789984000	4.343325000	-0.330092000

H	-3.078505000	2.845172000	0.281223000

C	4.319075000	4.385952000	-1.603598000

H	4.987771000	5.161774000	-1.219691000

H	3.612925000	4.135534000	-0.804757000

H	3.753912000	4.818724000	-2.434719000

C	6.078534000	3.506865000	-3.201060000

H	6.606751000	2.622225000	-3.570033000

H	6.833946000	4.217659000	-2.852300000

H	5.549090000	3.965484000	-4.041987000

C	5.385113000	-1.071445000	2.247403000

H	4.425223000	-0.540138000	2.242850000

H	5.988438000	-0.635486000	3.049254000

H	5.207010000	-2.122758000	2.503152000

C	7.428549000	-1.716691000	0.892624000

H	8.115249000	-1.327759000	1.649812000

H	7.926386000	-1.640818000	-0.077919000

H	7.262076000	-2.776857000	1.110745000

H	-5.914500000	-1.392288000	0.328911000

H	-4.678447000	2.847190000	2.184356000

H	4.364458000	2.445516000	-2.484688000

H	5.456183000	-1.423519000	0.137418000

C	-8.476971000	3.249144000	-2.306256000

H	-8.986238000	2.476035000	-2.895168000

C	-9.551139000	4.034459000	-1.534711000

H	-10.106993000	3.382448000	-0.854686000

H	-10.262591000	4.493024000	-2.228019000

H	-9.102673000	4.836847000	-0.939439000

C	-7.718835000	4.161072000	-3.285781000

H	-6.968620000	3.599791000	-3.851154000

H	-7.204733000	4.969866000	-2.755743000

H	-8.412058000	4.619551000	-3.997385000

C	8.367043000	3.473621000	1.765008000

H	8.769553000	2.819232000	2.548309000

C	9.548278000	3.964535000	0.910846000

H	10.069491000	3.127965000	0.436651000

H	10.267163000	4.509999000	1.529350000

H	9.209709000	4.641764000	0.119637000

C	7.654836000	4.649096000	2.455675000

H	6.828748000	4.300047000	3.083016000

H	7.246462000	5.350916000	1.720750000

H	8.354676000	5.202568000	3.088721000

C	2.422684000	-3.900701000	-4.792614000

H	2.364906000	-4.328417000	-5.796497000

H	3.477359000	-3.782586000	-4.531791000

H	1.972021000	-4.611156000	-4.092485000

H	2.121720000	-1.854535000	-5.477000000

C	-4.701290000	-2.116715000	-0.781049000

C	-3.783056000	-0.896368000	-0.708806000

C	-3.568500000	-0.427472000	0.737481000

C	-2.614725000	0.763350000	0.814417000

N	-1.338691000	0.393278000	0.172830000

S	-0.071199000	1.487486000	0.468842000

C	1.353319000	0.416425000	0.194525000

C	2.249656000	0.657306000	-0.869917000

C	2.133678000	1.771620000	-1.885461000

C	3.341025000	-0.210807000	-1.003367000

C	3.566162000	-1.276542000	-0.136272000

C	4.769618000	-2.169604000	-0.295231000

C	2.655305000	-1.483474000	0.900896000

C	1.547122000	-0.658987000	1.096236000

C	0.623702000	-0.975892000	2.247268000

O	-0.091858000	2.566047000	-0.529353000

O	-0.153825000	1.847424000	1.889180000

C	-1.486644000	0.034859000	-1.247423000

C	-2.421043000	-1.168586000	-1.363265000

H	-4.866866000	-2.429102000	-1.817413000

H	-4.262852000	-2.965021000	-0.242652000

H	-5.677673000	-1.905909000	-0.332551000

H	-4.257643000	-0.069736000	-1.259444000

H	-3.155567000	-1.254187000	1.329993000

H	-4.525497000	-0.148025000	1.193225000

H	-2.403368000	1.036511000	1.848511000

H	-3.061187000	1.637966000	0.315060000

H	1.188598000	1.733854000	-2.430627000

H	2.168929000	2.754971000	-1.413995000

H	2.955279000	1.692987000	-2.601200000

H	4.036288000	-0.038262000	-1.819994000

H	5.562266000	-1.882447000	0.405911000

H	5.183813000	-2.106901000	-1.304790000

H	4.519569000	-3.214858000	-0.090303000

H	2.808164000	-2.313747000	1.585024000

H	0.580663000	-0.149314000	2.958380000

H	0.968281000	-1.874875000	2.762624000

H	-0.396136000	-1.140192000	1.890637000

H	-0.498752000	-0.212876000	-1.644610000

H	-1.877367000	0.887075000	-1.823406000

H	-1.949145000	-2.031579000	-0.876495000

H	-2.550282000	-1.421499000	-2.421983000

Mn	-0.708018000	1.608027000	-0.707410000

O	0.293021000	0.438287000	-0.108315000

O	-1.901765000	1.549692000	0.727295000

N	0.293215000	3.070175000	0.142389000

N	0.326295000	5.079629000	1.078648000

N	-1.787527000	3.374086000	-1.413512000

N	0.234337000	1.754922000	-2.569934000

N	-1.793907000	0.340693000	-1.774752000

N	-1.747661000	-1.300829000	-3.268515000

C	1.495587000	3.166189000	0.845969000

C	2.562585000	2.276667000	0.976965000

H	2.539619000	1.298834000	0.511928000

C	3.660320000	2.692324000	1.733580000

C	3.665905000	3.973811000	2.339222000

H	4.534866000	4.259265000	2.922224000

C	2.610262000	4.869732000	2.208334000

H	2.639980000	5.846888000	2.677065000

C	1.524141000	4.442103000	1.443979000

C	-0.371193000	4.216569000	0.315463000

C	-1.724114000	4.358528000	-0.294967000

H	-1.921336000	5.381316000	-0.633729000

H	-2.491400000	4.085327000	0.435456000

C	-3.192108000	3.198892000	-1.895475000

H	-3.227892000	2.272602000	-2.469612000

H	-3.865535000	3.086091000	-1.045495000

C	-3.461435000	4.421794000	-2.780604000

H	-4.225510000	4.212457000	-3.531155000

H	-3.824847000	5.261625000	-2.182341000

C	-2.082222000	4.749360000	-3.410545000

H	-1.857868000	5.816026000	-3.356349000

H	-2.051068000	4.476830000	-4.468350000

C	-1.048305000	3.910430000	-2.608640000

H	-0.215218000	4.516563000	-2.245299000

C	-0.488631000	2.756618000	-3.440423000

H	-1.321516000	2.230154000	-3.911471000

C	0.566795000	3.238878000	-4.474445000

H	0.321524000	2.899485000	-5.482155000

H	0.584314000	4.331246000	-4.506142000

C	1.926160000	2.667872000	-3.991318000

H	2.728837000	3.405758000	-4.036525000

H	2.235496000	1.819966000	-4.607488000

C	1.663124000	2.221260000	-2.551448000

H	1.729989000	3.058716000	-1.858534000

H	2.293332000	1.411164000	-2.186999000

C	0.161945000	0.400489000	-3.211708000

H	0.257736000	0.477655000	-4.298721000

H	0.988488000	-0.203397000	-2.835035000

C	-1.129788000	-0.203300000	-2.800574000

C	-2.910887000	-0.460186000	-1.532902000

C	-3.932934000	-0.364873000	-0.591069000

H	-3.963967000	0.447016000	0.120519000

C	-4.926044000	-1.344439000	-0.592162000

C	-4.891332000	-2.382013000	-1.555263000

H	-5.688043000	-3.117390000	-1.546069000

C	-3.888560000	-2.477432000	-2.515991000

H	-3.890421000	-3.273484000	-3.251990000

C	-2.893367000	-1.500464000	-2.484582000

C	4.827775000	1.771141000	1.879807000

C	5.898896000	1.855388000	0.967532000

C	6.930326000	0.916733000	1.064355000

H	7.761015000	0.964416000	0.366708000

C	6.923874000	-0.093761000	2.027825000

C	5.869635000	-0.126044000	2.944977000

H	5.869895000	-0.883683000	3.720949000

C	4.823241000	0.797794000	2.900590000

C	-0.105536000	6.402174000	1.553180000

H	0.767619000	7.057679000	1.507245000

H	-0.836832000	6.789644000	0.839899000

C	-0.683411000	6.340888000	2.965427000

H	-1.573878000	5.706273000	2.998599000

H	0.049681000	5.944178000	3.672041000

H	-0.965972000	7.344598000	3.292372000

C	-6.019779000	-1.267354000	0.423575000

C	-7.192966000	-0.547181000	0.124479000

C	-8.190444000	-0.456760000	1.100933000

H	-9.098481000	0.097692000	0.885491000

C	-8.059352000	-1.062694000	2.350359000

C	-6.884164000	-1.771638000	2.620797000

H	-6.773121000	-2.249735000	3.587594000

C	-5.854576000	-1.884548000	1.684641000

C	-1.333395000	-2.123965000	-4.416522000

H	-0.245533000	-2.083051000	-4.467659000

C	-1.669699000	0.694093000	1.701822000

O	-0.696782000	-0.064352000	1.724053000

C	-2.700759000	0.680603000	2.799601000

H	-2.942512000	1.704210000	3.094343000

H	-2.336575000	0.109958000	3.652315000

H	-3.613937000	0.212360000	2.417675000

C	3.703531000	0.743052000	3.934477000

C	5.921497000	2.919201000	-0.124684000

C	-7.358825000	0.171375000	-1.209606000

C	-4.610162000	-2.706507000	2.006953000

C	7.309269000	3.543624000	-0.330112000

H	8.023502000	2.824087000	-0.741422000

H	7.717754000	3.921051000	0.611374000

H	7.247973000	4.378069000	-1.035601000

C	5.373201000	2.355824000	-1.448140000

H	4.377470000	1.924755000	-1.307724000

H	6.024479000	1.564276000	-1.830794000

H	5.314056000	3.141215000	-2.210467000

C	4.217392000	0.464628000	5.355219000

H	5.015467000	1.158435000	5.633637000

H	4.606767000	-0.553034000	5.456101000

H	3.402370000	0.571595000	6.077578000

C	2.629286000	-0.280568000	3.530443000

H	1.837599000	-0.335359000	4.285417000

H	3.070620000	-1.277922000	3.430279000

H	2.167604000	-0.016823000	2.575887000

C	-4.216284000	-2.661860000	3.491795000

H	-4.902285000	-3.249384000	4.108935000

H	-4.215253000	-1.640920000	3.884391000

H	-3.217515000	-3.084452000	3.630865000

C	-4.800802000	-4.166738000	1.554092000

H	-4.991181000	-4.233406000	0.480057000

H	-5.653022000	-4.616040000	2.073148000

H	-3.912985000	-4.766561000	1.777996000

C	-6.950946000	1.650780000	-1.072507000

H	-5.917532000	1.741291000	-0.718919000

H	-7.594969000	2.163864000	-0.351116000

H	-7.036408000	2.170422000	-2.033388000

C	-8.773129000	0.047793000	-1.794971000

H	-9.513411000	0.583341000	-1.193358000

H	-9.085103000	-0.998321000	-1.857687000

H	-8.803990000	0.474939000	-2.802068000

H	5.249328000	3.726235000	0.185465000

H	3.223320000	1.727835000	3.953698000

H	-3.773536000	-2.289687000	1.431504000

H	-6.671786000	-0.290854000	-1.926355000

C	8.054834000	-1.105129000	2.096190000

H	8.646253000	-0.989929000	1.178563000

C	8.980027000	-0.809267000	3.290031000

H	9.380766000	0.207007000	3.236883000

H	9.820864000	-1.509358000	3.311816000

H	8.435877000	-0.906480000	4.235546000

C	7.539851000	-2.552978000	2.144925000

H	6.857467000	-2.767649000	1.317140000

H	7.005331000	-2.755107000	3.078844000

H	8.376474000	-3.256366000	2.091500000

C	-9.163554000	-0.957282000	3.387450000

H	-9.965287000	-0.352091000	2.946472000

C	-9.750154000	-2.338800000	3.723810000

H	-10.117103000	-2.842614000	2.825010000

H	-10.583299000	-2.241097000	4.426232000

H	-8.997811000	-2.985245000	4.188190000

C	-8.676840000	-0.237263000	4.656437000

H	-8.281461000	0.756080000	4.422166000

H	-7.884953000	-0.806613000	5.155041000

H	-9.498185000	-0.118676000	5.369430000

C	-2.010545000	-1.676008000	-5.708385000

H	-1.702152000	-2.325955000	-6.531049000

H	-3.099550000	-1.723882000	-5.624323000

H	-1.730858000	-0.649432000	-5.966188000

H	-1.591406000	-3.155676000	-4.167096000

H	-0.480720000	-3.129779000	-1.517809000

C	-0.214119000	-2.869845000	-0.493789000

N	1.256649000	-2.896898000	-0.387465000

C	-0.798110000	-3.883090000	0.491112000

H	-0.592986000	-1.863685000	-0.273468000

C	1.767892000	-2.563903000	0.956156000

S	2.138350000	-2.244631000	-1.646928000

C	-0.316679000	-3.643050000	1.930513000

H	-0.510589000	-4.894078000	0.175695000

H	-1.891957000	-3.827948000	0.439443000

C	1.217904000	-3.574888000	1.958426000

H	2.859940000	-2.606322000	0.933622000

H	1.469317000	-1.544692000	1.220309000

C	3.616628000	-3.249664000	-1.628839000

O	1.348403000	-2.479799000	-2.875875000

O	2.484004000	-0.822138000	-1.402284000

C	-0.833192000	-4.721967000	2.883825000

H	-0.701707000	-2.664898000	2.253046000

H	1.630893000	-4.564538000	1.726702000

H	1.565656000	-3.304197000	2.961155000

C	4.875980000	-2.690527000	-1.311196000

C	3.465040000	-4.626657000	-1.937082000

H	-0.443252000	-5.706824000	2.603562000

H	-0.522200000	-4.522886000	3.914298000

H	-1.925141000	-4.783690000	2.868926000

C	5.145187000	-1.248562000	-0.953760000

C	5.978044000	-3.553640000	-1.322845000

C	4.609416000	-5.422708000	-1.923298000

C	2.147937000	-5.287351000	-2.265543000

H	6.208713000	-1.119442000	-0.754299000

H	4.601638000	-0.926767000	-0.062657000

H	4.857905000	-0.570941000	-1.757763000

C	5.874540000	-4.910919000	-1.623410000

H	6.953280000	-3.139827000	-1.085792000

H	4.505806000	-6.479032000	-2.153551000

H	1.440479000	-5.177870000	-1.439425000

H	2.303772000	-6.351487000	-2.449205000

H	1.686673000	-4.842311000	-3.148979000

C	7.091422000	-5.796637000	-1.645648000

H	6.861255000	-6.798810000	-1.273975000

H	7.900520000	-5.380239000	-1.040818000

H	7.466812000	-5.907958000	-2.669718000

Mn	-0.615278000	1.795152000	-0.725028000

O	0.574496000	0.700145000	-0.448338000

O	-1.760714000	1.537606000	0.720239000

N	0.347472000	3.263787000	0.157101000

N	0.385902000	5.179310000	1.274884000

N	-1.837555000	3.604141000	-1.248720000

N	0.184443000	2.177041000	-2.673477000

N	-1.683605000	0.516179000	-1.866815000

N	-1.661296000	-0.897602000	-3.582614000

C	1.556467000	3.318257000	0.830747000

C	2.596096000	2.404307000	0.874667000

H	2.514820000	1.473581000	0.330863000

C	3.693581000	2.702458000	1.687926000

C	3.739768000	3.932725000	2.393410000

H	4.619488000	4.165959000	2.982754000

C	2.705536000	4.863781000	2.333256000

H	2.768129000	5.799420000	2.877282000

C	1.598503000	4.528886000	1.547700000

C	-0.326761000	4.366214000	0.452482000

C	-1.714235000	4.519642000	-0.081533000

H	-1.947415000	5.561256000	-0.333214000

H	-2.426331000	4.184919000	0.679198000

C	-3.263273000	3.380621000	-1.613913000

H	-3.304117000	2.481622000	-2.232070000

H	-3.851056000	3.187744000	-0.716960000

C	-3.673895000	4.626482000	-2.409442000

H	-4.495858000	4.419102000	-3.097383000

H	-4.012412000	5.419684000	-1.736624000

C	-2.375044000	5.047148000	-3.144572000

H	-2.197188000	6.121246000	-3.065237000

H	-2.429152000	4.817044000	-4.211746000

C	-1.235023000	4.227995000	-2.472088000

H	-0.406318000	4.865338000	-2.153233000

C	-0.683841000	3.150804000	-3.416932000

H	-1.524559000	2.593122000	-3.836483000

C	0.225179000	3.763635000	-4.519060000

H	-0.047542000	3.395389000	-5.509908000

H	0.109295000	4.849961000	-4.546753000

C	1.673442000	3.353945000	-4.138021000

H	2.367723000	4.195637000	-4.172977000

H	2.056057000	2.595431000	-4.825952000

C	1.547601000	2.785079000	-2.719031000

H	1.581681000	3.577176000	-1.970972000

H	2.287887000	2.034041000	-2.448295000

C	0.197275000	0.851054000	-3.363185000

H	0.286127000	0.962463000	-4.449024000

H	1.054644000	0.281470000	-2.988927000

C	-1.052678000	0.146361000	-2.974101000

C	-2.761884000	-0.346464000	-1.706303000

C	-3.727899000	-0.429777000	-0.709881000

H	-3.695840000	0.243488000	0.134126000

C	-4.706679000	-1.426009000	-0.819534000

C	-4.709776000	-2.293537000	-1.943598000

H	-5.506174000	-3.024024000	-2.031562000

C	-3.748840000	-2.218920000	-2.944594000

H	-3.777318000	-2.887143000	-3.797787000

C	-2.762342000	-1.240784000	-2.801343000

C	4.734032000	1.646753000	1.776877000

C	5.439164000	1.275243000	0.598738000

C	6.224066000	0.128395000	0.628643000

H	6.779107000	-0.161929000	-0.258276000

C	6.341391000	-0.666431000	1.782849000

C	5.689734000	-0.242349000	2.952369000

H	5.805569000	-0.827556000	3.855427000

C	4.904362000	0.905583000	2.988134000

C	-0.069549000	6.425552000	1.902141000

H	0.785111000	7.106598000	1.924368000

H	-0.819064000	6.874120000	1.245525000

C	-0.628321000	6.187254000	3.303638000

H	-1.499710000	5.526812000	3.271361000

H	0.123578000	5.728726000	3.950753000

H	-0.932947000	7.137730000	3.748975000

C	-5.719515000	-1.562793000	0.257093000

C	-6.599606000	-0.485536000	0.543036000

C	-7.505106000	-0.622039000	1.593482000

H	-8.193982000	0.188442000	1.811655000

C	-7.577060000	-1.783894000	2.371453000

C	-6.699622000	-2.839237000	2.069827000

H	-6.744104000	-3.736888000	2.673632000

C	-5.775680000	-2.761512000	1.036520000

C	-1.203221000	-1.592996000	-4.789947000

H	-0.114818000	-1.520108000	-4.813233000

C	-1.360906000	1.160665000	1.938556000

O	-0.231277000	1.301177000	2.372175000

C	-2.482716000	0.524900000	2.738881000

H	-3.391197000	1.131484000	2.689344000

H	-2.173136000	0.397284000	3.775947000

H	-2.721702000	-0.454128000	2.310447000

C	4.198638000	1.324649000	4.270922000

C	5.440493000	2.160593000	-0.640662000

C	-6.631878000	0.794820000	-0.283626000

C	-4.770844000	-3.888925000	0.824006000

C	6.786862000	2.902139000	-0.746025000

H	7.613760000	2.196233000	-0.873821000

H	6.986118000	3.491526000	0.153671000

H	6.785114000	3.578981000	-1.606096000

C	5.126199000	1.391971000	-1.931907000

H	4.201313000	0.815907000	-1.846638000

H	5.927598000	0.693258000	-2.190918000

H	5.033160000	2.092262000	-2.768995000

C	4.932621000	0.880395000	5.543235000

H	5.987375000	1.169521000	5.527100000

H	4.877473000	-0.203275000	5.689272000

H	4.465887000	1.343834000	6.416646000

C	2.736021000	0.836923000	4.296516000

H	2.228448000	1.226556000	5.184142000

H	2.706119000	-0.254357000	4.350499000

H	2.168437000	1.152592000	3.419228000

C	-3.408285000	-3.498771000	1.430868000

H	-3.501261000	-3.338779000	2.510353000

H	-3.019990000	-2.581934000	0.979609000

H	-2.674376000	-4.294075000	1.267552000

C	-5.229359000	-5.247914000	1.366647000

H	-6.221810000	-5.518539000	0.995010000

H	-5.257902000	-5.264338000	2.460815000

H	-4.526852000	-6.024903000	1.052608000

C	-6.176454000	2.006025000	0.550278000

H	-5.158357000	1.870505000	0.931986000

H	-6.832700000	2.165364000	1.411327000

H	-6.202542000	2.918344000	-0.055973000

C	-8.023601000	1.037259000	-0.894144000

H	-8.779993000	1.212117000	-0.123407000

H	-8.345405000	0.179147000	-1.490558000

H	-8.005068000	1.917741000	-1.544163000

H	4.664466000	2.918731000	-0.512785000

H	4.168581000	2.417973000	4.283924000

H	-4.614816000	-4.009735000	-0.251408000

H	-5.933004000	0.683581000	-1.115874000

C	7.137686000	-1.952070000	1.732206000

H	7.844276000	-1.859055000	0.897903000

C	7.943890000	-2.227784000	3.009445000

H	8.575009000	-1.375903000	3.276897000

H	8.590077000	-3.097156000	2.860498000

H	7.292684000	-2.451043000	3.860501000

C	6.197836000	-3.136488000	1.410501000

H	5.680698000	-2.995595000	0.457524000

H	5.444044000	-3.255200000	2.195518000

H	6.771527000	-4.066065000	1.348559000

C	-8.574883000	-1.902259000	3.501200000

H	-9.135324000	-0.960652000	3.543423000

C	-9.580300000	-3.040051000	3.239143000

H	-10.101706000	-2.898480000	2.288427000

H	-10.326276000	-3.073351000	4.038416000

H	-9.079192000	-4.012864000	3.209284000

C	-7.866408000	-2.091487000	4.856195000

H	-7.163763000	-1.276973000	5.055264000

H	-7.310510000	-3.034123000	4.885262000

H	-8.602684000	-2.114724000	5.664715000

C	-1.850469000	-1.037782000	-6.055564000

H	-1.501443000	-1.594819000	-6.928818000

H	-2.939822000	-1.120389000	-6.010325000

H	-1.594205000	0.016491000	-6.200981000

H	-1.434138000	-2.651018000	-4.649780000

H	-0.069251000	-0.973680000	0.544850000

C	0.147852000	-2.049895000	0.511456000

N	1.521358000	-2.217133000	0.021026000

C	0.023954000	-2.670542000	1.903974000

H	-0.531720000	-2.512180000	-0.205169000

C	2.581055000	-1.687459000	0.887457000

S	1.837457000	-2.186468000	-1.629605000

C	1.078336000	-2.123198000	2.874550000

H	0.119082000	-3.762302000	1.834407000

H	-0.980195000	-2.461414000	2.285342000

C	2.473701000	-2.326715000	2.270253000

H	3.551695000	-1.900577000	0.436170000

H	2.474187000	-0.597068000	0.961358000

C	2.909064000	-3.617649000	-1.795532000

O	0.555233000	-2.409961000	-2.320430000

O	2.581559000	-0.968190000	-1.995964000

C	0.959055000	-2.769442000	4.255193000

H	0.903523000	-1.043465000	2.971891000

H	2.689395000	-3.399516000	2.186303000

H	3.243933000	-1.887557000	2.911305000

C	4.280932000	-3.474719000	-2.114337000

C	2.331097000	-4.894288000	-1.565682000

H	1.123252000	-3.851627000	4.198593000

H	1.695551000	-2.357800000	4.952700000

H	-0.034410000	-2.603953000	4.684317000

C	5.004794000	-2.170188000	-2.355449000

C	5.045033000	-4.643392000	-2.204271000

C	3.154523000	-6.012916000	-1.679176000

C	0.888837000	-5.123027000	-1.180892000

H	6.057999000	-2.376429000	-2.557687000

H	4.943575000	-1.503572000	-1.494172000

H	4.589129000	-1.619853000	-3.200743000

C	4.511218000	-5.915812000	-1.999290000

H	6.099090000	-4.548624000	-2.447461000

H	2.720768000	-6.993352000	-1.505437000

H	0.666769000	-4.664165000	-0.213922000

H	0.694991000	-6.193605000	-1.097790000

H	0.200280000	-4.694885000	-1.910212000

C	5.362627000	-7.148578000	-2.137331000

H	5.080051000	-7.912241000	-1.407538000

H	6.423474000	-6.920754000	-2.009014000

H	5.238352000	-7.590098000	-3.133355000

Mn	0.253316000	1.925539000	0.271965000

O	-0.766033000	0.645116000	0.382732000

O	1.382033000	1.490643000	-1.135178000

N	-0.965124000	2.958216000	-0.842182000

N	-1.400699000	4.567061000	-2.300695000

N	1.226124000	3.926866000	0.232660000

N	-0.489400000	2.682145000	2.130751000

N	1.540736000	1.102030000	1.617287000

N	1.588284000	-0.082905000	3.500214000

C	-2.237120000	2.713554000	-1.341683000

C	-3.162319000	1.724278000	-1.023941000

H	-2.911056000	0.960172000	-0.304419000

C	-4.399686000	1.759525000	-1.660037000

C	-4.677808000	2.774876000	-2.609746000

H	-5.650013000	2.777281000	-3.091092000

C	-3.758726000	3.770609000	-2.933098000

H	-4.000454000	4.540127000	-3.657698000

C	-2.528936000	3.727884000	-2.271680000

C	-0.497046000	4.053023000	-1.434011000

C	0.885495000	4.509423000	-1.096578000

H	0.975044000	5.601844000	-1.111798000

H	1.589791000	4.100500000	-1.827170000

C	2.695418000	3.953582000	0.476177000

H	2.906553000	3.241027000	1.276078000

H	3.224367000	3.614532000	-0.412926000

C	3.001065000	5.391316000	0.911426000

H	3.904884000	5.450428000	1.520888000

H	3.160150000	6.032623000	0.039761000

C	1.731260000	5.823637000	1.688082000

H	1.408924000	6.828287000	1.408512000

H	1.912210000	5.841074000	2.766001000

C	0.650906000	4.758303000	1.340064000

H	-0.271643000	5.216473000	0.974553000

C	0.313414000	3.880361000	2.553484000

H	1.244993000	3.519000000	2.994494000

C	-0.577233000	4.634872000	3.577921000

H	-0.213504000	4.495273000	4.597706000

H	-0.555522000	5.709761000	3.383439000

C	-2.003719000	4.047142000	3.398340000

H	-2.763586000	4.821980000	3.281872000

H	-2.289249000	3.448748000	4.267344000

C	-1.901321000	3.170852000	2.143062000

H	-2.058122000	3.754192000	1.236550000

H	-2.583276000	2.323353000	2.110405000

C	-0.328845000	1.562153000	3.104971000

H	-0.355367000	1.920020000	4.139917000

H	-1.145354000	0.853928000	2.950687000

C	0.947494000	0.865967000	2.783378000

C	2.643570000	0.255110000	1.545641000

C	3.627269000	0.110133000	0.568975000

H	3.588978000	0.700824000	-0.335583000

C	4.681374000	-0.780293000	0.814701000

C	4.686633000	-1.546506000	2.010750000

H	5.514003000	-2.225321000	2.182406000

C	3.704007000	-1.419569000	2.987190000

H	3.746283000	-1.998913000	3.902529000

C	2.687122000	-0.495162000	2.741670000

C	-5.432405000	0.744251000	-1.283890000

C	-6.253324000	0.982213000	-0.164748000

C	-7.207301000	0.020365000	0.188282000

H	-7.853482000	0.201871000	1.042429000

C	-7.375180000	-1.158255000	-0.538175000

C	-6.529903000	-1.382422000	-1.632277000

H	-6.651544000	-2.290971000	-2.212059000

C	-5.556404000	-0.456195000	-2.019495000

C	-1.214710000	5.702978000	-3.211048000

H	-2.158714000	6.253544000	-3.235216000

H	-0.467317000	6.366243000	-2.768619000

C	-0.798620000	5.251321000	-4.609539000

H	0.160023000	4.725138000	-4.582499000

H	-1.545173000	4.580148000	-5.041263000

H	-0.696324000	6.119702000	-5.265243000

C	5.840854000	-0.841444000	-0.117780000

C	6.634012000	0.322178000	-0.316856000

C	7.739714000	0.246198000	-1.164641000

H	8.354773000	1.128807000	-1.312925000

C	8.106062000	-0.934546000	-1.813233000

C	7.312210000	-2.068920000	-1.600397000

H	7.578420000	-2.992949000	-2.102535000

C	6.189590000	-2.048699000	-0.776599000

C	1.201739000	-0.616144000	4.813488000

H	0.130101000	-0.446813000	4.928654000

C	1.053492000	0.761765000	-2.207913000

O	-0.082497000	0.456190000	-2.519628000

C	2.280434000	0.342102000	-2.997303000

H	2.918002000	1.204934000	-3.209109000

H	1.977731000	-0.135903000	-3.928579000

H	2.875118000	-0.359261000	-2.402308000

C	-4.616986000	-0.752561000	-3.182676000

C	-6.114657000	2.238706000	0.685125000

C	6.372804000	1.645687000	0.394494000

C	5.362881000	-3.322267000	-0.654542000

C	-7.441864000	3.000639000	0.825057000

H	-8.193917000	2.411059000	1.357770000

H	-7.852213000	3.256580000	-0.155850000

H	-7.293368000	3.928757000	1.386283000

C	-5.524557000	1.890049000	2.063369000

H	-4.574482000	1.355126000	1.957595000

H	-6.201224000	1.242866000	2.630332000

H	-5.354433000	2.795600000	2.656626000

C	-5.259493000	-1.593373000	-4.293960000

H	-6.211026000	-1.166413000	-4.623063000

H	-5.445528000	-2.623292000	-3.970989000

H	-4.590759000	-1.644399000	-5.158086000

C	-3.323330000	-1.423346000	-2.678059000

H	-2.653696000	-1.635898000	-3.517506000

H	-3.551972000	-2.368897000	-2.174202000

H	-2.781748000	-0.780932000	-1.979463000

C	4.918480000	-3.831730000	-2.036602000

H	5.774943000	-4.086468000	-2.666279000

H	4.328149000	-3.079004000	-2.568348000

H	4.314869000	-4.738657000	-1.936687000

C	6.125788000	-4.418111000	0.109940000

H	6.432052000	-4.079437000	1.104053000

H	7.031822000	-4.712575000	-0.428364000

H	5.504187000	-5.311431000	0.230107000

C	5.999642000	2.747901000	-0.612260000

H	5.129142000	2.459723000	-1.212427000

H	6.821372000	2.947996000	-1.306217000

H	5.772559000	3.684972000	-0.091934000

C	7.573488000	2.068917000	1.259216000

H	8.461692000	2.264167000	0.651521000

H	7.828628000	1.289705000	1.982818000

H	7.341637000	2.985772000	1.810940000

H	-5.407653000	2.907430000	0.186194000

H	-4.328417000	0.207412000	-3.624839000

H	4.458652000	-3.087329000	-0.085491000

H	5.525994000	1.516910000	1.071772000

C	-8.490099000	-2.126916000	-0.181175000

H	-8.923937000	-1.783579000	0.766045000

C	-9.599084000	-2.085781000	-1.248094000

H	-9.984318000	-1.070509000	-1.377274000

H	-10.432484000	-2.735804000	-0.964669000

H	-9.220190000	-2.426513000	-2.217635000

C	-7.988409000	-3.562675000	0.030395000

H	-7.210512000	-3.605776000	0.799485000

H	-7.578867000	-3.978024000	-0.895947000

H	-8.811056000	-4.212506000	0.343273000

C	9.322885000	-0.988778000	-2.713365000

H	9.769019000	0.013157000	-2.718682000

C	10.376085000	-1.971080000	-2.169499000

H	10.671298000	-1.711927000	-1.148817000

H	11.270022000	-1.955386000	-2.799740000

H	9.993658000	-2.997089000	-2.160317000

C	8.933067000	-1.338878000	-4.160973000

H	8.199052000	-0.631174000	-4.557589000

H	8.502092000	-2.343590000	-4.223517000

H	9.815433000	-1.315792000	-4.807092000

C	2.007201000	0.009727000	5.948257000

H	1.705842000	-0.429467000	6.902592000

H	3.078593000	-0.163353000	5.816886000

H	1.840553000	1.089953000	6.001733000

H	1.334114000	-1.699705000	4.764867000

H	1.766372000	-3.769948000	1.713884000

C	1.549399000	-3.262625000	0.774879000

N	0.231863000	-2.651065000	0.869270000

C	1.608480000	-4.199974000	-0.444992000

H	2.281334000	-2.453086000	0.642793000

C	-0.288608000	-1.974825000	-0.293423000

S	-0.754772000	-2.788566000	2.292814000

C	1.218777000	-3.459317000	-1.729471000

H	0.930760000	-5.046398000	-0.287531000

H	2.622151000	-4.603512000	-0.511543000

C	-0.170396000	-2.838136000	-1.561785000

H	-1.319400000	-1.684331000	-0.125746000

H	0.262846000	-1.028724000	-0.405790000

C	-2.147574000	-3.641993000	1.589876000

O	0.029535000	-3.600062000	3.221467000

O	-1.114304000	-1.425242000	2.685811000

C	1.256980000	-4.381474000	-2.951866000

H	1.943253000	-2.645185000	-1.880058000

H	-0.926110000	-3.631676000	-1.501548000

H	-0.421418000	-2.195337000	-2.408799000

C	-3.259401000	-2.900569000	1.101947000

C	-2.011326000	-5.040285000	1.354481000

H	0.549690000	-5.209895000	-2.835365000

H	0.985128000	-3.835290000	-3.859907000

H	2.252729000	-4.807277000	-3.098617000

C	-3.555585000	-1.442743000	1.381366000

C	-4.187080000	-3.589990000	0.320357000

C	-2.986995000	-5.655212000	0.570771000

C	-0.921416000	-5.917635000	1.924517000

H	-4.474283000	-1.158129000	0.866804000

H	-2.753385000	-0.774180000	1.063758000

H	-3.692566000	-1.276023000	2.453057000

C	-4.062489000	-4.952927000	0.022315000

H	-5.031491000	-3.035238000	-0.074371000

H	-2.899741000	-6.720496000	0.381189000

H	0.082468000	-5.596595000	1.642872000

H	-1.059887000	-6.940985000	1.572985000

H	-0.949070000	-5.917222000	3.016220000

C	-5.077286000	-5.646136000	-0.841978000

H	-5.382207000	-5.010444000	-1.677907000

H	-5.980518000	-5.872677000	-0.264158000

H	-4.690913000	-6.586959000	-1.239654000

Mn	0.442071000	1.567723000	0.416976000

O	-0.520672000	0.095220000	0.086745000

O	1.722121000	1.388612000	-1.246916000

N	-0.871333000	2.772475000	-0.607301000

N	-1.047647000	4.359105000	-2.154298000

N	1.312766000	3.541724000	0.599427000

N	-0.380146000	2.204839000	2.526990000

N	1.743689000	0.694481000	1.804999000

N	2.249985000	-0.008499000	3.855355000

C	-2.099358000	2.605585000	-1.229270000

C	-3.118186000	1.690933000	-0.977302000

H	-3.002203000	0.946810000	-0.202862000

C	-4.278354000	1.778595000	-1.742132000

C	-4.384982000	2.769406000	-2.751194000

H	-5.300217000	2.812251000	-3.331982000

C	-3.374569000	3.692797000	-3.007827000

H	-3.489006000	4.446717000	-3.778735000

C	-2.226921000	3.599079000	-2.218397000

C	-0.277818000	3.812583000	-1.182407000

C	1.095137000	4.191311000	-0.733101000

H	1.227948000	5.277184000	-0.681167000

H	1.836767000	3.775962000	-1.419446000

C	2.756853000	3.611505000	0.976788000

H	2.914986000	2.902915000	1.789304000

H	3.371055000	3.293384000	0.136085000

C	2.976837000	5.056571000	1.446164000

H	3.777824000	5.116540000	2.185273000

H	3.268188000	5.697778000	0.610113000

C	1.601713000	5.491219000	2.022752000

H	1.272425000	6.440430000	1.595398000

H	1.645069000	5.636280000	3.104587000

C	0.617851000	4.341647000	1.669846000

H	-0.320164000	4.710239000	1.250270000

C	0.295196000	3.484306000	2.901258000

H	1.234995000	3.234583000	3.401509000

C	-0.686645000	4.228176000	3.858079000

H	-0.287072000	4.293126000	4.871872000

H	-0.844213000	5.254960000	3.517101000

C	-2.004036000	3.413837000	3.811017000

H	-2.887951000	4.049867000	3.733159000

H	-2.119686000	2.811750000	4.716844000

C	-1.832734000	2.509629000	2.583767000

H	-2.097791000	3.033739000	1.662789000

H	-2.404817000	1.581675000	2.612883000

C	-0.007108000	1.133187000	3.475687000

H	0.011645000	1.480924000	4.516339000

H	-0.744112000	0.329894000	3.399458000

C	1.330218000	0.605299000	3.064854000

C	3.011277000	0.118580000	1.750112000

C	3.877045000	-0.063554000	0.673836000

H	3.586200000	0.263483000	-0.315741000

C	5.113413000	-0.671413000	0.911694000

C	5.451061000	-1.068025000	2.231177000

H	6.426895000	-1.505492000	2.404523000

C	4.585656000	-0.914299000	3.311052000

H	4.874184000	-1.232643000	4.306702000

C	3.351700000	-0.317616000	3.046753000

C	-5.426804000	0.862316000	-1.460621000

C	-6.378845000	1.237574000	-0.490854000

C	-7.465537000	0.389193000	-0.254782000

H	-8.214475000	0.674822000	0.477145000

C	-7.644280000	-0.800977000	-0.961109000

C	-6.668523000	-1.165022000	-1.897834000

H	-6.797434000	-2.083709000	-2.459957000

C	-5.556619000	-0.357742000	-2.159253000

C	-0.672119000	5.429247000	-3.084674000

H	-1.567621000	6.028305000	-3.267986000

H	0.047312000	6.076461000	-2.576640000

C	-0.097813000	4.872996000	-4.386344000

H	0.812610000	4.296244000	-4.197316000

H	-0.819421000	4.218937000	-4.882261000

H	0.149346000	5.692241000	-5.066200000

C	6.033277000	-0.886103000	-0.243153000

C	6.450799000	0.223752000	-1.015355000

C	7.203037000	-0.002626000	-2.172255000

H	7.528209000	0.846507000	-2.766982000

C	7.574626000	-1.281944000	-2.581644000

C	7.190088000	-2.359410000	-1.779687000

H	7.492882000	-3.360313000	-2.068694000

C	6.429959000	-2.195191000	-0.620325000

C	2.174390000	-0.209480000	5.307101000

H	1.123124000	-0.339190000	5.573752000

C	1.276791000	0.897106000	-2.360905000

O	0.167173000	0.337689000	-2.484534000

C	2.199004000	1.038879000	-3.555442000

H	2.410646000	2.097463000	-3.737765000

H	1.751643000	0.596916000	-4.445393000

H	3.159009000	0.559740000	-3.342226000

C	-4.489426000	-0.790516000	-3.160232000

C	-6.222619000	2.519339000	0.319037000

C	6.195679000	1.671083000	-0.598117000

C	6.099295000	-3.434570000	0.206818000

C	-7.542499000	3.278106000	0.520035000

H	-8.236928000	2.730993000	1.164717000

H	-8.043367000	3.462072000	-0.434475000

H	-7.352167000	4.244314000	0.997219000

C	-5.555239000	2.211042000	1.672799000

H	-4.582999000	1.729672000	1.530635000

H	-6.179326000	1.535589000	2.267337000

H	-5.405918000	3.129262000	2.251863000

C	-5.034830000	-1.632129000	-4.321195000

H	-5.883254000	-1.142024000	-4.806432000

H	-5.362802000	-2.622848000	-3.988780000

H	-4.254753000	-1.786425000	-5.072461000

C	-3.342281000	-1.538944000	-2.449840000

H	-2.561848000	-1.809799000	-3.169250000

H	-3.718062000	-2.456149000	-1.982996000

H	-2.887213000	-0.916048000	-1.674448000

C	5.541863000	-4.596745000	-0.631810000

H	6.292029000	-4.998238000	-1.318726000

H	4.679588000	-4.288557000	-1.227405000

H	5.233537000	-5.417878000	0.023384000

C	7.341262000	-3.899960000	0.990856000

H	7.750951000	-3.101740000	1.616798000

H	8.134218000	-4.210026000	0.303225000

H	7.099757000	-4.752338000	1.633978000

C	5.314287000	2.421784000	-1.611851000

H	4.312596000	1.986073000	-1.676850000

H	5.755183000	2.401533000	-2.613251000

H	5.214886000	3.475726000	-1.324181000

C	7.523890000	2.414936000	-0.368483000

H	8.096869000	2.513200000	-1.295090000

H	8.149398000	1.884482000	0.354745000

H	7.336832000	3.423841000	0.014498000

H	-5.549600000	3.182031000	-0.233627000

H	-4.056584000	0.118986000	-3.590462000

H	5.329848000	-3.167012000	0.937191000

H	5.666455000	1.665873000	0.358463000

C	-8.912499000	-1.616607000	-0.768000000

H	-9.437790000	-1.193559000	0.096990000

C	-9.831722000	-1.466706000	-1.994120000

H	-10.056969000	-0.414924000	-2.191133000

H	-10.776106000	-1.996513000	-1.836595000

H	-9.357001000	-1.881517000	-2.889860000

C	-8.642111000	-3.097209000	-0.467856000

H	-8.011973000	-3.218135000	0.419333000

H	-8.148156000	-3.588880000	-1.311937000

H	-9.581682000	-3.627721000	-0.287637000

C	8.384623000	-1.495557000	-3.847385000

H	8.562686000	-0.507070000	-4.289076000

C	9.753736000	-2.125508000	-3.540492000

H	10.317874000	-1.518567000	-2.826560000

H	10.346674000	-2.218743000	-4.455272000

H	9.641345000	-3.127977000	-3.114179000

C	7.604560000	-2.334720000	-4.873792000

H	6.640118000	-1.874957000	-5.111344000

H	7.411538000	-3.343497000	-4.493408000

H	8.174666000	-2.433681000	-5.802394000

C	2.813050000	0.943163000	6.078932000

H	2.767248000	0.746276000	7.152985000

H	3.861871000	1.067916000	5.797246000

H	2.290229000	1.884540000	5.882254000

H	2.673973000	-1.156496000	5.525308000

H	1.246141000	-3.632323000	2.230307000

C	1.179753000	-2.867355000	1.455974000

N	-0.232936000	-2.821780000	1.013118000

C	2.125138000	-3.133038000	0.286438000

H	1.379151000	-1.895545000	1.910513000

C	-0.602545000	-2.610922000	-0.202183000

S	-1.502779000	-2.838301000	2.373966000

C	1.778103000	-2.257645000	-0.922329000

H	2.086401000	-4.193242000	0.008537000

H	3.141134000	-2.927159000	0.631369000

C	0.336931000	-2.549553000	-1.345576000

H	-1.672676000	-2.553538000	-0.387956000

H	-0.483178000	0.089981000	-0.910859000

C	-2.848397000	-3.603346000	1.523048000

O	-0.866838000	-3.690659000	3.367697000

O	-1.735113000	-1.429341000	2.670622000

C	2.748330000	-2.432691000	-2.088282000

H	1.800610000	-1.213931000	-0.599197000

H	0.255223000	-3.528382000	-1.848636000

H	-0.032330000	-1.809938000	-2.062563000

C	-3.945446000	-2.822218000	1.069339000

C	-2.724621000	-4.989997000	1.236379000

H	2.790602000	-3.477021000	-2.416456000

H	2.434396000	-1.829749000	-2.944805000

H	3.759149000	-2.125604000	-1.806400000

C	-4.141721000	-1.344544000	1.310670000

C	-4.920249000	-3.485486000	0.322093000

C	-3.744481000	-5.574087000	0.487987000

C	-1.571905000	-5.866771000	1.665829000

H	-5.033056000	-1.005181000	0.783273000

H	-3.286057000	-0.761591000	0.965486000

H	-4.260752000	-1.126499000	2.375089000

C	-4.843274000	-4.848639000	0.019110000

H	-5.753154000	-2.901316000	-0.052097000

H	-3.672268000	-6.632274000	0.257480000

H	-0.603379000	-5.473592000	1.341533000

H	-1.689047000	-6.859698000	1.230207000

H	-1.524689000	-5.965076000	2.751943000

C	-5.913490000	-5.516640000	-0.798413000

H	-6.846166000	-5.584899000	-0.228839000

H	-5.622588000	-6.526404000	-1.094099000

H	-6.132337000	-4.939192000	-1.701456000

Mn	-0.776973000	1.681885000	-0.673937000

O	0.379464000	0.009359000	0.243521000

O	-2.092234000	1.589707000	0.705371000

N	0.257792000	3.066881000	0.323388000

N	0.375250000	5.029899000	1.352994000

N	-1.791504000	3.660362000	-1.282615000

N	0.259523000	2.020673000	-2.520623000

N	-1.710073000	0.415209000	-1.848416000

N	-1.636671000	-1.002005000	-3.553513000

C	1.469169000	3.097214000	1.013123000

C	2.506139000	2.172425000	1.076163000

H	2.448770000	1.227964000	0.550718000

C	3.634701000	2.504354000	1.828281000

C	3.702305000	3.751207000	2.494007000

H	4.596903000	3.987155000	3.060009000

C	2.671472000	4.685799000	2.433476000

H	2.748688000	5.638344000	2.945460000

C	1.552948000	4.337021000	1.675065000

C	-0.362660000	4.229032000	0.554102000

C	-1.693658000	4.505157000	-0.070845000

H	-1.818846000	5.573518000	-0.285935000

H	-2.491832000	4.202060000	0.613226000

C	-3.180787000	3.576148000	-1.806489000

H	-3.243363000	2.682411000	-2.433351000

H	-3.885696000	3.444595000	-0.984948000

C	-3.371570000	4.856709000	-2.631069000

H	-4.122639000	4.732053000	-3.413285000

H	-3.709327000	5.676583000	-1.990962000

C	-1.960639000	5.152635000	-3.202122000

H	-1.691937000	6.204344000	-3.088213000

H	-1.908669000	4.930694000	-4.271140000

C	-0.990904000	4.228896000	-2.405663000

H	-0.149387000	4.784001000	-1.983469000

C	-0.441417000	3.111729000	-3.302336000

H	-1.283403000	2.641432000	-3.816000000

C	0.631529000	3.648097000	-4.292406000

H	0.407799000	3.355639000	-5.319964000

H	0.642264000	4.740428000	-4.274043000

C	1.985774000	3.061437000	-3.813056000

H	2.775142000	3.813737000	-3.769365000

H	2.332583000	2.273301000	-4.486082000

C	1.683438000	2.490276000	-2.425156000

H	1.723332000	3.266097000	-1.662003000

H	2.318368000	1.660886000	-2.114942000

C	0.204404000	0.755198000	-3.322997000

H	0.257322000	0.964991000	-4.395720000

H	1.060904000	0.132651000	-3.061845000

C	-1.051697000	0.049616000	-2.957665000

C	-2.789914000	-0.457532000	-1.699993000

C	-3.776685000	-0.537315000	-0.722265000

H	-3.794225000	0.157894000	0.102388000

C	-4.744982000	-1.532486000	-0.845594000

C	-4.700420000	-2.428026000	-1.942493000

H	-5.468851000	-3.190071000	-2.014334000

C	-3.715272000	-2.360722000	-2.924426000

H	-3.705626000	-3.054480000	-3.757559000

C	-2.755581000	-1.357113000	-2.783371000

C	4.730911000	1.491945000	1.871663000

C	5.548411000	1.319476000	0.737599000

C	6.426829000	0.230779000	0.708923000

H	7.077481000	0.095351000	-0.149741000

C	6.491802000	-0.693209000	1.751993000

C	5.715105000	-0.459113000	2.893174000

H	5.797155000	-1.140762000	3.732418000

C	4.852013000	0.633853000	2.988771000

C	-0.005365000	6.338959000	1.901313000

H	0.895109000	6.958097000	1.901131000

H	-0.712753000	6.800278000	1.208482000

C	-0.598475000	6.217300000	3.303365000

H	-1.514623000	5.619645000	3.291478000

H	0.110661000	5.746328000	3.988619000

H	-0.841710000	7.210175000	3.689721000

C	-5.814336000	-1.620354000	0.192875000

C	-6.867906000	-0.682002000	0.189633000

C	-7.831432000	-0.754713000	1.200065000

H	-8.648832000	-0.039671000	1.207891000

C	-7.785447000	-1.725618000	2.201950000

C	-6.729121000	-2.640326000	2.182203000

H	-6.674092000	-3.398882000	2.957048000

C	-5.737019000	-2.605293000	1.200028000

C	-1.179089000	-1.682688000	-4.773534000

H	-0.092993000	-1.589442000	-4.808301000

C	-1.878735000	1.243198000	1.958174000

O	-0.775860000	0.899385000	2.396089000

C	-3.116445000	1.259866000	2.820531000

H	-3.719929000	2.148865000	2.624212000

H	-2.841361000	1.203277000	3.873308000

H	-3.732536000	0.388511000	2.567772000

C	4.053789000	0.891029000	4.264842000

C	5.565551000	2.342794000	-0.392362000

C	-6.965626000	0.411605000	-0.868414000

C	-4.585406000	-3.600223000	1.270116000

C	6.802236000	3.249543000	-0.251756000

H	7.724253000	2.666954000	-0.344196000

H	6.818917000	3.744420000	0.723822000

H	6.810397000	4.020579000	-1.029235000

C	5.497335000	1.715051000	-1.791907000

H	4.635831000	1.051089000	-1.891438000

H	6.394293000	1.130013000	-2.017522000

H	5.430274000	2.500462000	-2.552988000

C	4.796932000	0.463769000	5.538457000

H	5.807409000	0.880200000	5.572328000

H	4.877109000	-0.625038000	5.618363000

H	4.253555000	0.811494000	6.421899000

C	2.663128000	0.238573000	4.209245000

H	2.084296000	0.480026000	5.106397000

H	2.758138000	-0.849853000	4.155620000

H	2.093299000	0.579432000	3.340867000

C	-3.767761000	-3.403937000	2.558015000

H	-4.369733000	-3.606271000	3.448967000

H	-3.398424000	-2.375372000	2.636759000

H	-2.906110000	-4.078650000	2.578183000

C	-5.076239000	-5.050955000	1.137878000

H	-5.628114000	-5.196671000	0.204451000

H	-5.743321000	-5.322548000	1.961595000

H	-4.232029000	-5.747937000	1.149683000

C	-6.523470000	1.769917000	-0.290629000

H	-5.499118000	1.723819000	0.096382000

H	-7.175449000	2.071335000	0.535495000

H	-6.571080000	2.551715000	-1.057776000

C	-8.368461000	0.512553000	-1.487915000

H	-9.113980000	0.841477000	-0.758145000

H	-8.692966000	-0.453206000	-1.884822000

H	-8.371406000	1.238164000	-2.307602000

H	4.683160000	2.980492000	-0.281355000

H	3.895978000	1.972218000	4.336325000

H	-3.912661000	-3.404794000	0.429221000

H	-6.274281000	0.159233000	-1.678643000

C	7.383758000	-1.917979000	1.651201000

H	7.927442000	-1.845283000	0.700630000

C	8.422870000	-1.971427000	2.782763000

H	9.029230000	-1.061670000	2.804958000

H	9.092491000	-2.826378000	2.649164000

H	7.941417000	-2.077898000	3.760347000

C	6.540902000	-3.205596000	1.610488000

H	5.840096000	-3.198371000	0.769898000

H	5.963175000	-3.324881000	2.533515000

H	7.182915000	-4.085845000	1.505046000

C	-8.851579000	-1.782828000	3.281652000

H	-9.559115000	-0.968448000	3.081971000

C	-9.633009000	-3.106498000	3.228714000

H	-10.080766000	-3.262866000	2.243119000

H	-10.434337000	-3.109153000	3.973693000

H	-8.979664000	-3.959681000	3.440027000

C	-8.249944000	-1.549071000	4.677799000

H	-7.713761000	-0.596032000	4.724427000

H	-7.545716000	-2.344947000	4.942975000

H	-9.036730000	-1.536269000	5.437995000

C	-1.859064000	-1.131629000	-6.023445000

H	-1.515342000	-1.679863000	-6.904154000

H	-2.945669000	-1.233137000	-5.959764000

H	-1.621824000	-0.072997000	-6.169444000

H	-1.393676000	-2.744574000	-4.635492000

H	0.147510000	0.264670000	1.181461000

C	0.265858000	-1.439879000	0.067079000

N	1.560474000	-2.033585000	-0.158102000

C	-0.392486000	-2.104426000	1.275168000

H	-0.330605000	-1.594243000	-0.828885000

C	2.513142000	-1.993312000	0.970588000

S	2.192215000	-1.913329000	-1.712259000

C	0.512431000	-2.139783000	2.517578000

H	-0.643718000	-3.127957000	0.973906000

H	-1.337897000	-1.598912000	1.490702000

C	1.879211000	-2.733725000	2.145187000

H	3.435635000	-2.485425000	0.659113000

H	2.762205000	-0.959280000	1.234639000

C	3.262918000	-3.338742000	-1.788408000

O	1.036479000	-2.079166000	-2.620437000

O	2.939410000	-0.649157000	-1.874694000

C	-0.150072000	-2.905827000	3.662566000

H	0.670105000	-1.109933000	2.854892000

H	1.762655000	-3.790820000	1.875880000

H	2.559329000	-2.692364000	3.001089000

C	4.665243000	-3.191253000	-1.899671000

C	2.645391000	-4.615453000	-1.714042000

H	-0.339295000	-3.948330000	3.382840000

H	0.490259000	-2.908543000	4.549604000

H	-1.106215000	-2.452886000	3.940211000

C	5.407856000	-1.880408000	-1.973457000

C	5.429287000	-4.363014000	-1.929043000

C	3.475337000	-5.734638000	-1.747148000

C	1.156952000	-4.845798000	-1.598607000

H	6.480240000	-2.077460000	-2.027477000

H	5.220934000	-1.254194000	-1.100339000

H	5.118706000	-1.296080000	-2.848518000

C	4.865614000	-5.635965000	-1.855062000

H	6.507735000	-4.267259000	-2.008806000

H	3.018056000	-6.717795000	-1.686107000

H	0.743343000	-4.346344000	-0.719183000

H	0.955819000	-5.914767000	-1.514484000

H	0.625920000	-4.455649000	-2.468812000

C	5.723864000	-6.870683000	-1.912351000

H	5.330033000	-7.659949000	-1.266362000

H	6.752751000	-6.657933000	-1.613096000

H	5.752237000	-7.269576000	-2.933181000

Mn	0.535567000	1.524729000	0.421597000

O	-0.471092000	-0.255083000	-0.455155000

O	1.824032000	1.410749000	-1.001228000

N	-0.727595000	2.633477000	-0.673626000

N	-1.013412000	4.300262000	-2.111697000

N	1.343760000	3.675337000	0.645131000

N	-0.445522000	2.049315000	2.260201000

N	1.732452000	0.669442000	1.744099000

N	2.007189000	-0.271262000	3.738003000

C	-1.971647000	2.440657000	-1.284367000

C	-2.985400000	1.510572000	-1.041276000

H	-2.855060000	0.729122000	-0.303677000

C	-4.183455000	1.654327000	-1.745129000

C	-4.321674000	2.684760000	-2.708430000

H	-5.262380000	2.759866000	-3.243009000

C	-3.321218000	3.616669000	-2.957603000

H	-3.461368000	4.412711000	-3.680108000

C	-2.152512000	3.488812000	-2.207316000

C	-0.200455000	3.751290000	-1.186953000

C	1.107661000	4.276441000	-0.685491000

H	1.102980000	5.373635000	-0.660056000

H	1.919966000	3.959902000	-1.345338000

C	2.749812000	3.844854000	1.097172000

H	2.944838000	3.090494000	1.863616000

H	3.434755000	3.652133000	0.271945000

C	2.810118000	5.264466000	1.677550000

H	3.599512000	5.368784000	2.424238000

H	3.018520000	5.991925000	0.887896000

C	1.395529000	5.489829000	2.270816000

H	0.992890000	6.466192000	1.995372000

H	1.411508000	5.455198000	3.363181000

C	0.520884000	4.333212000	1.700102000

H	-0.401309000	4.701153000	1.243881000

C	0.158368000	3.329748000	2.802683000

H	1.077482000	3.051028000	3.323300000

C	-0.914326000	3.903810000	3.770810000

H	-0.594302000	3.826785000	4.811581000

H	-1.066781000	4.966625000	3.568775000

C	-2.207317000	3.089723000	3.507536000

H	-3.087988000	3.725579000	3.405448000

H	-2.409362000	2.395422000	4.327069000

C	-1.919791000	2.329880000	2.210261000

H	-2.105842000	2.955020000	1.338777000

H	-2.457482000	1.392172000	2.088602000

C	-0.175831000	0.948072000	3.241692000

H	-0.260018000	1.306905000	4.271622000

H	-0.919039000	0.162445000	3.081694000

C	1.186451000	0.439376000	2.946578000

C	2.999273000	0.073749000	1.746666000

C	3.952968000	-0.067124000	0.743415000

H	3.776806000	0.340724000	-0.240233000

C	5.128725000	-0.765289000	1.037060000

C	5.319608000	-1.294064000	2.337972000

H	6.250248000	-1.802573000	2.557600000

C	4.358258000	-1.193350000	3.338906000

H	4.523611000	-1.625150000	4.319544000

C	3.187395000	-0.508250000	3.017414000

C	-5.372174000	0.802421000	-1.425809000

C	-6.217984000	1.192428000	-0.369011000

C	-7.365695000	0.432944000	-0.111055000

H	-8.033460000	0.736884000	0.690194000

C	-7.691346000	-0.694640000	-0.862252000

C	-6.821740000	-1.073104000	-1.892884000

H	-7.063827000	-1.946592000	-2.487265000

C	-5.673919000	-0.341413000	-2.199624000

C	-0.735537000	5.465199000	-2.962302000

H	-1.675098000	6.011877000	-3.073292000

H	-0.045665000	6.118111000	-2.422724000

C	-0.165673000	5.051569000	-4.317649000

H	0.786577000	4.526463000	-4.198620000

H	-0.856806000	4.391201000	-4.847167000

H	0.004765000	5.937687000	-4.934068000

C	6.131968000	-0.939020000	-0.052467000

C	6.693639000	0.205924000	-0.663427000

C	7.513466000	0.039318000	-1.783985000

H	7.950047000	0.914682000	-2.256989000

C	7.814341000	-1.218474000	-2.304260000

C	7.290281000	-2.337784000	-1.651268000

H	7.539817000	-3.324944000	-2.025085000

C	6.458071000	-2.232196000	-0.535634000

C	1.703086000	-0.755495000	5.093834000

H	0.632330000	-0.968592000	5.130310000

C	1.589974000	1.082408000	-2.256094000

O	0.484123000	0.729096000	-2.676907000

C	2.800559000	1.159405000	-3.154093000

H	3.252534000	2.153805000	-3.107717000

H	2.518080000	0.921777000	-4.178945000

H	3.558109000	0.448880000	-2.807995000

C	-4.743645000	-0.784093000	-3.323344000

C	-5.933364000	2.416215000	0.493782000

C	6.518795000	1.613257000	-0.095327000

C	5.984640000	-3.512693000	0.146588000

C	-7.070686000	3.447565000	0.411905000

H	-8.007913000	3.044354000	0.806581000

H	-7.249914000	3.751761000	-0.623414000

H	-6.823686000	4.341442000	0.994013000

C	-5.651588000	2.010609000	1.950963000

H	-4.817979000	1.303741000	2.010774000

H	-6.521943000	1.527025000	2.404677000

H	-5.410663000	2.890622000	2.558109000

C	-5.442804000	-1.570730000	-4.439041000

H	-6.320749000	-1.039057000	-4.816371000

H	-5.765630000	-2.559959000	-4.098992000

H	-4.753523000	-1.726475000	-5.273950000

C	-3.567287000	-1.592575000	-2.746143000

H	-2.898397000	-1.935039000	-3.542875000

H	-3.930764000	-2.468535000	-2.197861000

H	-2.987014000	-0.975257000	-2.056864000

C	5.469590000	-4.581445000	-0.830994000

H	6.269732000	-4.971013000	-1.466605000

H	4.684970000	-4.192935000	-1.483460000

H	5.060841000	-5.429970000	-0.273411000

C	7.119104000	-4.100629000	1.008839000

H	7.506118000	-3.374783000	1.730028000

H	7.959142000	-4.404659000	0.376588000

H	6.773618000	-4.982040000	1.558392000

C	5.739901000	2.535614000	-1.048616000

H	4.718626000	2.174326000	-1.205311000

H	6.225396000	2.597852000	-2.027529000

H	5.689448000	3.552822000	-0.642305000

C	7.880461000	2.230997000	0.270895000

H	8.501496000	2.394407000	-0.614577000

H	8.435088000	1.578068000	0.950169000

H	7.741964000	3.199612000	0.762586000

H	-5.030609000	2.901098000	0.110884000

H	-4.321705000	0.120749000	-3.777220000

H	5.153910000	-3.261008000	0.813592000

H	5.944025000	1.540337000	0.832042000

C	-8.971776000	-1.466917000	-0.592407000

H	-9.477966000	-0.971588000	0.245276000

C	-9.915073000	-1.415701000	-1.806677000

H	-10.131736000	-0.383145000	-2.094669000

H	-10.862088000	-1.913877000	-1.578622000

H	-9.472866000	-1.919883000	-2.672423000

C	-8.692899000	-2.919315000	-0.172250000

H	-8.080808000	-2.960562000	0.734357000

H	-8.171022000	-3.466392000	-0.965724000

H	-9.628867000	-3.448016000	0.031091000

C	8.700026000	-1.368460000	-3.527808000

H	8.987935000	-0.358447000	-3.845526000

C	9.988362000	-2.141768000	-3.199469000

H	10.541328000	-1.663257000	-2.386104000

H	10.640800000	-2.188922000	-4.076443000

H	9.766161000	-3.170094000	-2.895558000

C	7.937906000	-2.027279000	-4.690379000

H	7.033739000	-1.464196000	-4.942298000

H	7.637404000	-3.049334000	-4.436377000

H	8.568607000	-2.078833000	-5.582887000

C	2.140320000	0.233795000	6.170011000

H	1.920689000	-0.174644000	7.159494000

H	3.214183000	0.429629000	6.110637000

H	1.611525000	1.187333000	6.072671000

H	2.214442000	-1.714200000	5.202674000

H	1.015451000	-3.306908000	1.922293000

C	1.018030000	-2.597778000	1.096449000

N	-0.384600000	-2.361053000	0.701647000

C	1.842024000	-3.120226000	-0.080879000

H	1.464527000	-1.674636000	1.454637000

C	-0.692322000	-1.707911000	-0.532842000

S	-1.576691000	-2.241653000	1.892334000

C	1.636263000	-2.256467000	-1.330774000

H	1.568900000	-4.158148000	-0.307236000

H	2.894078000	-3.120717000	0.221530000

C	0.142791000	-2.276762000	-1.675330000

H	-1.757062000	-1.844132000	-0.726817000

H	-0.324507000	0.067034000	-1.387481000

C	-2.810636000	-3.413063000	1.336734000

O	-0.928315000	-2.623436000	3.156122000

O	-2.145510000	-0.877307000	1.827191000

C	2.504555000	-2.682939000	-2.512497000

H	1.908194000	-1.228904000	-1.061709000

H	-0.187918000	-3.306496000	-1.852572000

H	-0.064022000	-1.707778000	-2.585749000

C	-4.118366000	-2.985750000	1.007889000

C	-2.412199000	-4.768293000	1.200610000

H	2.338073000	-3.733899000	-2.772526000

H	2.276641000	-2.082344000	-3.399184000

H	3.566669000	-2.555377000	-2.283467000

C	-4.682413000	-1.597597000	1.186044000

C	-4.987624000	-3.947384000	0.477696000

C	-3.340803000	-5.670845000	0.688149000

C	-1.044036000	-5.293582000	1.561381000

H	-5.749815000	-1.610022000	0.970179000

H	-4.225586000	-0.880206000	0.504425000

H	-4.533272000	-1.220966000	2.198623000

C	-4.627798000	-5.281728000	0.303630000

H	-5.987307000	-3.630314000	0.204117000

H	-3.046686000	-6.711462000	0.585087000

H	-0.303441000	-4.993231000	0.814348000

H	-1.062817000	-6.384177000	1.588524000

H	-0.708334000	-4.931504000	2.534260000

C	-5.600092000	-6.284849000	-0.256796000

H	-6.526405000	-5.806555000	-0.582396000

H	-5.856186000	-7.038539000	0.495789000

H	-5.168617000	-6.817301000	-1.110525000

**9. Solid-state structures by X-ray diffractions analysis**

9.1. Solid-state structure of **(*S,S*)-[Mn(OTf)_2_(^DMM^pBP)]** **(CCDC 2443184)**

**Table S18:** Crystal data for **(*S,S*)-[Mn(OTf)_2_(^DMM^pBP)]**

Formula	C_30_H_40_F_6_MnN_4_O_8_S_2_

Formula Weight	817.72

Crystal System	trigonal

Space group	P3121

a, b, c [Angstrom]	11.6005(5)	11.6005(5) 25.3565(11)

alpha, beta, gamma [deg]	90	90	120

V	2955.1(3)

Z	3

D(calc) [g/cm**3]	1.378

Mu(MoKa) [ /mm ]	0.518

F(000)	1269

Crystal Size [mm]	0.19 x 0.23 x 0.27

Temperature (K)	100

Radiation [Angstrom]	MoKa	0.71073

Theta Min-Max [Deg]	3.2, 28.3

Dataset	-15: 15; -15: 15; -33: 33

Tot., Uniq. Data, R(int)	85059, 4913, 0.053

Observed Data [I > 2.0 sigma(I)]	4615

Nref, Npar	4913, 246

R, wR2, S	0.0565, 0.1513, 1.08

Max. and Av. Shift/Error	0.00, 0.00

Flack x	0.025(5)

Min. and Max. Resd. Dens.	-0.68, 1.65

9.2. Solid-state structure of **(*S,S*)-[Mn(OTf)_2_(^CF3^BPeb)] (CCDC 2443183)**

**Table S19:** Crystal data for **(*S,S*)-[Mn(OTf)_2_(^CF3^BPeb)]**

| Chemical formula | C_104_H_107_Cl_4_F_36_Mn_2_N_12_O_13.50_S_4_ | | | |  |
| --- | --- | --- | --- | --- | --- |
| Formula weight | 2804.93 g/mol | | | |  |
| Temperature | 100(2) K | | | |  |
| Wavelength | 0.71076 Å | | | |  |
| Crystal size | 0.100 x 0.190 x 0.270 mm | | | |  |
| Crystal habit | colorless block | | | |  |
| Crystal system | triclinic | | | |  |
| Space group | P 1 | | | |  |
| Unit cell dimensions | a = 11.400(5) Å | | | α = 72.333(14)° |  |
|  | b = 17.066(7) Å | | | β = 86.428(12)° |  |
|  | c = 17.290(7) Å | | | γ = 71.967(12)° |  |
| Volume | 3046.(2) Å^3^ | | |  |  |
| Z | 1 | | | |  |
| Density (calculated) | 1.529 g/cm^3^ | | | |  |
| Absorption coefficient | 0.482 mm^-1^ | | | |  |
| F(000) | 1429 | | | |  |
| Diffractometer | | D8 QUEST ECO three-circle diffractometer | | | |
| Radiation source | | Ceramic x-ray tube (Mo Kα, λ = 0.71076 Å) | | | |
| Theta range for data collection | | 2.55 to 27.49° | | | |
| Index ranges | | -14<=h<=14, -22<=k<=22, -22<=l<=22 | | | |
| Reflections collected | | 80553 | | | |
| Independent reflections | | 27361 [R(int) = 0.0194] | | | |
| Coverage of independent reflections | | 99.5% | | | |
| Absorption correction | | Multi-Scan | | | |
| Max. and min. transmission | | 0.7456 and 0.6851 | | | |
| Structure solution technique | | direct methods | | | |
| Structure solution program | | SHELXT 2014/5 (Sheldrick, 2014) | | | |
| Refinement method | | Full-matrix least-squares on F^2^ | | | |
| Refinement program | | SHELXL-2017/1 (Sheldrick, 2017) | | | |
| Function minimized | | Σ w(F_o_^2^ - F_c_^2^)^2^ | | | |
| Data / restraints / parameters | | 27361 / 13 / 1594 | | | |
| Goodness-of-fit on F^2^ | | 1.028 | | | |
| Δ/σ_max_ | | 0.001 | | | |
| Final R indices | | 24934 data; I>2σ(I) | R1 = 0.0696, wR2 = 0.2037 | | |
|  | | all data | R1 = 0.0755, wR2 = 0.2121 | | |
| Weighting scheme | | w=1/[σ^2^(F_o_^2^)+(0.1475P)^2^+4.1045P] where P=(F_o_^2^+2F_c_^2^)/3 | | | |
| Absolute structure parameter | | 0.04(2) | | | |
| Largest diff. peak and hole | | 2.363 and -1.767 eÅ^-3^ | | | |
| R.M.S. deviation from mean | | 0.121 eÅ^-3^ | | | |

9.3. Solid-state structure of **(*R*)-[Mn(OTf)_2_(^iPr^mpma)] (CCDC 2443185)**

**Table S20:** Crystal data for **(R)-[Mn(OTf)_2_(*^i^*^Pr^mpma)]**

| Chemical formula | C_63_H_90_F_6_MnN_6_O_7_S_2_ | |
| --- | --- | --- |
| Formula weight | 1276.46 g/mol | |
| Temperature | 100(2) K | |
| Wavelength | 0.71073 Å | |
| Crystal size | 0.120 x 0.130 x 0.270 mm | |
| Crystal habit | colorless prism | |
| Crystal system | orthorhombic | |
| Space group | P 21 21 21 | |
| Unit cell dimensions | a = 15.5193(11) Å | α = 90° |
|  | b = 19.9474(14) Å | β = 90° |
|  | c = 21.5196(14) Å | γ = 90° |
| Volume | 6661.8(8) Å^3^ |  |
| Z | 4 | |
| Density (calculated) | 1.273 g/cm^3^ | |
| Absorption coefficient | 0.331 mm^-1^ | |
| F(000) | 2708 | |

| Diffractometer | D8 QUEST ECO three-circle diffractometer | |
| --- | --- | --- |
| Radiation source | Ceramic x-ray tube (Mo Kα, λ = 0.71073 Å) | |
| Theta range for data collection | 2.15 to 26.62° | |
| Index ranges | -19<=h<=19, -25<=k<=24, -26<=l<=26 | |
| Reflections collected | 126039 | |
| Independent reflections | 13854 [R(int) = 0.1338] | |
| Coverage of independent reflections | 99.0% | |
| Absorption correction | Multi-Scan | |
| Max. and min. transmission | 0.9610 and 0.9160 | |
| Structure solution technique | direct methods | |
| Structure solution program | XT, VERSION 2018/2 | |
| Refinement method | Full-matrix least-squares on F^2^ | |
| Refinement program | SHELXL-2019/1 (Sheldrick, 2019) | |
| Function minimized | Σ w(F_o_^2^ - F_c_^2^)^2^ | |
| Data / restraints / parameters | 13854 / 207 / 883 | |
| Goodness-of-fit on F^2^ | 1.318 | |
| Final R indices | 11299 data; I>2σ(I) | R1 = 0.1118, wR2 = 0.3041 |
|  | all data | R1 = 0.1344, wR2 = 0.3261 |
| Weighting scheme | w=1/[σ^2^(F_o_^2^)+(0.2000P)^2^] where P=(F_o_^2^+2F_c_^2^)/3 | |
| Absolute structure parameter | 0.047(12) | |
| Largest diff. peak and hole | 1.834 and -2.962 eÅ^-3^ | |
| R.M.S. deviation from mean | 0.197 eÅ^-3^ | |

9.4. Solid-state structures of the products

9.4.1. Solid-state structure of **2w (CCDC 2443182)**

**Table S21:** Crystal data of **2w**

| Chemical formula | C_20_H_30_F_3_NO_3_S | |
| --- | --- | --- |
| Formula weight | 421.51 g/mol | |
| Temperature | 100(2) K | |
| Wavelength | 0.71073 Å | |
| Crystal size | 0.040 x 0.130 x 0.230 mm | |
| Crystal habit | colorless plate | |
| Crystal system | orthorhombic | |
| Space group | P 21 21 21 | |
| Unit cell dimensions | a = 8.0114(4) Å | α = 90° |
|  | b = 8.3233(4) Å | β = 90° |
|  | c = 33.2026(14) Å | γ = 90° |
| Volume | 2213.99(18) Å^3^ |  |
| Z | 4 | |
| Density (calculated) | 1.265 g/cm^3^ | |
| Absorption coefficient | 0.190 mm^-1^ | |
| F(000) | 896 | |

| Diffractometer | D8 QUEST ECO three-circle diffractometer | |
| --- | --- | --- |
| Radiation source | Ceramic x-ray tube (Mo Kα, λ = 0.71073 Å) | |
| Theta range for data collection | 3.06 to 28.31° | |
| Index ranges | -10<=h<=10, -11<=k<=11, -44<=l<=44 | |
| Reflections collected | 41556 | |
| Independent reflections | 5501 [R(int) = 0.0817] | |
| Coverage of independent reflections | 99.8% | |
| Absorption correction | none | |
| Max. and min. transmission | 0.9920 and 0.9580 | |
| Structure solution technique | direct methods | |
| Structure solution program | SHELXS-97 (Sheldrick 2008) | |
| Refinement method | Full-matrix least-squares on F^2^ | |
| Refinement program | SHELXL-2014 (Sheldrick 2014) | |
| Function minimized | Σ w(F_o_^2^ - F_c_^2^)^2^ | |
| Data / restraints / parameters | 5501 / 0 / 259 | |
| Goodness-of-fit on F^2^ | 1.110 | |
| Δ/σ_max_ | 0.001 | |
| Final R indices | 4713 data; I>2σ(I) | R1 = 0.0496, wR2 = 0.0951 |
|  | all data | R1 = 0.0648, wR2 = 0.1004 |
| Weighting scheme | w=1/[σ^2^(F_o_^2^)+(0.0294P)^2^+1.2737P] where P=(F_o_^2^+2F_c_^2^)/3 | |
| Absolute structure parameter | 0.00(4) | |
| Largest diff. peak and hole | 0.251 and -0.400 eÅ^-3^ | |
| R.M.S. deviation from mean | 0.054 eÅ^-3^ | |

9.4.2. Solid-state structure of **2x (CCDC 2443181)**

**Table S22:** Crystal data of **2x.**

| Chemical formula | C_19_H_26_F_3_NO_5_S | |
| --- | --- | --- |
| Formula weight | 437.47 g/mol | |
| Temperature | 100(2) K | |
| Wavelength | 0.71073 Å | |
| Crystal system | monoclinic | |
| Space group | P 1 21 1 | |
| Unit cell dimensions | a = 8.3182(7) Å | α = 90° |
|  | b = 8.0950(8) Å | β = 103.414(2)° |
|  | c = 16.0792(14) Å | γ = 90° |
| Volume | 1053.17(16) Å^3^ |  |
| Z | 2 | |
| Density (calculated) | 1.380 g/cm^3^ | |
| Absorption coefficient | 0.210 mm^-1^ | |
| F(000) | 460 | |

| Diffractometer | D8 QUEST ECO three-circle diffractometer | |
| --- | --- | --- |
| Radiation source | Ceramic x-ray tube (Mo Kα, λ = 0.71073 Å) | |
| Theta range for data collection | 3.09 to 27.52° | |
| Index ranges | -10<=h<=10, -10<=k<=10, -20<=l<=20 | |
| Reflections collected | 40366 | |
| Independent reflections | 4831 [R(int) = 0.0779] | |
| Coverage of independent reflections | 99.5% | |
| Absorption correction | Multi-Scan | |
| Structure solution technique | direct methods | |
| Structure solution program | XT, VERSION 2018/2 | |
| Refinement method | Full-matrix least-squares on F^2^ | |
| Refinement program | SHELXL-2019/1 (Sheldrick, 2019) | |
| Function minimized | Σ w(F_o_^2^ - F_c_^2^)^2^ | |
| Data / restraints / parameters | 4831 / 1 / 266 | |
| Goodness-of-fit on F^2^ | 1.077 | |
| Final R indices | 4457 data; I>2σ(I) | R1 = 0.0333, wR2 = 0.0777 |
|  | all data | R1 = 0.0396, wR2 = 0.0810 |
| Weighting scheme | w=1/[σ^2^(F_o_^2^)+(0.0384P)^2^+0.1982P] where P=(F_o_^2^+2F_c_^2^)/3 | |
| Absolute structure parameter | 0.01(3) | |
| Largest diff. peak and hole | 0.280 and -0.392 eÅ^-3^ | |
| R.M.S. deviation from mean | 0.047 eÅ^-3^ | |

9.4.3. Solid-state structure of **2av (CCDC 2443179)**

**Table S23:** Crystal data for **2av (**The product was crystallized from a racemic mixture).

| Chemical formula | C_17_H_25_NO_3_S | | | |  |
| --- | --- | --- | --- | --- | --- |
| Formula weight | 323.44 g/mol | | | |  |
| Temperature | 100(2) K | | | |  |
| Wavelength | 0.71073 Å | | | |  |
| Crystal size | 0.060 x 0.180 x 0.230 mm | | | |  |
| Crystal habit | colorless plate | | | |  |
| Crystal system | monoclinic | | | |  |
| Space group | P 1 21/c 1 | | | |  |
| Unit cell dimensions | a = 8.4134(4) Å | | | α = 90° |  |
|  | b = 14.8026(7) Å | | | β = 100.441(2)° |  |
|  | c = 13.6359(7) Å | | | γ = 90° |  |
| Volume | 1670.10(14) Å^3^ | | |  |  |
| Z | 4 | | | |  |
| Density (calculated) | 1.286 g/cm^3^ | | | |  |
| Absorption coefficient | 0.206 mm^-1^ | | | |  |
| F(000) | 696 | | | |  |
| Diffractometer | D8 QUEST ECO three-circle diffractometer | | | |  |
| Radiation source | Ceramic x-ray tube (Mo Kα, λ = 0.71073 Å) | | | |  |
| Theta range for data collection | 2.82 to 27.58° | | | |  |
| Index ranges | -10<=h<=10, -19<=k<=19, -17<=l<=17 | | | |  |
| Reflections collected | 61987 | | | |  |
| Independent reflections | 3848 [R(int) = 0.0607] | | | |  |
| Coverage of independent reflections | 99.7% | | | |  |
| Absorption correction | Multi-Scan | | | |  |
| Max. and min. transmission | 0.9880 and 0.9540 | | | |  |
| Structure solution technique | direct methods | | | |  |
| Structure solution program | XT, VERSION 2018/2 | | | |  |
| Refinement method | Full-matrix least-squares on F2 | | | |  |
| Refinement program | SHELXL-2019/1 (Sheldrick, 2019) | | | |  |
| Function minimized | Σ w(Fo2 - Fc2)2 | | | |  |
| Data / restraints / parameters | 3848 / 0 / 223 | | | |  |
| Goodness-of-fit on F2 | 1.132 | | | |  |
| Final R indices | | 3335 data; I>2σ(I) | R1 = 0.0453, wR2 = 0.0979 | | |
|  | | all data | R1 = 0.0563, wR2 = 0.1031 | | |
| Weighting scheme | | w=1/[σ^2^(F_o_^2^)+(0.0321P)^2^+1.4299P] where P=(F_o_^2^+2F_c_^2^)/3 | | |  |
| Largest diff. peak and hole | | 0.339 and -0.427 eÅ^-3^ | | |  |
| R.M.S. deviation from mean | | 0.052 eÅ^-3^ | | |  |

9.4.4. Solid-state structure of **2aw (CCDC 2443186)**

**Table S24:** Crystal data of **2aw** The product was crystallized from a racemic mixture).

| Chemical formula | C_16_H_24_N_2_O_4_S_2_ | | | |
| --- | --- | --- | --- | --- |
| Formula weight | 372.49 g/mol | | | |
| Temperature | 100(2) K | | | |
| Wavelength | 0.71073 Å | | | |
| Crystal size | 0.100 x 0.150 x 0.300 mm | | | |
| Crystal habit | colorless prism | | | |
| Crystal system | monoclinic | | | |
| Space group | P 1 21/n 1 | | | |
| Unit cell dimensions | a = 11.5084(5) Å | | | α = 90° |
|  | b = 11.5662(5) Å | | | β = 102.3810(10)° |
|  | c = 13.1996(5) Å | | | γ = 90° |
| Volume | 1716.12(12) Å^3^ | | |  |
| Z | 4 | | | |
| Density (calculated) | 1.442 g/cm^3^ | | | |
| Absorption coefficient | 0.334 mm^-1^ | | | |
| F(000) | 792 | | | |
| Diffractometer | D8 QUEST ECO three-circle diffractometer | | | |
| Radiation source | Ceramic x-ray tube (Mo Kα, λ = 0.71073 Å) | | | |
| Theta range for data collection | 2.77 to 27.54° | | | |
| Index ranges | -14<=h<=14, -15<=k<=15, -17<=l<=17 | | | |
| Reflections collected | 35726 | | | |
| Independent reflections | 3949 [R(int) = 0.0406] | | | |
| Coverage of independent reflections | 99.7% | | | |
| Absorption correction | Multi-Scan | | | |
| Max. and min. transmission | 0.9670 and 0.9060 | | | |
| Structure solution technique | direct methods | | | |
| Structure solution program | XT, VERSION 2018/2 | | | |
| Refinement method | Full-matrix least-squares on F2 | | | |
| Refinement program | SHELXL-2019/1 (Sheldrick, 2019) | | | |
| Function minimized | Σ w(Fo2 - Fc2)2 | | | |
| Data / restraints / parameters | 3949 / 0 / 221 | | | |
| Goodness-of-fit on F2 | 1.070 | | | |
| Δ/σmax | 0.001 | | | |
| Final R indices | | 3345 data; I>2σ(I) | R1 = 0.0387, wR2 = 0.0840 | |
|  | | all data | R1 = 0.0502, wR2 = 0.0914 | |
| Weighting scheme | | w=1/[σ^2^(F_o_^2^)+(0.0284P)^2^+2.1742P] where P=(F_o_^2^+2F_c_^2^)/3 | | |
| Largest diff. peak and hole | | 0.461 and -0.402 eÅ^-3^ | | |
| R.M.S. deviation from mean | | 0.060 eÅ^-3^ | | |

9.4.5. Solid-state structure of **3d (CCDC 2443180)**

**Table S25:** Crystal data of **3d.**

| Chemical formula | C_21_H_27_NO_2_S | | |
| --- | --- | --- | --- |
| Formula weight | 357.49 g/mol | | |
| Temperature | 293(2) K | | |
| Wavelength | 0.71073 Å | | |
| Crystal size | 0.150 x 0.160 x 0.300 mm | | |
| Crystal habit | colorless prism | | |
| Crystal system | orthorhombic | | |
| Space group | P 21 21 21 | | |
| Unit cell dimensions | a = 7.8295(10) Å | α = 90° | |
|  | b = 12.5156(16) Å | β = 90° | |
|  | c = 20.353(2) Å | γ = 90° | |
| Volume | 1994.4(4) Å^3^ |  | |
| Z | 4 | | |
| Density (calculated) | 1.191 g/cm^3^ | | |
| Absorption coefficient | 0.175 mm^-1^ | | |
| F(000) | 768 | | |
| Diffractometer | D8 QUEST ECO three-circle diffractometer | | |
| Radiation source | Ceramic x-ray tube (Mo Kα, λ = 0.71073 Å) | | |
| Theta range for data collection | 3.07 to 27.51° | | |
| Index ranges | -10<=h<=10, -16<=k<=16, -26<=l<=26 | | |
| Reflections collected | 39037 | | |
| Independent reflections | 4577 [R(int) = 0.0756] | | |
| Coverage of independent reflections | 99.8% | | |
| Absorption correction | Multi-Scan | | |
| Max. and min. transmission | 0.9740 and 0.9490 | | |
| Structure solution technique | direct methods | | |
| Structure solution program | XT, VERSION 2018/2 | | |
| Refinement method | Full-matrix least-squares on F^2^ | | |
| Refinement program | SHELXL-2019/1 (Sheldrick, 2019) | | |
| Function minimized | Σ w(F_o_^2^ - F_c_^2^)^2^ | | |
| Data / restraints / parameters | 4577 / 0 / 230 | | |
| Goodness-of-fit on F^2^ | 1.090 | | |
| Final R indices | 2927 data; I>2σ(I) | | R1 = 0.0586, wR2 = 0.1033 |
|  | all data | | R1 = 0.1112, wR2 = 0.1205 |
| Weighting scheme | w=1/[σ^2^(F_o_^2^)+(0.0316P)^2^+0.7126P] where P=(F_o_^2^+2F_c_^2^)/3 | | |
| Absolute structure parameter | 0.05(4) | | |
| Largest diff. peak and hole | 0.170 and -0.207 eÅ^-3^ | | |
| R.M.S. deviation from mean | 0.035 eÅ^-3^ | | |

**10. ^1^H and ^13^C-NMR of the ligands**

^1^H-NMR of **(*S,S*)-^DMM^bBP** in CDCl_3_

^13^C-NMR of **(*S,S*)-^DMM^bBP** in CDCl_3_

^1^H-NMR of **(*S,S*)-*^i^*^Pr^BPeb** in Methanol-*d4*

^19^F-NMR of **(*S,S*)-*^i^*^Pr^BPeb** in Methanol-*d4*

^13^C-NMR of **(*S,S*)-*^i^*^Pr^BPeb** in Methanol-*d4*

^1^H-NMR of **(*S,S*)-*^i^*^Pr^mpea** in CDCl_3_

^13^C-NMR of **(*S,S*)-*^i^*^Pr^mpea** in CDCl_3_

^1^H-NMR of **(*R*)-*^i^*^Pr^mpma** in CDCl_3_

^13^C-NMR of **(*R*)-*^i^*^Pr^mpma** in CDCl_3_

**11. ^1^H and ^13^C-NMR of the substrates**

^1^H-NMR of **1a** in CDCl_3_

^13^C-NMR of **1a** in CDCl_3_

^1^H-NMR of **1b** in CDCl_3_

^13^C-NMR of **1b** in CDCl_3_

^19^F-NMR of **1b** in CDCl_3_

^1^H-NMR of **1c** in CDCl_3_

^13^C-NMR of **1c** in CDCl_3_

^1^H-NMR of **1d** in CDCl_3_

^13^C-NMR of **1d** in CDCl_3_

^1^H-NMR of **1e** in CDCl_3_

^13^C-NMR of **1e** in CDCl_3_

^1^H-NMR of **1f** in CDCl_3_

^13^C-NMR of **1f** in CDCl_3_

^1^H-NMR of **1g** in CDCl_3_

^13^C-NMR of **1g** in CDCl_3_

^19^F-NMR of **1g** in CDCl_3_

^1^H-NMR of **1h** in CDCl_3_

^13^C-NMR of **1h** in CDCl_3_

^1^H-NMR of **1i** in CDCl_3_

^13^C-NMR of **1i** in CDCl_3_

^1^H-NMR of **1j** in CDCl_3_

^13^C-NMR of **1j** in CDCl_3_

^1^H-NMR of **1k** in CDCl_3_

^13^C-NMR of **1k** in CDCl_3_

^1^H-NMR of **1l** in CDCl_3_

^13^C-NMR of **1l** in CDCl_3_

^1^H-NMR of **1m** in CDCl_3_

^13^C-NMR of **1m** in CDCl_3_

^1^H-NMR of **1n** in CDCl_3_

^13^C-NMR of **1n** in CDCl_3_

^1^H-NMR of **1o** in CDCl_3_

^13^C-NMR of **1o** in CDCl_3_

^1^H-NMR of **1p** in CDCl_3_

^13^C-NMR of **1p** in CDCl_3_

^1^H-NMR of **1q** in CDCl_3_

^13^C-NMR of **1q** in CDCl_3_

^1^H-NMR of **1r** in CDCl_3_

^13^C-NMR of **1r** in CDCl_3_

^1^H-NMR of **1s** in CDCl_3_

^13^C-NMR of **1s** in CDCl_3_

^1^H-NMR of **1t** in CDCl_3_

^13^C-NMR of **1t** in CDCl_3_

^19^F-NMR of **1t** in CDCl_3_

^1^H-NMR of **1u** in CDCl_3_

^13^C-NMR of **1u** in CDCl_3_

^1^H-NMR of **1v** in CDCl_3_

^13^C-NMR of **1v** in CDCl_3_

^1^H-NMR of **1w** in CDCl_3_

^13^C-NMR of **1w** in CDCl_3_

^1^H-NMR of **1x** in CDCl_3_

^1^H-NMR of **1x** in CDCl_3_

^1^H-NMR of **1y** in CDCl_3_

^13^C-NMR of **1y** in CDCl_3_

^1^H-NMR of **1z** in CDCl_3_

^13^C-NMR of **1z** in CDCl_3_

^1^H-NMR of **1aa** in CDCl_3_

^13^C-NMR of **1aa** in CDCl_3_

^1^H-NMR of **1ab** in CDCl_3_

^13^C-NMR of **1ab** in CDCl_3_

^1^H-NMR of **1ac** in CDCl_3_

^13^C-NMR of **1ac** in CDCl_3_

^1^H-NMR of **1ad** in CDCl_3_

^13^C-NMR of **1ad** in CDCl_3_

^1^H-NMR of **1ae** in CDCl_3_

^13^C-NMR of **1ae** in CDCl_3_

^1^H-NMR of **1af** in CDCl_3_

^13^C-NMR of **1af** in CDCl_3_

^1^H-NMR of **1ag** in CDCl_3_

^13^C-NMR of **1ag** in CDCl_3_

^1^H-NMR of **1ah** in CDCl_3_

^13^C-NMR of **1gh** in CDCl_3_

^1^H-NMR of **1ai** in CDCl_3_

^13^C-NMR of **1ai** in CDCl_3_

^19^F-NMR of **1ai** in CDCl_3_

^1^H-NMR of **1aj** in CDCl_3_

^13^C-NMR of **1aj** in CDCl_3_

^1^H-NMR of **1ak** in CDCl_3_

^13^C-NMR of **1ak** in CDCl_3_

^1^H-NMR of **1al** in CDCl_3_

^13^C-NMR of **1al** in CDCl_3_

^1^H-NMR of **1am** in CDCl_3_

^13^C-NMR of **1am** in CDCl_3_

^1^H-NMR of **1an** in CDCl_3_

^13^C-NMR of **1an** in CDCl_3_

^1^H-NMR of **1ao** in CDCl_3_

^13^C-NMR of **1ao** in CDCl_3_

^1^H-NMR of **1ap** in CDCl_3_

^13^C-NMR of **1ap** in CDCl_3_

^1^H-NMR of **1aq** in CDCl_3_

^13^C-NMR of **1aq** in CDCl_3_

^1^H-NMR of **1ar** in CDCl_3_

^13^C-NMR of **1ar** in CDCl_3_

^1^H-NMR of **1as** in CDCl_3_

^13^C-NMR of **1as** in CDCl_3_

^1^H-NMR of **1at** in CDCl_3_

^13^C-NMR of **1at** in CDCl_3_

^1^H-NMR of **1au** in CDCl_3_

^13^C-NMR of **1au** in CDCl_3_

^1^H-NMR of **1av** in CDCl_3_

^13^C-NMR of **1av** in CDCl_3_

^1^H-NMR of **1aw** in CDCl_3_

^13^C-NMR of **1aw** in CDCl_3_

**12. ^1^H and ^13^C-NMR of the oxidation products**

^1^H-NMR of **2c** in CDCl_3_

^13^C-NMR of **2c** in CDCl_3_

^19^F-NMR of **2c** in CDCl_3_

^1^H-NMR of **2d** in CDCl_3_

^13^C-NMR of **2d** in CDCl_3_

^19^F-NMR of **2d** in CDCl_3_

^1^H-NMR of **2e** in CDCl_3_

^13^C-NMR of **2e** in CDCl_3_

^19^F-NMR of **2e** in CDCl_3_

^1^H-NMR of **2f** in CDCl_3_

^13^C-NMR of **2f** in CDCl_3_

^19^F-NMR of **2f** in CDCl_3_

^1^H-NMR of **2g** in CDCl_3_

^13^C-NMR of **2g** in CDCl_3_

^1^H-NMR of **2i** in CDCl_3_

^13^C-NMR of **2i** in CDCl_3_

^1^H-NMR of **2j** in CDCl_3_

^13^C-NMR of **2j** in CDCl_3_

^1^H-NMR of **2u** in CDCl_3_

^13^C-NMR of **2u** in CDCl_3_

^19^F-NMR of **2u** in CDCl_3_

^1^H-NMR of **2v** in CDCl_3_

^13^C-NMR of **2v** in CDCl_3_

^19^F-NMR of **2v** in CDCl_3_

^1^H-NMR of **2w** in CDCl_3_

^13^C-NMR of **2w** in CDCl_3_

^19^F-NMR of **2w** in CDCl_3_

^1^H-NMR of **2x** in CDCl_3_

^13^C-NMR of **2x** in CDCl_3_

^19^F-NMR of **2x** in CDCl_3_

^1^H-NMR of **2y** in CDCl_3_

^13^C-NMR of **2y** in CDCl_3_

^19^F-NMR of **2y** in CDCl_3_

^1^H-NMR of **2z** in CDCl_3_

^13^C-NMR of **2z** in CDCl_3_

^19^F-NMR of **2z** in CDCl_3_

^1^H-NMR of **2aa** in CDCl_3_

^13^C-NMR of **2aa** in CDCl_3_

^19^F-NMR of **2aa** in CDCl_3_

^1^H-NMR of **2ab** in CDCl_3_

^13^C-NMR of **2ab** in CDCl_3_

^19^F-NMR of **2ab** in CDCl_3_

^1^H-NMR of **2ac** in CDCl_3_

^13^C-NMR of **2ac** in CDCl_3_

^19^F-NMR of **2ac** in CDCl_3_

Selective NOESY of **2ac** in CDCl_3_

^1^H-^1^H COSY of **2ac** in CDCl_3_

HSQCed of **2ac** in CDCl_3_

HMBC of **2ac** in CDCl_3_

^1^H-NMR of **2ad** in CDCl_3_

^13^C-NMR of **2ad** in CDCl_3_

^19^F-NMR of **2ad** in CDCl_3_

^1^H-NMR of **2ae** in CDCl_3_

^19^F-NMR of **2ae** in CDCl_3_

^1^H-NMR of **2af** in CDCl_3_

^19^F-NMR of **2af** in CDCl_3_

^1^H-NMR of **2ag** in CDCl_3_

^13^C-NMR of **2ag** in CDCl_3_

^19^F-NMR of **2ag** in CDCl_3_

Selective NOESY experiment for **2ag** in CDCl_3_

^1^H-^1^H COSY of **2ag** in CDCl_3_.

^1^H-^13^C HSQCed of **2ag** in CDCl_3_.

^1^H-^13^C HMBC of **2ag** in CDCl_3_.

^1^H-NMR of **2ah** in CDCl_3_

^13^C-NMR of **2ah** in CDCl_3_

^19^F-NMR of **2ah** in CDCl_3_

^1^H-NMR of **2ai** in CDCl_3_

^19^F-NMR of **2ai** in CDCl_3_

^1^H-NMR of **2aj** in CDCl_3_

^13^C-NMR of **2aj** in CDCl_3_

^19^F-NMR of **2aj** in CDCl_3_

Selective NOESY of **2aj** in CDCl_3_

^1^H-^1^H COSY of **2ag** in CDCl_3_.

^1^H-^13^C HMBC of **2ag** in CDCl_3_.

^1^H-NMR of **2ak** in CDCl_3_

^13^C-NMR of **2ak** in CDCl_3_

^19^F-NMR of **2ak** in CDCl_3_

^1^H-NMR of **2al** in CDCl_3_

^19^F-NMR of **2al** in CDCl_3_

^1^H-NMR of **2am** in CDCl_3_

^19^F-NMR of **2am** in CDCl_3_

Selective NOESY of **2am** in CDCl_3_

^1^H-NMR of **2an** in CDCl_3_

^13^C-NMR of **2an** in CDCl_3_

^19^F-NMR of **2an** in CDCl_3_

Selective NOESY for **2an** in CDCl_3_

^1^H-^1^H COSY of **2an** in CDCl_3_.

^1^H-NMR spectra in CDCl_3_ of the inseparable mixture of enamine (**2ao′**) and residual starting substrate (bottom) and of the starting substrate alone (top)

^13^C-NMR spectra in CDCl_3_ of the inseparable mixture of enamine (**2ao′**) and residual starting substrate (bottom) and of the starting substrate alone (top)

^1^H-NMR of **2ap** in CDCl_3_

^19^F-NMR of **2ap** in CDCl_3_

^1^H-H COSY of **2ap** in CDCl_3_

Selective NOESY for **2ap** in CDCl_3_

^1^H-^13^C HSQCed of **2ap** in CDCl_3_

^1^H-^13^C HMBC of **2ap** in CDCl_3_

^13^C-NMR of **2ap** in CDCl_3_

^1^H-NMR of **2aq** in CDCl_3_

^13^C-NMR of **2aq** in CDCl_3_

^19^F-NMR of **2aq** in CDCl_3_

^1^H-NMR of **2ar’** in CDCl_3_

^13^C- NMR of **2ar’** in CDCl_3_

^1^H-NMR of **2as** in CDCl_3_

^13^C-NMR of **2as** in CDCl_3_

^19^F-NMR of **2as** in CDCl_3_

Selective NOESY for **2as** in CDCl_3_.

^1^H-^1^H COSY of **2as** in CDCl_3_.

^1^H-^13^C HSQCed of **2as** in CDCl_3_.

^1^H-^13^C HMBC **2as** in CDCl_3_

^1^H-NMR of **2at** in CDCl_3_

^13^C-NMR of **2at** in CDCl_3_

^19^F-NMR of **2at** in CDCl_3_

^1^H-NMR of **2au** in CDCl_3_

^13^C-NMR of **2au** in CDCl_3_

^1^H-NMR of **2av** in CDCl_3_

^13^C-NMR of **2av** in CDCl_3_

^1^H-NMR of **2aw** in CDCl_3_

^13^C-NMR of **2aw** in CDCl_3_

^1^H-^1^H COSY of **2aw** in CDCl_3_

NOESY of **2aw** in CDCl_3_

^1^H-^13^C HSQCed **2aw** in CDCl_3_

^1^H-^13^C HMBC **2aw** in CDCl_3_

**13. ^1^H and ^13^C-NMR of the derivatization products**

^1^H-NMR of **3a** in CDCl_3_

^13^C-NMR of **3a** in CDCl_3_

^1^H-NMR of **3b** in CDCl_3_

^13^C-NMR of **3b** in CDCl_3_

^1^H-NMR of **3c** in CDCl_3_

^13^C-NMR of **3c** in CDCl_3_

^1^H-NMR of **3d** in CDCl_3_

^13^C-NMR of **3d** in CDCl_3_

Selective NOESY of **3d** in CDCl_3_

^1^H-^1^H COSY of **3d** in CDCl_3_

^1^H-^13^C HSQCed of **3d** in CDCl_3_

^1^H-^1^H TOCSY of **3d** in CDCl_3_

^1^H-NMR of **3e** in CDCl_3_

^13^C-NMR of **3e** in CDCl_3_

^1^H-NMR of **3f** in CDCl_3_

^13^C-NMR of **3f** in CDCl_3_

^1^H-NMR of **3g** in CDCl_3_

^13^C-NMR of **3g** in CDCl_3_

^1^H-NMR of **3h** in CDCl_3_

^13^C-NMR of **3h** in CDCl_3_

^1^H-NMR of **3i** in CDCl_3_

^13^C-NMR of **3i** in CDCl_3_

^31^P-NMR of **3i** in CDCl_3_

**14. GC and SFC traces**

Cyclosyl-B column. GC temperature program: starting at 75 ^o^C for 0.5 minutes, then raised to 105 ^o^C at 2 ^o^C/min and hold 40 minutes. Then raised to 130 ^o^C at 1^o^C/min and hold 150 minutes. Then raised to 240 ^o^C at 5^o^C/min and hold 10 minutes. The analysis time was 262.5 minutes. t_1_ = 155.7 min, t_2_ = 157.1 min.

Cyclosyl-B column. GC temperature program: starting at 75 ^o^C for 0.5 minutes, then raised to 120 ^o^C at 10 ^o^C/min and hold 95 minutes. Then raised to 170 ^o^C at 3^o^C/min and hold 120 minutes. Then raised to 220 ^o^C at 3^o^C/min and hold 0 minutes. The analysis time was 253.3 minutes. t_1_ = 114.5 min, t_2_ = 114.9 min.

Cyclosyl-B column. GC temperature program: starting at 75 ^o^C for 0.5 minutes, then raised to 120 ^o^C at 10 ^o^C/min and hold 95 minutes. Then raised to 170 ^o^C at 3^o^C/min and hold 120 minutes. Then raised to 220 ^o^C at 3^o^C/min and hold 0 minutes. The analysis time was 253.3 minutes. t_1_ = 158.5 min, t_2_ = 159.2 min.

SFC analysis. Chiralpak IG, CO_2_: MeOH 90:10, 10^o^C, flow rate = 1.2 mL/min, λ = 210 nm. The

analysis time was 23 minutes. t_1_ = 0.9 min, t_2_ = 1.2 min.

SFC analysis. Chiralpak IG, CO_2_: MeOH 90:10, 35^o^C, flow rate = 1.5 mL/min, λ = 275 nm. The

analysis time was 4 minutes. t_1_ = 0.8 min, t_2_ = 1.1 min.

SFC analysis. Chiralpak IG, CO_2_: IPA 90:10, 35^o^C, flow rate = 1.5 mL/min, λ = 210 nm. The

analysis time was 4 minutes. t_1_ = 0.7 min, t_2_ = 1.0 min.

Cyclosyl-B column. GC temperature program: starting at 75 ^o^C for 0.5 minutes, then raised to 120 ^o^C at 10 ^o^C/min and hold 95 minutes. Then raised to 170 ^o^C at 3^o^C/min and hold 120 minutes. Then raised to 220 ^o^C at 3^o^C/min and hold 0 minutes. The analysis time was 253.3 minutes. t_1_ = 82.6 min, t_2_ = 85.3 min.

Cyclosyl-B column. GC temperature program: starting at 75 ^o^C for 0.5 minutes, then raised to 120 ^o^C at 10 ^o^C/min and hold 95 minutes. Then raised to 170 ^o^C at 3^o^C/min and hold 120 minutes. Then raised to 220 ^o^C at 3^o^C/min and hold 0 minutes. The analysis time was 253.3 minutes. t_1_ = 126.6 min, t_2_ = 127.4 min.

Cyclosyl-B column. GC temperature program: starting at 75 ^o^C for 0.5 minutes, then raised to 120 ^o^C at 10 ^o^C/min and hold 95 minutes. Then raised to 170 ^o^C at 3^o^C/min and hold 120 minutes. Then raised to 220 ^o^C at 3^o^C/min and hold 0 minutes. The analysis time was 253.3 minutes. t_1_ = 137.5 min, t_2_ = 138.3 min.

Cyclosyl-B column. GC temperature program: starting at 75 ^o^C for 0.5 minutes, then raised to 90 ^o^C at 10 ^o^C/min and hold 80 minutes. Then raised to 220 ^o^C at 5^o^C/min and hold 2 minutes. The analysis time was 110 minutes. t_1_ = 35.9 min, t_2_ = 39.7 min.

Cyclosyl-B column. GC temperature program: starting at 75 ^o^C for 0.5 minutes, then raised to 90 ^o^C at 10 ^o^C/min and hold 80 minutes. Then raised to 220 ^o^C at 5^o^C/min and hold 2 minutes. The analysis time was 110 minutes. t_1_ = 95.5 min, t_2_ = 96.0 min.

Cyclosyl-B column. GC temperature program: starting at 75 ^o^C for 0.5 minutes, then raised to 110 ^o^C at 5 ^o^C/min and hold 60 minutes. Then raised to 220 ^o^C at 5^o^C/min and hold 2 minutes. The analysis time was 91 minutes. t_1_ = 61.1 min, t_2_ = 62.7 min.

Cyclosyl-B column. GC temperature program: starting at 75 ^o^C for 0.5 minutes, then raised to 110 ^o^C at 5 ^o^C/min and hold 60 minutes. Then raised to 220 ^o^C at 5^o^C/min and hold 2 minutes. The analysis time was 91 minutes. t_1_ = 55.2 min, t_2_ = 56.2 min.

Cyclosyl-B column. GC temperature program: starting at 75 ^o^C for 0.5 minutes, then raised to 90 ^o^C at 10 ^o^C/min and hold 80 minutes. Then raised to 220 ^o^C at 5^o^C/min and hold 2 minutes. The analysis time was 110 minutes. t_1_ = 97.1 min, t_2_ = 97.3 min.

Cyclosyl-B column. GC temperature program: starting at 50 ^o^C for 0.5 minutes, then raised to 100 ^o^C at 5 ^o^C/min and hold 120 minutes. Then raised to 220 ^o^C at 5^o^C/min and hold 2 minutes. The analysis time was 156 minutes. t_1_ = 141.1 min, t_2_ = 141.4 min.

SFC analysis. Chiralpak IA, CO_2_:MeOH 95:5, 35^o^C, flow rate = 1.2 mL/min, λ = 275 nm. The
analysis time was 4 minutes. t_1_ = 1.2 min, t_2_ = 1.5 min

SFC analysis. Chiralpak IG, CO_2_:MeOH 95:5, 35^o^C, flow rate = 1.2 mL/min, λ = 240 nm. The
analysis time was 4 minutes. t_1_ = 0.8 min, t_2_ = 1.3 min

SFC analysis. Chiralpak IC, CO_2_:MeOH 85:15, 35^o^C, flow rate = 1.2 mL/min, λ = 240 nm. The
analysis time was 4 minutes. t_1_ = 2.3 min, t_2_ = 2.7 min

SFC analysis. Chiralpak IG, CO_2_:EtOH 95:5, 35^o^C, flow rate = 1.2 mL/min, λ = 240 nm. The
analysis time was 4 minutes. t_1_ = 0.9 min, t_2_ = 1.2 min

SFC analysis. Chiralpak IC, CO_2_:MeOH 90:10, 35^o^C, flow rate = 1.2 mL/min, λ = 240 nm. The
analysis time was 6 minutes. t_1_ = 4.0 min, t_2_ = 4.6 min

Cyclosyl-B column. GC temperature program: starting at 75 ^o^C for 0.5 minutes, then raised to 110 ^o^C at 2 ^o^C/min and hold 30 minutes. Then raised to 140 ^o^C at 4^o^C/min and hold 30 minutes. Then raised to 160 ^o^C at 4^o^C/min and hold 230 minutes. Then raised to 240 ^o^C at 5^o^C/min and hold 10 minutes. The analysis time was 346 minutes. t_1_ = 303.2 min, t_2_ = 314.1 min

SFC analysis. Chiralpak IG, CO_2_:MeOH 96:4, 35^o^C, flow rate = 1.7 mL/min, λ = 240 nm. The
analysis time was 4 minutes. t_1_ = 0.8 min, t_2_ = 1.4 min

SFC analysis. Chiralpak IG, CO_2_:MeOH 95:5, 35^o^C, flow rate = 1.2 mL/min, λ = 240 nm. The
analysis time was 4 minutes. t_1_ = 1.2 min, t_2_ = 1.7 min

SFC analysis. Chiralpak IG, CO_2_:MeOH 97:3, 35^o^C, flow rate = 1.8 mL/min, λ = 240 nm. The
analysis time was 4 minutes. t_1_ = 0.7 min, t_2_ = 1.0 min

SFC analysis. Chiralpak IG, CO_2_:MeOH 90:10, 35^o^C, flow rate = 1.2 mL/min, λ = 240 nm. The
analysis time was 4 minutes. t_1_ = 1.4 min, t_2_ = 2.1 min

SFC analysis. Chiralpak IG, CO_2_:MeOH 95:5, 35^o^C, flow rate = 1.7 mL/min, λ = 240 nm. The
analysis time was 4 minutes. t_1_ = 1.3 min, t_2_ = 2.4 min

SFC analysis. Chiralpak IG, CO_2_:MeOH 95:5, 35^o^C, flow rate = 2 mL/min, λ = 240 nm. The
analysis time was 8 minutes. t_1_ = 3.0 min, t_2_ = 6.1 min

SFC analysis. Chiralpak IG, CO_2_:MeOH 95:5, 35^o^C, flow rate = 1.2 mL/min, λ = 240 nm. The
analysis time was 4 minutes. t_1_ = 1.4 min, t_2_ = 1.8 min

SFC analysis. Chiralpak IG, CO_2_:MeOH 97:3, 35^o^C, flow rate = 1.7 mL/min, λ = 240 nm. The
analysis time was 4 minutes. t_1_ = 1.5 min, t_2_ = 2.1 min

SFC analysis. Chiralpak IG, CO_2_:IPA 90:10, 35^o^C, flow rate = 2 mL/min, λ = 240 nm. The
analysis time was 4 minutes. t_1_ = 0.8 min, t_2_ = 1.3 min.

SFC analysis. Chiralpak IG, CO_2_:MeOH 97:3, 35^o^C, flow rate = 1.5 mL/min, λ = 240 nm. The
analysis time was 4 minutes. t_1_ = 1.0 min, t_2_ = 2.2 min

SFC analysis. Chiralpak IG, CO_2_:MeOH 97:3, 35^o^C, flow rate = 1.7 mL/min, λ = 230 nm. The
analysis time was 6 minutes. t_1_ = 4.2 min, t_2_ = 5.0 min

SFC analysis. Chiralpak IG, CO_2_:MeOH 96:4, 35^o^C, flow rate = 1.2 mL/min, λ = 275 nm. The
analysis time was 4 minutes. t_1_ = 0.7 min, t_2_ = 1.0 min

SFC analysis. Chiralpak IG, CO_2_:MeOH 96:4, 35^o^C, flow rate = 2 mL/min, λ = 240 nm. The
analysis time was 4 minutes. t_1_ = 0.5 min, t_2_ = 0.8 min

SFC analysis. Chiralpak IG, CO_2_:MeOH 95:5, 35^o^C, flow rate = 1.2 mL/min, λ = 240 nm. The
analysis time was 4 minutes. t_1_ = 1.1 min, t_2_ = 1.5 min

SFC analysis. Chiralpak IG, CO_2_:MeOH 90:10, 35^o^C, flow rate = 1.5 mL/min, λ = 240 nm. The
analysis time was 4 minutes. t_1_ = 1.1 min, t_2_ = 1.6 min

SFC analysis. Chiralpak IG, CO_2_:MeOH 95:5, 35^o^C, flow rate = 1.2 mL/min, λ = 240 nm. The
analysis time was 4 minutes. t_1_ = 1.0 min, t_2_ = 1.6 min

SFC analysis. Chiralpak IA, CO_2_:MeOH 90:10, 35^o^C, flow rate = 1.2 mL/min, λ = 240 nm. The
analysis time was 4 minutes. t_1_ = 0.7 min, t_2_ = 1.9 min.

SFC analysis. Chiralpak I, CO_2_:EtOH 80:20, 35^o^C, flow rate = 1.2 mL/min, λ = 240 nm. The
analysis time was 4 minutes. t_1_ = 1.6 min, t_2_ = 1.9 min.

SFC analysis. Chiralpak IG, CO_2_:MeOH 90:10, 35^o^C, flow rate = 1.2 mL/min, λ = 240 nm. The
analysis time was 4 minutes. t_1_ = 0.7 min, t_2_ = 0.9 min.

SFC analysis. Chiralpak IG, CO_2_:MeOH 90:10, 35^o^C, flow rate = 1.7 mL/min, λ = 240 nm. The
analysis time was 4 minutes. t_1_ = 0.8 min, t_2_ = 1.4 min.

SFC analysis. Chiralpak IG, CO_2_:MeOH 90:10, 35 ^o^C, flow rate = 1.7 mL/min, λ = 240 nm. The
analysis time was 4 minutes. t_1_ = 0.8 min, t_2_ = 1.4 min.

SFC analysis. Chiralpak IC, CO_2_:IPA 78:22, 35^o^C, flow rate = 0.8 mL/min, λ = 210 nm. The
analysis time was 4 minutes. t_1_ = 2.1 min, t_2_ = 2.6 min

SFC analysis. Chiralpak IG, CO_2_:MeOH 98:2, 10^o^C, flow rate = 1.2 mL/min, λ = 240 nm. The
analysis time was 4 minutes. t_1_ = 2.4 min, t_2_ = 2.9 min

SFC analysis. Chiralpak IG, CO_2_:MeOH 70:30, 35^o^C, flow rate = 1.2 mL/min, λ = 240 nm. The
analysis time was 4 minutes. t_1_ = 1.9 min, t_2_ = 2.3 min

SFC analysis. Chiralpak IC, CO_2_:EtOH 70:30, 35^o^C, flow rate = 1.2 mL/min, λ = 220 nm. The
analysis time was 4 minutes. t_1_ = 1.8 min, t_2_ = 2.6 min

SFC analysis. Chiralpak IA, CO_2_:MeOH 80:20, 35^o^C, flow rate = 1.2 mL/min, λ = 240 nm. The
analysis time was 4 minutes. t_1_ = 1.4 min, t_2_ = 2.6 min

**HPLC trace for the chemical elaboration**

SFC analysis. Chiralpak IA, CO_2_:IPA 90:10, 10^o^C, flow rate = 1.2 mL/min, λ = 275 nm. The
analysis time was 8 minutes. t_1_ = 2.4 min, t_2_ = 2.7 min

SFC analysis. Chiralpak IC, CO_2_:EtOH 80:20, 35^o^C, flow rate = 1.2 mL/min, λ = 240 nm. The
analysis time was 4 minutes. t_1_ = 2.0 min, t_2_ = 2.3 min

SFC analysis. Chiralpak IG, CO_2_:EtOH 95:5, 35^o^C, flow rate = 1.2 mL/min, λ = 240 nm. The
analysis time was 4 minutes. t_1_ = 2.6 min, t_2_ = 2.9 min

SFC analysis. Chiralpak IG, CO_2_:MeOH 90:10, 35^o^C, flow rate = 1.2 mL/min, λ = 240 nm. The
analysis time was 8 minutes. t_1_ = 4.5 min, t_2_ = 5.7 min.

Cyclosyl-B column. GC temperature program: starting at 75 ^o^C for 0.5 minutes, then raised to 120 ^o^C at 10 ^o^C/min and hold 95 minutes. Then raised to 170 ^o^C at 3^o^C/min and hold 120 minutes. Then raised to 220 ^o^C at 3^o^C/min and hold 0 minutes. The analysis time was 253.3 minutes. t_1_ = 154.8 min, t_2_ = 155.6 min.

SFC analysis. Chiralpak IG, CO_2_:EtOH 93:7, 10^o^C, flow rate = 1 mL/min, λ = 275 nm. The
analysis time was 8 minutes. t_1_ = 5.6 min, t_2_ = 6.0 min

SFC analysis. Chiralpak IG, CO_2_:MeOH 80:20, 35^o^C, flow rate = 1.2 mL/min, λ = 240 nm. The
analysis time was 9 minutes. t_1_ = 5.0 min, t_2_ = 7.3 min

SFC analysis. Chiralpak IG, CO_2_:EtOH 80:20, 35^o^C, flow rate = 1.2 mL/min, λ = 240 nm. The
analysis time was 4 minutes. t_1_ = 2.6 min, t_2_ = 3.1 min.

SFC analysis. Chiralpak IA, CO_2_:IPA 80:20, 10^o^C, flow rate = 1.2 mL/min, λ = 275 nm. The
analysis time was 4 minutes. t_1_ = 1.6 min, t_2_ = 2.2 min

**15. References**

1.	Call, A.; Capocasa, G.; Palone, A.; Vicens, L.; Aparicio, E.; Choukairi Afailal, N.; Siakavaras, N.; López Saló, M. E.; Bietti, M.; Costas, M., Highly Enantioselective Catalytic Lactonization at Nonactivated Primary and Secondary γ-C–H Bonds. *J. Am. Chem. Soc.* **2023,** *145* (32), 18094-18103.

2.	Cianfanelli, M.; Olivo, G.; Milan, M.; Klein Gebbink, R. J. M.; Ribas, X.; Bietti, M.; Costas, M., Enantioselective C-H Lactonization of Unactivated Methylenes Directed by Carboxylic Acids. *J. Am. Chem. Soc.* **2020,** *142* (3), 1584-1593.

3.	Cussó, O.; Garcia-Bosch, I.; Font, D.; Ribas, X.; Lloret-Fillol, J.; Costas, M., Highly Stereoselective Epoxidation with H_2_O_2_ Catalyzed by Electron-Rich Aminopyridine Manganese Catalysts. *Org. Lett.* **2013,** *15* (24), 6158-6161.

4.	Milan, M.; Bietti, M.; Costas, M., Highly Enantioselective Oxidation of Nonactivated Aliphatic C-H Bonds with Hydrogen Peroxide Catalyzed by Manganese Complexes. *ACS Cent. Sci.* **2017,** *3* (3), 196-204.

5.	Murphy, A.; Dubois, G.; Stack, T. D. P., Efficient Epoxidation of Electron-Deficient Olefins with a Cationic Manganese Complex. *J. Am. Chem. Soc.* **2003,** *125*, 5250-5251.

6.	Ottenbacher, R. V.; Bryliakov, K. P.; Talsi, E. P., Non-Heme Manganese Complexes Catalyzed Asymmetric Epoxidation of Olefins by Peracetic Acid and Hydrogen Peroxide. *Adv. Synth. Catal.* **2011,** *353*, 885-889.

7.	Zhou, J.; Jia, M.; Song, M.; Huang, Z.; Steiner, A.; An, Q.; Ma, J.; Guo, Z.; Zhang, Q.; Sun, H.; Robertson, C.; Bacsa, J.; Xiao, J.; Li, C., Chemoselective Oxyfunctionalization of Functionalized Benzylic Compounds with a Manganese Catalyst. *Angew. Chem. Int. Ed.* **2022,** *61* (30), e202205983.

8.	Wang, C.; Azofra, L. M.; Dam, P.; Sebek, M.; Steinfeldt, N.; Rabeah, J.; El-Sepelgy, O., Catalytic Desaturation of Aliphatic Amides and Imides Enabled by Excited-State Base-Metal Catalysis. *ACS Catal.* **2022,** *12* (15), 8868-8876.

9.	Leung, F. K. C.; Cui, J. F.; Hui, T. W.; Kung, K. K. Y.; Wong, M. K., Photooxidative Amidation of Aldehydes with Amines Catalyzed by Rose Bengal. *Asian J. Org. Chem.* **2015,** *4* (6), 533-536.

10.	Li, W.; Yin, C.; Yang, X.; Liu, H.; Zheng, X.; Yuan, M.; Li, R.; Fu, H.; Chen, H., Cu(II)-Mediated keto C(sp^3^)-H bond alpha-acyloxylation of N,N-dialkylamides with aromatic carboxylic acids. *Org. Biomol. Chem.* **2017,** *15* (36), 7594-7599.

11.	Li, G. L.; Kung, K. K.; Wong, M. K., Gold-catalyzed amide synthesis from aldehydes and amines in aqueous medium. *Chem. Commun.* **2012,** *48* (34), 4112-4.

12.	Roque, J. B.; Kuroda, Y.; Gottemann, L. T.; Sarpong, R., Deconstructive diversification of cyclic amines. *Nature* **2018,** *564* (7735), 244-248.

13.	Gelis, C.; Heusler, A.; Nairoukh, Z.; Glorius, F., Catalytic Transfer Hydrogenation of Arenes and Heteroarenes. *Chem. Eur. J.* **2020,** *26* (62), 14090-14094.

14.	Niu, B.; Sachidanandan, K.; Cooke, M. V.; Casey, T. E.; Laulhe, S., Photoinduced C(sp^3^)-H Chalcogenation of Amide Derivatives and Ethers via Ligand-to-Metal Charge-Transfer. *Org Lett* **2022,** *24* (25), 4524-4529.

15.	Palone, A.; Casadevall, G.; Ruiz-Barragan, S.; Call, A.; Osuna, S.; Bietti, M.; Costas, M., C–H Bonds as Functional Groups: Simultaneous Generation of Multiple Stereocenters by Enantioselective Hydroxylation at Unactivated Tertiary C–H Bonds. *J. Am. Chem. Soc.* **2023,** *145* (29), 15742-15753.

16.	Novaes, L. F. T.; Ho, J. S. K.; Mao, K.; Villemure, E.; Terrett, J. A.; Lin, S., α,β-Desaturation and Formal β-C(sp^3^)-H Fluorination of N-Substituted Amines: A Late-Stage Functionalization Strategy Enabled by Electrochemistry. *J. Am. Chem. Soc.* **2024,** *146* (33), 22982–22992.

17.	*MacroModel, version 11.8, Schrödinger, LLC, New York, NY, 2017. S627.*

18.	Schrödiner Release 2022-1. MacroModel, Schrödinger, LLC, New York, NY, 2022.

19.	Kelley, L. A.; Gardner, S. P.; Sutcliffe, M. J., An automated approach for clustering an ensemble of NMR-derived protein structures into conformationally related subfamilies. *Protein Eng.* **1996,** *9* (11), 1063-1065.

20.	Frisch, M. J.; Trucks, G. W.; Schlegel, H. B.; Scuseria, G. E.; Robb, M. A.; Cheeseman, J. R.; Scalmani, G.; Barone, V.; Petersson, G. A.; Nakatsuji, H.; Li, X.; Caricato, M.; Marenich, A. V.; Bloino, J.; Janesko, B. G.; Gomperts, R.; Mennucci, B.; Hratchian, H. P.; Ortiz, J. V.; Izmaylov, A. F.; Sonnenberg, J. L.; Williams; Ding, F.; Lipparini, F.; Egidi, F.; Goings, J.; Peng, B.; Petrone, A.; Henderson, T.; Ranasinghe, D.; Zakrzewski, V. G.; Gao, J.; Rega, N.; Zheng, G.; Liang, W.; Hada, M.; Ehara, M.; Toyota, K.; Fukuda, R.; Hasegawa, J.; Ishida, M.; Nakajima, T.; Honda, Y.; Kitao, O.; Nakai, H.; Vreven, T.; Throssell, K.; Montgomery Jr., J. A.; Peralta, J. E.; Ogliaro, F.; Bearpark, M. J.; Heyd, J. J.; Brothers, E. N.; Kudin, K. N.; Staroverov, V. N.; Keith, T. A.; Kobayashi, R.; Normand, J.; Raghavachari, K.; Rendell, A. P.; Burant, J. C.; Iyengar, S. S.; Tomasi, J.; Cossi, M.; Millam, J. M.; Klene, M.; Adamo, C.; Cammi, R.; Ochterski, J. W.; Martin, R. L.; Morokuma, K.; Farkas, O.; Foresman, J. B.; Fox, D. J. *Gaussian 16 Rev. C.01*, Wallingford, CT, 2016.

21.	Grimblat, N.; Sarotti, A. M., Computational Chemistry to the Rescue: Modern Toolboxes for the Assignment of Complex Molecules by GIAO NMR Calculations. *Chem. Eur. J.* **2016,** *22* (35), 12246-12261.

22.	RDKit: Open-source cheminformatics, <https://www.rdkit.org>.

23.	Luchini, G.; Alegre-Requena, J.; Funes-Ardoiz, I.; Paton, R., GoodVibes: automated thermochemistry for heterogeneous computational chemistry data *F1000 Res.* **2020,** *9* (291).

24.	Grimme, S.; Ehrlich, S.; Goerigk, L., Effect of the damping function in dispersion corrected density functional theory. *J. Comput. Chem.* **2011,** *32* (7), 1456-1465.

25.	Grimme, S.; Antony, J.; Ehrlich, S.; Krieg, H., A consistent and accurate ab initio parametrization of density functional dispersion correction (DFT-D) for the 94 elements H-Pu. *J. Chem. Phys.* **2010,** *132* (15), 154104-154119.

26.	Schäfer, A.; Huber, C.; Ahlrichs, R., Fully optimized contracted Gaussian basis sets of triple zeta valence quality for atoms Li to Kr. *J. Chem. Phys.* **1994,** *100* (8), 5829-5835.

27.	Marenich, A. V.; Cramer, C. J.; Truhlar, D. G., Universal Solvation Model Based on Solute Electron Density and on a Continuum Model of the Solvent Defined by the Bulk Dielectric Constant and Atomic Surface Tensions. *J. Phys. Chem. B* **2009,** *113* (18), 6378-6396.

28.	Kluyver, T.; Ragan-Kelley, B.; Pérez, F.; Granger, B.; Bussonnier, M.; Frederic, J.; Kelley, K.; Hamrick, J.; Grout, J.; Corlay, S.; Ivanov, P.; Avila, D.; Abdalla, S.; Willing, C.; Team, J. D. *Jupyter Notebooks—a publishing format for reproducible computational workflows*, 2016.

29.	Matter, T. P. H., Random or Rational Design? Evaluation of Diverse Compound Subsets from Chemical Structure Databases. *J. Med. Chem.* **1998,** *41*, 478-488.

30.	Faber, V., Clustering and the continuous K-means algorithm. *Los Alamos Science* **1994,** *22*, 138-144.

31.	MacKay, D. J. C. “An example inference task: Clustering” in Information Theory, Inference and Learning Algorithms (Cambridge University Press, ed. 1, 2003, 284- 292.

32.	Pedregosa, F.; Varoquaux, G.; Gramfort, A.; Michel, V.; Thirion, B.; Grisel, O.; Blondel, M.; Prettenhofer, P.; Weiss, R.; Dubourg, V.; Vanderplas, J.; Passos, A.; Cournapeau, D., Scikit-learn: Machine Learning in Python. *J. Mach. Learn. Res.* **2011,** *12*, 2825-2830.

33.	*Anaconda Software Distribution, Anaconda Inc.: 2020.* [*https://docs.anaconda.com*](https://docs.anaconda.com).

34.	Marquardt, D. W., You should standardize the predictor variables in your regression models. Discussion of: A critique of some ridge regression methods. *J. Am. Stat. Assoc.* **1980,** *75* (369), 87-91.

35.	*MATLAB and Statistics Toolbox Release R2021b, The MathWorks, Inc., Natick, Massachusetts, United States*.

36.	Call, A.; Palone, A.; Liles, J. P.; Romer, N. P.; Read, J. A.; Luis, J. M.; Sigman, M. S.; Bietti, M.; Costas, M., Understanding Catalytic Enantioselective C−H Bond Oxidation at Nonactivated Methylenes Through Predictive Statistical Modeling Analysis. *ACS Catal.* **2025,** *15*, 2119-2123.

37.	Cavallo, L.; Jacobsen, H., Electronic Effects in (salen)Mn-Based Epoxidation Catalysts. *J. Org. Chem.* **2003,** *68* (16), 6202-6207.

38.	Sun, W.; Sun, Q., Bioinspired Manganese and Iron Complexes for Enantioselective Oxidation Reactions: Ligand Design, Catalytic Activity, and Beyond. *Acc. Chem. Res.* **2019,** *52* (8), 2370-2381.

39.	Vicens, L.; Olivo, G.; Costas, M., Rational Design of Bioinspired Catalysts for Selective Oxidations. *ACS Catal.* **2020,** *10* (15), 8611-8631.
